# Supplementary material for: Asymmetric formal sp2-hydrocarbonations of dienes and alkynes via palladium hydride catalysis
Source: Nat Commun. 2023 Oct 9;14:6303. doi: 10.1038/s41467-023-42160-2 (PMC10562392; doi:10.1038/s41467-023-42160-2)
Supplement: Supplementary file 1 — Supplementary information [file 41467_2023_42160_MOESM1_ESM.pdf]

Supplementary Information for

Asymmetric Formal sp^2-Hydrocarbonations of Dienes and Alkynes via Palladium Hydride Catalysis

Ming-Qiao Tang[1#], Zi-Jiang Yang[1#], Zhi-Tao He[1,2]*

[1]CAS Key Laboratory of Synthetic Chemistry of Natural Substances, Shanghai Institute of Organic Chemistry, University of Chinese Academy of Sciences, Shanghai 200032, China.

[2]School of Chemistry and Materials Science, Hangzhou Institute for Advanced Study, University of Chinese Academy of Sciences, Hangzhou 310024, China.

[#]These authors contributed equally: M.-Q. Tang, Z.-J. Yang

Supplementary Methods……………………………………………………………….…..…S2

1. General information ……..………………….................................……………………………..…S2

2. Synthesis of substrates ………………….........................…………………………...……..……S2

3. Development of reaction conditions…………………………..........................………………..…S4

4. General procedure for formal hydroalkenylation...…S7

5. General procedure for formal hydroallenylation………………………………..….........……...S19

6. General procedure for formal hydroketenimination......……………………………………..…...S24

7. General procedure for hydroalkenylation of alkynes………...…………..........................……...S27

8. Mechanistic studies……………………………………...…………...............................………...S33

9. Convergent and stereodivergent synthesis…...…………..S38

10. X-ray structure of **4t**…….………………………………..……….……………...……………...S41

11. Copies of ^{1}H NMR, ^{13}C NMR, ^{19}F NMR and ^{31}P NMR spectra…..…....……………………….…..….S43

Supplementary References………...……………………………..……………………….…...……S98

Supplementary Methods

1. General information

All air-sensitive procedures were conducted by Schlenk techniques under argon. Unless otherwise indicated, all commercially available starting materials and dry solvents were purchased and used directly without further purification. ^{1}H, ^{13}C and ^{19}F NMR spectra were acquired on 400 MHz Bruker or 500 MHz Agilent instruments at Shanghai Institute of Organic Chemistry. For High-resolution mass spectra: ESI mass spectra were recorded on Thermo Scientific Q Exactive HF Orbitrap-FTMS; MALDI was measured on Voyager-DE STR; EI mass spectra were recorded on Waters Premier GC-TOF MS; FI mass spectra were recorded on JEOL-AccuTOF-GCv4G-GCT MS. Optical rotation was measured using a 1 mL cell with 1.0 dm path length on a JASCO P-1030 polarimeter. HPLC analysis was conducted on a Shimadzu HPLC system equipped with Daicel or Chiralpak chiral-stationary-phase columns (ϕ 4.6 mm×250 mm). Chemical shifts are reported in δ (ppm) referenced to an internal TMS standard (0 ppm) or $CHCl_3$ in $CDCl_3$ (7.26 ppm) for ^{1}H NMR, $CDCl_3$ (δ = 77.10 ppm) for ^{13}C NMR, and $CFCl_3$ (0 ppm) for ^{19}F NMR. Coupling constants (*J*) are reported in Hz. Multiplicities are reported using the following abbreviations: br = broad, s = singlet, d = doublet, t = triplet, q = quartet, m = multiplet. Column chromatography was performed with 300-400 mesh silica gel using flash column chromatography technique. The X-ray crystal structure was measured on Bruker D8 VENTURE.

2. Synthesis of substrates

2.1 Synthesis of (*E*)-dienes

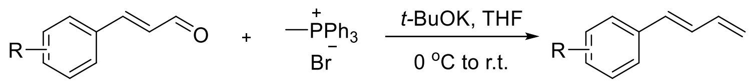

15 **16** **1**

General procedure: to a Schlenk tube with methyltriphenylphosphonium bromide **16** (1.6 g, 4.4 mmol) and *t*-BuOK (0.54 g, 4.8 mmol) was added dry THF (10 mL) at 0 °C under nitrogen. Then the reaction was stirred at 0 ºC for 30 min. Next aldehyde **15** (4.0 mmol) was added to the reaction. The resulting mixture was gradually allowed to warm to room temperature and stirred for 12 h. After this time, the reaction was quenched by saturated NH_4Cl aqueous solution (5 mL) and extracted with *n*-hexane (10 × 3 mL). The combined organic extracts were washed with brine (10 mL), dried over anhydrous Na_2SO_4, filtered, condensed and purified by flash column chromatography to give the pure diene (*E*)-**1**.

2.2 Synthesis of dienes as E/Z mixture

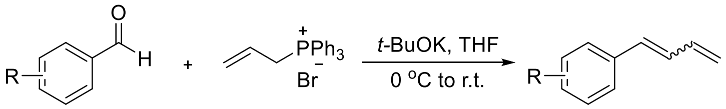

17 **18** **1**

General procedure: to a Schlenk tube with allyltriphenylphosphonium bromide **18** (1.7 g, 4.4 mmol) and *t*-BuOK (0.54 g, 4.8 equiv) was added dry THF (10 mL) at 0 °C under nitrogen. Then the reaction was stirred at 0 ºC for 30 min. Next aldehyde **17** (4.0 mmol) was added to the reaction. The resulting

mixture was gradually allowed to warm to room temperature and stirred for 12 h. After this time, the reaction was quenched by saturated NH_4Cl aqueous solution (5.0 mL) and extracted with *n*-hexane (10 × 3 mL). The combined organic extracts were washed with brine (10 mL), dried over anhydrous Na_2SO_4, filtered, condensed and purified by flash column chromatography to give diene **1** as Z/E mixture.

Note: all these diene substrates below are known[1-5].

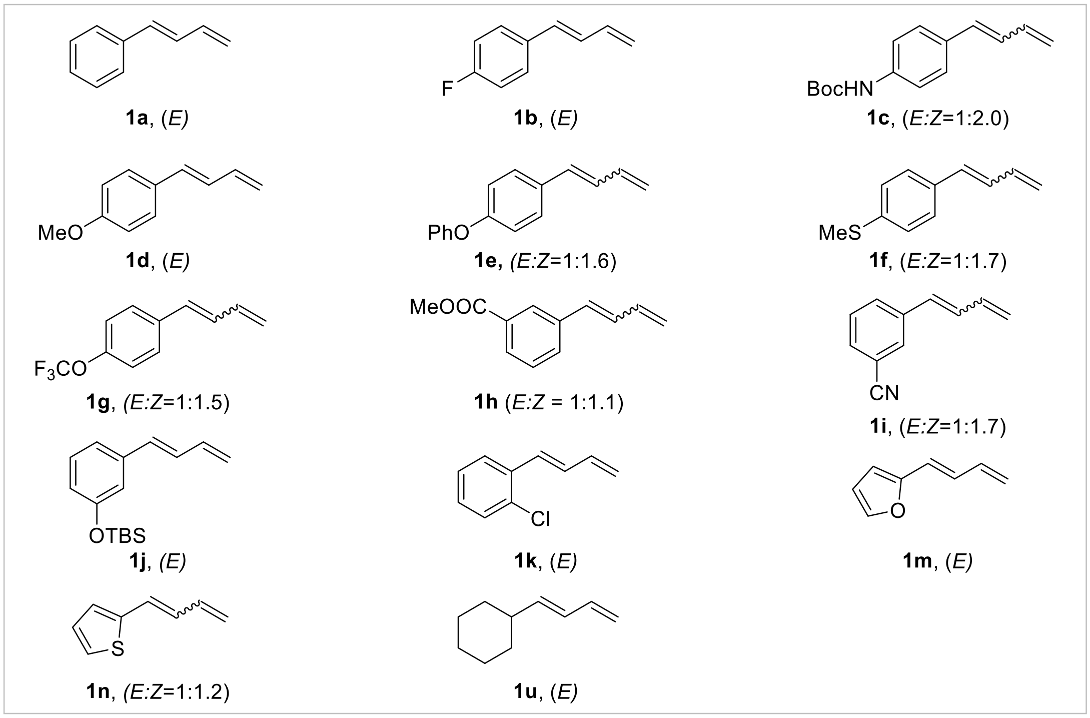

Supplementary Figure 1. Diene substrates prepared

2.3 Synthesis of alkynes

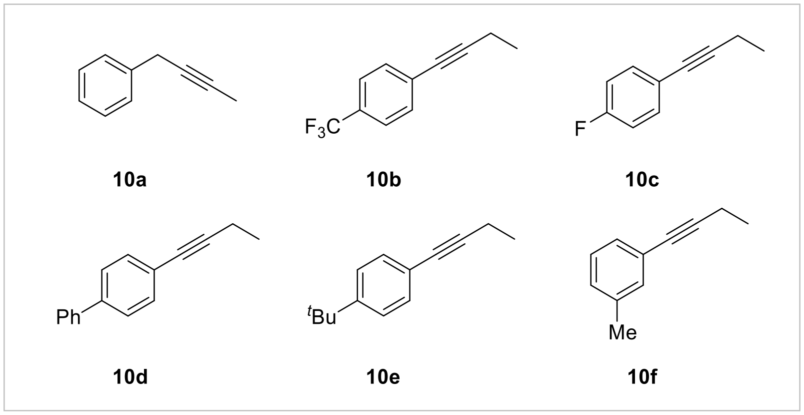

Supplementary Figure 2. Alkyne substrates prepared

Aryl alkynes including **10a-10f** are known and prepared based on reported methods[6].

3. Development of reaction conditions

Supplementary Table 1. Evaluation of ligands for hydroalkenylation of 1,3-dienes

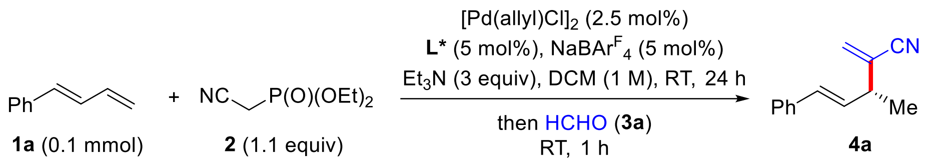

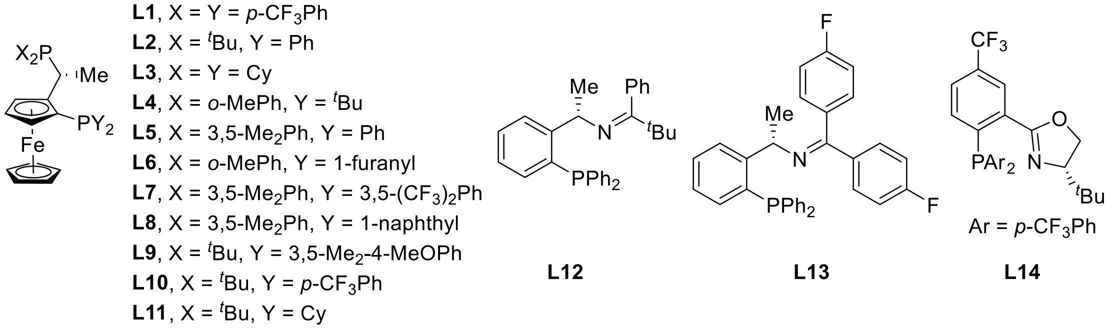

Entry[a]	L	Yield (%)[b]	Ee (%)[c]
1	**L1**	trace	
2	**L2**	trace	
3	**L3**	trace	
4	**L4**	trace	
5	**L5**	trace	
6	**L6**	trace	
7	**L7**	trace	
8	**L8**	trace	
9	**L9**	54	84
10	**L10**	46	86
11	**L11**	50	94
12	**L12**	trace	
13	**L13**	trace	
14	**L14**	trace	

[a]The reaction was carried out with HCHO (37% aqueous solution, 12 equiv) for the second step. $NaBAr^F_4$, Ar^F = 3,5-$(CF_3)_2$Ph. [b]Yield was determined by ^{1}H NMR with 1,3,5-trimethoxybenzene as the internal standard. [c]Determined by HPLC analysis.

Supplementary Table 2. Evaluation of additional conditions for hydroalkenylation of 1,3-dienes

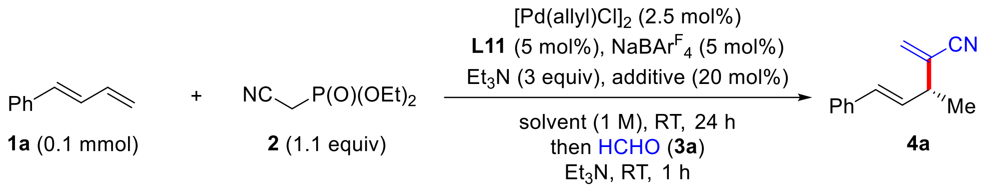

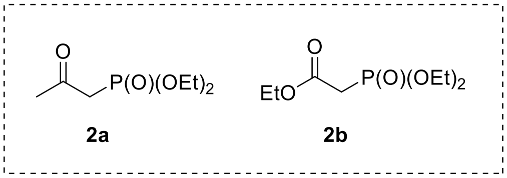

Entry[a]	Solvent	Additive	Yield (%)[b]	Ee (%)[c]
1	MeCN	/	24	94
2	THF	/	/	
3	$PhCF_3$	/	36	94
4	*n*-hexane	/	24	94
5	DCM	$Ph_2P(O)OH$	68	95
6[d]	DCM	$Et_3N{\cdot}HBAr^F{}_4$	/	
7[e]	DCM	$Ph_2P(O)OH$	82[h]	94
8[e,f]	DCM	$Ph_2P(O)OH$	trace	
9[e,g]	DCM	$Ph_2P(O)OH$	/	

[a]The reaction was carried out with HCHO (37% aqueous solution, 12 equiv) for the second step. $NaBAr^F{}_4$, Ar^F = 3,5-$(CF_3)_2$Ph. [b]Yield was determined by ^{1}H NMR with 1,3,5-trimethoxybenzene as the internal standard. [c]Determined by HPLC analysis. [d]$Et_3N{\cdot}HBAr^F{}_4$ (7 mol%) was used. [e]**2**(2.0 equiv) and DCM (2 M) for 48 h were adopted for first step. Additional Et_3N (1.0 equiv) was used for the second Wittig reaction. [f]**2a** was used instead of **2**. [g]**2b** was used instead of **2**. [h]Isolated yield.

Supplementary Table 3. Condition evaluations for hydroallenylation of 1,3-dienes

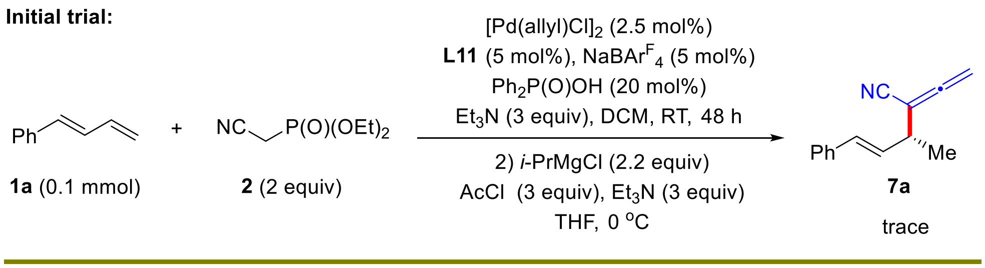

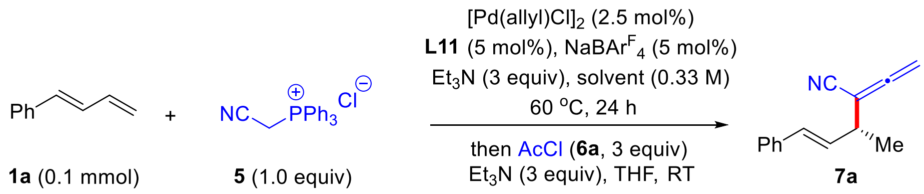

Entry	solvent	T/°C	Yield (%)[a]	Ee (%)[b]
1	DCM	60	62	91
2[c]	DCM	60	20	85
3	MeCN	60	71	86
4	DMF	60	62	90
5	MeOH	60	trace	
6	THF	60	68	80
7	$PhCF_3$	60	71	88
8	DCE	60	60	91
9[d]	DCM/Et_3N (1/2)	60	66	94
10[d]	DCM/Et_3N (2/1)	60	47	93
11[d]	DCM/Et_3N (1/1)	60	49	95
12[d]	DCM/Et_3N (1/1)	50	61	95
13[d,e]	DCM/Et_3N (1/1)	50	83	94

[a]Yield was determined by ^{1}H NMR with 1,3,5-trimethoxybenzene as the internal standard. [b]Determined by HPLC analysis. [c]Br^- anion for **5** was used. [d]Et_3N (3 equiv) was not used for the first step, but DCM/Et_3N as the mixed solvents were adopted. [f]DCM/Et_3N (0.67 M) for 36 h were adopted for the first step. Isolated yield. $NaBAr^F_4$, Ar^F = 3,5-$(CF_3)_2$Ph.

Supplementary Table 4. Condition evaluations for hydroalkenylation of alkynes

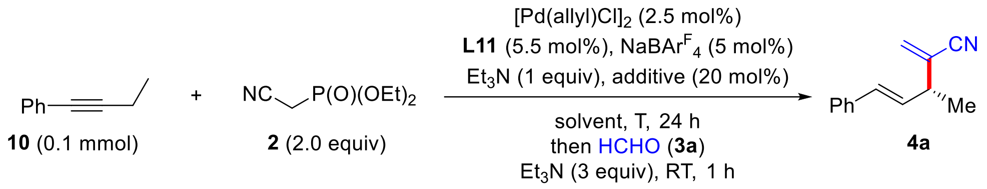

Entry[a]	Solvent	T/°C	Additive	Yield (%)[b]	Ee (%)[c]
1	*n*-hexane (2 M)	60	$Ph_2P(O)OH$	13	82
2	$PhCF_3$ (2 M)	60	$Ph_2P(O)OH$	11	86
3	DMF (2 M)	60	$Ph_2P(O)OH$	14	88
4	DCE (2 M)	60	$Ph_2P(O)OH$	7	88
5	DCM (2 M)	60	$Ph_2P(O)OH$	4	90
6	^{i}PrOH (2 M)	60	$Ph_2P(O)OH$	36	83
7	MeCN (2 M)	60	$Ph_2P(O)OH$	20	88
8	MeOH (2 M)	60	$Ph_2P(O)OH$	88	83
9	MeOH (2 M)	70	$Ph_2P(O)OH$	90	83
10	MeOH (0.5 M)	70	$Ph_2P(O)OH$	52	90
11	MeOH (0.5 M)	80	$Ph_2P(O)OH$	64	89
12	MeOH (0.5 M)	70	$Ph_2CHCOOH$	71	88
13	MeOH (0.5 M)	70	adamantoic acid	77	88
14	MeOH (0.5 M)	70	phthalic acid	53	90

15	MeOH (0.5 M)	70	PhCOOH	68	90

[a]The reaction was carried out with HCHO (37% aqueous solution, 12 equiv) for the second step. $NaBAr^F_4$, Ar^F = 3,5-$(CF_3)_2Ph$. [b]Yield was determined by ^1H NMR with CH_2Br_2 as the internal standard. [c]Determined by HPLC analysis.

4. General procedure for formal hydroalkenylation

4.1 For the introduction of *di*-substituted alkenes

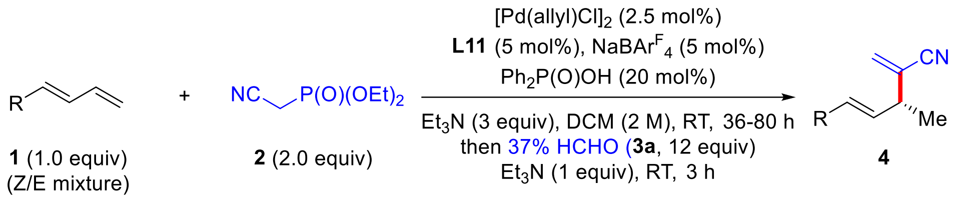

General procedure (**4a** as an example, standard condition): In a N_2-filled glovebox, $[Pd(allyl)Cl]_2$ (0.9 mg, 0.0025 mmol), **L11** (2.8 mg, 0.0050 mmol), $NaBAr^F_4$ (4.4 mg, 0.0050 mmol,), $Ph_2P(O)OH$ (4.4 mg, 0.020 mmol) and DCM (0.050 mL) were added sequentially to a 4 mL vial. The resulting yellow solution was allowed to stir at ambient temperature for 1 min. Then diene **1a** (13 mg, 0.10 mmol), diethyl (cyanomethyl)phosphonate **2** (35 mg, 0.20 mmol) and Et_3N (42 µL, 0.30 mmol) were added sequentially to the reaction. The reaction mixture continued to stir at room temperature for 48 h. After this time, aqueous formaldehyde **3a** (0.10 mL, 37% in water, 1.2 mmol) and Et_3N (14 µL, 0.10 mmol) were added to the reaction and the resulting mixture continued to stir for another 3 h at room temperature. After this time, the reaction was extracted with CH_2Cl_2 (2 mL × 3), dried over Na_2SO_4, filtered, concentrated and purified by flash silica gel chromatography (*n*-hexane/ethyl acetate = 20:1) to give the pure product **4a**.

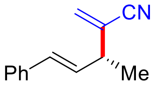

(*E*)-3-Methyl-2-methylene-5-phenylpent-4-enenitrile (**4a**)

The reaction time for the first step was 48 h. Colorless oil, 82% yield (15.0 mg), $[\alpha]_D^{25}$ +33.6 (*c* 0.49, $CHCl_3$) for 94% ee; ^1H NMR (400 MHz, chloroform-*d*) δ 7.40 – 7.35 (m, 2H), 7.35 – 7.28 (m, 2H), 7.28 – 7.21 (m, 1H), 6.49 (dd, J = 15.9, 1.2 Hz, 1H), 6.12 (dd, J = 15.9, 7.3 Hz, 1H), 5.89 (s, 1H), 5.79 (d, J = 1.4 Hz, 1H), 3.29 – 3.19 (m, 1H), 1.37 (d, J = 7.0 Hz, 3H). ^{13}C NMR (101 MHz, chloroform-*d*) δ 136.7, 131.6, 129.7, 129.2, 128.7, 127.8, 127.5, 126.4, 118.1, 41.7, 19.0. HRMS (ESI): $[M+Na]^{\oplus}$ calcd for $C_{13}H_{13}NNa^{\oplus}$ 206.0940, found 206.0939. HPLC analysis: Chiracel OJ-H column; detected at 254 nm, 20 °C; ^{i}PrOH : *n*-hexane = 0.5 : 99.5; flow = 1.5 mL/min; Retention time: 28.7 min (minor), 30.4 min (major).

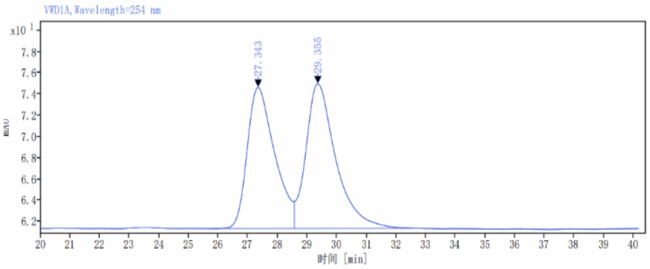

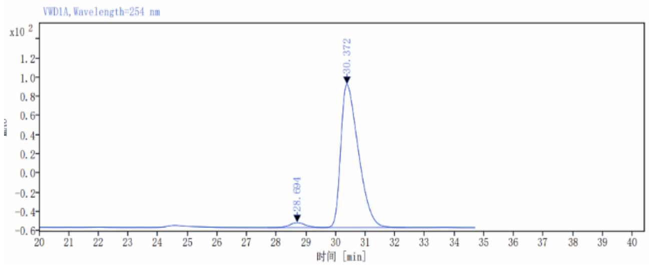

Peak	Retention time/ min	Area%
1	27.343	47.21
2	29.355	52.79

Peak	Retention time/ min	Area%
1	28.694	2.93
2	30.372	97.07

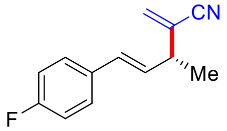

(*E*)-5-(4-Fluorophenyl)-3-methyl-2-methylenepent-4-enenitrile (4b)

The reaction time for the first step was 48 h. Colorless oil, 89% yield (17.8 mg), $[\alpha]_D^{25}$ +28.1 (*c* 0.75, $CHCl_3$) for 93% ee; ^{1}H NMR (400 MHz, chloroform-*d*) δ 7.39 – 7.29 (m, 2H), 7.06 – 6.96 (m, 2H), 6.45 (dd, *J* = 15.9, 1.2 Hz, 1H), 6.04 (dd, *J* = 15.9, 7.3 Hz, 1H), 5.89 (d, *J* = 0.7 Hz, 1H), 5.79 (d, *J* = 1.3 Hz, 1H), 3.29 – 3.17 (m, 1H), 1.37 (d, *J* = 6.9 Hz, 3H). ^{13}C NMR (101 MHz, chloroform-*d*) δ 162.4 (d, *J*= 247.0 Hz), 132.8 (d, *J*= 3.4 Hz), 130.4, 129.5 (d, *J*= 2.3 Hz), 129.3, 128.0 (d, *J*= 8.1 Hz), 127.4, 118.0, 115.6 (d, *J*= 21.6 Hz), 41.7, 19.0. ^{19}F NMR (376 MHz, chloroform-*d*) δ -114.21 – -114.28 (m, 1F). HRMS (ESI): $[M+H]^{\oplus}$ calcd for $C_{13}H_{13}NF^{\oplus}$ 202.1027, found 202.1027. HPLC analysis: Chiracel OB-H column; detected at 254 nm, 25 °C; ^{i}PrOH : *n*-hexane = 5 : 95; flow = 0.7 mL/min; Retention time: 13.4 min (minor), 15.5 min (major).

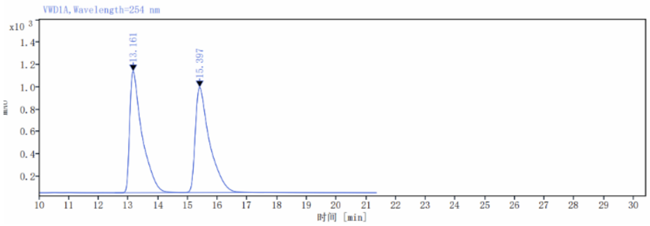

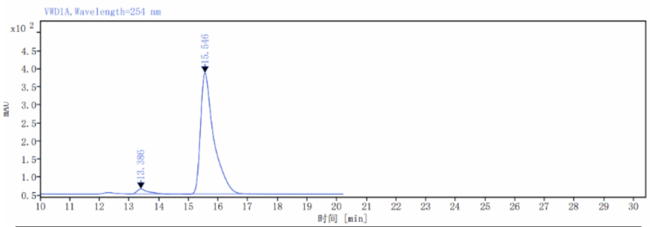

Peak	Retention time/ min	Area%
1	13.161	49.92
2	15.397	50.08

Peak	Retention time/ min	Area%
1	13.386	3.36
2	15.546	96.64

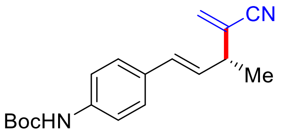

Tert-butyl (*E*)-(4-(4-cyano-3-methylpenta-1,4-dien-1-yl)phenyl)carbamate (4c)

The reaction time for the first step was 48 h. Yellow oil, 77% yield (23.0 mg), $[\alpha]_D^{25}$ +30.4 (*c* 0.86, $CHCl_3$) for 92% ee; ^{1}H NMR (400 MHz, chloroform-*d*) δ 7.33 – 7.28 (m, 4H), 6.52 (s, 1H), 6.42 (d, *J* = 15.9 Hz, 1H), 6.02 (dd, *J* = 15.9, 7.4 Hz, 1H), 5.88 (s, 1H), 5.79 (d, *J* = 1.3 Hz, 1H), 3.25 – 3.18 (m, 1H), 1.49 (s, 9H), 1.36 (d, *J* = 6.9 Hz, 3H). ^{13}C NMR (101 MHz, chloroform-*d*) δ 152.7, 138.0, 131.5, 131.0, 129.1, 128.3, 127.6, 127.1, 118.5, 118.1, 80.7, 41.7, 28.4, 19.0. HRMS (ESI): $[M+Na]^{\oplus}$ calcd for $C_{18}H_{22}O_2N_2Na^{\oplus}$ 321.1573, found 321.1572. HPLC analysis: Chiracel OJ-H column; detected at 254 nm, 25 °C; ^{i}PrOH : *n*-hexane = 12 : 88; flow = 1.0 mL/min; Retention time: 23.2 min (minor), 27.7 min (major).

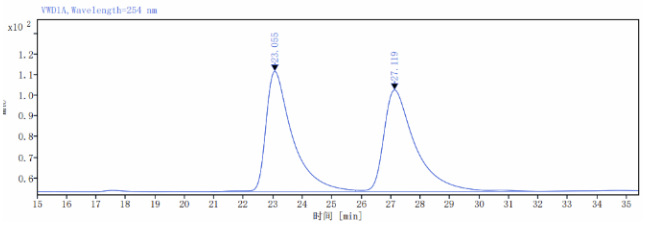

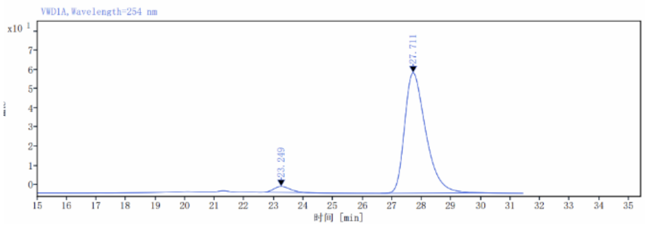

Peak	Retention time/ min	Area%
1	23. 055	49. 98
2	27. 119	50. 02

Peak	Retention time/ min	Area%
1	23. 249	3. 76
2	27. 711	96. 24

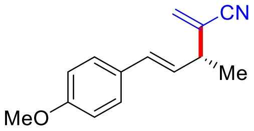

(*E*)-5-(4-Methoxyphenyl)-3-methyl-2-methylenepent-4-enenitrile (**4d**)

The reaction concentration was 1M and the reaction time was 36 h for the first step. Colorless oil, 92% yield (19.5 mg), $[\alpha]_D^{25}$ +37.8 (*c* 0.80, $CHCl_3$) for 92% ee; ^{1}H NMR (400 MHz, chloroform-*d*) δ 7.3 – 7.3 (m, 2H), 6.9 – 6.8 (m, 2H), 6.4 (dd, *J* = 15.9, 1.2 Hz, 1H), 6.0 (dd, *J* = 15.9, 7.4 Hz, 1H), 5.9 (d, *J* = 0.8 Hz, 1H), 5.8 (d, *J* = 1.3 Hz, 1H), 3.8 (s, 3H), 3.3 – 3.2 (m, 1H), 1.4 (d, *J* = 7.0 Hz, 3H). ^{13}C NMR (101 MHz, chloroform-*d*) δ 159.4, 131.0, 129.4, 129.1, 127.7, 127.6, 127.5, 118.2, 114.1, 55.4, 41.7, 19.1. HRMS (ESI): $[M+H]^{\oplus}$ calcd for $C_{14}H_{16}ON^{\oplus}$ 214.1226, found 214.1226. HPLC analysis: Chiracel OJ-H column; detected at 254 nm, 25 °C; ^{i}PrOH : *n*-hexane = 12 : 88; flow = 1.0 mL/min; Retention time: 21.0 min (major), 22.6 min (minor).

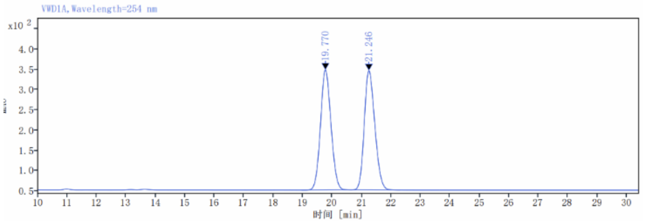

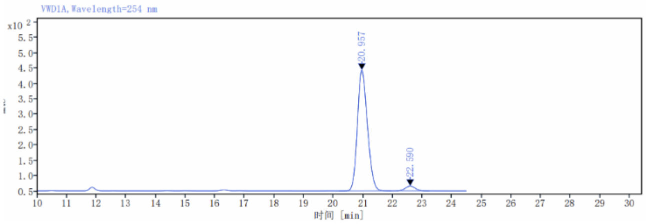

Peak	Retention time/ min	Area%
1	19. 770	50. 04
2	21. 246	49. 96

Peak	Retention time/ min	Area%
1	20. 957	96. 27
2	22. 590	3. 73

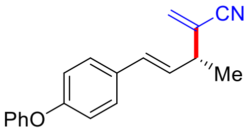

(*E*)-3-Methyl-2-methylene-5-(4-phenoxyphenyl)pent-4-enenitrile (**4e**)

The reaction time for the first step was 48 h. Colorless oil, 93% yield (25.5 mg), $[\alpha]_D^{25}$ +30.9 (*c* 0.90, $CHCl_3$) for 92% ee; ^{1}H NMR (400 MHz, chloroform-*d*) δ 7.36-7.32 (m, 4H), 7.15 – 7.08 (m, 1H), 7.04 – 6.92 (m, 4H), 6.47 (dd, *J* = 15.9, 1.2 Hz, 1H), 6.04 (dd, *J* = 15.9, 7.4 Hz, 1H), 5.90 (d, *J* = 0.8 Hz, 1H), 5.80 (d, *J* = 1.3 Hz, 1H), 3.28 – 3.19 (m, 1H), 1.38 (d, *J* = 6.9 Hz, 3H). ^{13}C NMR (101 MHz, chloroform-*d*) δ 157.1, 157.0, 131.9, 130.8, 129.8, 129.2, 128.8, 127.8, 127.6, 123.4, 119.01, 118.96, 118.1, 41.7, 19.0. HRMS (ESI): $[M+Na]^{\oplus}$ calcd for $C_{19}H_{17}ONNa^{\oplus}$ 298.1202, found 298.1200. HPLC analysis: Chiracel IG-

3 column; detected at 254 nm, 25 °C; ^{i}PrOH : *n*-hexane = 5 : 95; flow = 0.7 mL/min; Retention time: 14.4 min (minor), 15.0 min (major).

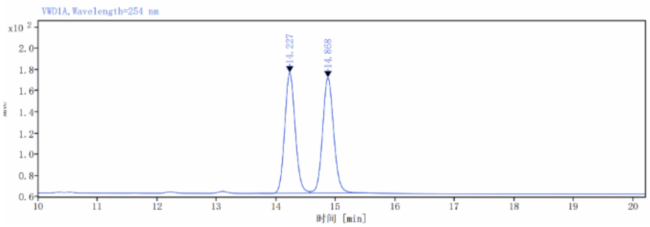

Peak	Retention time/ min	Area%
1	14. 227	49. 51
2	14. 868	50. 49

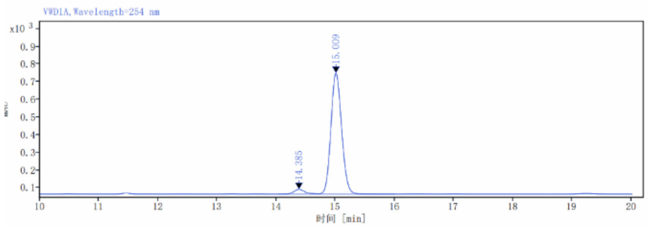

Peak	Retention time/ min	Area%
1	14. 385	3. 84
2	15. 009	96. 16

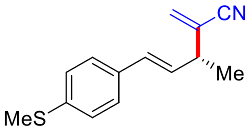

(*E*)-3-Methyl-2-methylene-5-(4-(methylthio)phenyl)pent-4-enenitrile (**4f**)

The reaction time for the first step was 48 h. Colorless oil, 83% yield (19.0 mg), $[\alpha]_D^{25}$ +42.2 (*c* 0.72, $CHCl_3$) for 93% ee; ^{1}H NMR (400 MHz, chloroform-*d*) δ 7.29 (d, *J* = 8.3 Hz, 2H), 7.20 (d, *J* = 8.4 Hz, 2H), 6.44 (d, *J* = 15.9 Hz, 1H), 6.08 (dd, *J* = 15.9, 7.4 Hz, 1H), 5.89 (s, 1H), 5.79 (d, *J* = 1.3 Hz, 1H), 3.26-3.19 (m, 1H), 2.48 (s, 3H), 1.37 (d, *J* = 6.9 Hz, 3H). ^{13}C NMR (101 MHz, chloroform-*d*) δ 138.1, 133.6, 130.9, 129.2, 129.1, 127.5, 126.8, 126.7, 118.1, 41.7, 19.0, 15.9. HRMS (ESI): $[M+Na]^{\oplus}$ calcd for $C_{14}H_{15}NNaS^{\oplus}$ 252.0817, found 252.0814. HPLC analysis: Chiracel OJ-H column; detected at 254 nm, 25 °C; ^{i}PrOH : *n*-hexane = 12 : 88; flow = 1.0 mL/min; Retention time: 21.1 min (major), 24.3 min (minor).

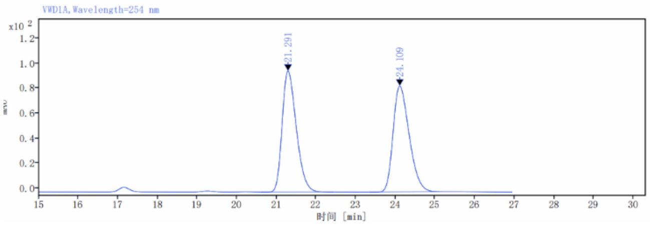

Peak	Retention time/ min	Area%
1	21. 291	50. 14
2	24. 109	49. 86

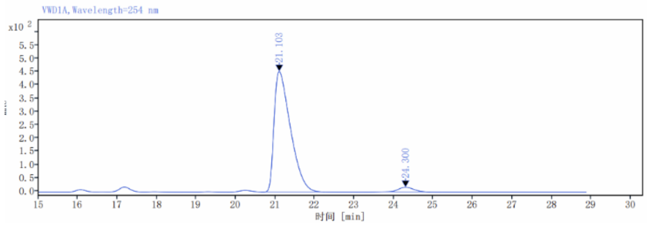

Peak	Retention time/ min	Area%
1	21. 103	96. 55
2	24. 300	3. 45

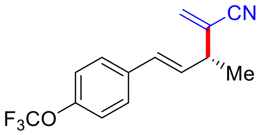

(*E*)-3-Methyl-2-methylene-5-(4-(trifluoromethoxy)phenyl)pent-4-enenitrile (**4g**)

The reaction time for the first step was 80 h. Colorless oil, 73% yield (19.6 mg), $[\alpha]_D^{25}$ +20.5 (*c* 0.97, $CHCl_3$) for 94% ee; ^{1}H NMR (400 MHz, chloroform-*d*) δ 7.41 – 7.35 (m, 2H), 7.19 – 7.13 (m, 2H), 6.48 (dd, *J* = 16.0, 1.2 Hz, 1H), 6.11 (dd, *J* = 15.9, 7.3 Hz, 1H), 5.90 (s, 1H), 5.80 (d, *J* = 1.2 Hz, 1H), 3.31 – 3.19 (m, 1H), 1.38 (d, *J* = 6.9 Hz, 3H). ^{13}C NMR (101 MHz, chloroform-*d*) δ 148.7 (d, *J* = 1.9 Hz), 135.4, 130.8, 130.2, 129.4, 127.7, 127.2, 121.2, 120.5 (q, J=257.2 Hz), 118.0, 41.7, 18.9. ^{19}F NMR (376 MHz, chloroform-*d*) δ -57.91 (s, 3F). HRMS (ESI): $[M+Na]^{\oplus}$ calcd for $C_{14}H_{12}ONF_3Na^{\oplus}$ 290.0763, found

290.0762. HPLC analysis: Chiracel OJ-H column; detected at 254 nm, 25 °C; ^{i}PrOH : *n*-hexane = 12 : 88; flow = 1.0 mL/min; Retention time: 6.2 min (minor), 6.5 min (major).

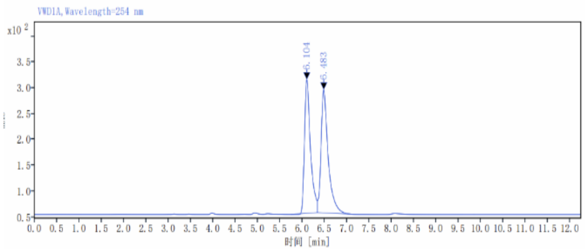

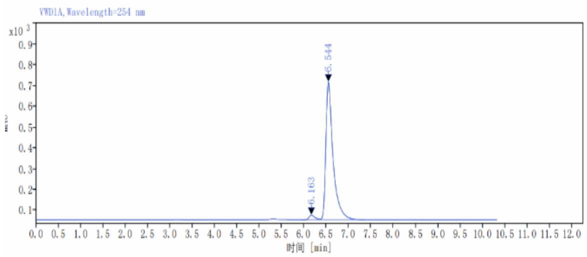

Peak	Retention time/ min	Area%
1	6. 104	47. 56
2	6. 483	52. 44

Peak	Retention time/ min	Area%
1	6. 163	2. 69
2	6. 544	97. 31

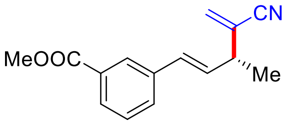

Methyl (*E*)-3-(4-cyano-3-methylpenta-1,4-dien-1-yl)benzoate (4h)

The reaction time for the first step was 72 h. Colorless oil, 65% yield (15.7 mg), $[\alpha]_D^{25}$ +22.6 (*c* 0.77, $CHCl_3$) for 95% ee; ^{1}H NMR (400 MHz, chloroform-*d*) δ 8.05 (s, 1H), 7.95 – 7.87 (m, 1H), 7.58 – 7.52 (m, 1H), 7.40 (t, *J* = 7.7 Hz, 1H), 6.53 (d, *J* = 15.9 Hz, 1H), 6.21 (dd, *J* = 15.9, 7.3 Hz, 1H), 5.92 (s, 1H), 5.81 (d, *J* = 1.3 Hz, 1H), 3.93 (s, 3H), 3.27 (p, *J* = 7.0 Hz, 1H), 1.39 (d, *J* = 6.9 Hz, 3H). ^{13}C NMR (101 MHz, chloroform-*d*) δ 167.0, 137.0, 131.0, 130.8, 130.64, 130.57, 129.4, 128.80, 128.75, 127.4, 127.2, 118.0, 52.3, 41.7, 18.9. HRMS (ESI): $[M+H]^{\oplus}$ calcd for $C_{15}H_{16}O_2N^{\oplus}$ 242.1176, found 242.1173. HPLC analysis: Chiracel OD-H column; detected at 254 nm, 25 °C; ^{i}PrOH : *n*-hexane = 20 : 80; flow = 1.0 mL/min; Retention time: 9.5 min (minor), 10.5 min (major).

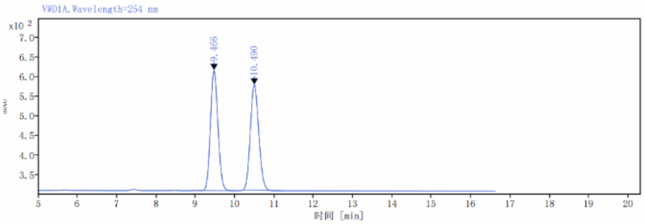

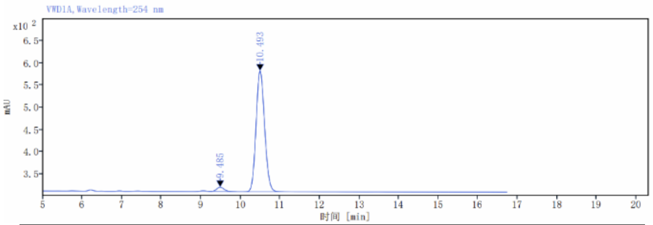

Peak	Retention time/ min	Area%
1	9. 466	50. 27
2	10. 490	49. 73

Peak	Retention time/ min	Area%
1	9. 485	2. 76
2	10. 493	97. 24

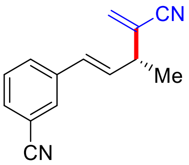

(*E*)-3-(4-Cyano-3-methylpenta-1,4-dien-1-yl)benzonitrile (4i)

50 °C was adopted and the reaction time was 72 h for the first step. Colorless oil, 71% yield (14.8 mg), $[\alpha]_D^{25}$ +25.3 (*c* 0.14, $CHCl_3$) for 91% ee; ^{1}H NMR (400 MHz, chloroform-*d*) δ 7.64 (s, 1H), 7.62 – 7.56 (m, 1H), 7.53 (dt, *J* = 7.7, 1.4 Hz, 1H), 7.43 (t, *J* = 7.7 Hz, 1H), 6.48 (d, *J* = 16.0 Hz, 1H), 6.20 (dd, *J* = 15.9, 7.3 Hz, 1H), 5.93 (s, 1H), 5.82 (d, *J* = 1.3 Hz, 1H), 3.31-3.24 (m, 1H), 1.39 (d, *J* = 6.9 Hz, 3H). ^{13}C

NMR (101 MHz, chloroform-*d*) δ 137.9, 132.6, 131.1, 130.6, 130.0, 129.7, 129.5, 129.5, 126.9, 118.7, 117.8, 112.9, 41.7, 18.8. HRMS (ESI): [M+H]$^{\oplus}$ calcd for $C_{14}H_{13}N_2^{\oplus}$ 209.1073, found 209.1070. HPLC analysis: Chiracel OJ-H column; detected at 254 nm, 25 °C; ^{i}PrOH : *n*-hexane = 12 : 88; flow = 1.0 mL/min; Retention time: 24.2 min (minor), 26.9 min (major).

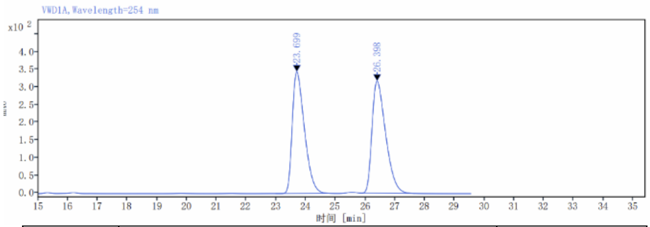

Peak	Retention time/ min	Area%
1	23. 699	49. 18
2	26. 398	50. 82

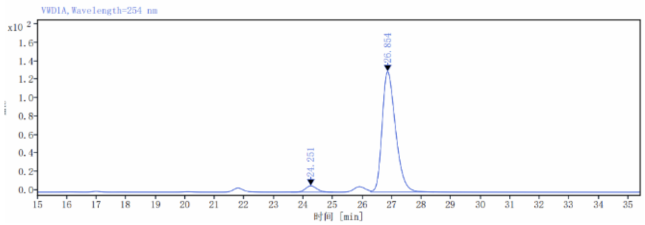

Peak	Retention time/ min	Area%
1	24. 251	4. 22
2	26. 854	95. 78

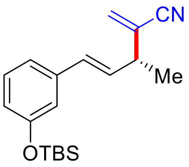

(*E*)-5-(3-((Tert-butyldimethylsilyl)oxy)phenyl)-3-methyl-2-methylenepent-4-enenitrile (**4j**)

The reaction time for the first step was 72 h. Colorless oil, 68% yield (21.2 mg), $[\alpha]_D^{25}$ +19.7 (*c* 0.95, $CHCl_3$) for 94% ee; ^{1}H NMR (400 MHz, chloroform-*d*) δ 7.17 (t, *J* = 7.9 Hz, 1H), 6.98 (dt, *J* = 7.8, 1.5 Hz, 1H), 6.84 (t, *J* = 2.0 Hz, 1H), 6.73 (ddd, *J* = 8.0, 2.5, 1.0 Hz, 1H), 6.48 – 6.38 (m, 1H), 6.08 (dd, *J* = 15.9, 7.3 Hz, 1H), 5.89 (d, *J* = 0.8 Hz, 1H), 5.79 (d, *J* = 1.2 Hz, 1H), 3.29 – 3.18 (m, 1H), 1.37 (d, *J* = 7.0 Hz, 3H), 0.99 (s, 9H), 0.21 (s, 6H). ^{13}C NMR (101 MHz, chloroform-*d*) δ 156.0, 138.1, 131.5, 129.7, 129.6, 129.3, 127.5, 119.6, 119.5, 118.11, 118.08, 41.7, 25.8, 19.0, 18.3, -4.3. HRMS (ESI): [M+H]$^{\oplus}$ calcd for $C_{19}H_{28}ONSi^{\oplus}$ 314.1935, found 314.1931. HPLC analysis: Chiracel OJ-H column; detected at 254 nm, 25 °C; ^{i}PrOH : *n*-hexane = 2 : 98; flow = 0.7 mL/min; Retention time: 10.6 min (minor), 11.7 min (major).

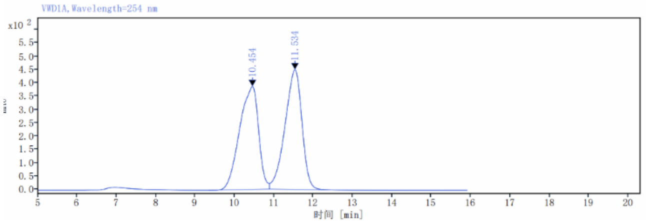

Peak	Retention time/ min	Area%
1	10. 454	49. 08
2	11. 534	50. 92

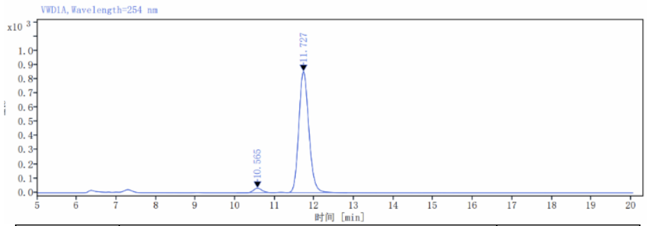

Peak	Retention time/ min	Area%
1	10. 565	2. 93
2	11. 727	97. 07

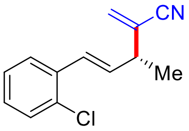

(*E*)-5-(2-Chlorophenyl)-3-methyl-2-methylenepent-4-enenitrile (**4k**)

The reaction time for the first step was 72 h. Yellow oil, 78% yield (17.0 mg), $[\alpha]_D^{25}$ +14.3 (*c* 0.60, $CHCl_3$) for 90% ee; ^{1}H NMR (400 MHz, chloroform-*d*) δ 7.52 (dd, *J* = 7.5, 2.0 Hz, 1H), 7.35 (dd, *J* = 7.6, 1.8 Hz,

1H), 7.25 – 7.15 (m, 2H), 6.89 (d, J = 15.8 Hz, 1H), 6.12 (dd, J = 15.8, 7.4 Hz, 1H), 5.91 (s, 1H), 5.82 (d, J = 1.3 Hz, 1H), 3.30 (p, J = 7.1 Hz, 1H), 1.40 (d, J = 7.0 Hz, 3H). ^{13}C NMR (101 MHz, chloroform-d) δ 134.8, 133.1, 132.5, 129.8, 129.5, 128.9, 128.0, 127.3, 126.98, 126.96, 117.9, 41.9, 19.0. HRMS (ESI): $[M+Na]^{\oplus}$ calcd for $C_{13}H_{12}NClNa^{\oplus}$ 240.0550, found 240.0549. HPLC analysis: Chiracel OJ-H column; detected at 254 nm, 20 °C; ^{i}PrOH : n-hexane = 1 : 99; flow = 0.7 mL/min; Retention time: 18.6 min (minor), 20.4 min (major).

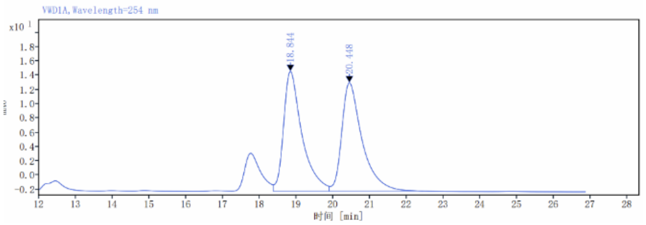

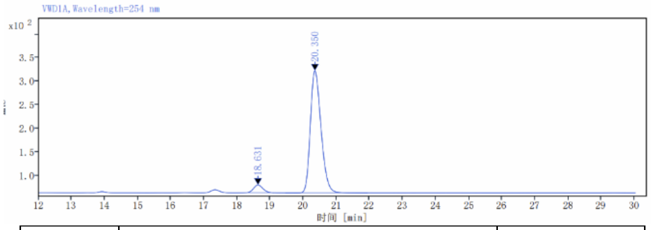

Peak	Retention time/ min	Area%
1	18. 844	49. 94
2	20. 448	50. 06

Peak	Retention time/ min	Area%
1	18. 631	5. 18
2	20. 350	94. 82

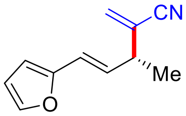

(*E*)-5-(Furan-2-yl)-3-methyl-2-methylenepent-4-enenitrile (**4l**)

The reaction time for the first step was 48 h. Colorless oil, 88% yield (15.3 mg), $[\alpha]_D^{25}$ +46.1 (c 0.61, $CHCl_3$) for 93% ee; ^{1}H NMR (400 MHz, chloroform-d) δ 7.34 (d, J = 1.8 Hz, 1H), 6.37-6.36 (m, 1H), 6.30 (dd, J = 15.9, 1.2 Hz, 1H), 6.23 (d, J = 3.3 Hz, 1H), 6.08 (dd, J = 15.9, 7.2 Hz, 1H), 5.89 (s, 1H), 5.79 (d, J = 1.3 Hz, 1H), 3.20 (p, J = 7.1 Hz, 1H), 1.35 (d, J = 6.9 Hz, 3H). ^{13}C NMR (101 MHz, chloroform-d) δ 152.2, 142.1, 129.4, 128.3, 127.3, 119.9, 118.0, 111.4, 108.2, 41.3, 18.8. HRMS (ESI): $[M+H]^{\oplus}$ calcd for $C_{11}H_{12}ON^{\oplus}$ 174.0913, found 174.0912. HPLC analysis: Chiracel OJ-H column; detected at 254 nm, 25 °C; ^{i}PrOH : n-hexane = 12 : 88; flow = 1.0 mL/min; Retention time: 9.8 min (minor), 11.9 min (major).

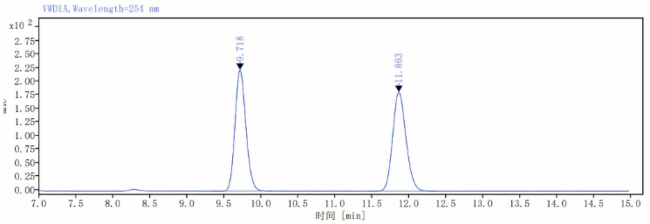

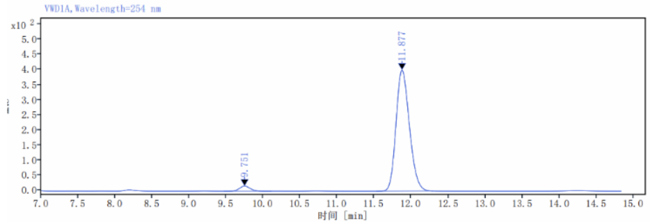

Peak	Retention time/ min	Area%
1	9. 718	49. 98
2	11. 863	50. 02

Peak	Retention time/ min	Area%
1	9. 751	3. 24
2	11. 877	96. 76

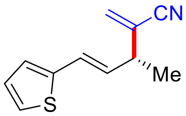

(*E*)-3-Methyl-2-methylene-5-(thiophen-2-yl)pent-4-enenitrile (**4m**)

The reaction time for the first step was 48 h. Yellow oil, 86% yield (16.3 mg), $[\alpha]_D^{25}$ +44.8 (c 0.65, $CHCl_3$) for 90% ee; ^{1}H NMR (400 MHz, chloroform-d) δ 7.19 – 7.13 (m, 1H), 6.98 – 6.93 (m, 2H), 6.61 (dd, J =

15.8, 1.3 Hz, 1H), 5.96 (dd, J = 15.7, 7.2 Hz, 1H), 5.90 (d, J = 0.8 Hz, 1H), 5.79 (d, J = 1.3 Hz, 1H), 3.24-3.17 (m, 1H), 1.36 (d, J = 6.9 Hz, 3H). ^{13}C NMR (101 MHz, chloroform-*d*) δ 141.7, 129.4, 129.2, 127.5, 127.3, 126.0, 124.8, 124.4, 118.0, 41.5, 18.8. HRMS (ESI): [M+H]$^{\oplus}$ calcd for $C_{11}H_{12}NS^{\oplus}$ 190.0685, found 190.0683. HPLC analysis: Chiracel OJ-H column; detected at 254 nm, 25 °C; ^{i}PrOH : *n*-hexane = 12 : 88; flow = 1.0 mL/min; Retention time: 9.5 min (minor), 12.3 min (major).

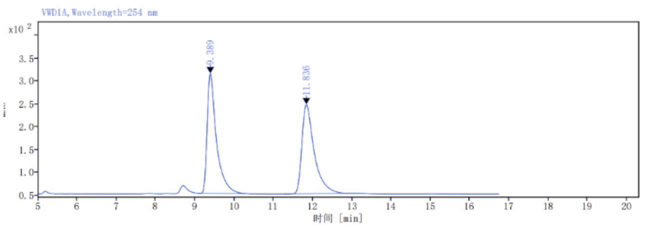

Peak	Retention time/ min	Area%
1	9. 389	50. 12
2	11. 836	49. 88

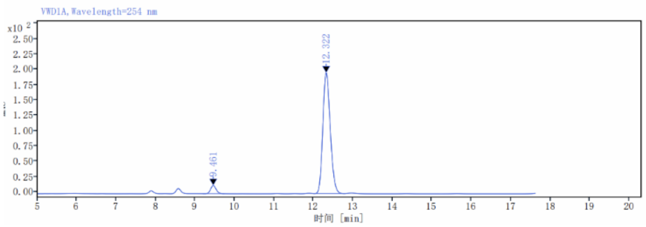

Peak	Retention time/ min	Area%
1	9. 461	4. 77
2	12. 322	95. 23

4.2 For the introduction of *tri*-substituted and *tetra*-substituted alkenes

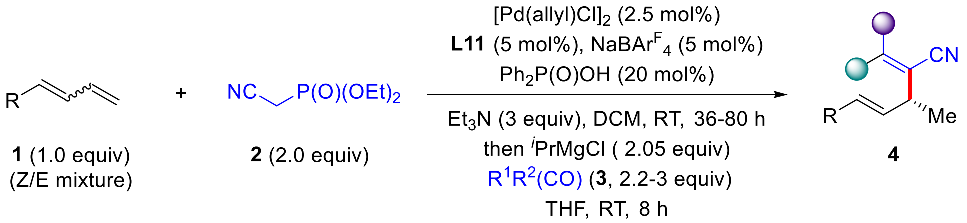

General procedure (**4n** as an example, standard condition): In a N_2-filled glovebox, $[Pd(allyl)Cl]_2$ (0.9 mg, 0.0025 mmol), **L11** (2.8 mg, 0.0050 mmol), $NaBAr^F{}_4$ (4.4 mg, 0.0050 mmol), $Ph_2P(O)OH$ (4.4 mg, 0.020 mmol) and DCM (0.050 mL) were added sequentially to a 4 mL vial. The resulting yellow solution was allowed to stir at room temperature for 1 min. Then diene **1a** (13 mg, 0.10 mmol), diethyl (cyanomethyl)phosphonate **2** (35 mg, 0.20 mmol) and Et_3N (42 µL, 0.30 mmol) were added sequentially to the reaction. The resulting mixture continued to stir at room temperature for 48 h. Then the reaction solvent was removed under nitrogen. THF (0.20 mL) and *i*-PrMgCl (1.0 M in THF, 0.21 mL) were added to the reaction sequentially. The resulting solution continued to stir at room temperature for 30 min. Next acetaldehyde **3n** (5.0 M in THF, 60 µL, 0.30 mmol) was added and the reaction continued to stir for additional 8 h at room temperature. After this time, the reaction was quenched with saturated NH_4Cl aqueous solution (3.0 mL), extracted with CH_2Cl_2 (5.0 mL × 3), dried over Na_2SO_4, filtered, concentrated and purified by flash silica gel chromatography (*n*-hexane/ethyl acetate = 20:1) to give the pure product **4n**.

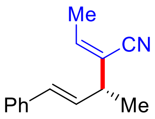

(*R*,2*Z*,4*E*)-2-Ethylidene-3-methyl-5-phenylpent-4-enenitrile (4n)

Colorless oil, 87% yield (17.2 mg), $[\alpha]_D^{25}$ +40.4 (*c* 0.44, $CHCl_3$) for 94% ee; ^{1}H NMR (400 MHz, chloroform-*d*) δ 7.40 – 7.34 (m, 2H), 7.31 (t, J = 7.6 Hz, 2H), 7.27 – 7.22 (m, 1H), 6.45 (d, J = 15.9 Hz, 1H), 6.35 – 6.25 (m, 1H), 6.14 (dd, J = 15.9, 7.3 Hz, 1H), 3.19 (p, J = 7.2 Hz, 1H), 2.00 (d, J = 6.9 Hz,

3H), 1.35 (d, J = 6.9 Hz, 3H). ^{13}C NMR (101 MHz, chloroform-d) δ 141.6, 136.8, 130.8, 130.7, 128.6, 127.7, 126.4, 120.3, 116.9, 41.4, 19.4, 17.2. HRMS (EI): [M]$^{\oplus}$ calcd for $C_{14}H_{15}N^{\oplus}$ 197.1199, found 197.1202. HPLC analysis: Chiracel (OD-H) + (OD-H) column; detected at 254 nm, 20 °C; ^{i}PrOH : n-hexane = 3 : 97; flow = 0.7 mL/min; Retention time: 18.3 min (minor), 18.9 min (major).

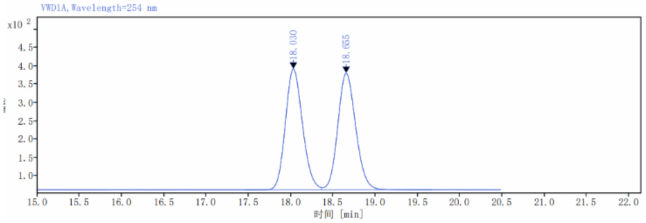

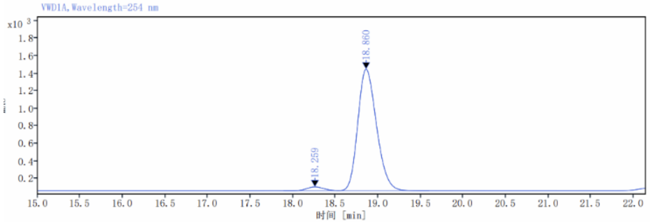

Peak	Retention time/ min	Area%
1	18. 030	49. 72
2	18. 655	50. 28

Peak	Retention time/ min	Area%
1	18. 259	2. 84
2	18. 860	97. 16

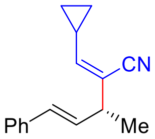

(*R*,2*Z*,4*E*)-2-(Cyclopropylmethylene)-3-methyl-5-phenylpent-4-enenitrile (**4o**)

The reaction time for the first step was 48 h. Aldehyde **3** (3 equiv) was used. Colorless oil, 79% yield (17.7 mg), $[\alpha]_D^{25}$ +83.2 (c 0.80, $CHCl_3$) for 94% ee; ^{1}H NMR (400 MHz, chloroform-d) δ 7.38 – 7.36 (m, 1H), 7.31 (t, J = 7.5 Hz, 2H), 7.25 – 7.21 (m, 1H), 6.44 (d, J = 15.9 Hz, 1H), 6.13 (dd, J = 15.9, 7.3 Hz, 1H), 5.54 (d, J = 10.4 Hz, 1H), 3.15 (p, J = 7.0 Hz, 1H), 1.98 – 1.89 (m, 1H), 1.34 (d, J = 6.9 Hz, 3H), 1.04 – 0.96 (m, 2H), 0.62 – 0.54 (m, 2H). ^{13}C NMR (101 MHz, chloroform-d) δ 151.3, 136.9, 130.9, 130.7, 128.6, 127.6, 126.4, 117.9, 115.6, 41.1, 19.4, 14.3, 8.49, 8.47. HRMS (ESI): [M+Na]$^{\oplus}$ calcd for $C_{16}H_{17}NNa^{\oplus}$ 246.1253, found 246.1253. HPLC analysis: Chiracel OD-H column; detected at 254 nm, 25 °C; ^{i}PrOH : n-hexane = 10 : 90; flow = 0.7 mL/min; Retention time: 7.3 min (minor), 7.8 min (major).

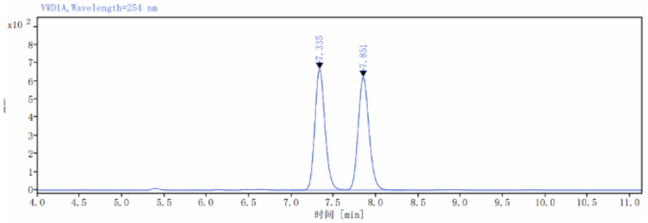

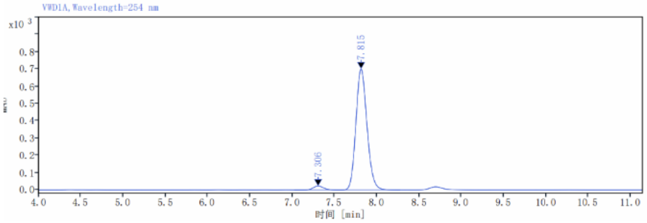

Peak	Retention time/ min	Area%
1	7. 335	49. 96
2	7. 851	50. 04

Peak	Retention time/ min	Area%
1	7. 306	2. 87
2	7. 815	97. 13

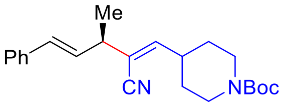

***tert*-Butyl 4-((*R*,1*Z*,4*E*)-2-cyano-3-methyl-5-phenylpenta-1,4-dien-1-yl)piperidine-1-carboxylate** (**4p**)

The reaction time for the first step was 48 h. Aldehyde **3** (2.2 equiv) was used. Yellow oil, 77% yield (28.0 mg), $[\alpha]_D^{25}$ +42.0 (c 1.1, $CHCl_3$) for 94% ee; ^{1}H NMR (400 MHz, chloroform-d) δ 7.40 – 7.35 (m, 2H), 7.32 (t, J = 7.6 Hz, 2H), 7.28 – 7.21 (m, 1H), 6.45 (d, J = 16.1 Hz, 1H), 6.12 (dd, J = 15.9, 7.3 Hz, 1H),

6.02 (dd, J = 9.7, 1.1 Hz, 1H), 4.24 - 4.01 (m, 2H), 3.17 (p, J = 7.0 Hz, 1H), 2.87 – 2.64 (m, 3H), 1.70-1.66 (m, 2H), 1.46 (s, 9H), 1.39 – 1.30 (m, 5H). ^{13}C NMR (101 MHz, chloroform-d) δ 154.7, 149.5, 136.7, 131.1, 130.3, 128.7, 127.8, 126.4, 118.6, 116.8, 93.8, 79.7, 41.3, 39.0, 31.1, 28.5, 19.4. HRMS (ESI): [M+Na]$^{\oplus}$ calcd for $C_{23}H_{30}O_2N_2Na^{\oplus}$ 389.2199, found 389.2201. HPLC analysis: Chiracel OD-H column; detected at 254 nm, 25 ^{o}C; ^{i}PrOH : n-hexane = 20 : 80; flow = 1.0 mL/min; Retention time: 5.5 min (minor), 6.1 min (major).

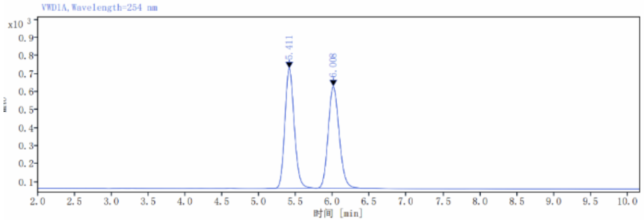

Peak	Retention time/ min	Area%
1	5. 411	50. 17
2	6. 008	49. 83

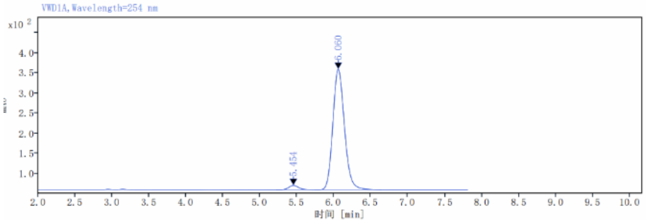

Peak	Retention time/ min	Area%
1	5. 454	2. 87
2	6. 060	97. 13

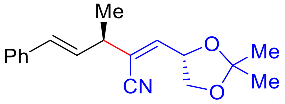

(*R*,2*Z*,4*E*)-2-(((*S*)-2,2-Dimethyl-1,3-dioxolan-4-yl)methylene)-3-methyl-5-phenylpent-4-enenitrile (4q)

The reaction time for the first step was 48 h. Aldehyde **3** (3 equiv) was used. Colorless oil, 62% yield (17.5 mg), >20:1 dr, $[\alpha]_D^{25}$ +42.3 (c 0.78, $CHCl_3$); ^{1}H NMR (400 MHz, chloroform-d) δ 7.40 – 7.28 (m, 4H), 7.27 – 7.24 (m, 1H), 6.48 (d, J = 15.9 Hz, 1H), 6.22 (dd, J = 8.5, 1.2 Hz, 1H), 6.11 (dd, J = 15.9, 7.5 Hz, 1H), 5.01 – 4.96 (m, 1H), 4.24 (dd, J = 8.4, 6.4 Hz, 1H), 3.65 (dd, J = 8.4, 6.8 Hz, 1H), 3.24 (p, J = 7.0 Hz, 1H), 1.45 (s, 3H), 1.42 – 1.35 (m, 6H). ^{13}C NMR (101 MHz, chloroform-d) δ 143.0, 136.5, 131.7, 129.5, 128.7, 127.9, 126.5, 122.2, 115.9, 110.5, 74.4, 68.8, 41.6, 26.7, 25.6, 19.0. HRMS (ESI): [M+Na]$^{\oplus}$ calcd for $C_{18}H_{21}O_2NNa^{\oplus}$ 306.1465, found 306.1464.

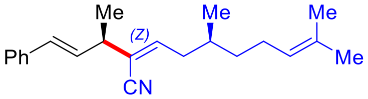

(*S*,*Z*)-5,9-Dimethyl-2-((*R*,*E*)-4-phenylbut-3-en-2-yl)deca-2,8-dienenitrile (4r)

The reaction time for the first step was 48 h. Aldehyde **3** (2.2 equiv) and i-PrMgCl (2.2 equiv) were used. Colorless oil, 63% yield (19.3 mg), >20:1 dr, $[\alpha]_D^{25}$ +43.0 (c 0.85, $CHCl_3$); ^{1}H NMR (400 MHz, chloroform-d) δ 7.40 – 7.34 (m, 2H), 7.31 (t, J = 7.6 Hz, 2H), 7.27 – 7.20 (m, 1H), 6.45 (d, J = 15.6 Hz, 1H), 6.24 (t, J = 7.7 Hz, 1H), 6.15 (dd, J = 15.9, 7.2 Hz, 1H), 5.10 – 5.06 (m, 1H), 3.21 (p, J = 7.0 Hz, 1H), 2.43 – 2.36 (m, 1H), 2.29 – 2.22 (m, 1H), 2.07 – 1.92 (m, 2H), 1.71 – 1.60 (m, 8H), 1.36 (d, J = 6.9 Hz, 3H), 1.27 – 1.21 (m, 1H), 0.93 (d, J = 6.8 Hz, 3H). ^{13}C NMR (101 MHz, chloroform-d) δ 145.7, 136.9, 131.7, 130.8, 128.6, 127.7, 126.4, 124.3, 120.0, 117.1, 41.5, 38.7, 36.7, 32.6, 25.8, 25.5, 19.5, 19.4, 17.7 (one aryl carbon signal was not observed because of overlapping). HRMS (ESI): [M+Na]$^{\oplus}$ calcd for $C_{22}H_{29}NNa^{\oplus}$ 330.2192, found 330.2191.

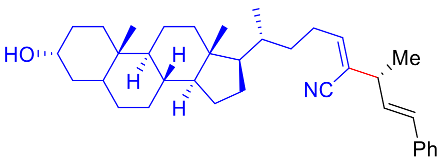

(6*R*,*Z*)-6-((3*R*,8*R*,9*S*,10*S*,13*R*,14*S*,17*R*)-3-Hydroxy-10,13-dimethylhexadecahydro-1*H*-cyclopenta[a]phenanthren-17-yl)-2-((*E*)-4-phenylbut-3-en-2-yl)hept-2-enenitrile (**4s**)

The reaction time for the first step was 48 h. Aldehyde **3** (2.2 equiv) was used. Colorless oil, 75% yield (38.6 mg), >20:1 dr, $[\alpha]_D^{25}$ +33.5 (*c* 1.1, $CHCl_3$); ^1H NMR (400 MHz, chloroform-*d*) δ 7.38 – 7.36 (m, 2H), 7.31 (t, *J* = 7.6 Hz, 2H), 7.27 – 7.20 (m, 1H), 6.45 (d, *J* = 15.9 Hz, 1H), 6.21 (t, *J* = 7.6 Hz, 1H), 6.14 (dd, *J* = 15.9, 7.3 Hz, 1H), 3.66 – 3.58 (m, 1H), 3.18 (p, *J* = 7.0 Hz, 1H), 2.48 – 2.36 (m, 1H), 2.23 – 2.23 (m, 1H), 2.02 – 1.91 (m, 1H), 1.89 – 1.73 (m, 4H), 1.69 – 1.63 (m, 1H), 1.60 – 1.33 (m, 14H), 1.29 – 1.16 (m, 5H), 1.15 – 1.09 (m, 2H), 1.06 – 1.04 (m, 3H), 0.96 (d, *J* = 6.5 Hz, 3H), 0.92 (s, 3H), 0.64 (s, 3H). ^{13}C NMR (101 MHz, chloroform-*d*) δ 147.5, 136.9, 130.80, 130.76, 128.6, 127.6, 126.4, 118.8, 117.1, 71.9, 56.5, 56.0, 42.8, 42.1, 41.3, 40.5, 40.2, 36.5, 35.9, 35.6, 35.4, 34.9, 34.6, 30.6, 28.5, 28.4, 27.3, 26.5, 24.3, 23.4, 20.9, 19.4, 18.6, 12.1. HRMS (ESI): $[M+Na]^{\oplus}$ calcd for $C_{36}H_{51}ONNa^{\oplus}$ 536.3863, found 536.3865.

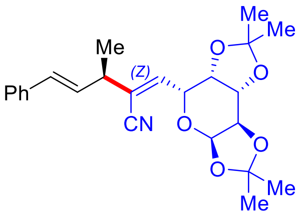

(*R*,2*Z*,4*E*)-3-Methyl-5-phenyl-2-(((3*aR*,5*R*,5*aS*,8*aS*,8*bR*)-2,2,7,7-tetramethyltetrahydro-5*H*-bis([1,3]dioxolo)[4,5-b:4',5'-d]pyran-5-yl)methylene)pent-4-enenitrile (**4t**)

The reaction time for the first step was 48 h. Aldehyde **3** (2.2 equiv) was used. White solid, 74% yield (30.6 mg), 16:1 dr, $[\alpha]_D^{25}$ -77.3 (*c* 1.0, $CHCl_3$); ^1H NMR (400 MHz, chloroform-*d*) δ 7.38 – 7.34 (m, 2H), 7.33 – 7.29 (m, 2H), 7.27 – 7.20 (m, 1H), 6.49 (d, *J* = 15.6 Hz, 1H), 6.36 (dd, *J* = 8.5, 1.1 Hz, 1H), 6.14 (dd, *J* = 15.9, 7.3 Hz, 1H), 5.54 (d, *J* = 5.0 Hz, 1H), 4.78 (dd, *J* = 8.5, 2.0 Hz, 1H), 4.66 (dd, *J* = 7.8, 2.5 Hz, 1H), 4.35 (dd, *J* = 5.0, 2.5 Hz, 1H), 4.27 (dd, *J* = 7.8, 2.0 Hz, 1H), 3.26 (p, *J* = 7.0 Hz, 1H), 1.64 (s, 3H), 1.47 (s, 3H), 1.38 (d, *J* = 6.9 Hz, 3H), 1.34 (s, 6H). 13C NMR (101 MHz, Chloroform-*d*) δ 141.4, 136.7, 131.5, 129.7, 128.6, 127.7, 126.5, 121.6, 116.1, 109.7, 109.3, 96.3, 72.9, 70.8, 70.1, 67.5, 41.5, 26.2, 26.0, 25.0, 24.3, 19.0. HRMS (ESI): $[M+H]^{\oplus}$ calcd for $C_{24}H_{30}O_5N^{\oplus}$ 412.2118, found 412.2114.

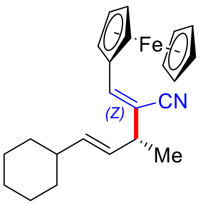

(*E*)-2-((*Z*)-Ferrocenylidene)-3-methyl-5-cyclohexylpent-4-enenitrile (**4u**)

$[Pd(allyl)Cl]_2$ (5 mol%), **L11** (10 mol%) and $NaBAr^F_4$ (10 mol%) were used. The reaction time for the first step was 48 h. Aldehyde **3** (2.2 equiv) was used. Red oil, 40% yield (14.8 mg), $[\alpha]_D^{25}$ +219.8 (c 0.010, $CHCl_3$) for 92% ee; ^1H NMR (400 MHz, chloroform-*d*) δ 6.71 (s, 1H), 5.52 (dd, *J* = 15.5, 6.5 Hz, 1H), 5.39 (dd, *J* = 15.5, 6.6 Hz, 1H), 4.81 (d, *J* = 28.1 Hz, 2H), 4.40 (s, 2H), 4.18 (s, 5H), 2.99 (t, *J* = 6.8 Hz,

1H), 2.01 – 1.94 (m, 1H), 1.69 (dd, *J* = 32.8, 12.2 Hz, 5H), 1.30 – 1.07 (m, 8H). ^{13}C NMR (101 MHz, chloroform-*d*) δ 142.2, 137.7, 128.8, 119.3, 111.9, 70.8, 70.7, 69.74, 69.72, 69.4, 42.2, 40.6, 33.1, 33.0, 26.2, 26.1, 19.7 (one alkyl carbon signal was not observed because of overlapping). HRMS (ESI): [M]$^{\oplus}$ calcd for $C_{23}H_{27}FeNNa^{\oplus}$ 373.1487, found 373.1478. HPLC analysis: Chiracel (AD-H) + (AD-H) column; detected at 254 nm, 20 °C; ^{i}PrOH : *n*-hexane = 5 : 95; flow = 0.7 mL/min; Retention time: 15.5 min (major), 18.4 min (minor).

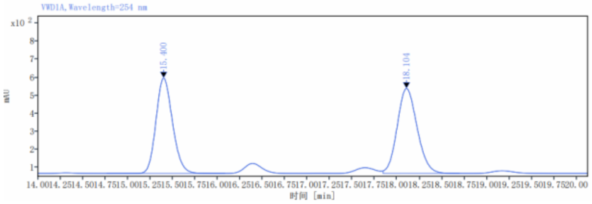

Peak	Retention time/ min	Area%
1	15. 400	48. 20
2	18. 104	51. 80

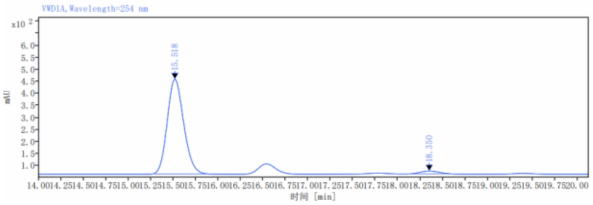

Peak	Retention time/ min	Area%
1	15. 518	96. 99
2	18. 350	3. 01

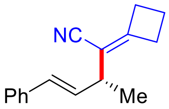

(*E*)-2-Cyclobutylidene-3-methyl-5-phenylpent-4-enenitrile (**4v**)

The reaction time for the first step was 48 h. Ketone **3** (3 equiv) was used. Colorless oil, 55% yield (12.2 mg), $[\alpha]_D^{25}$ +135.0 (*c* 0.54, $CHCl_3$) for 94% ee; ^{1}H NMR (400 MHz, chloroform-*d*) δ 7.40 – 7.34 (m, 2H), 7.31 (t, *J* = 7.6 Hz, 2H), 7.27 – 7.19 (m, 1H), 6.41 (d, *J* = 15.9 Hz, 1H), 6.16 (dd, *J* = 15.8, 7.3 Hz, 1H), 3.15 (p, *J* = 6.9 Hz, 1H), 2.94 (t, *J* = 7.9 Hz, 2H), 2.86 (t, *J* = 7.9 Hz, 2H), 2.09 (p, *J* = 7.9 Hz, 2H), 1.32 (d, *J* = 7.0 Hz, 3H). ^{13}C NMR (101 MHz, chloroform-*d*) δ 162.0, 136.9, 130.9, 129.9, 128.6, 127.6, 126.3, 116.6, 110.4, 36.9, 31.7, 30.7, 19.4, 16.3. HRMS (ESI): [M+H]$^{\oplus}$ calcd for $C_{16}H_{18}N^{\oplus}$ 224.1434, found 224.1435. HPLC analysis: Chiracel OJ-H column; detected at 254 nm, 25 °C; ^{i}PrOH : *n*-hexane = 2 : 98; flow = 1.0 mL/min; Retention time: 16.5 min (minor), 19.5 min (major).

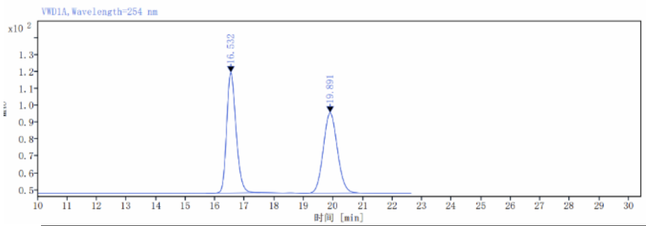

Peak	Retention time/ min	Area%
1	16. 532	50. 13
2	19. 891	49. 87

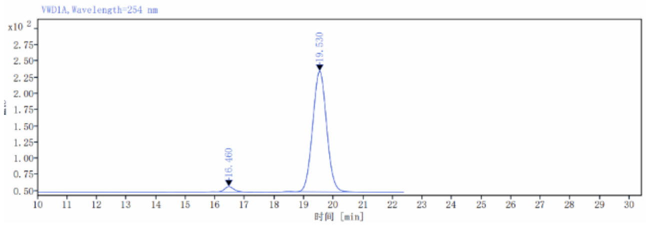

Peak	Retention time/ min	Area%
1	16. 460	2. 74
2	19. 530	97. 26

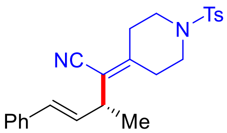

(*E*)-3-Methyl-5-phenyl-2-(1-tosylpiperidin-4-ylidene)pent-4-enenitrile (**4w**)

The reaction time for the first step was 48 h. Ketone **3** (3 equiv) was used. White soild, 70% yield (28.6 mg), $[\alpha]_D^{25}$ +67.9 (*c* 1.1, $CHCl_3$) for 94% ee; ^{1}H NMR (400 MHz, chloroform-*d*) δ 7.63 (d, *J* = 8.1 Hz,

2H), 7.37 – 7.20 (m, 7H), 6.34 (d, *J* = 15.8 Hz, 1H), 6.11 (dd, *J* = 15.9, 7.0 Hz, 1H), 3.43 (p, *J* = 7.2 Hz, 1H), 3.26 – 2.97 (m, 4H), 2.81 – 2.66 (m, 2H), 2.57 – 2.54 (m, 2H), 2.43 (s, 3H), 1.31 (d, *J* = 6.9 Hz, 3H). ^{13}C NMR (101 MHz, chloroform-*d*) δ 151.1, 144.0, 136.6, 133.0, 130.4, 130.3, 129.9, 128.6, 127.8, 127.6, 126.3, 116.6, 114.2, 46.8, 46.4, 35.9, 33.8, 29.5, 21.6, 19.8. HRMS (ESI): $[M+Na]^{\oplus}$ calcd for $C_{24}H_{26}O_2N_2NaS^{\oplus}$ 429.1607, found 429.1606. HPLC analysis: Chiracel OD-H column; detected at 254 nm, 40 °C; ^{i}PrOH : *n*-hexane = 20 : 80; flow = 1.0 mL/min; Retention time: 19.4 min (major), 29.0 min (minor).

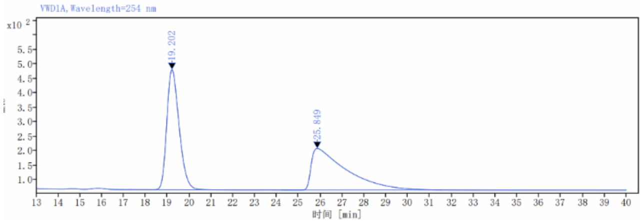

Peak	Retention time/ min	Area%
1	19. 202	50. 50
2	25. 849	49. 50

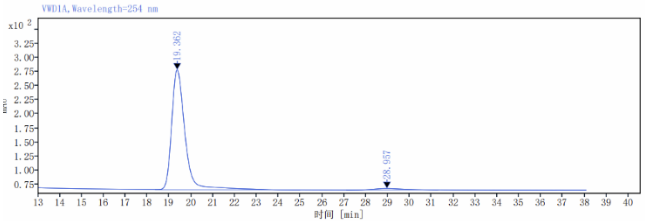

Peak	Retention time/ min	Area%
1	19. 362	97. 27
2	28. 957	2. 73

5. General procedure for formal hydroallenylation

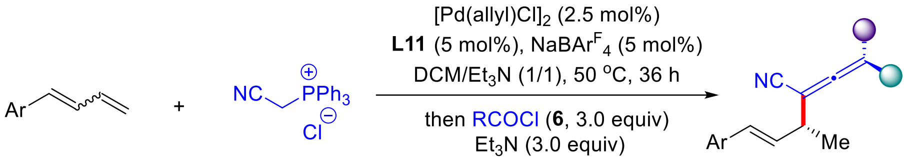

1 (1.0 equiv)
(Z/E mixture)

5 (1.0 equiv)

7

General procedure (**7a** as an example, standard condition): In a N_2-filled glovebox, $[Pd(allyl)Cl]_2$ (0.9 mg, 0.0025 mmol), **L11** (2.8 mg, 0.0050 mmol), $NaBAr^F{}_4$ (4.4 mg, 0.0050 mmol), (cyanomethyl)triphenylphosphonium chloride **5** (34 mg, 0.10 mmol) and DCM/Et_3N (v/v = 1:1, 0.15 mL) were added sequentially to a 4 mL vial. The resulting yellow solution was allowed to stir at room temperature for 1 min. Then diene **1a** (13 mg, 0.10 mmol) was added to the reaction. The resulting solution continued to stir at 50 °C for 36 h. After this time, the reaction solvent was removed. Next dry THF (1.0 mL) and Et_3N (42 μL, 0.30 mmol, 3.0 equiv) were added to the reaction sequentially. The resulting solution was allowed to stir at room temperature for 3 min. Then acetyl chloride (24 mg, 0.30 mmol) was added to the solution slowly, and the resulting mixture continued to stir for additional 8 h at room temperature. After this time, the reaction was quenched with saturated $NaHCO_3$ aqueous solution (3 mL), extracted with CH_2Cl_2 (5.0 mL × 3), dried over Na_2SO_4, filtered, concentrated and purified by flash silica gel chromatography (*n*-hexane/ethyl acetate = 20:1) to give the pure product **7a**.

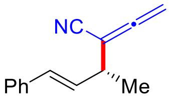

(*E*)-3-Methyl-5-phenyl-2-vinylidenepent-4-enenitrile (7a)

Yellow oil, 83% yield (16.2 mg), $[\alpha]_D^{25}$ -26.1 (*c* 0.71, $CHCl_3$) for 94% ee; ^{1}H NMR (400 MHz, chloroform-*d*) δ 7.37 (d, *J* = 7.2 Hz, 2H), 7.31 (t, *J* = 7.5 Hz, 2H), 7.28 – 7.20 (m, 1H), 6.51 (d, *J* = 15.8 Hz, 1H), 6.11 (dd, *J* = 15.8, 7.7 Hz, 1H), 5.31 (d, *J* = 2.7 Hz, 2H), 3.22 – 3.15 (m, 1H), 1.33 (d, *J* = 6.9

Hz, 3H). ^{13}C NMR (101 MHz, chloroform-*d*) δ 214.0, 136.6, 131.3, 129.9, 128.6, 127.8, 126.5, 114.6, 86.9, 81.9, 38.7, 19.1. HRMS (ESI): $[M+Na]^{\oplus}$ calcd for $C_{14}H_{13}NNa^{\oplus}$ 218.0940, found 218.0941. HPLC analysis: Chiracel OJ-H column; detected at 254 nm, 25 °C; ^{i}PrOH : *n*-hexane = 10 : 90; flow = 0.7 mL/min; Retention time: 16.8 min (major), 17.9 min (minor).

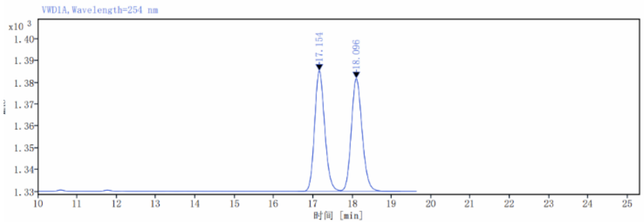

Peak	Retention time/ min	Area%
1	17. 154	49. 92
2	18. 096	50. 08

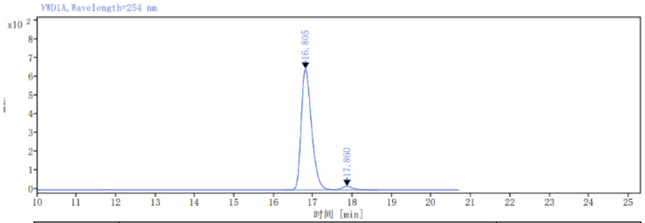

Peak	Retention time/ min	Area%
1	16. 805	97. 00
2	17. 860	3. 00

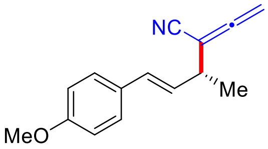

(*E*)-5-(4-Methoxyphenyl)-3-methyl-2-vinylidenepent-4-enenitrile (**7b**)

Yellow oil, 72% yield (16.2 mg), $[\alpha]_D^{25}$ -16.4 (*c* 0.76, $CHCl_3$) for 94% ee; ^{1}H NMR (400 MHz, chloroform-*d*) δ 7.34 – 7.28 (m, 2H), 6.89 – 6.80 (m, 2H), 6.46 (d, *J* = 15.7 Hz, 1H), 5.97 (dd, *J* = 15.8, 7.7 Hz, 1H), 5.30 (dd, *J* = 2.6, 1.0 Hz, 2H), 3.81 (s, 3H), 3.23 – 3.11 (m, 1H), 1.32 (d, *J* = 6.9 Hz, 3H). ^{13}C NMR (101 MHz, chloroform-*d*) δ 214.0, 159.3, 130.7, 129.4, 127.7, 127.6, 114.7, 114.0, 87.1, 81.8, 55.4, 38.8, 19.2. HRMS (ESI): $[M+H]^{\oplus}$ calcd for $C_{15}H_{16}ON^{\oplus}$ 226.1226, found 226.1227. HPLC analysis: Chiracel AD-H column; detected at 254 nm, 25 °C; ^{i}PrOH : *n*-hexane = 2 : 98; flow = 0.7 mL/min; Retention time: 14.0 min (major), 15.0 min (minor).

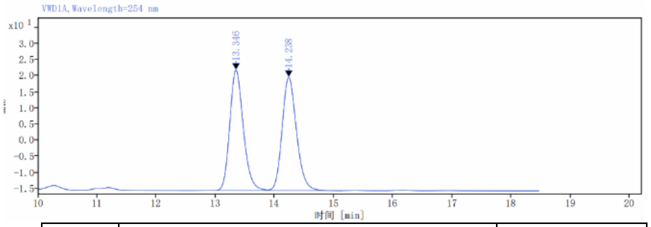

Peak	Retention time/ min	Area%
1	13. 346	50. 11
2	14. 238	49. 89

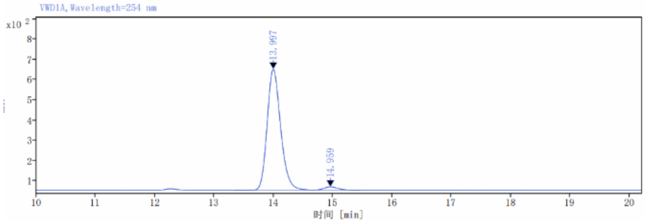

Peak	Retention time/ min	Area%
1	13. 997	97. 15
2	14. 959	2. 85

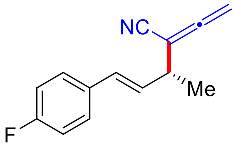

(*E*)-5-(4-Fluorophenyl)-3-methyl-2-vinylidenepent-4-enenitrile (**7c**)

Yellow oil, 81% yield (17.2 mg), $[\alpha]_D^{25}$ -27.4 (*c* 0.84, $CHCl_3$) for 95% ee; ^{1}H NMR (400 MHz, chloroform-*d*) δ 7.40 – 7.30 (m, 2H), 7.00 (dd, *J* = 9.8, 7.6 Hz, 2H), 6.48 (d, *J* = 15.8 Hz, 1H), 6.02 (dd, *J* = 15.8, 7.7 Hz, 1H), 5.37 – 5.28 (m, 2H), 3.24 – 3.11 (m, 1H), 1.33 (d, *J* = 6.9 Hz, 3H). ^{13}C NMR (101

MHz, chloroform-*d*) δ 214.0, 162.4 (d, *J* = 247.1 Hz), 132.8 (d, *J* = 3.4 Hz), 130.2, 129.7 (d, *J* = 2.3 Hz), 128.0 (d, *J* = 8.1 Hz), 115.6 (d, *J* = 21.6 Hz), 114.6, 86.9, 81.9, 38.7, 19.1. ^{19}F NMR (376 MHz, chloroform-*d*) δ -114.1 – -114.6 (m, 1F). HRMS (ESI): [M+Na]$^{\oplus}$ calcd for $C_{14}H_{12}NFNa^{\oplus}$ 236.0846, found 236.0844. HPLC analysis: Chiracel AD-H column; detected at 254 nm, 25 °C; ^{i}PrOH : *n*-hexane = 2 : 98; flow = 0.7 mL/min; Retention time: 9.7 min (major), 10.4 min (minor).

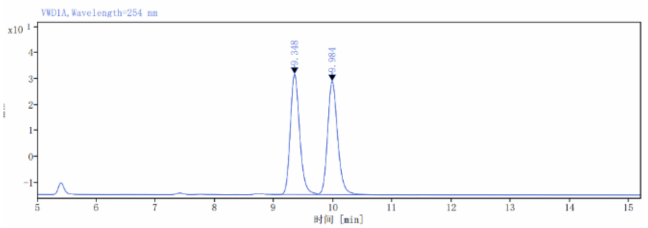

Peak	Retention time/ min	Area%
1	9.348	49.72
2	9.984	50.28

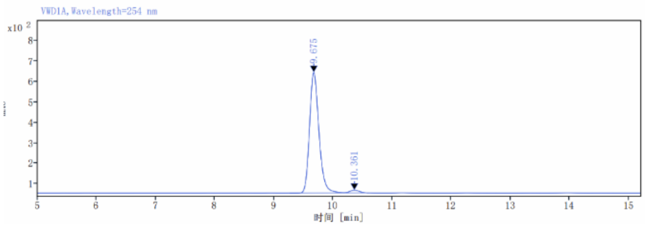

Peak	Retention time/ min	Area%
1	9.675	97.60
2	10.361	2.40

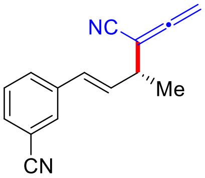

(*E*)-3-(4-Cyano-3-methylhexa-1,4,5-trien-1-yl)benzonitrile (**7d**)

Yellow oil, 85% yield (18.7 mg), $[\alpha]_D^{25}$ -32.6 (*c* 0.81, $CHCl_3$) for 94% ee; ^{1}H NMR (400 MHz, chloroform-*d*) δ 7.64 (s, 1H), 7.58 (d, *J* = 7.8 Hz, 1H), 7.52 (d, *J* = 7.8 Hz, 1H), 7.42 (t, *J* = 7.7 Hz, 1H), 6.50 (d, *J* = 15.8 Hz, 1H), 6.18 (dd, *J* = 15.8, 7.7 Hz, 1H), 5.35 (d, *J* = 2.6 Hz, 2H), 3.27 – 3.16 (m, 1H), 1.34 (d, *J* = 6.9 Hz, 3H). ^{13}C NMR (126 MHz, chloroform-*d*) δ 214.0, 137.8, 132.8, 131.0, 130.7, 129.9, 129.5, 129.3, 118.7, 114.4, 112.8, 86.5, 82.2, 38.6, 18.8. HRMS (ESI): [M+H]$^{\oplus}$ calcd for $C_{15}H_{13}N_2^{\oplus}$ 221.1073, found 221.1074. HPLC analysis: Chiracel OJ-H column; detected at 254 nm, 25 °C; ^{i}PrOH : *n*-hexane = 20 : 80; flow = 1.0 mL/min; Retention time: 11.4 min (major), 13.7 min (minor).

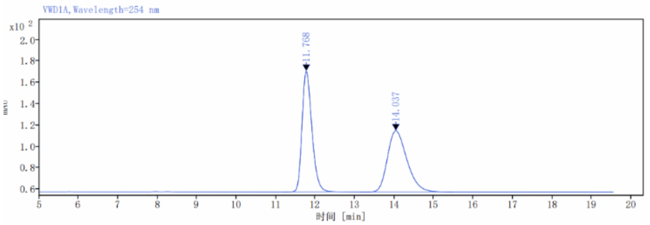

Peak	Retention time/ min	Area%
1	11.768	49.99
2	14.037	50.01

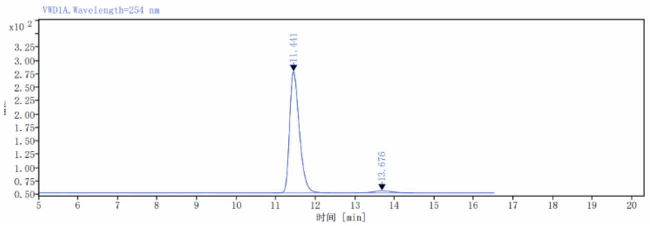

Peak	Retention time/ min	Area%
1	11.441	96.96
2	13.676	3.04

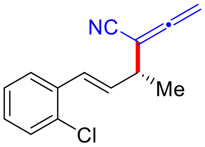

(*E*)-5-(2-Chlorophenyl)-3-methyl-2-vinylidenepent-4-enenitrile (**7e**)

Yellow oil, 84% yield (19.4 mg), $[\alpha]_D^{25}$ -25.9 (*c* 0.83, $CHCl_3$) for 90% ee; ^{1}H NMR (400 MHz, chloroform-*d*) δ 7.51 (dd, *J* = 7.4, 2.0 Hz, 1H), 7.35 (dd, *J* = 7.5, 1.8 Hz, 1H), 7.24 - 7.16 (m, 2H), 6.90 (d, *J* = 15.8 Hz, 1H), 6.10 (dd, *J* = 15.8, 7.7 Hz, 1H), 5.33 (d, *J* = 2.7 Hz, 2H), 3.31 – 3.20 (m, 1H), 1.36 (d, *J* = 6.9 Hz, 3H). ^{13}C NMR (126 MHz, chloroform-*d*) δ 214.1, 134.8, 133.1, 132.7, 129.7, 128.8, 127.7, 127.0, 126.9, 114.5, 86.8, 82.0, 38.8, 19.1. HRMS (ESI): $[M+Na]^{\oplus}$ calcd for $C_{14}H_{12}NClNa^{\oplus}$ 252.0550, found 252.0549. HPLC analysis: Chiracel OJ-H column; detected at 254 nm, 25 °C; ^{i}PrOH : *n*-hexane = 2 : 98; flow = 0.7 mL/min; Retention time: 18.0 min (major), 19.2 min (minor).

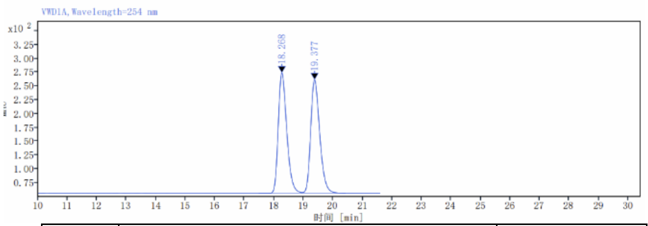

Peak	Retention time/ min	Area%
1	18. 268	49. 92
2	19. 377	50. 08

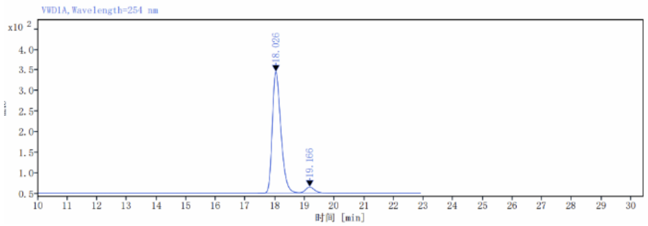

Peak	Retention time/ min	Area%
1	18. 026	95. 21
2	19. 166	4. 79

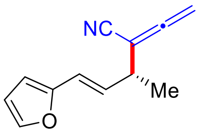

(*E*)-5-(Furan-2-yl)-3-methyl-2-vinylidenepent-4-enenitrile (**7f**)

Yellow oil, 58% yield (10.8 mg), $[\alpha]_D^{25}$ +3.2 (*c* 0.62, $CHCl_3$) for 95% ee; ^{1}H NMR (400 MHz, chloroform-*d*) δ 7.34 (d, *J* = 1.8 Hz, 1H), 6.40 – 6.29 (m, 2H), 6.24 (d, *J* = 3.3 Hz, 1H), 6.06 (dd, *J* = 15.8, 7.6 Hz, 1H), 5.31 (d, *J* = 2.6 Hz, 2H), 3.24 – 3.06 (m, 1H), 1.31 (d, *J* = 6.9 Hz, 3H). ^{13}C NMR (101 MHz, chloroform-*d*) δ 214.0, 152.1, 142.1, 128.5, 119.7, 114.6, 111.4, 108.2, 86.7, 82.0, 38.4, 18.9. HRMS (ESI): $[M+H]^{\oplus}$ calcd for $C_{12}H_{12}ON^{\oplus}$ 186.0913, found 186.0914. HPLC analysis: Chiracel OJ-H column; detected at 254 nm, 25 °C; ^{i}PrOH : *n*-hexane = 12 : 88; flow = 1.0 mL/min; Retention time: 12.2 min (major), 13.3 min (minor).

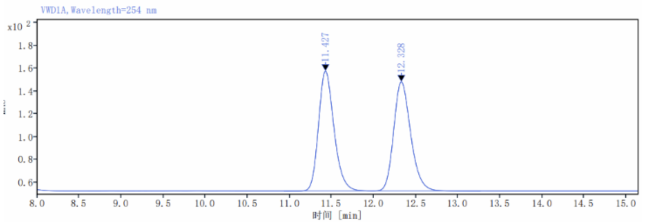

Peak	Retention time/ min	Area%
1	11. 427	50. 00
2	12. 328	50. 00

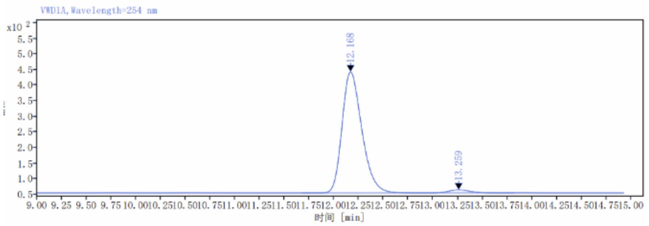

Peak	Retention time/ min	Area%
1	12. 168	97. 46
2	13. 259	2. 54

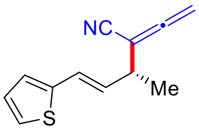

(*E*)-3-Methyl-5-(thiophen-2-yl)-2-vinylidenepent-4-enenitrile (**7g**)

Yellow oil, 80% yield (16.0 mg), $[\alpha]_D^{25}$ -8.6 (*c* 0.71, $CHCl_3$) for 95% ee; ^{1}H NMR (400 MHz, chloroform-*d*) δ 7.19 – 7.13 (m, 1H), 7.00 – 6.92 (m, 2H), 6.64 (d, *J* = 15.6 Hz, 1H), 5.95 (dd, *J* = 15.6, 7.6 Hz, 1H), 5.32 (d, *J* = 2.6 Hz, 2H), 3.22 – 3.10 (m, 1H), 1.32 (d, *J* = 6.9 Hz, 3H). ^{13}C NMR (101 MHz, chloroform-*d*) δ 214.0, 141.6, 129.4, 127.4, 126.0, 124.5, 124.4, 114.6, 86.7, 82.0, 38.6, 18.9. HRMS (ESI): $[M+H]^{\oplus}$ calcd for $C_{12}H_{12}NS^{\oplus}$ 202.0685, found 202.0685. HPLC analysis: Chiracel OJ-H column; detected at 254 nm, 25 ^oC; ^{i}PrOH : *n*-hexane = 12 : 88; flow = 1.0 mL/min; Retention time: 12.1 min (major), 13.3 min (minor).

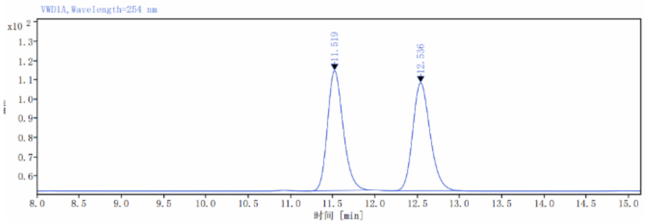

Peak	Retention time/ min	Area%
1	11. 519	50. 04
2	12. 536	49. 96

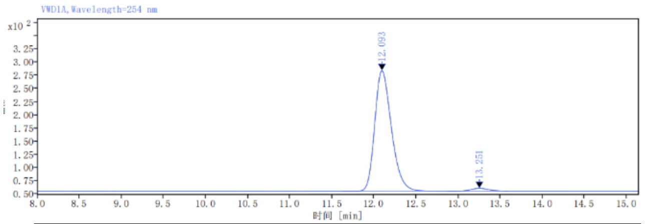

Peak	Retention time/ min	Area%
1	12. 093	97. 65
2	13. 251	2. 35

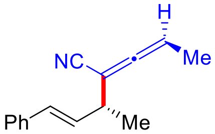

(*E*)-2-(4-Phenylbut-3-en-2-yl)penta-2,3-dienenitrile (7h)

Yellow oil, 77% yield (16.0 mg), >20:1 dr, $[\alpha]_D^{25}$ -38.5 (*c* 0.70, $CHCl_3$) for 94% ee; ^{1}H NMR (400 MHz, chloroform-*d*) δ 7.40 – 7.34 (m, 2H), 7.31 (t, *J* = 7.6 Hz, 2H), 7.27 – 7.21 (m, 1H), 6.50 (d, *J* = 15.8 Hz, 1H), 6.10 (dd, *J* = 15.8, 7.7 Hz, 1H), 5.67 (qd, *J* = 7.4, 2.4 Hz, 1H), 3.24 – 3.07 (m, 1H), 1.79 (d, *J* = 7.4 Hz, 3H), 1.31 (d, *J* = 6.9 Hz, 3H). ^{13}C NMR (126 MHz, chloroform-*d*) δ 211.0, 136.8, 131.0, 130.4, 128.6, 127.7, 126.4, 115.3, 93.2, 86.4, 39.2, 19.2, 13.4. HRMS (EI): $[M]^{\oplus}$ calcd for $C_{15}H_{15}N^{\oplus}$ 209.1199, found 209.1194. HPLC analysis: Chiracel OJ-H column; detected at 254 nm, 20 ^oC; ^{i}PrOH : *n*-hexane = 1 : 99; flow = 1.0 mL/min; Retention time: 21.5 min (major), 22.7 min (minor).

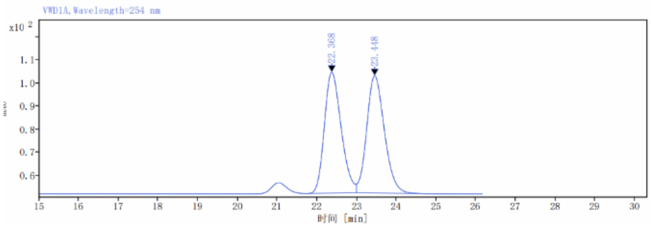

Peak	Retention time/ min	Area%
1	22. 368	49. 45
2	23. 448	50. 55

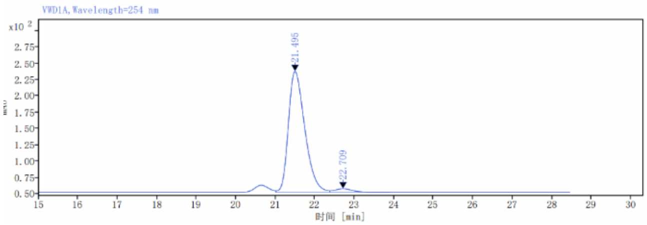

Peak	Retention time/ min	Area%
1	21. 495	97. 03
2	22. 709	2. 97

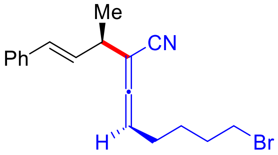

(*E*)-8-Bromo-2-(4-phenylbut-3-en-2-yl)octa-2,3-dienenitrile (7i)

Colorless oil, 74% yield (24.5 mg), >20:1 dr, $[\alpha]_D^{25}$ -2.0 (*c* 1.2, $CHCl_3$) for 94% ee; ^{1}H NMR (500 MHz, chloroform-*d*) δ 7.37 (d, *J* = 7.7 Hz, 2H), 7.31 (t, *J* = 7.7 Hz, 2H), 7.27 – 7.21 (m, 1H), 6.50 (d, *J* = 15.9 Hz, 1H), 6.10 (ddd, *J* = 15.8, 7.7, 1.7 Hz, 1H), 5.72 – 5.68 (m, 1H), 3.37 (t, *J* = 6.6 Hz, 2H), 3.20 – 3.14 (m, 1H), 2.17 (q, *J* = 7.3 Hz, 2H), 1.92 – 1.85 (m, 2H), 1.61 (p, *J* = 7.5 Hz, 2H), 1.32 (d, *J* = 6.8 Hz, 3H). ^{13}C NMR (101 MHz, chloroform-*d*) δ 210.3, 136.7, 131.1, 130.2, 128.6, 127.7, 126.4, 115.2, 97.8, 87.5, 39.2, 33.3, 31.9, 27.1, 27.0, 19.2. HRMS (ESI): $[M+H]^{\oplus}$ calcd for $C_{18}H_{21}NBr^{\oplus}$ 330.0852, found 330.0851. HPLC analysis: Chiracel AD-H column; detected at 254 nm, 25 °C; ^{i}PrOH : *n*-hexane = 2 : 98; flow = 0.7 mL/min; Retention time: 10.6 min (major), 12.7 min (minor).

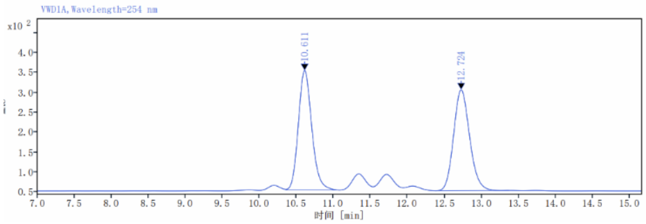

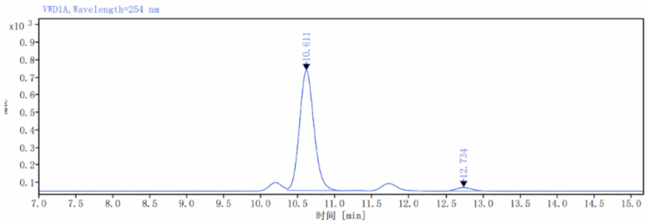

Peak	Retention time/ min	Area%
1	10. 611	49. 91
2	12. 724	50. 09

Peak	Retention time/ min	Area%
1	10. 611	96. 96
2	12. 734	3. 04

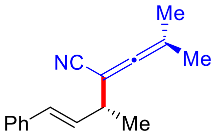

(*E*)-4-Methyl-2-(4-phenylbut-3-en-2-yl)penta-2,3-dienenitrile (7j)

Yellow oil, 70% yield (15.5 mg), $[\alpha]_D^{25}$ -63.1 (*c* 0.70, $CHCl_3$) for 95% ee; ^{1}H NMR (400 MHz, chloroform-*d*) δ 7.40 – 7.35 (m, 2H), 7.31 (t, *J* = 7.6 Hz, 2H), 7.27 – 7.21 (m, 1H), 6.50 (d, *J* = 15.8 Hz, 1H), 6.10 (dd, *J* = 15.8, 7.6 Hz, 1H), 3.13 (p, *J* = 7.1 Hz, 1H), 1.81 (d, *J* = 1.7 Hz, 6H), 1.28 (d, *J* = 6.9 Hz, 3H). ^{13}C NMR (126 MHz, chloroform-*d*) δ 208.4, 136.9, 130.9, 130.8, 128.6, 127.6, 126.4, 115.9, 103.6, 84.6, 39.5, 19.82, 19.80, 19.2. HRMS (ESI): $[M+H]^{\oplus}$ calcd for $C_{16}H_{18}N^{\oplus}$ 224.1434, found 224.1435. HPLC analysis: Chiracel OJ-H column; detected at 254 nm, 25 °C; ^{i}PrOH : *n*-hexane = 2 : 98; flow = 0.7 mL/min; Retention time: 14.6 min (major), 16.7 min (minor).

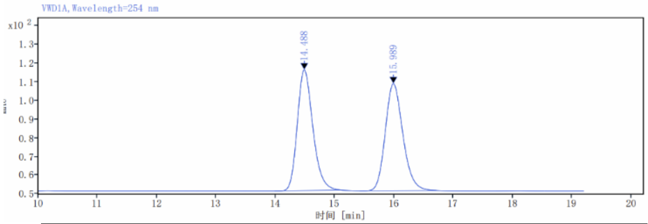

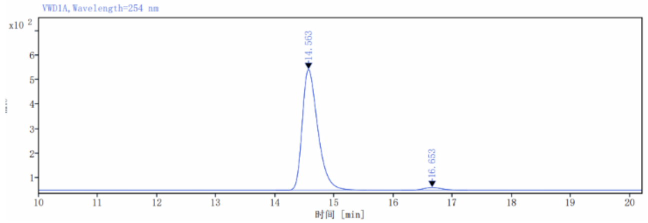

Peak	Retention time/ min	Area%
1	14. 488	49. 93
2	15. 989	50. 07

Peak	Retention time/ min	Area%
1	14. 563	97. 49
2	16. 653	2. 51

6. General procedure for formal hydroketenimination

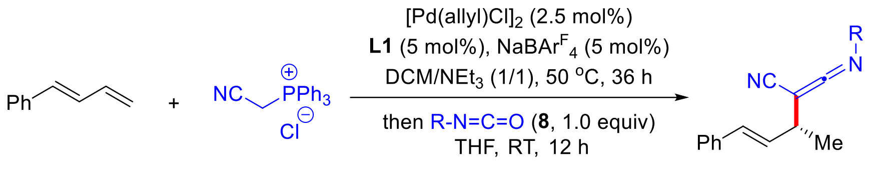

1 (1.0 equiv) **5** (1.0 equiv) **9**

General procedure (**9a** as an example, standard condition): In a N_2-filled glovebox, $[Pd(allyl)Cl]_2$ (0.9 mg, 0.0025 mmol), **L11** (2.8 mg, 0.0050 mmol), $NaBAr^F{}_4$ (4.4 mg, 0.005 mmol), (cyanomethyl)triphenylphosphonium chloride **5** (34 mg, 0.10 mmol) and DCM/Et_3N (v/v = 1:1, 0.15 mL) were added sequentially to a 4 mL vial. The resulting yellow solution was allowed to stir at room temperature for 1 min. Then diene **1a** (13 mg, 0.10 mmol) was added to the reaction and the resulting mixture was allowed to stir at 50 °C for 36 h. After this time, the reaction solvent was removed. Then dry THF (0.50 mL) and 1-isocyanato-4-methoxybenzene **8a** (15 mg, 0.10 mmol) were added to the reaction sequentially. The resulting reaction solution continued to stir for another 12 h at room temperature. After this time, the reaction was concentrated and purified by flash silica gel chromatography to give the pure **9a**.

Note: as compounds **9** are sensitive to silica gel, a quick purification process is required.

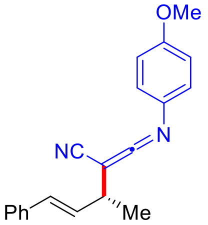

(*E*)-2-(((4-Methoxyphenyl)imino)methylene)-3-methyl-5-phenylpent-4-enenitrile (9a)

Yellow oil, 44% yield (13.3 mg) (the yield determined by crude ^{1}H NMR was 70%), >20:1 dr, $[\alpha]_D^{25}$ -33.6 (*c* 0.63, $CHCl_3$) for 94% ee; ^{1}H NMR (400 MHz, chloroform-*d*) δ 7.38 – 7.22 (m, 7H), 6.95 – 6.87 (m, 2H), 6.55 (d, *J* = 15.8 Hz, 1H), 6.17 (dd, *J* = 15.8, 7.6 Hz, 1H), 3.83 (s, 3H), 3.29 (p, *J* = 7.0 Hz, 1H), 1.38 (d, *J* = 6.9 Hz, 3H). ^{13}C NMR (101 MHz, chloroform-*d*) δ 178.0, 160.5, 136.7, 131.00, 130.96, 128.9, 128.6, 127.8, 126.5, 115.5, 115.1, 55.7, 51.0, 35.9, 20.1 (one aryl carbon signal was not observed because of overlapping). HRMS (EI): $[M]^{\oplus}$ calcd for $C_{20}H_{18}ON_2{}^{\oplus}$ 302.1414, found 302.1411. HPLC analysis: Chiracel AD-H column; detected at 254 nm, 25 °C; ^{i}PrOH : *n*-hexane = 10 : 90; flow = 0.7 mL/min; Retention time: 12.7 min (major), 13.4 min (minor).

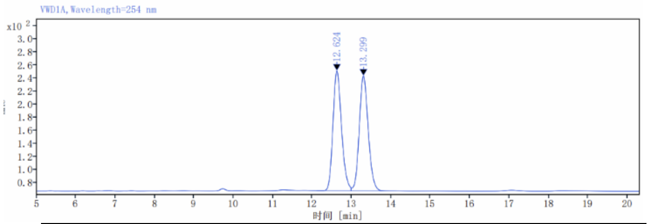

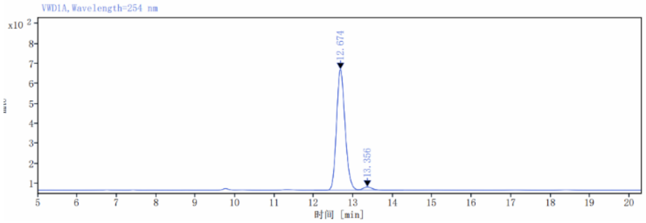

Peak	Retention time/ min	Area%
1	12. 624	49. 94
2	13. 299	50. 06

Peak	Retention time/ min	Area%
1	12. 674	97. 24
2	13. 356	2. 76

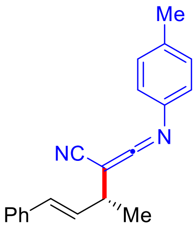

(*E*)-3-Methyl-5-phenyl-2-((p-tolylimino)methylene)pent-4-enenitrile (9b)

Yellow oil, 58% yield (16.8 mg), >20:1 dr, $[\alpha]_D^{25}$ -32.7 (*c* 0.47, $CHCl_3$) for 94% ee; ^{1}H NMR (400 MHz, chloroform-*d*) δ 7.35 (d, *J* = 7.1 Hz, 2H), 7.30 (t, *J* = 7.5 Hz, 2H), 7.27 – 7.16 (m, 5H), 6.56 (d, *J* = 15.8 Hz, 1H), 6.17 (dd, *J* = 15.8, 7.6 Hz, 1H), 3.30 (p, *J* = 7.0 Hz, 1H), 2.38 (s, 3H), 1.39 (d, *J* = 6.9 Hz, 3H). ^{13}C NMR (101 MHz, chloroform-*d*) δ 178.7, 139.9, 136.6, 133.9, 131.0, 130.9, 130.5, 128.6, 127.8, 126.5, 124.7, 115.4, 51.0, 35.8, 21.3, 20.1. HRMS (EI): $[M]^{\oplus}$ calcd for $C_{20}H_{18}N_2^{\oplus}$ 286.1465, found 286.1461. HPLC analysis: Chiracel OD-H column; detected at 254 nm, 25 °C; ^{i}PrOH : *n*-hexane = 2 : 98; flow = 0.7 mL/min; Retention time: 8.6 min (minor), 9.1 min (major).

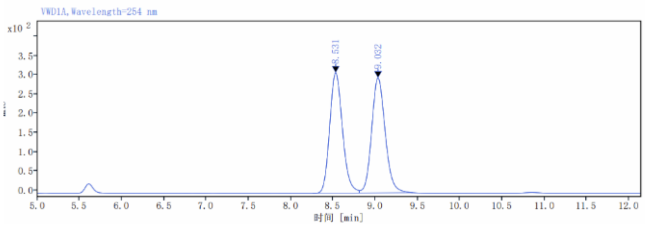

Peak	Retention time/ min	Area%
1	8. 531	49. 78
2	9. 032	50. 22

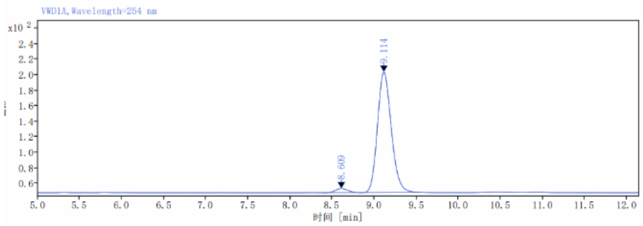

Peak	Retention time/ min	Area%
1	8. 609	2. 95
2	9. 114	97. 05

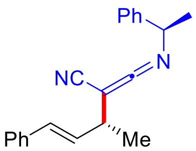

(3*R*,*E*)-3-Methyl-5-phenyl-2-((((*R*)-1-phenylethyl)imino)methylene)pent-4-enenitrile (9c)

(*R*)-(1-isocyanatoethyl)benzene **8c** (2.0 equiv) was adopted. Yellow oil, 67% yield (19.7 mg), >20:1 dr, $[\alpha]_D^{25}$ -33.1 (*c* 0.92, $CHCl_3$); ^{1}H NMR (400 MHz, chloroform-*d*) δ 7.34 – 7.23 (m, 10H), 6.40 (d, *J* = 15.7 Hz, 1H), 5.94 (dd, *J* = 15.8, 7.6 Hz, 1H), 4.91 (q, *J* = 6.8 Hz, 1H), 3.09 (p, *J* = 7.1 Hz, 1H), 1.63 (d, *J* = 6.9 Hz, 3H), 1.21 (d, *J* = 6.9 Hz, 3H). ^{13}C NMR (101 MHz, chloroform-*d*) δ 176.6, 140.4, 136.6, 130.80, 130.78, 128.9, 128.6, 128.4, 127.7, 126.4, 126.2, 116.1, 62.6, 50.2, 35.0, 23.1, 19.9. HRMS (ESI): $[M+Na]^{\oplus}$ calcd for $C_{21}H_{20}N_2Na^{\oplus}$ 323.1519, found 323.1515.

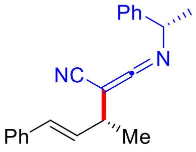

(3*R*,*E*)-3-Methyl-5-phenyl-2-((((*S*)-1-phenylethyl)imino)methylene)pent-4-enenitrile (9d)

(*S*)-(1-isocyanatoethyl)benzene **8d** (2.0 equiv) was adopted. Yellow oil, 60% yield (17.9 mg), >20:1 dr, $[\alpha]_D^{25}$ -12.5 (*c* 0.77, $CHCl_3$); ^{1}H NMR (500 MHz, chloroform-*d*) δ 7.34 – 7.23 (m, 10H), 6.41 (dd, *J* = 15.9, 1.2 Hz, 1H), 5.95 (dd, *J* = 15.8, 7.6 Hz, 1H), 4.91 (q, *J* = 6.8 Hz, 1H), 3.15 – 3.03 (m, 1H), 1.63 (d,

J = 6.9 Hz, 3H), 1.22 (d, J = 6.8 Hz, 3H). ^{13}C NMR (101 MHz, chloroform-d) δ 176.5, 140.4, 136.6, 130.80, 130.79, 128.9, 128.6, 128.4, 127.7, 126.5, 126.2, 116.1, 62.6, 50.1, 35.0, 23.1, 19.9. HRMS (ESI): $[M+H]^{\oplus}$ calcd for $C_{21}H_{21}N_2^{\oplus}$ 301.1699, found 301.1698.

7. General procedure for formal hydroalkenylation of alkynes

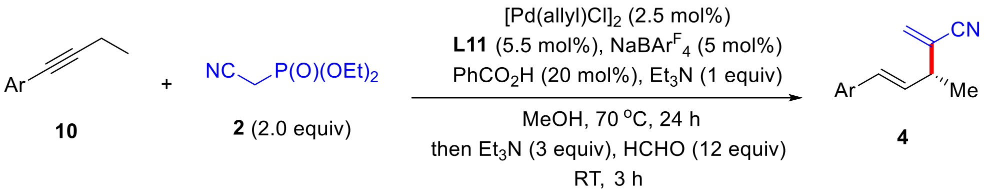

General procedure: in a N_2-filled glovebox, $[Pd(allyl)Cl]_2$ (0.9 mg, 0.0050 mmol), **L11** (3.1 mg, 0.0055 mmol), sodium tetrakis[3,5-bis(trifluoromethyl)phenyl]borate ($NaBAr^F{}_4$, 4.4 mg, 0.0050 mmol), benzoic acid (2.4 mg, 0.020 mmol) and dry MeOH (0.20 mL) were added to a 4 mL vial sequentially. The resulting mixture was stirred at room temperature for 5 min. Then Et_3N (14 μL, 0.10 mmol), alkyne **10** (0.10 mmol) and phosphonate **2** (35 mg, 0.20 mmol) were added to the reaction sequentially. The resulting mixture continued to stir at 70 °C for 24 h. After this time, the reaction solution was cooled to room temperature. Et_3N (42 μL, 0.30 mmol) and HCHO aqueous solution (0.10 mL, 37 wt.%, 1.2 mmol) were added to the reaction. Then resulting mixture continued to stir at room temperature for 3 h. After this time, the reaction was condensed and purified by flash column chromatography to give **4**.

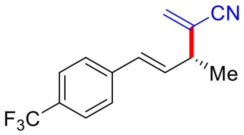

(*E*)-3-Methyl-2-methylene-5-(4-(trifluoromethyl)phenyl)pent-4-enenitrile (4x)

Colorless oil, 55% yield (13.8 mg), $[\alpha]_D^{25}$ +19.8 (c 0.39, $CHCl_3$) for 84% ee; ^{1}H NMR (400 MHz, chloroform-d) δ 7.57 (d, J = 8.1 Hz, 2H), 7.47 (d, J = 8.1 Hz, 2H), 6.53 (d, J = 15.9 Hz, 1H), 6.23 (dd, J = 15.9, 7.3 Hz, 1H), 5.92 (s, 1H), 5.82 (d, J = 1.2 Hz, 1H), 3.28 (p, J = 6.9 Hz, 1H), 1.39 (d, J = 6.9 Hz, 3H). ^{13}C NMR (101 MHz, chloroform-d) δ 144.1, 132.5, 130.4, 129.7 (q, J = 32.5 Hz),129.5, 127.0, 126.6, 125.6 (q, J = 3.8 Hz), 124.2 (q, J = 271.1 Hz), 117.9, 41.7, 18.8. ^{19}F NMR (376 MHz, chloroform-d) δ -62.57 (s, 1F). HRMS (EI): $[M]^{\oplus}$ calcd for $C_{14}H_{12}NF_3^{\oplus}$ 251.0916, found 251.0912. HPLC analysis: Chiracel (OD-H) + (OD-H) column (these two columns were connected to each other); detected at 254 nm, 20 °C; ^{i}PrOH : n-hexane = 0.5 : 99.5; flow = 0.5 mL/min; Retention time: 31.2 min (major), 32.4 min (minor).

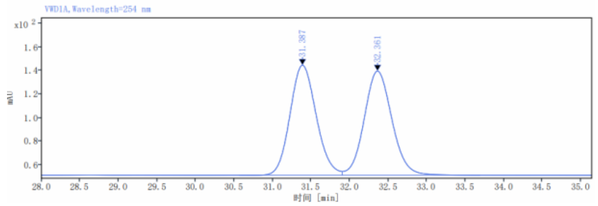

Peak	Retention time/ min	Area%
1	31. 387	50. 03
2	32. 361	49. 97

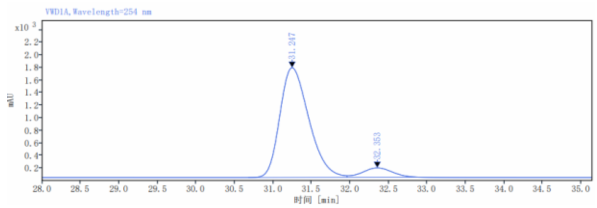

Peak	Retention time/ min	Area%
1	31. 247	91. 98
2	32. 353	8. 02

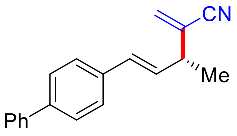

(*E*)-5-([1,1'-Biphenyl]-4-yl)-3-methyl-2-methylenepent-4-enenitrile (4y)

White solid, 50% yield (13.0 mg), $[\alpha]_D^{25}$ +36.8 (*c* 0.35, $CHCl_3$) for 87% ee; ^1H NMR (400 MHz, chloroform-*d*) δ 7.62 – 7.55 (m, 4H), 7.45 (t, *J* = 7.7 Hz, 4H), 7.38 – 7.33 (m, 1H), 6.54 (d, *J* = 15.9 Hz, 1H), 6.18 (dd, *J* = 15.9, 7.3 Hz, 1H), 5.91 (s, 1H), 5.82 (d, *J* = 1.3 Hz, 1H), 3.27 (p, *J* = 7.1 Hz, 1H), 1.40 (d, *J* = 6.9 Hz, 3H). ^{13}C NMR (101 MHz, chloroform-*d*) δ 140.7, 140.6, 135.7, 131.2, 129.8, 129.3, 128.9, 127.5, 127.4, 127.37, 127.0, 126.9, 118.1, 41.8, 19.0. HRMS (ESI): $[M+Na]^{\oplus}$ calcd for $C_{19}H_{17}NNa^{\oplus}$ 282.1253, found 282.1251. HPLC analysis: Chiracel AD-H column; detected at 254 nm, 25 °C; ^{i}PrOH : *n*-hexane = 10 : 90; flow = 1.0 mL/min; Retention time: 7.0 min (major), 7.6 min (minor).

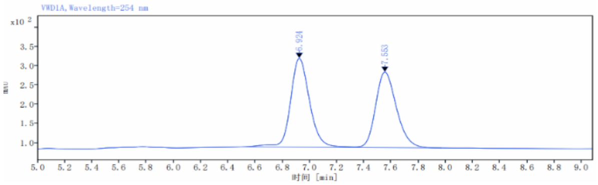

Peak	Retention time/ min	Area%
1	6. 924	51. 84
2	7. 553	48. 16

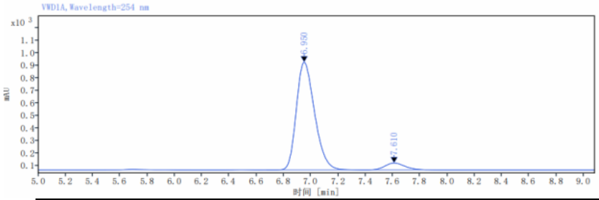

Peak	Retention time/ min	Area%
1	6. 950	93. 66
2	7. 610	6. 34

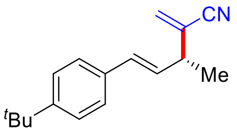

(*E*)-5-(4-(*tert*-Butyl)phenyl)-3-methyl-2-methylenepent-4-enenitrile (4z)

Yellow oil, 56% yield (13.4 mg), $[\alpha]_D^{25}$ +27.4 (*c* 0.50, $CHCl_3$) for 88% ee; ^1H NMR (400 MHz, chloroform-*d*) δ 7.38 – 7.30 (m, 4H), 6.47 (d, *J* = 15.8 Hz, 1H), 6.08 (dd, *J* = 15.9, 7.4 Hz, 1H), 5.88 (s, 1H), 5.79 (d, *J* = 1.3 Hz, 1H), 3.23 (p, *J* = 7.1 Hz, 1H), 1.37 (d, *J* = 6.9 Hz, 3H), 1.32 (s, 9H). ^{13}C NMR (101 MHz, chloroform-*d*) δ 151.0, 133.9, 131.4, 129.1, 128.9, 127.7, 126.2, 125.6, 118.1, 41.7, 34.7, 31.4, 19.1. HRMS (ESI): $[M+Na]^{\oplus}$ calcd for $C_{17}H_{21}NNa^{\oplus}$ 262.1566, found 262.1564. HPLC analysis: Chiracel OJ-H column; detected at 254 nm, 25 °C; ^{i}PrOH : *n*-hexane = 10 : 90; flow = 0.7 mL/min; Retention time: 12.3 min (major), 14.2 min (minor).

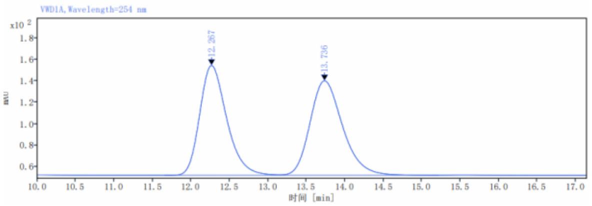

Peak	Retention time/ min	Area%
1	12. 267	49. 06
2	13. 736	50. 94

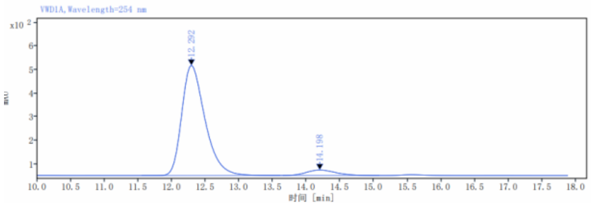

Peak	Retention time/ min	Area%
1	12. 292	94. 22
2	14. 198	5. 78

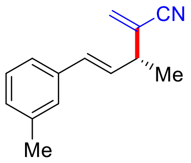

(E)-3-Methyl-2-methylene-5-(m-tolyl)pent-4-enenitrile (4aa)

Yellow oil, 57% yield (11.2 mg), $[\alpha]_D^{25}$ +31.1 (c 0.38, $CHCl_3$) for 89% ee; ^1H NMR (400 MHz, chloroform-d) δ 7.24 – 7.15 (m, 3H), 7.07 (d, J = 7.2 Hz, 1H), 6.47 (d, J = 15.9 Hz, 1H), 6.11 (dd, J = 15.9, 7.4 Hz, 1H), 5.89 (s, 1H), 5.80 (s, 1H), 3.29 – 3.18 (m, 1H), 2.35 (s, 3H), 1.37 (d, J = 6.9 Hz, 3H). ^{13}C NMR (101 MHz, chloroform-d) δ 138.3, 136.6, 131.7, 129.5, 129.2, 128.6, 128.6, 127.6, 127.1, 123.6, 118.1, 41.7, 21.4, 19.0. HRMS (ESI): $[M+Na]^{\oplus}$ calcd for $C_{14}H_{15}NNa^{\oplus}$ 220.1097, found 220.1095. HPLC analysis: Chiracel OJ-H column; detected at 254 nm, 25 °C; ^{i}PrOH : n-hexane = 10 : 90; flow = 0.7 mL/min; Retention time: 11.6 min (minor), 12.7 min (major).

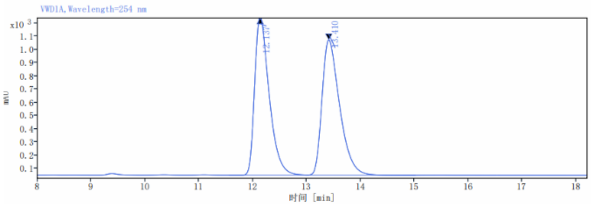

Peak	Retention time/ min	Area%
1	12. 137	49. 17
2	13. 410	50. 83

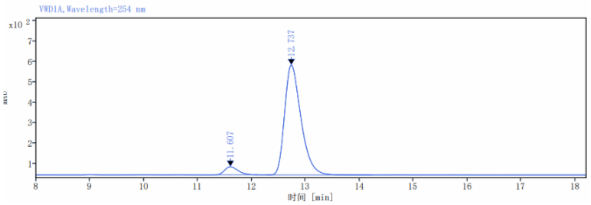

Peak	Retention time/ min	Area%
1	11. 607	5. 67
2	12. 737	94. 33

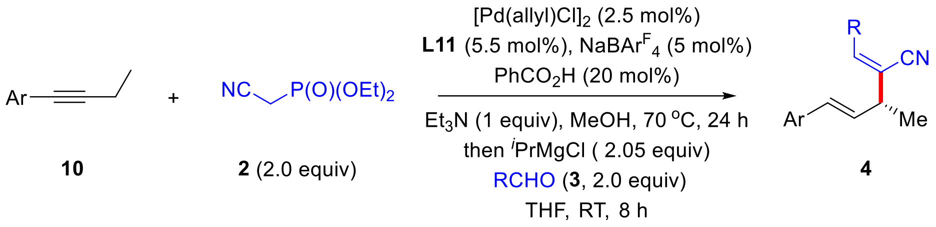

General procedure: In a N_2-filled glovebox, $[Pd(allyl)Cl]_2$ (0.90 mg, 0.0025 mmol), **L11** (3.1 mg, 0.0055 mmol), $NaBAr^F_4$ (4.4 mg, 0.0050 mmol), benzoic acid (2.4 mg, 0.20 mmol) and MeOH (0.20 mL) were added sequentially to a 4 mL vial. The resulting yellow solution was allowed to stir at room temperature for 1 min. Then alkyne **10** (0.10 mmol, 1.0 equiv), phosphonate **2** (35 mg, 0.20 mmol) and Et_3N (14 µL, 0.10 mmol) were added sequentially to the reaction. The resulting solution continued to stir at 70 °C for 24 h. After this time, the reaction solvent was removed. Dry THF (0.20 mL) and i-PrMgCl (1.0 M in THF, 0.205 mL) were added to the reaction sequentially. The resulting solution was allowed to stir at room temperature for 30 min. Then aldehyde **3** (0.20 mmol) was added to the reaction and the resulting mixture continued to stir for additional 8 h at room temperature. After this time, the reaction was quenched with saturated NH_4Cl aqueous solution (3 mL), extracted with CH_2Cl_2 (5 mL × 3), dried over Na_2SO_4, filtered, concentrated and purified by flash silica gel chromatography to give the pure product **4**.

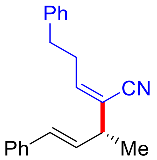

(*Z*)-5-Phenyl-2-((*E*)-4-phenylbut-3-en-2-yl)pent-2-enenitrile (4ab)

Colorless oil, 68% yield (19.6 mg), $[\alpha]_D^{25}$ +26.2 (*c* 0.78, $CHCl_3$) for 90% ee; ^{1}H NMR (400 MHz, chloroform-*d*) δ 7.38 – 7.15 (m, 10H), 6.40 (d, *J* = 15.9 Hz, 1H), 6.22 (t, *J* = 7.4 Hz, 1H), 6.08 (dd, *J* = 15.9, 7.2 Hz, 1H), 3.15 (p, *J* = 7.1 Hz, 1H), 2.78 (dd, *J* = 11.3, 4.5 Hz, 2H), 2.70 (q, *J* = 8.6, 7.7 Hz, 2H), 1.31 (d, *J* = 6.9 Hz, 3H). ^{13}C NMR (101 MHz, chloroform-*d*) δ 145.4, 140.2, 136.8, 130.9, 130.6, 128.6, 128.6, 128.5, 127.7, 126.4, 126.4, 120.0, 116.8, 41.4, 34.9, 33.1, 19.3. HRMS (ESI): $[M+Na]^{\oplus}$ calcd for $C_{21}H_{21}NNa^{\oplus}$ 310.1566, found 310.1563. HPLC analysis: Chiracel OD-H column; detected at 254 nm, 25 °C; ^{i}PrOH : *n*-hexane = 2 : 98; flow = 0.7 mL/min; Retention time: 16.7 min (minor), 17.5 min (major).

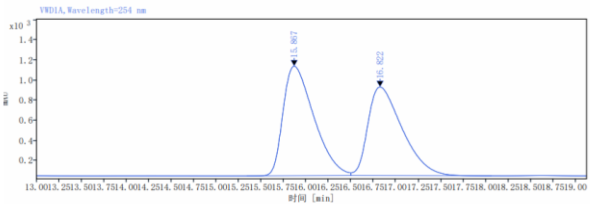

Peak	Retention time/ min	Area%
1	15. 867	52. 63
2	16. 822	47. 37

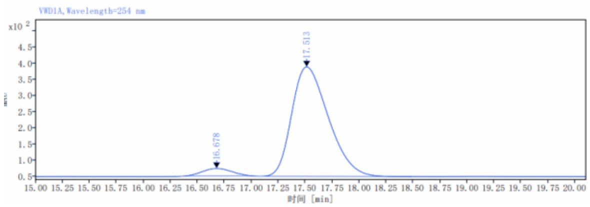

Peak	Retention time/ min	Area%
1	16. 678	5. 10
2	17. 513	94. 90

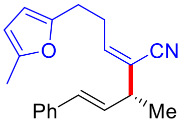

(*Z*)-5-(5-Methylfuran-2-yl)-2-((*E*)-4-phenylbut-3-en-2-yl)pent-2-enenitrile (4ac)

Yellow oil, 63% yield (18.2 mg), $[\alpha]_D^{25}$ +30.1 (*c* 0.73, $CHCl_3$) for 90% ee; ^{1}H NMR (400 MHz, chloroform-*d*) δ 7.41 – 7.31 (m, 4H), 7.29 – 7.24 (m, 1H), 6.45 (d, *J* = 15.9 Hz, 1H), 6.26 (t, *J* = 6.9 Hz, 1H), 6.14 (dd, *J* = 15.9, 7.2 Hz, 1H), 5.92 – 5.86 (m, 2H), 3.26 – 3.15 (m, 1H), 2.76 (t, *J* = 4.8 Hz, 4H), 2.27 (d, *J* = 7.2 Hz, 3H), 1.36 (d, *J* = 6.9 Hz, 3H). ^{13}C NMR (101 MHz, chloroform-*d*) δ 152.1, 151.0, 145.2, 136.9, 130.9, 130.6, 128.6, 127.7, 126.4, 120.1, 116.8, 106.5, 106.0, 41.4, 30.2, 27.1, 19.4, 13.6. HRMS (ESI): $[M+Na]^{\oplus}$ calcd for $C_{20}H_{21}ONNa^{\oplus}$ 314.1515 found, 314.1513. HPLC analysis: Chiracel OJ-H column; detected at 254 nm, 25 °C; ^{i}PrOH : *n*-hexane = 5 : 95; flow = 1.0 mL/min; Retention time: 12.1 min (minor), 12.6 min (major).

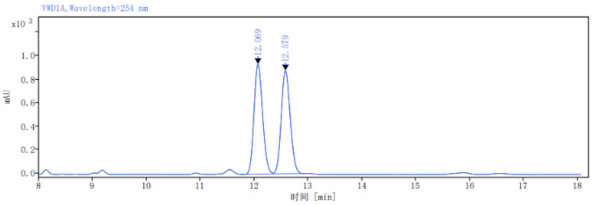

Peak	Retention time/ min	Area%
1	12. 069	50. 39
2	12. 579	49. 61

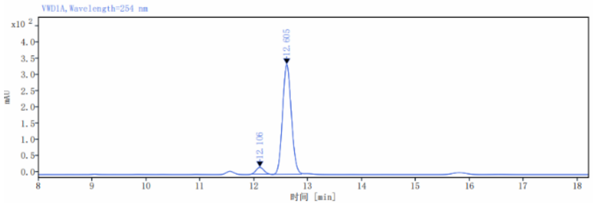

Peak	Retention time/ min	Area%
1	12. 106	5. 09
2	12. 605	94. 91

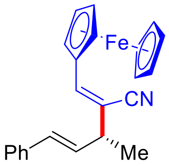

(*E*)-2-((*Z*)-Ferrocenylidene)-3-methyl-5-phenylpent-4-enenitrile (**4ad**)

Red solid, 63% yield (23.5 mg), $[\alpha]_D^{25}$ +103.0 (*c* 0.025, $CHCl_3$) for 90% ee; ^{1}H NMR (400 MHz, chloroform-*d*) δ 7.44 (d, *J* = 7.1 Hz, 2H), 7.36 (t, *J* = 7.5 Hz, 2H), 7.31 – 7.28 (m, 1H), 6.83 (s, 1H), 6.54 (d, *J* = 15.9 Hz, 1H), 6.25 (dd, *J* = 15.9, 7.1 Hz, 1H), 4.85 (dq, *J* = 14.2, 1.8 Hz, 2H), 4.45 (t, *J* = 1.9 Hz, 2H), 4.21 (s, 5H), 3.28 (p, *J* = 7.0 Hz, 1H), 1.45 (d, *J* = 7.0 Hz, 3H). ^{13}C NMR (101 MHz, chloroform-*d*) δ 143.1, 137.0, 131.1, 130.7, 128.7, 127.7, 126.5, 119.3, 110.9, 70.9, 70.8, 69.8, 69.7, 69.6, 42.5, 19.6. HRMS (ESI): $[M]^{\oplus}$ calcd for $C_{23}H_{21}FeN^{\oplus}$ 367.1018 found, 367.1012. HPLC analysis: Chiracel AD-H column; detected at 254 nm, 25 °C; ^{i}PrOH : *n*-hexane = 10 : 90; flow = 1.0 mL/min; Retention time: 7.3 min (major), 8.1 min (minor).

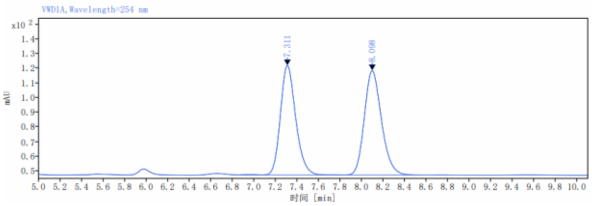

Peak	Retention time/ min	Area%
1	7.311	48.41
2	8.098	51.59

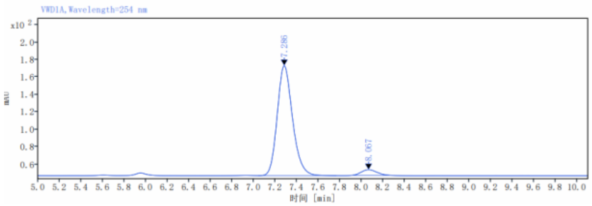

Peak	Retention time/ min	Area%
1	7.286	94.94
2	8.067	5.06

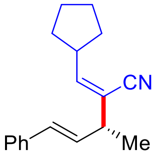

(2*Z*,4*E*)-2-(Cyclopentylmethylene)-3-methyl-5-phenylpent-4-enenitrile (**4ae**)

Yellow oil, 60% yield (15.1 mg), $[\alpha]_D^{25}$ +41.8 (*c* 0.50, $CHCl_3$) for 90% ee; ^{1}H NMR (400 MHz, chloroform-*d*) δ 7.43 – 7.28 (m, 4H), 7.27 – 7.20 (m, 1H), 6.45 (d, *J* = 15.7 Hz, 1H), 6.21 – 6.07 (m, 2H), 3.16 (p, *J* = 7.1 Hz, 1H), 2.96 (dt, *J* = 17.3, 8.6 Hz, 1H), 1.98 – 1.88 (m, 2H), 1.68 (ddd, *J* = 27.0, 8.1, 4.2 Hz, 4H), 1.35 (d, *J* = 6.9 Hz, 3H), 1.32 – 1.23 (m, 2H). ^{13}C NMR (101 MHz, chloroform-*d*) δ 151.7, 136.9, 130.9, 130.8, 128.6, 127.6, 126.4, 117.3, 117.2, 42.4, 41.3, 33.2, 25.6, 19.5. HRMS (ESI): $[M+Na]^{\oplus}$ calcd for $C_{18}H_{21}NNa^{\oplus}$ 274.1566, found 274.1563. HPLC analysis: Chiracel OJ-H column; detected at 254 nm, 25 °C; ^{i}PrOH : *n*-hexane = 5 : 95; flow = 1.0 mL/min; Retention time: 12.3 min (major), 13.0 min (minor).

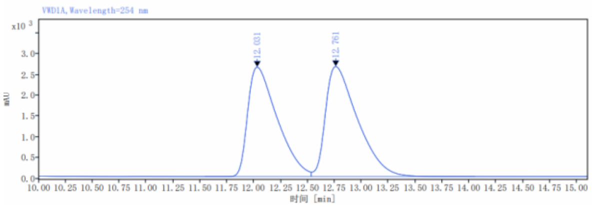

Peak	Retention time/ min	Area%
1	12.031	47.74
2	12.761	52.26

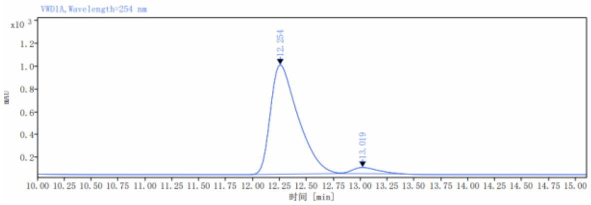

Peak	Retention time/ min	Area%
1	12.254	94.94
2	13.019	5.06

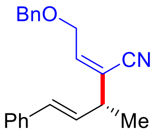

(2*Z*,4*E*)-2-(2-(Benzyloxy)ethylidene)-3-methyl-5-phenylpent-4-enenitrile (4af)

Yellow oil, 60% yield (18.1 mg), $[\alpha]_D^{25}$ +27.7 (*c* 0.07, $CHCl_3$) for 90% ee; ^{1}H NMR (400 MHz, chloroform-*d*) δ 7.34 (dq, *J* = 14.9, 7.4 Hz, 9H), 7.27 – 7.22 (m, 1H), 6.47 (d, *J* = 15.9 Hz, 1H), 6.37 (t, *J* = 6.4 Hz, 1H), 6.11 (dd, *J* = 15.9, 7.4 Hz, 1H), 4.55 (s, 2H), 4.34 (d, *J* = 6.4 Hz, 2H), 3.22 (t, *J* = 7.1 Hz, 1H), 1.36 (d, *J* = 6.9 Hz, 3H). ^{13}C NMR (101 MHz, chloroform-*d*) δ 142.2, 137.5, 136.7, 131.5, 129.8, 128.7, 128.6, 128.1, 128.0, 127.8, 126.5, 121.0, 116.1, 73.3, 68.5, 41.5, 19.1. HRMS (ESI): $[M+H]^{\oplus}$ calcd for $C_{21}H_{22}ON^{\oplus}$ 304.1696, found 304.1695. HPLC analysis: Chiracel OD-H column; detected at 254 nm, 25 °C; ^{i}PrOH : *n*-hexane = 15 : 85; flow = 1.0 mL/min; Retention time: 8.8 min (minor), 10.3 min (major).

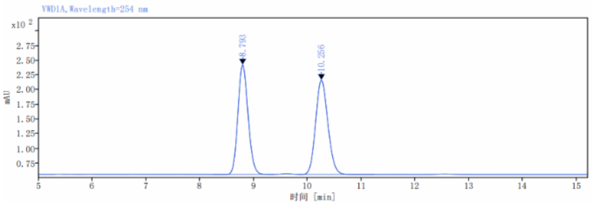

Peak	Retention time/ min	Area%
1	8. 793	49. 09
2	10. 256	50. 91

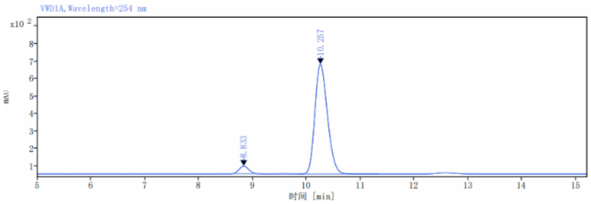

Peak	Retention time/ min	Area%
1	8. 833	5. 11
2	10. 257	94. 89

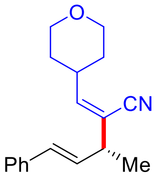

(2*Z*,4*E*)-3-Methyl-5-phenyl-2-((tetrahydro-2H-pyran-4-yl)methylene)pent-4-enenitrile (4ag)

Yellow oil, 60% yield (16.6 mg), $[\alpha]_D^{25}$ +25.7 (*c* 0.68, $CHCl_3$) for 90% ee; ^{1}H NMR (400 MHz, chloroform-*d*) δ 7.41 (d, *J* = 7.1 Hz, 2H), 7.35 (t, *J* = 7.5 Hz, 2H), 7.29 (d, *J* = 6.9 Hz, 1H), 6.49 (d, *J* = 15.9 Hz, 1H), 6.16 (dd, *J* = 15.9, 7.3 Hz, 1H), 6.08 (d, *J* = 9.6 Hz, 1H), 4.05 – 3.98 (m, 2H), 3.50 (td, *J* = 11.7, 2.2 Hz, 2H), 3.21 (p, *J* = 7.0 Hz, 1H), 2.90 – 2.79 (m, 1H), 1.65 (d, *J* = 7.0 Hz, 2H), 1.62 – 1.51 (m, 2H), 1.39 (d, *J* = 6.9 Hz, 3H). ^{13}C NMR (101 MHz, chloroform-*d*) δ 149.6, 136.7, 131.1, 130.4, 128.7, 127.8, 126.4, 118.4, 116.8, 67.1, 41.3, 38.0, 31.7, 19.4. HRMS (EI): $[M]^{\oplus}$ calcd for $C_{18}H_{21}ON^{\oplus}$ 267.1618, found 267.1621. HPLC analysis: Chiracel AD-H column; detected at 254 nm, 25 °C; ^{i}PrOH : *n*-hexane = 5 : 95; flow = 1.0 mL/min; Retention time: 6.7 min (minor), 7.0 min (major).

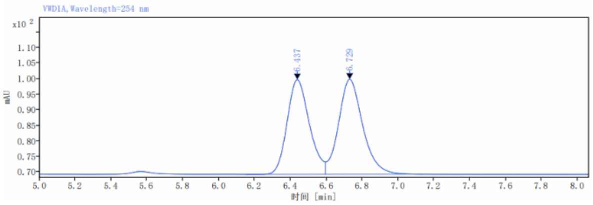

Peak	Retention time/ min	Area%
1	6. 437	47. 49
2	6. 729	52. 21

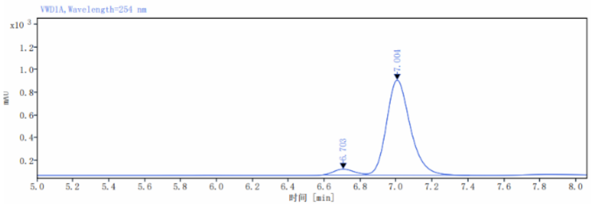

Peak	Retention time/ min	Area%
1	6. 703	5. 02
2.	7. 004	94. 98

8. Preliminary mechanistic studies

8.1 Reaction analysis

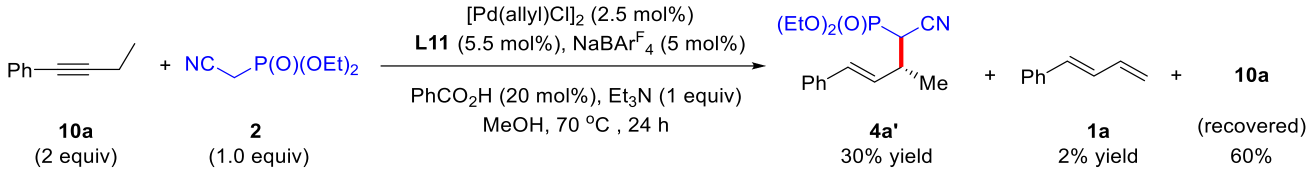

In a N_2-filled glovebox, $[Pd(allyl)Cl]_2$ (0.9 mg, 0.0050 mmol), **L11** (3.1 mg, 0.0055 mmol), sodium tetrakis[3,5-bis(trifluoromethyl)phenyl]borate ($NaBAr^F_4$, 4.4 mg, 0.0050 mmol), benzoic acid (2.4 mg, 0.20 mmol) and dry MeOH (0.2 mL) were added to a 4 mL vial sequentially. The resulting mixture was stirred at room temperature for 5 min. Then Et_3N (14 μL, 0.10 mmol), 1-Phenyl-1-butyne **10a** (26 mg, 0.20 mmol) and phosphonate **2** (18 mg, 0.10 mmol) were added to the reaction sequentially. The resulting mixture continued to stir at 70 °C for 24 h. After this time, the crude mixture was cooled to room temperature, condensed and analyzed by crude ^{1}H NMR with CH_2Br_2 (14 μL, 0.2 mmol) as internal standard.

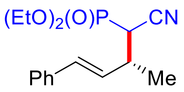

Diethyl ((1*R*,*E*)-1-cyano-2-methyl-4-phenylbut-3-en-1-yl)phosphonate (4a')

Colorless oil. 2:1 dr. ^{1}H NMR (400 MHz, chloroform-*d*) δ 7.51 – 7.22 (m, 5H), 6.69 – 6.50 (m, 1H), 6.41 – 6.12 (m, 1H), 4.36 – 4.09 (m, 4H), 3.21 – 2.96 (m, 2H), 1.47 – 1.23 (m, 9H). ^{13}C NMR (101 MHz, chloroform-*d*) δ 136.6, 136.4, 132.1, 131.1, 130.8, 130.7, 128.9, 128.6, 128.6, 127.8, 127.8, 126.4, 126.4, 64.1, 64.1, 64.0, 63.7, 63.6, 63.5, 63.4, 37.8, 37.2, 36.4, 36.0, 35.83, 35.78, 20.7, 20.6, 17.8, 17.7, 16.4, 16.34, 16.31, 16.26. ^{31}P NMR (162 MHz, chloroform-*d*) δ 30.32 (s, 1P). HRMS (ESI): $[M+Na]^{\oplus}$ calcd for $C_{16}H_{23}O_3NPNa^{\oplus}$ 308.1410, found 308.1408.

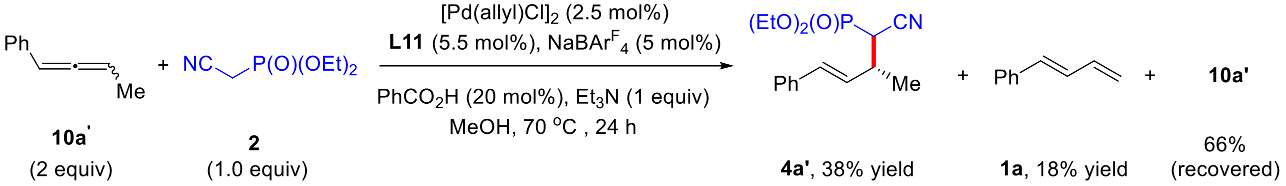

The procedure was same to that with **10a** as the substrate described above, except that **10a'** was used as the reactant. In this context, **4a'** was observed in 38% yield and **1a** was observed in 18% yield along with 66% of **10a'** recovered.

8.2 Compound 11 as substrate

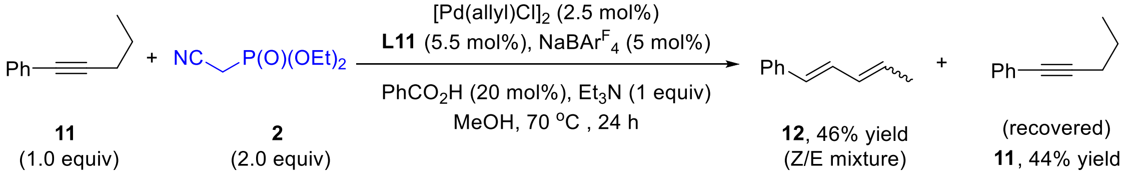

In a N_2-filled glovebox, $[Pd(allyl)Cl]_2$ (0.9 mg, 0.0050 mmol), **L11** (3.1 mg, 0.0055 mmol), sodium tetrakis[3,5-bis(trifluoromethyl)phenyl]borate ($NaBAr^F_4$, 4.4 mg, 0.0050 mmol), benzoic acid (2.4 mg, 0.20 mmol) and dry MeOH (0.2 mL) were added to a 4 mL vial sequentially. The resulting mixture was stirred at room temperature for 5 min. Then Et_3N (14 μL, 0.10 mmol), 1-Phenyl-1-pentyne **11** (14 mg, 0.10 mmol) and phosphonate **2** (35 mg, 0.20 mmol) were added to the reaction sequentially. The resulting mixture continued to stir at 70 °C for 24 h. After this time, the reaction was cooled to room temperature, condensed and analyzed by crude ^1H NMR with CH_2Br_2 (14 μL, 0.2 mmol) as internal standard.

8.3 Diene 1a as substrate

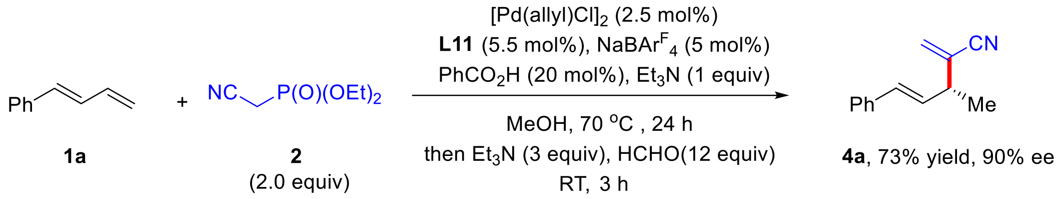

In a N_2-filled glovebox, $[Pd(allyl)Cl]_2$ (0.9mg, 0.0050 mmol), **L11** (3.1mg, 0.0055 mmol), The sodium tetrakis[3,5-bis(trifluoromethyl)phenyl]borate ($NaBAr^F_4$, 4.4 mg, 0.0050 mmol), benzoic acid (2.4 mg, 0.20 mmol) and dry MeOH (0.2 mL) were added to a 4 mL vial sequentially. The resulting mixture was stirred at room temperature for 5 min. Then Et_3N (14 μL, 0.10 mmol), 1-Phenyl-1-pentyne **1a** (13 mg, 0.10 mmol) and diethyl (cyanomethyl) phosphonate **2** (35 mg, 0.20 mmol) were added to the reaction sequentially. The resulting solution continued to stir at 70 °C for 24 h. After this time, Et_3N (42 μL, 0.30 mmol) and HCHO aqueous solution (0.10 mL, 37 wt.%, 1.20 mmol) were added to the reaction. The resulting mixture continued to stir at room temperature for 3 h. After this time, the reaction was condensed and purified by flash column chromatography (*n*-hexane/ethyl acetate = 20/1) to give **4a** in 73% yield and 90% ee.

8.4 Deuterium experiments

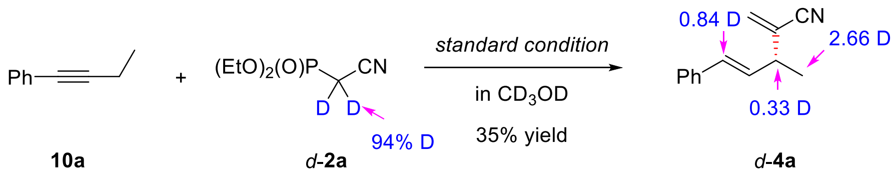

The reaction procedure was the same as that described in part 7 titled “General procedure for formal hydroalkenylation of alkynes”, with the changes that *d*-**2a** as the nucleophile and CD_3OD as the solvent for the first step were adopted.

^1H NMR of *d*-**4d**

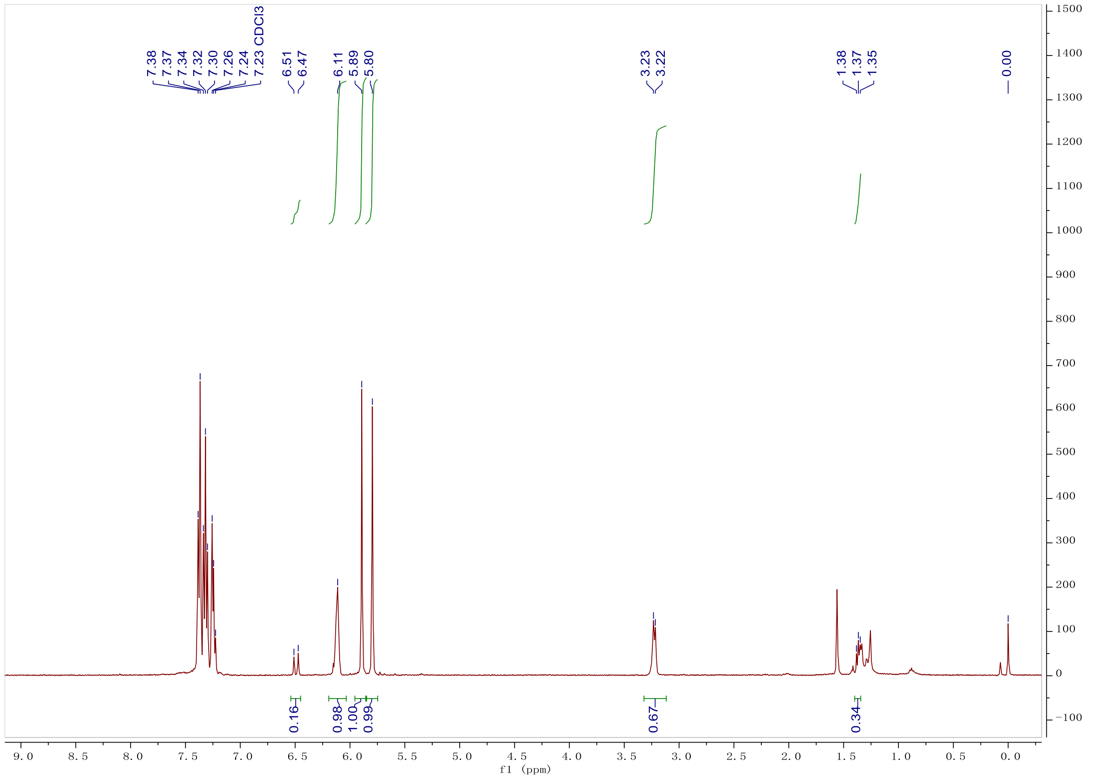
7.38
7.37
7.34
7.32
7.30
7.26
7.24
7.23 CDCl3
6.51
6.47
6.11
5.89
5.80
3.23
3.22
1.38
1.37
1.35
0.00
0.16
0.98
1.00
0.99
0.67
0.34
f1 (ppm)

^{2}H NMR of *d*-**4d**

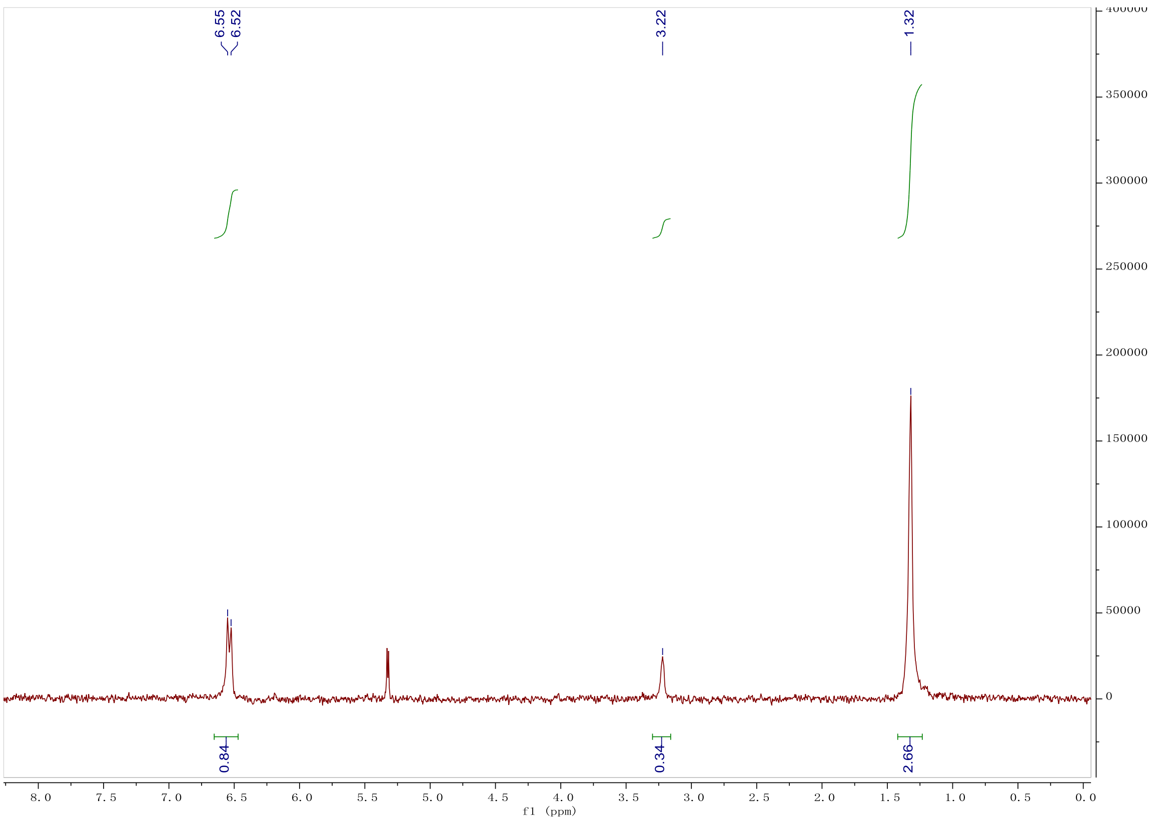
6.55
6.52
3.22
1.32
0.84
0.34
2.66
f1 (ppm)

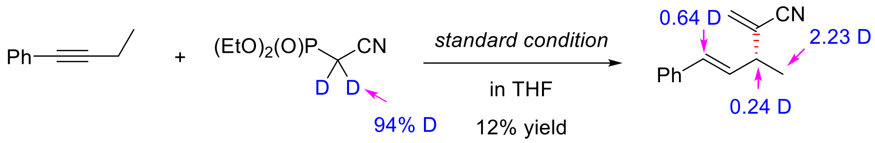

10a *d*-**2a** *d*-**4a**

The reaction procedure was the same as that described in part 7 titled "General procedure for formal hydroalkenylation of alkynes", with the changes that *d*-**2a** as the nucleophile and THF as the solvent for the first step were adopted.

^{1}H NMR of *d*-**4d**

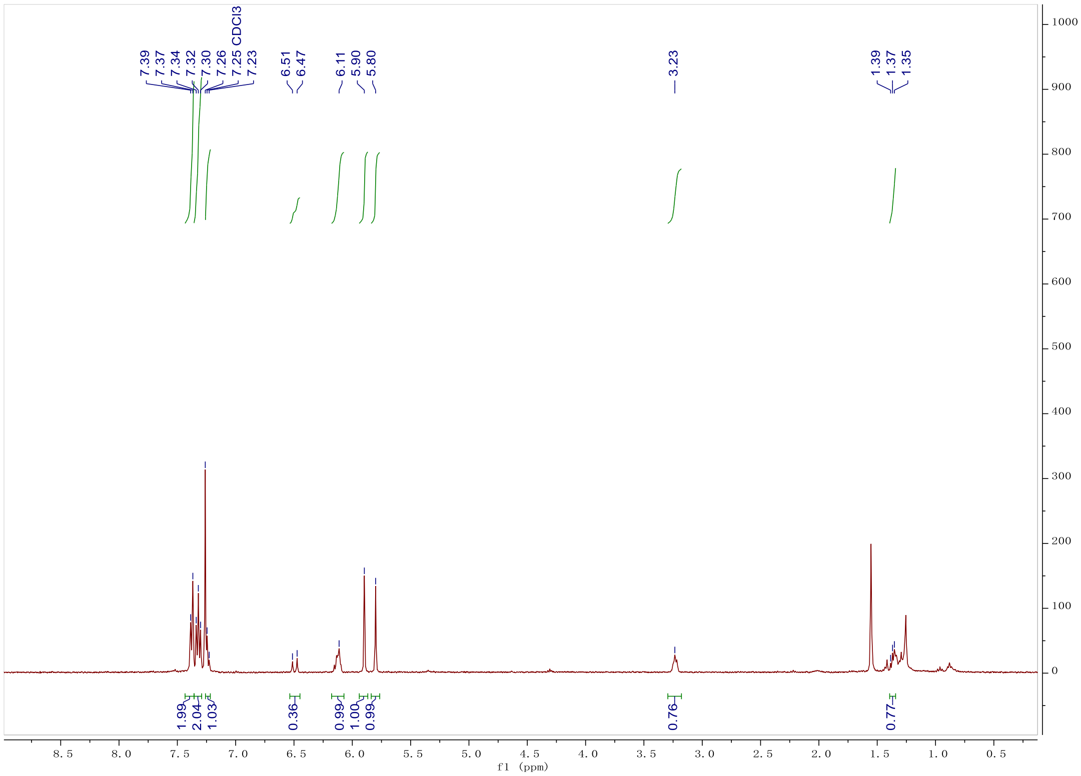

^{2}H NMR of *d*-**4d**

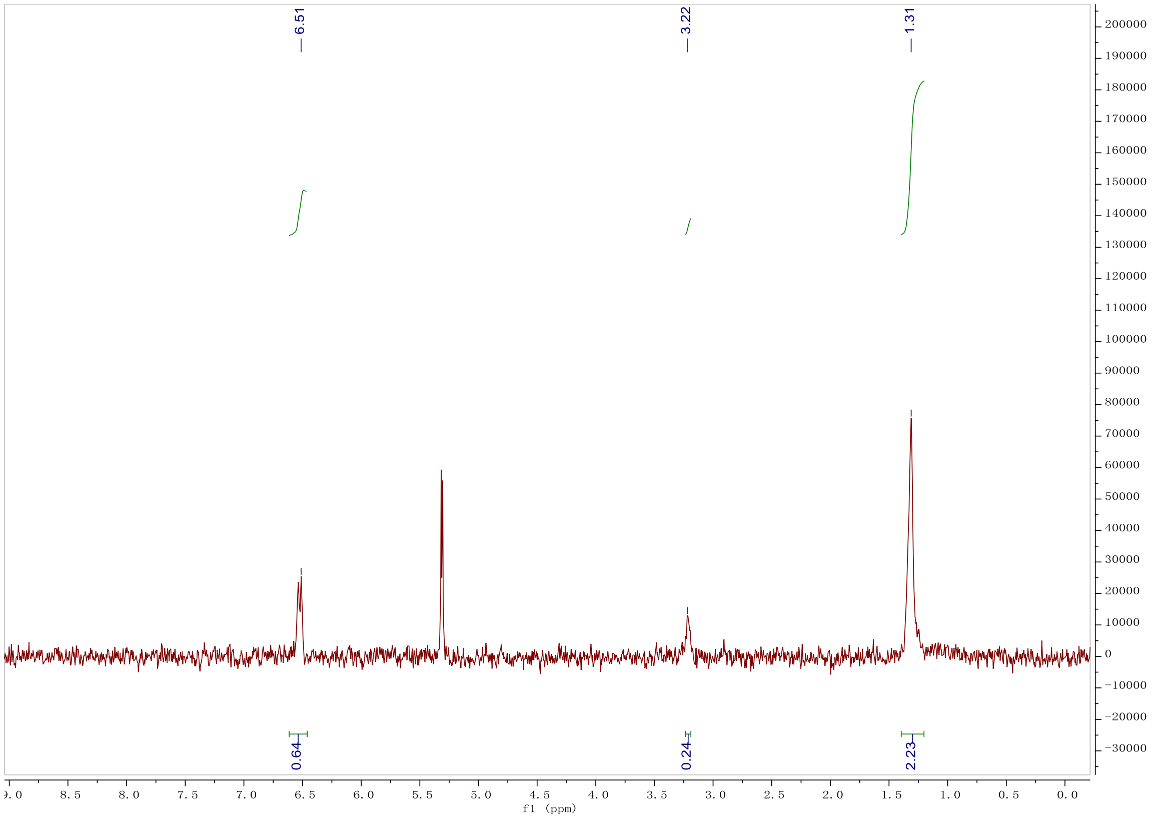

8.5 Internal alkyl alkyne 13 as the substrate

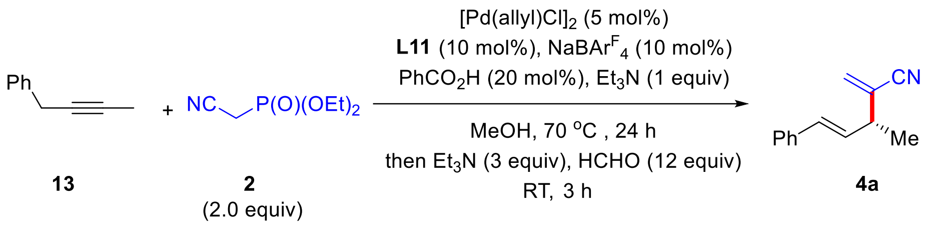

In the N_2-filled glovebox, $[Pd(allyl)Cl]_2$ (1.8 mg, 0.0050 mmol), **L11** (5.6 mg, 0.010 mmol), sodium tetrakis[3,5-bis(trifluoromethyl)phenyl]borate ($NaBAr^F{}_4$, 8.8 mg, 0.010 mmol), benzoic acid (2.4 mg, 0.20 mmol) and dry MeOH (0.2 mL) were added to a 4 mL vial sequentially. The resulting mixture was stirred at room temperature for 5 min. Then Et_3N (14 μL, 0.10 mmol), 2-butyn-1-ylbenzene **13** (13 mg, 0.10 mmol) and diethyl (cyanomethyl) phosphonate **2** (35 mg, 0.20 mmol) were added to the reaction sequentially. The resulting reaction continued to stir at 70 °C for 24 h. After this time, to the reaction were added Et_3N (42 μL, 0.30 mmol) and HCHO aqueous solution (0.10 mL, 37 wt.%, 1.2 mmol). Then the resulting mixture continued to stir at room temperature for 3 h. After this time, the reaction was condensed and purified by flash column chromatography (*n*-hexane/ethyl acetate = 20/1) to give **4a** in 55% yield and 90% ee.

8.6 The elucidation of isomerization between (*Z*)- and (*E*)-diene

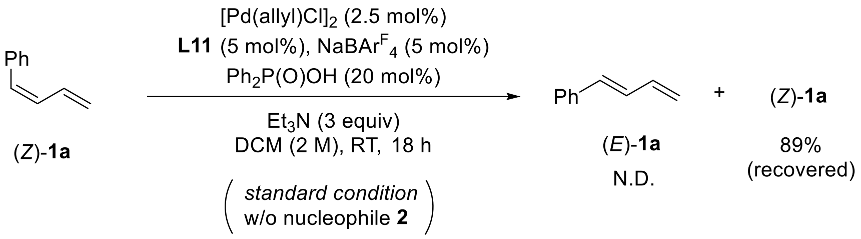

In a N_2-filled glovebox, $[Pd(allyl)Cl]_2$ (0.9 mg, 0.0025 mmol), **L11** (2.8 mg, 0.0050 mmol), $NaBAr^F{}_4$ (4.4 mg, 0.0050 mmol,), $Ph_2P(O)OH$ (4.4 mg, 0.020 mmol) and DCM (0.050 mL) were added sequentially to a 4 mL vial. The resulting yellow solution was allowed to stir at ambient temperature for 1 min. Then diene (*Z*)-**1a** (13 mg, 0.10 mmol), and Et_3N (42 µL, 0.30 mmol) were added sequentially to the reaction. The reaction mixture continued to stir at room temperature for 18 h. After this time, the reaction was extracted with CH_2Cl_2 (2 mL × 3), dried over Na_2SO_4, filtered, concentrated, purified by flash silica gel chromatography and analyzed by ^1H NMR. (*Z*)-**1a** was recovered in 89% yield with no detection of (*E*)-**1a**.

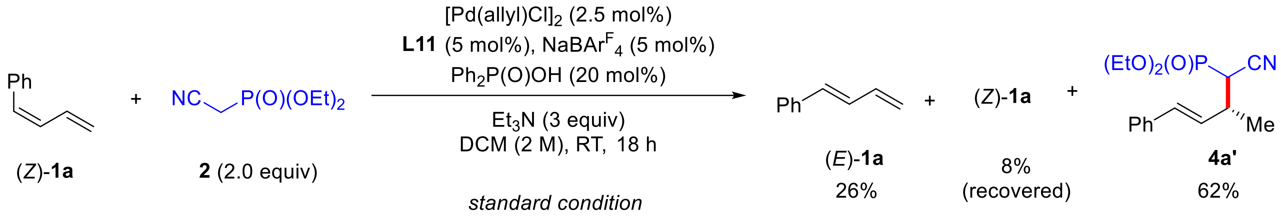

In a N_2-filled glovebox, $[Pd(allyl)Cl]_2$ (0.9 mg, 0.0025 mmol), **L11** (2.8 mg, 0.0050 mmol), $NaBAr^F{}_4$ (4.4 mg, 0.0050 mmol,), $Ph_2P(O)OH$ (4.4 mg, 0.020 mmol) and DCM (0.050 mL) were added sequentially to a 4 mL vial. The resulting yellow solution was allowed to stir at ambient temperature for 1 min. Then diene (*Z*)-**1a** (13 mg, 0.10 mmol), diethyl (cyanomethyl)phosphonate **2** (35 mg, 0.20 mmol) and Et_3N (42 µL, 0.30 mmol) were added sequentially to the reaction. The reaction mixture continued to stir at room temperature for 18 h. After this time, the reaction was extracted with CH_2Cl_2 (2 mL × 3), dried over Na_2SO_4, filtered, concentrated, purified by flash silica gel chromatography and analyzed by ^1H NMR. (*Z*)-**1a** was recovered in 8% yield along with (*E*)-**1a** in 26% yield and **4a’** in 62% yield.

9. Convergent and stereodivergent synthesis

9.1 Convergent synthesis

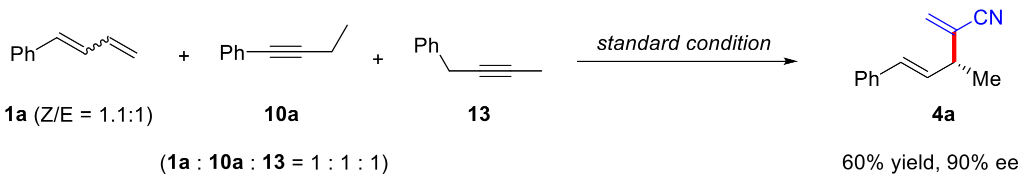

The reaction procedure was the same as that described in part 7 titled “General procedure for formal hydroalkenylation of alkynes”, with the changes that 1-phenyl-1-butyne **10a** (4.3 mg, 0.033 mmol), buta-1,3-dien-1-ylbenzene **1a** (4.3 mg, 0.033 mmol), 2-butyn-1-ylbenzene **13** (4.3 mg, 0.033 mmol) for the first step were adopted.

9.2 Stereodivergent synthesis

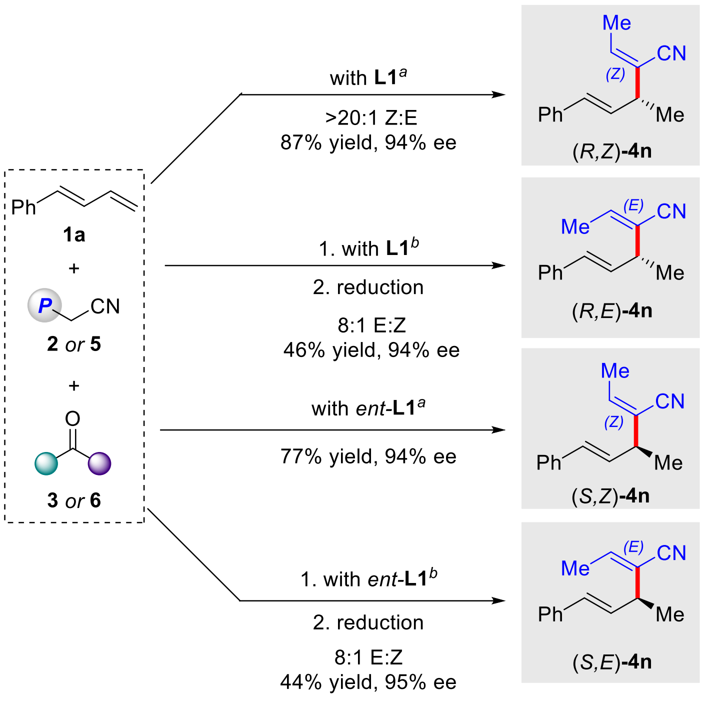

Supplementary Figure 3. Stereodivergent synthesis

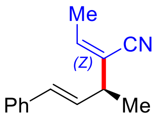

(*S*,*Z*)-**4n**.

The synthetic procedure and corresponding NMR spectra for this compound was the same as that for (*R*,*Z*)-**4n** with the only change that *ent*-**L11** was used instead. Colourless oil, 77% yield (15.2 mg), $[\alpha]_D^{25}$ -40.7 (*c* 0.59, $CHCl_3$) for 94% ee; HPLC analysis: Chiracel (OD-H) + (OD-H) column (the two columns were connected to each other); detected at 254 nm, 20 °C; ^{i}PrOH : *n*-hexane = 3 : 97; flow = 0.7 mL/min; Retention time: 18.3 min (major), 19.0 min (minor).

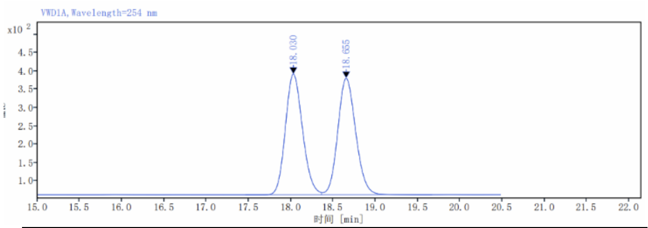

Peak	Retention time/ min	Area%
1	18. 030	49. 72
2	18. 655	50. 28

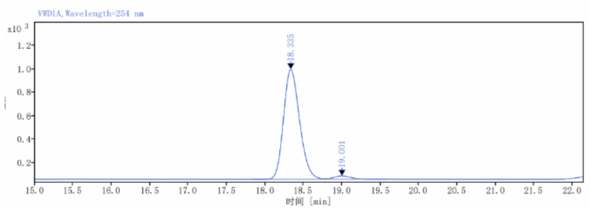

Peak	Retention time/ min	Area%
1	18. 335	96. 94
2	19. 001	3. 06

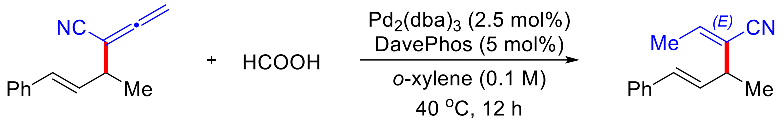

7a or *ent*-**7a** **19** **4n**

In a N_2-filled glovebox, $Pd_2(dba)_3$ (2.3 mg, 0.0025 mmol, 2.5 mol%), DavePhos (2.0 mg, 0.0050 mmol, 5.0 mol%) and *o*-Xylene (1.0 mL) were added sequentially to a 4 mL vial. The resulting solution was allowed to stir at ambient temperature for 1 min. Then **7a** or *ent*-**7a** (24 mg, 0.10 mmol) and formic acid **19** (4.6 mg, 0.10 mmol) was added. The reaction solution was allowed to stir at 40 °C for 12 h. After this time, the solution was concentrated and purified by preparative TLC plate to provide pure (*S*,*E*)-**4n** and (*R*,*E*)-**4n.**

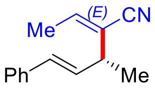

(*R*,*E*)-**4n**

Colorless oil, 46% yield (9.0 mg). $[\alpha]_D^{25}$ +127.9 (*c* 0.40, $CHCl_3$) for 94% ee; ^{1}H NMR (400 MHz, chloroform-*d*) δ 7.36 (d, *J* = 7.5 Hz, 2H), 7.31 (t, *J* = 7.5 Hz, 2H), 7.27 – 7.21 (m, 1H), 6.50 – 6.39 (m, 2H), 6.17 (dd, *J* = 15.9, 7.0 Hz, 1H), 3.58 – 3.43 (m, 1H), 1.88 (d, *J* = 7.1 Hz, 3H), 1.35 (*d*, J = 6.9 Hz, 3H). ^{13}C NMR (126 MHz, chloroform-*d*) δ 142.1, 136.8, 130.4, 130.3, 128.6, 127.7, 126.4, 120.0, 118.7, 35.3, 19.3, 14.4. HRMS (EI): $[M]^{\oplus}$ calcd for $C_{14}H_{15}N^{\oplus}$ 197.1199, found 197.1202. HPLC analysis: Chiracel OJ-H column; detected at 254 nm, 25 °C; ^{i}PrOH : *n*-hexane = 5 : 95; flow = 0.7 mL/min; Retention time: 17.2 min (minor), 21.0 min (major).

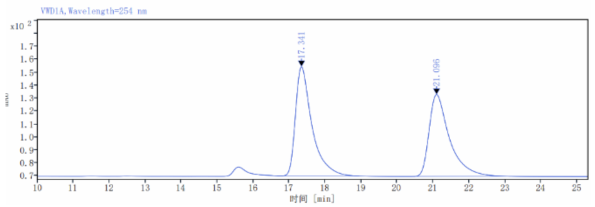

Peak	Retention time/ min	Area%
1	17. 341	51. 83
2	21. 096	48. 17

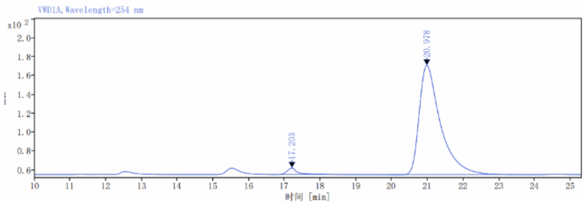

Peak	Retention time/ min	Area%
1	17. 203	3. 05
2	20. 978	96. 95

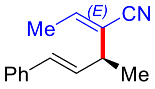

(*S*,*E*)-**4n**

The corresponding NMR spectra for this compound was the same as that for (*R*,*E*)-**4n**. Colorless oil, 42% yield (8.2 mg). $[\alpha]_D^{25}$ -129.3 (*c* 0.32, $CHCl_3$) for 95% ee; HPLC analysis: Chiracel OJ-H column; detected at 254 nm, 25 °C; ^{i}PrOH : *n*-hexane = 5 : 95; flow = 0.7 mL/min; Retention time: 17.1 min (minor), 20.8 min (major).

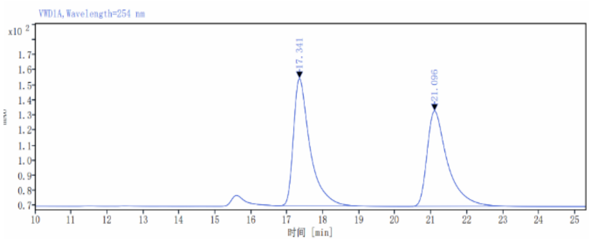

Peak	Retention time/ min	Area%
1	17. 341	51. 83
2	21. 096	48. 17

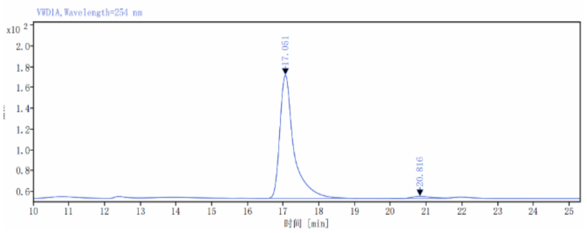

Peak	Retention time/ min	Area%
1	17. 051	97. 71
2	20. 816	2. 29

10. X-ray structure of 4t

The Crystallographic data for the structure **4t** has been deposited at the Cambridge Crystallographic Data Centre, under deposition number CCDC 2251290.

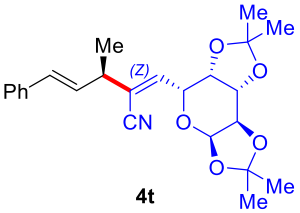

4t

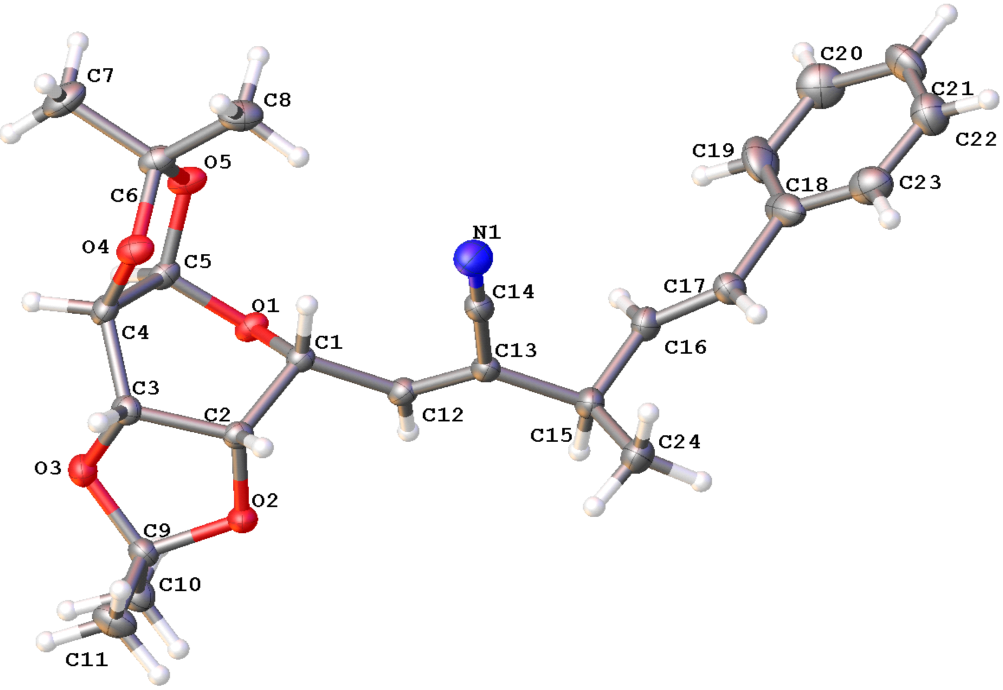

Table 1. Crystal data and structure refinement for **4t**.

Identification code	**4t**
Empirical formula	C24 H29 N O5
Formula weight	411.48
Temperature	213.00 K
Wavelength	1.34139 Å

Crystal system	Monoclinic	
Space group	P 1 21 1	
Unit cell dimensions	a = 6.51240(10) Å	a= 90°.
	b = 7.18510(10) Å	b= 90.4300(10)°.
	c = 23.8510(4) Å	g = 90°.
Volume	1116.01(3) $Å^3$	
Z	2	
Density (calculated)	1.225 Mg/m^3	
Absorption coefficient	0.442 mm^{-1}	
F(000)	440	
Crystal size	0.07 x 0.07 x 0.05 mm^3	
Theta range for data collection	3.224 to 54.858°.	
Index ranges	-7<=h<=7, -8<=k<=7, -28<=l<=28	
Reflections collected	12638	
Independent reflections	4018 [R(int) = 0.0419]	
Completeness to theta = 53.594°	99.4 %	
Absorption correction	Semi-empirical from equivalents	
Max. and min. transmission	0.7508 and 0.6284	
Refinement method	Full-matrix least-squares on F^2	
Data / restraints / parameters	4018 / 67 / 295	
Goodness-of-fit on F^2	1.051	
Final R indices [I>2sigma(I)]	R1 = 0.0305, wR2 = 0.0745	
R indices (all data)	R1 = 0.0322, wR2 = 0.0758	
Absolute structure parameter	0.07(9)	
Extinction coefficient	n/a	
Largest diff. peak and hole	0.118 and -0.172 e.$Å^{-3}$	

11. Copies of ^{1}H NMR, ^{13}C NMR, ^{19}F NMR and ^{31}P NMR spectra

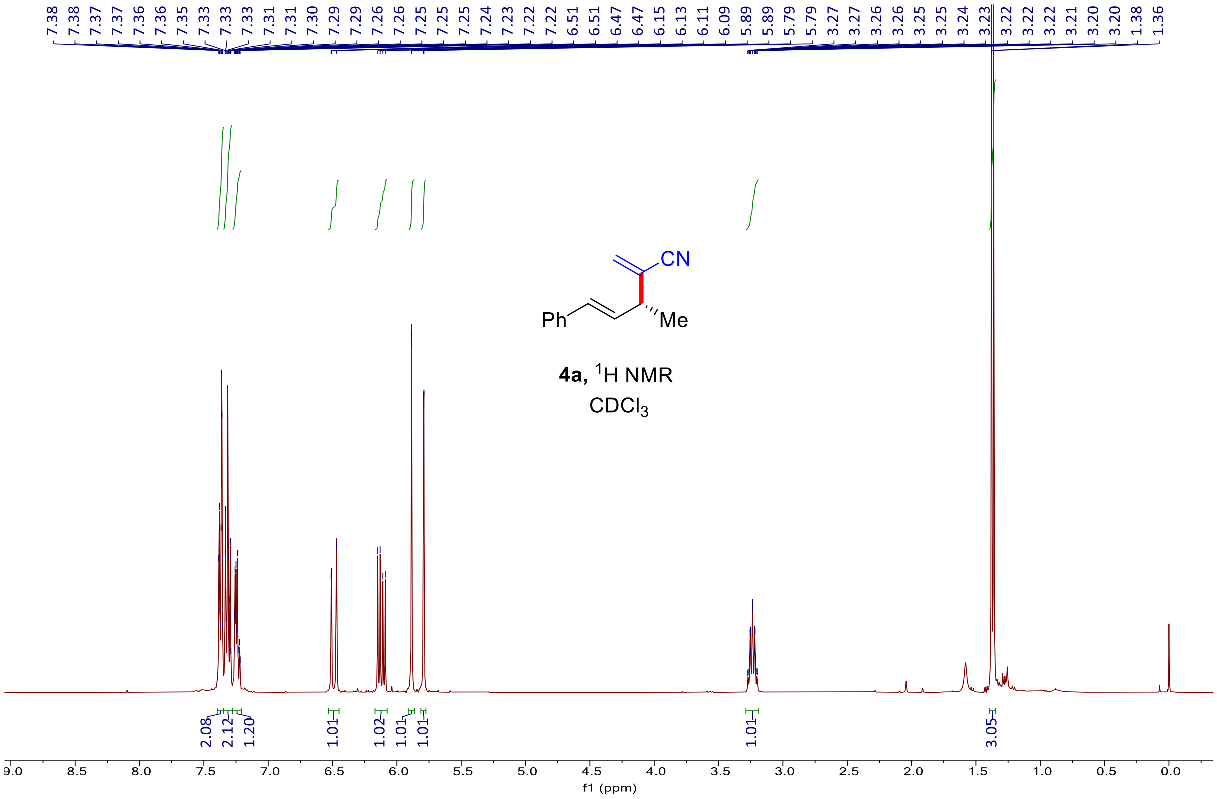

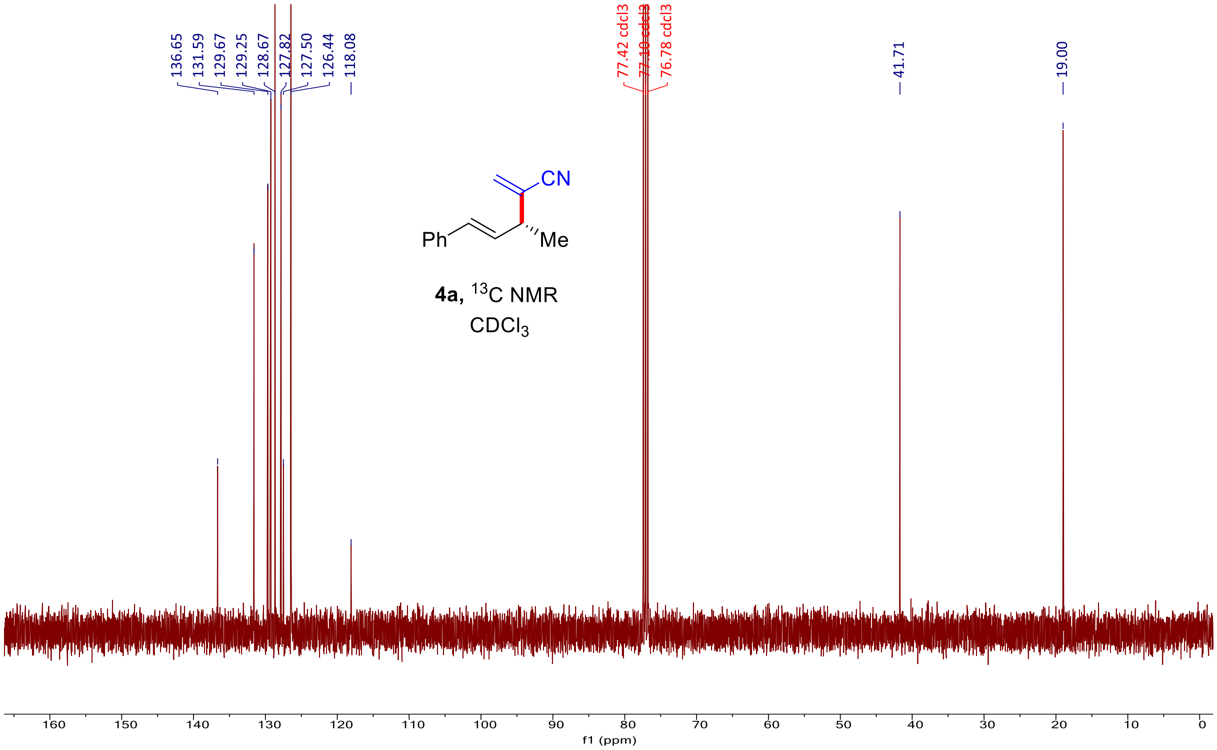

Supplementary Figure 4. ^{1}H NMR and ^{13}C NMR spectra of compound **4a**

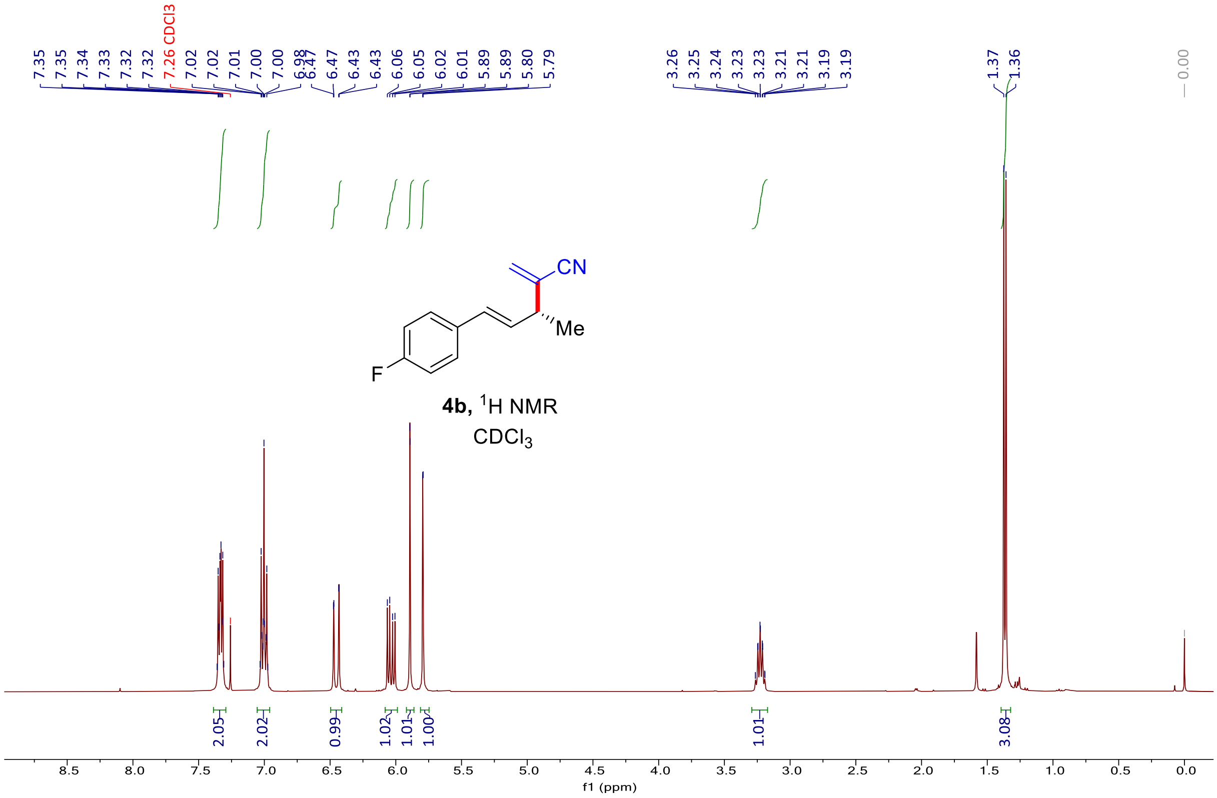

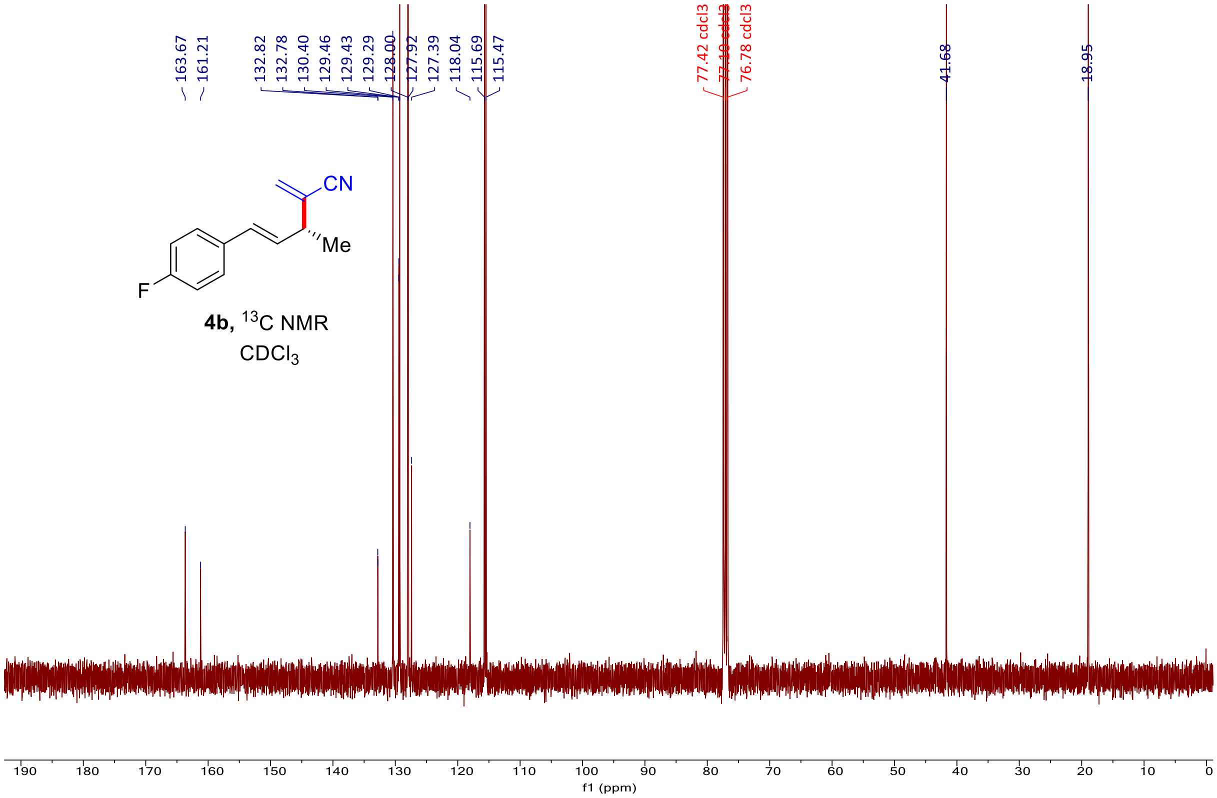

Supplementary Figure 5. ^{1}H NMR and ^{13}C NMR spectra of compound **4b**

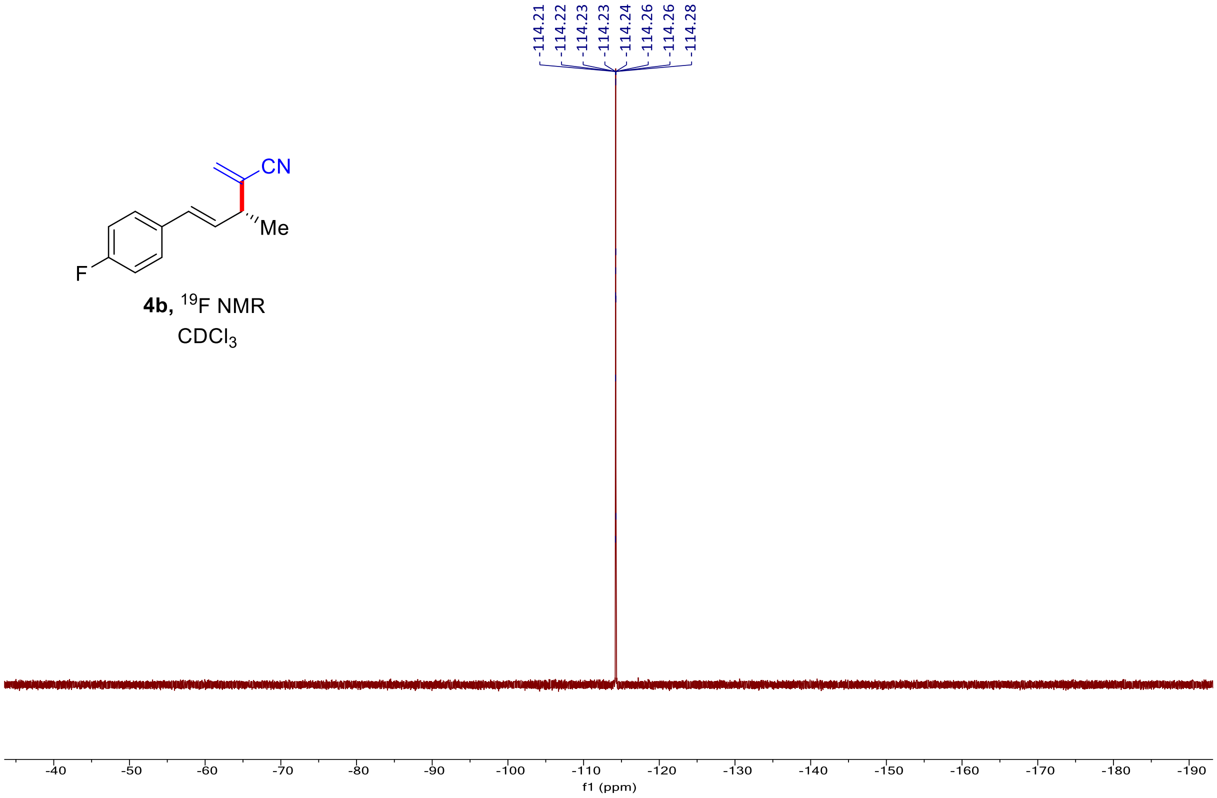

Supplementary Figure 6. ^{19}F NMR spectra of compound **4a**

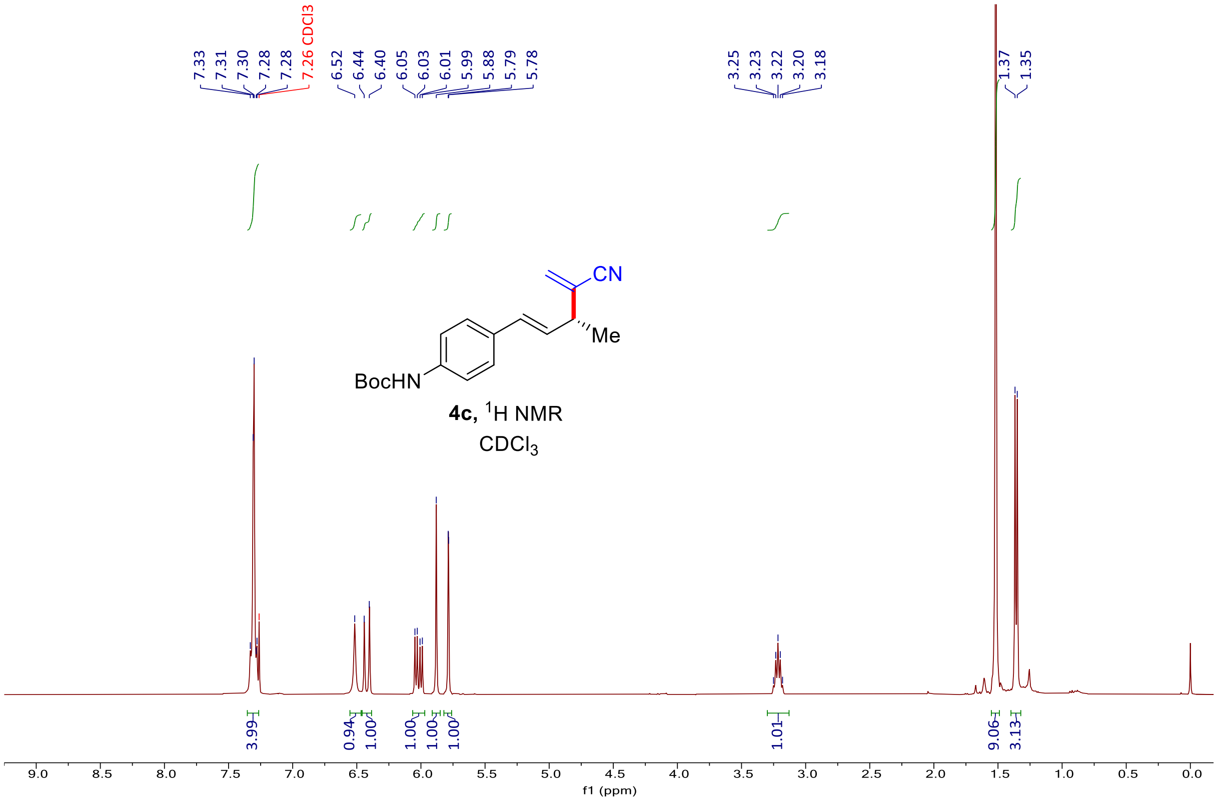

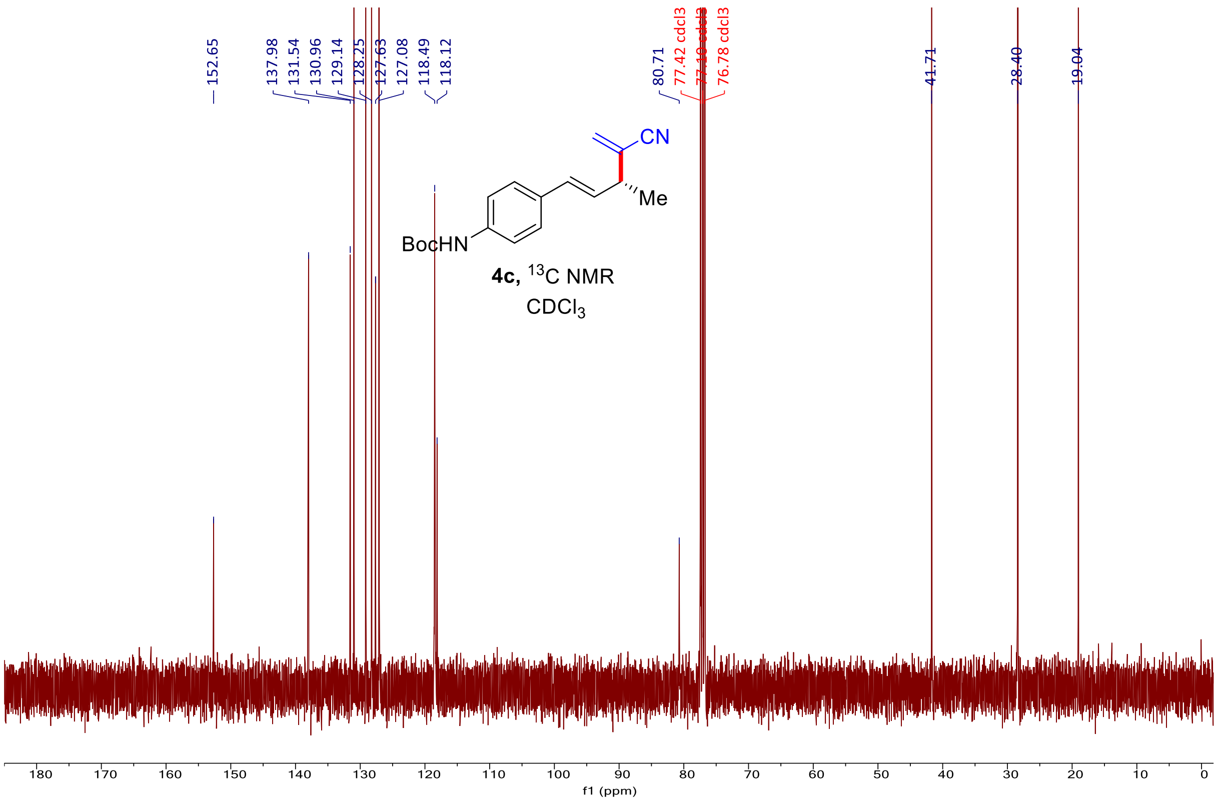

Supplementary Figure 7. ^{1}H NMR and ^{13}C NMR spectra of compound **4c**

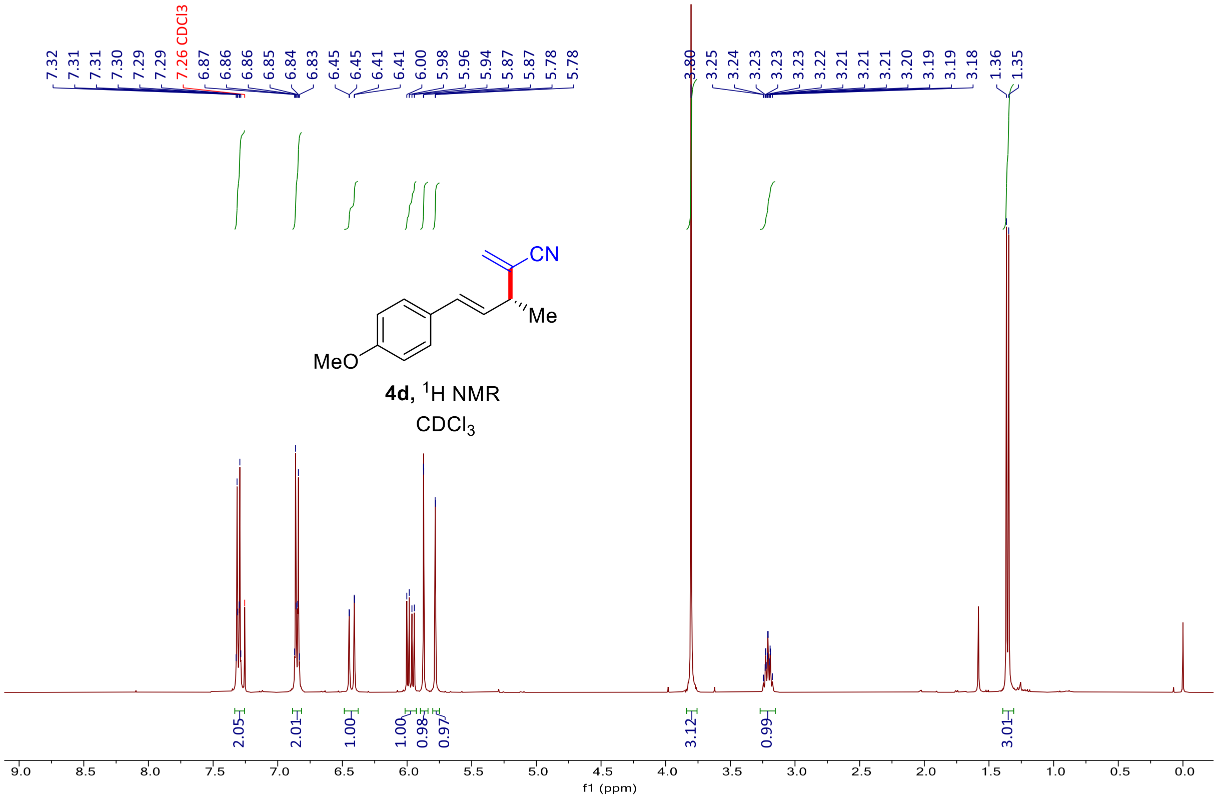

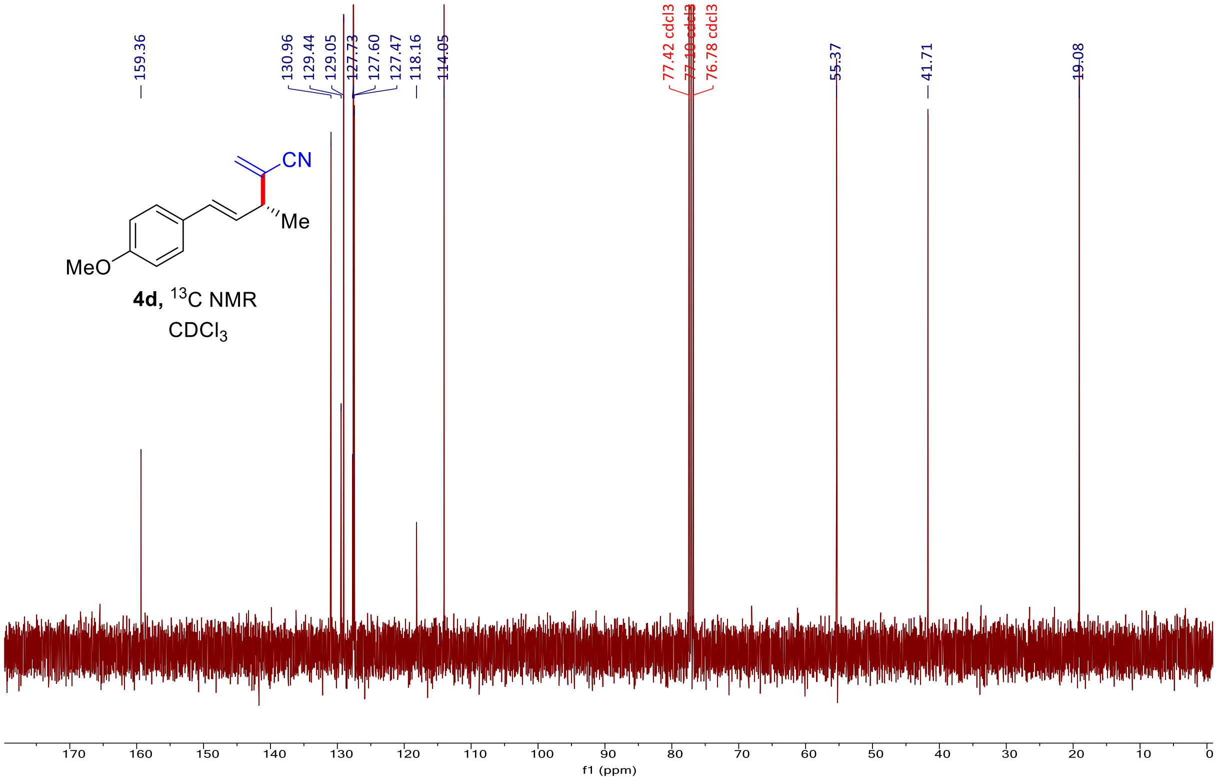

Supplementary Figure 8. ^{1}H NMR and ^{13}C NMR spectra of compound **4d**

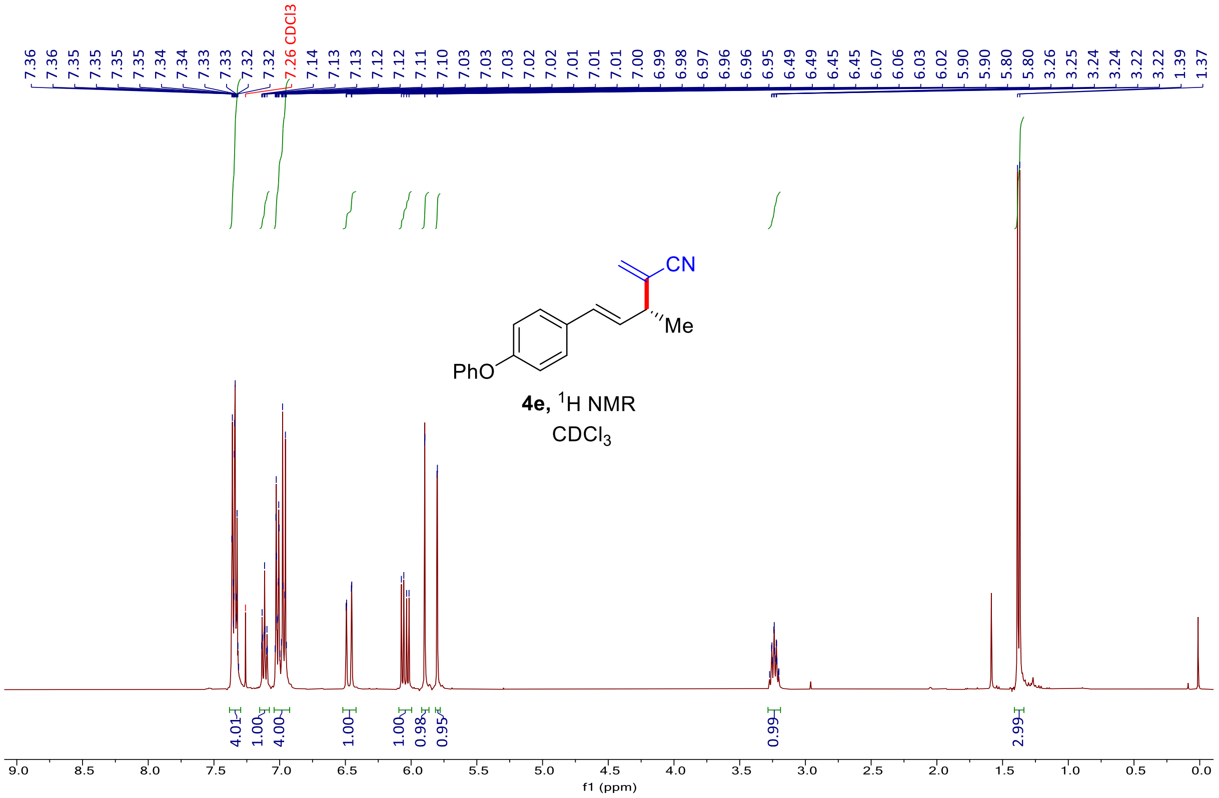

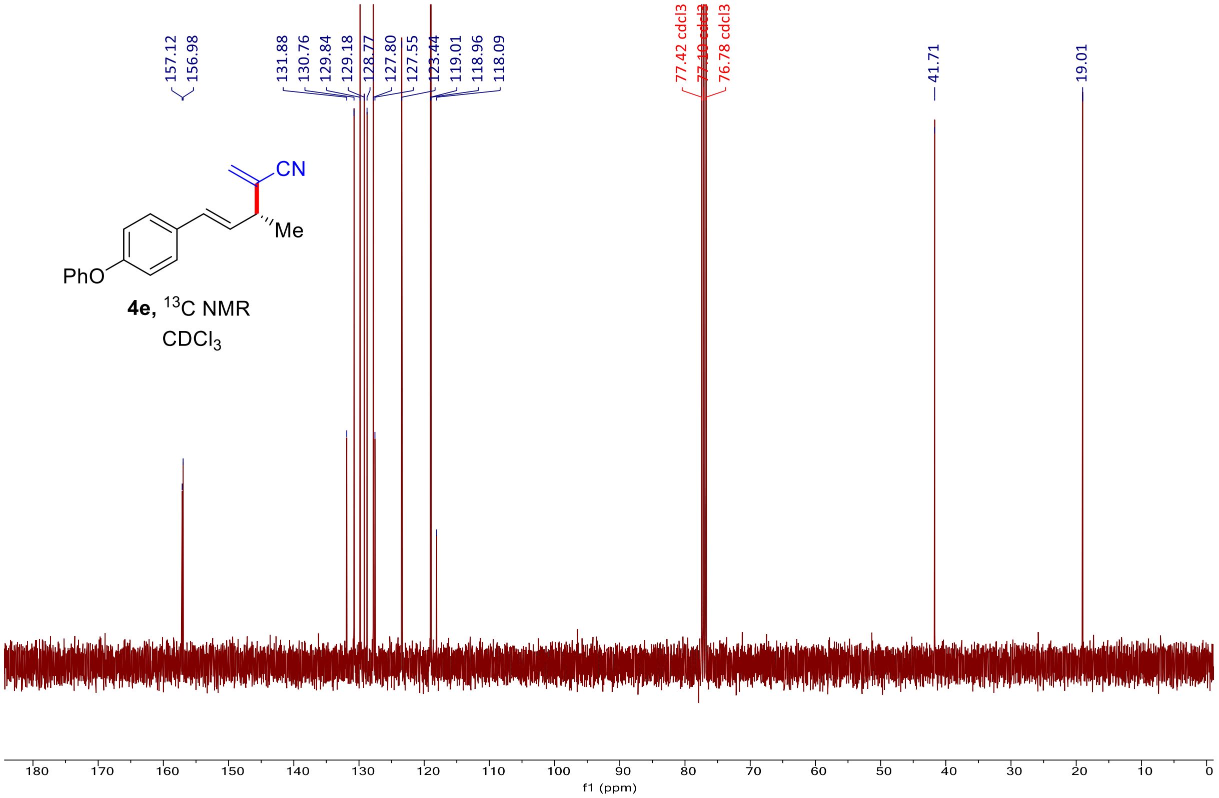

Supplementary Figure 9. ^{1}H NMR and ^{13}C NMR spectra of compound **4e**

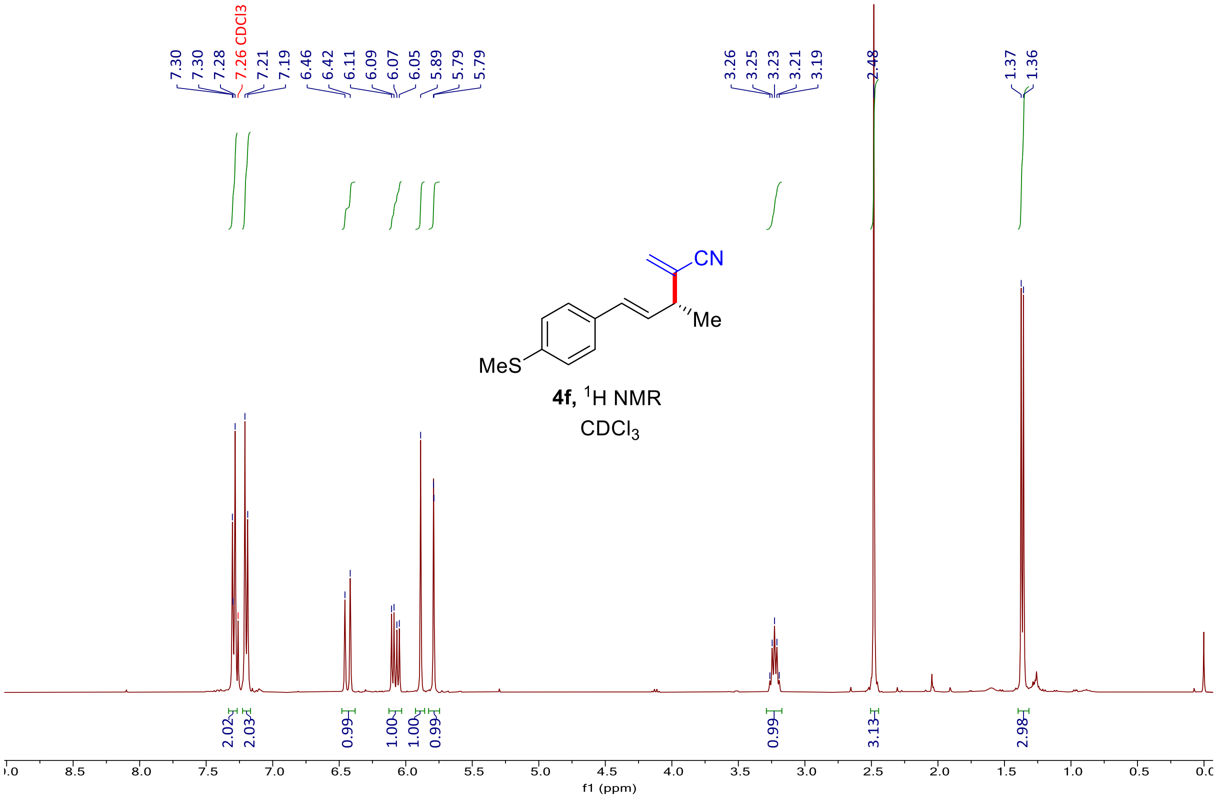

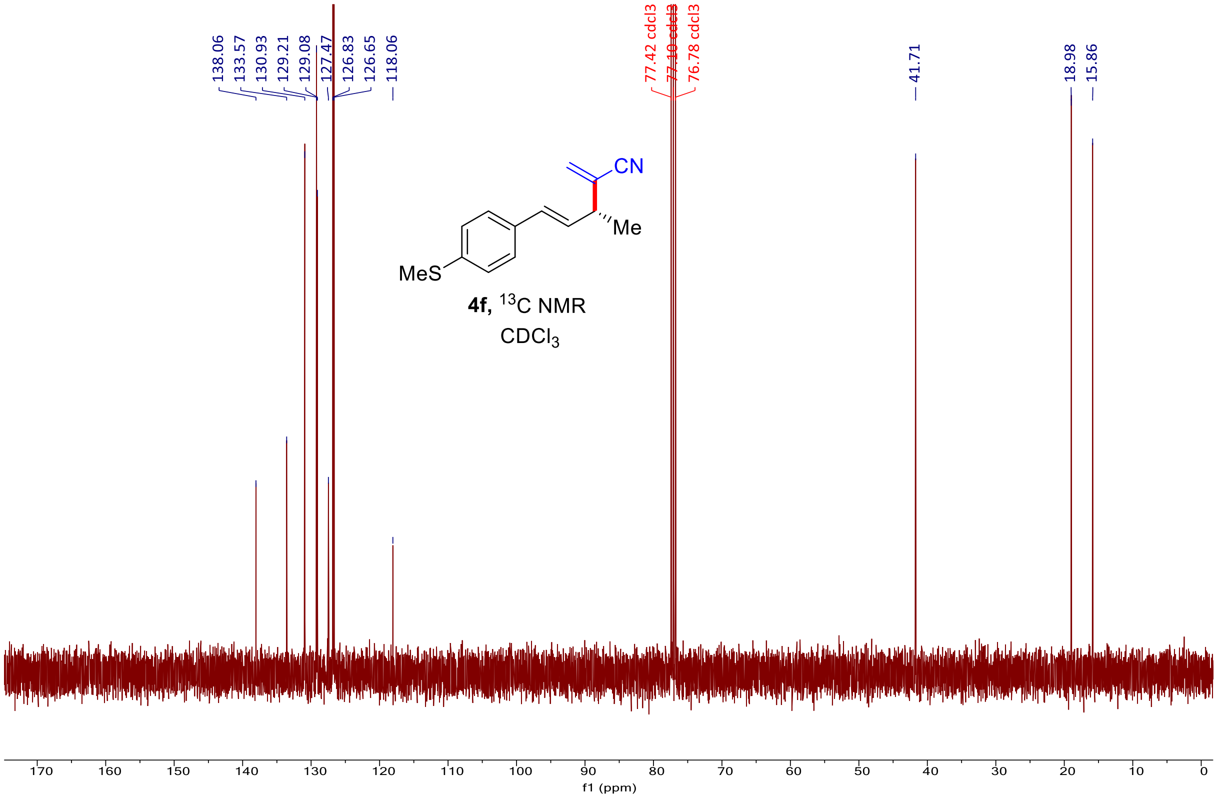

Supplementary Figure 10. ^{1}H NMR and ^{13}C NMR spectra of compound **4f**

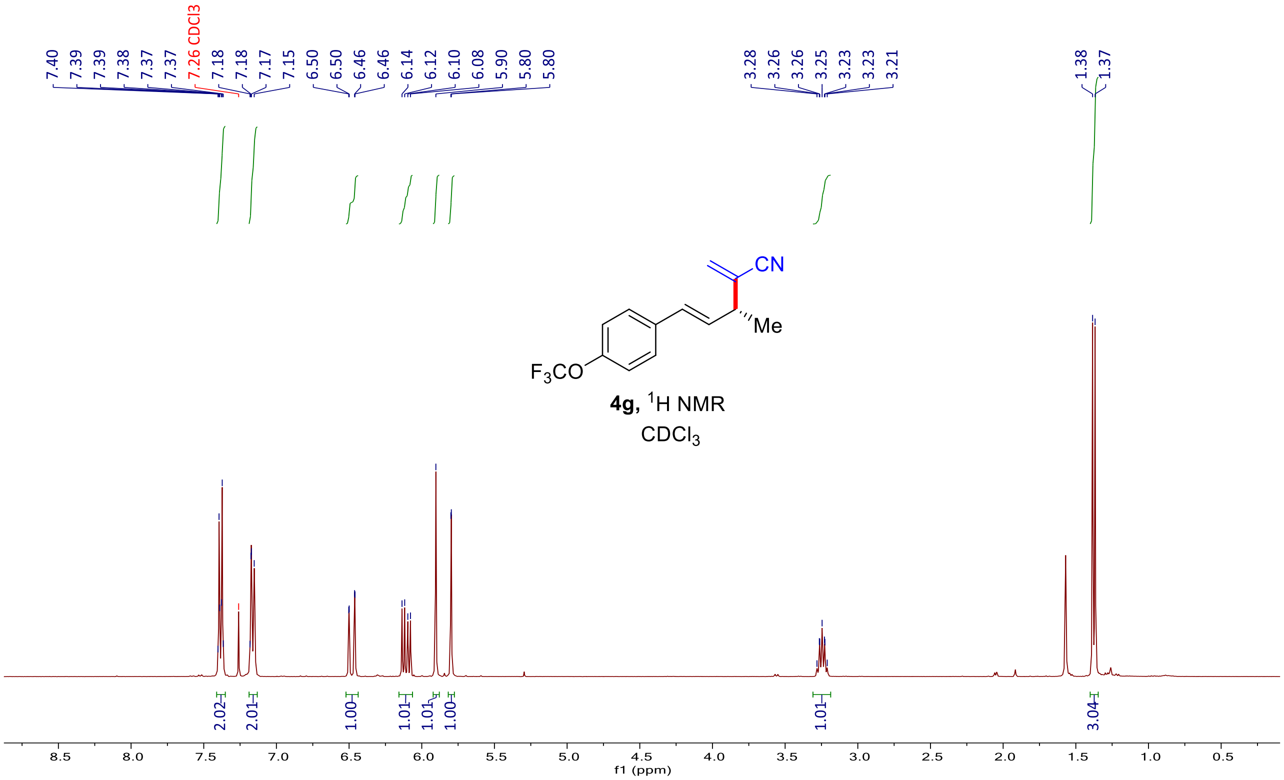

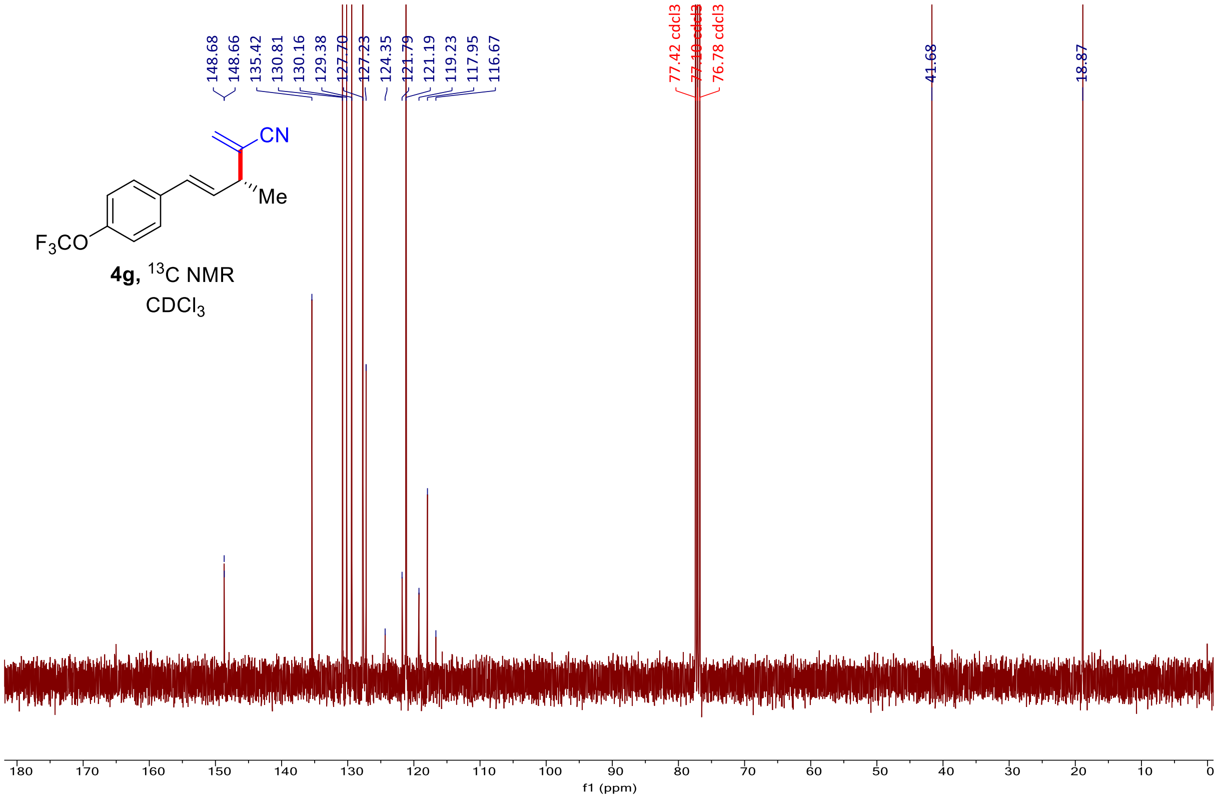

Supplementary Figure 11. ^{1}H NMR and ^{13}C NMR spectra of compound **4g**

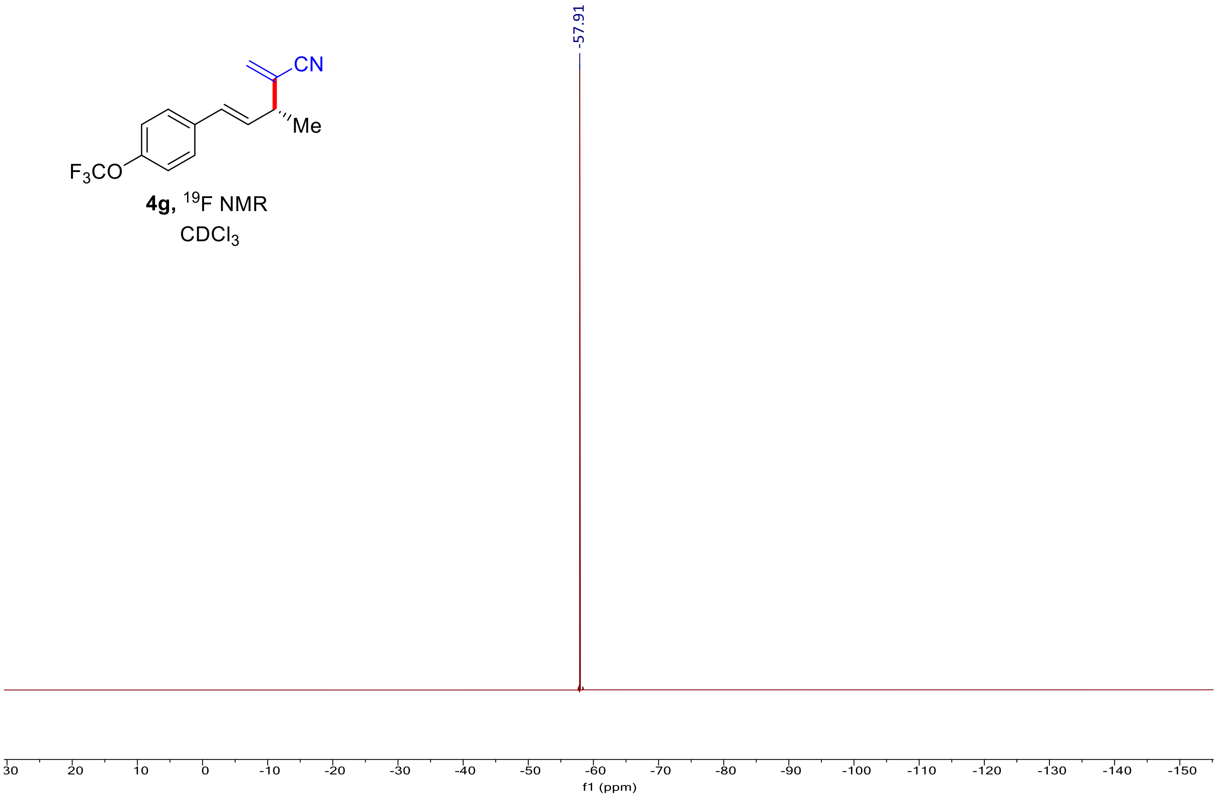

Supplementary Figure 12. ^{19}F NMR spectra of compound **4g**

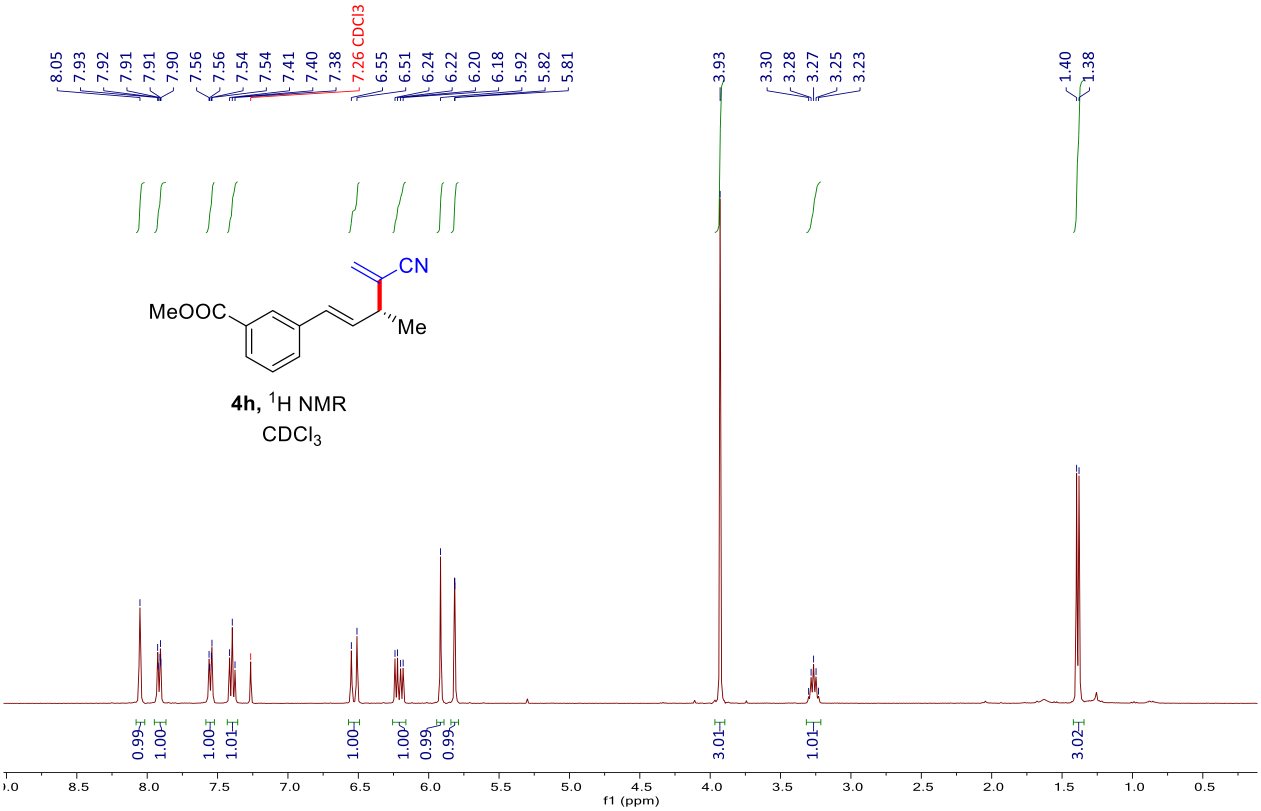

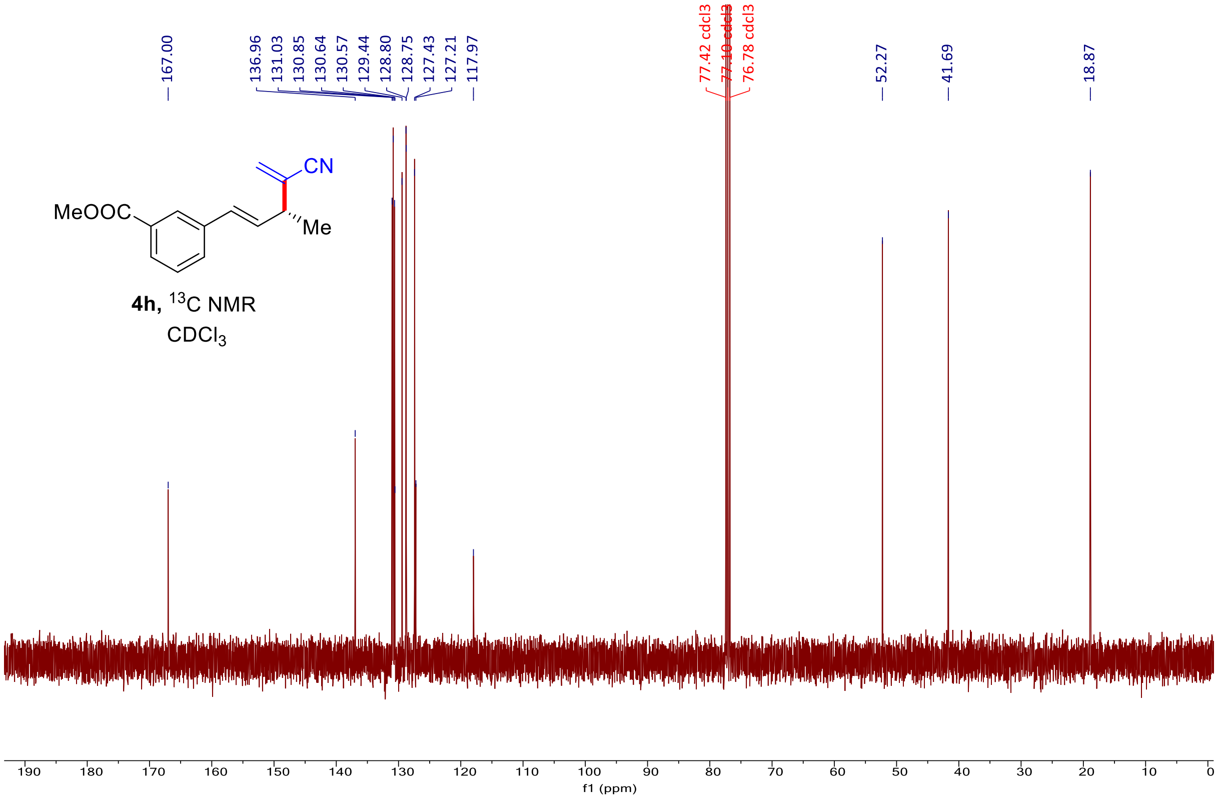

Supplementary Figure 13. ^{1}H NMR and ^{13}C NMR spectra of compound **4h**

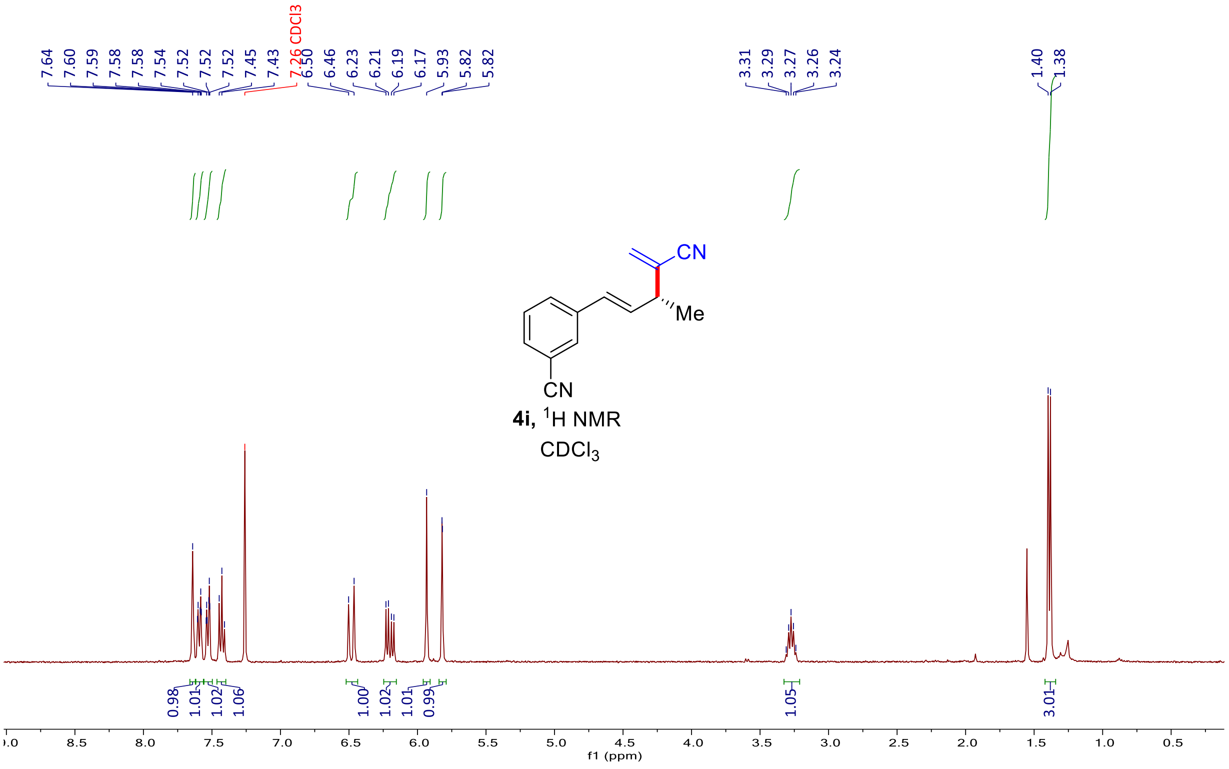

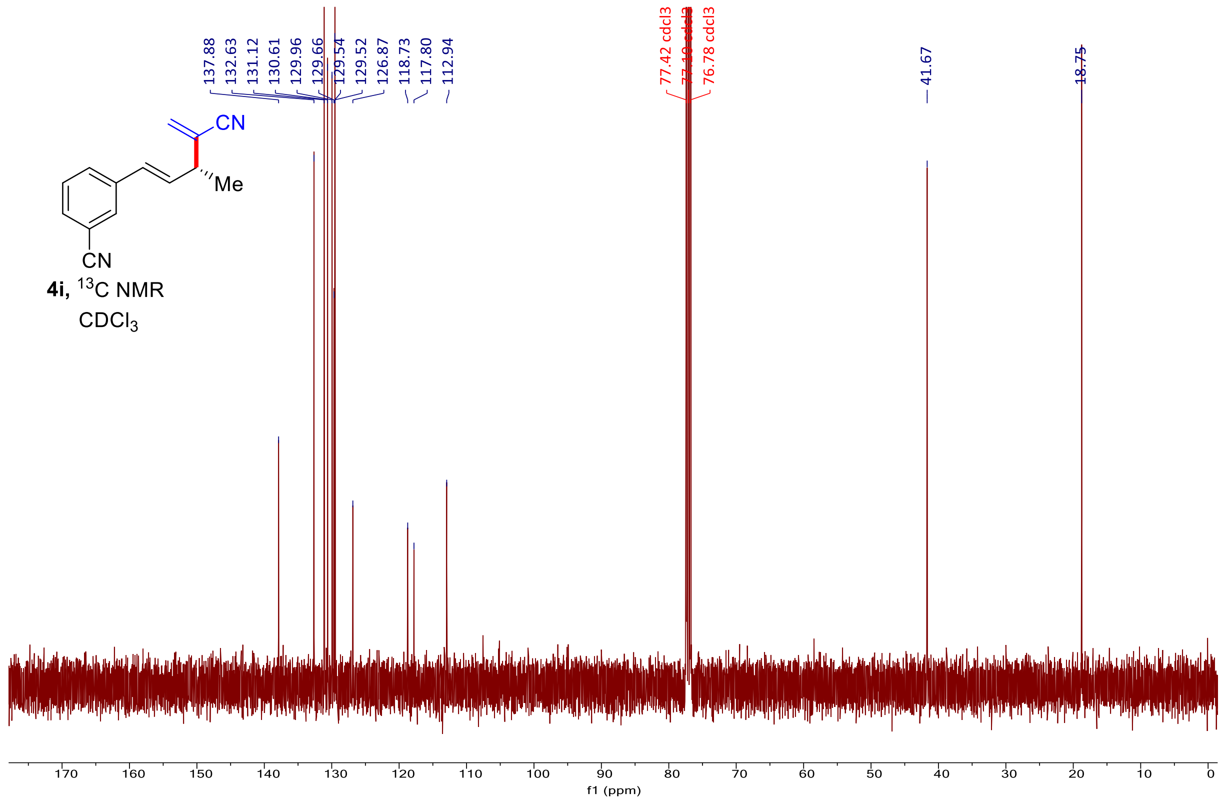

Supplementary Figure 14. ^{1}H NMR and ^{13}C NMR spectra of compound **4i**

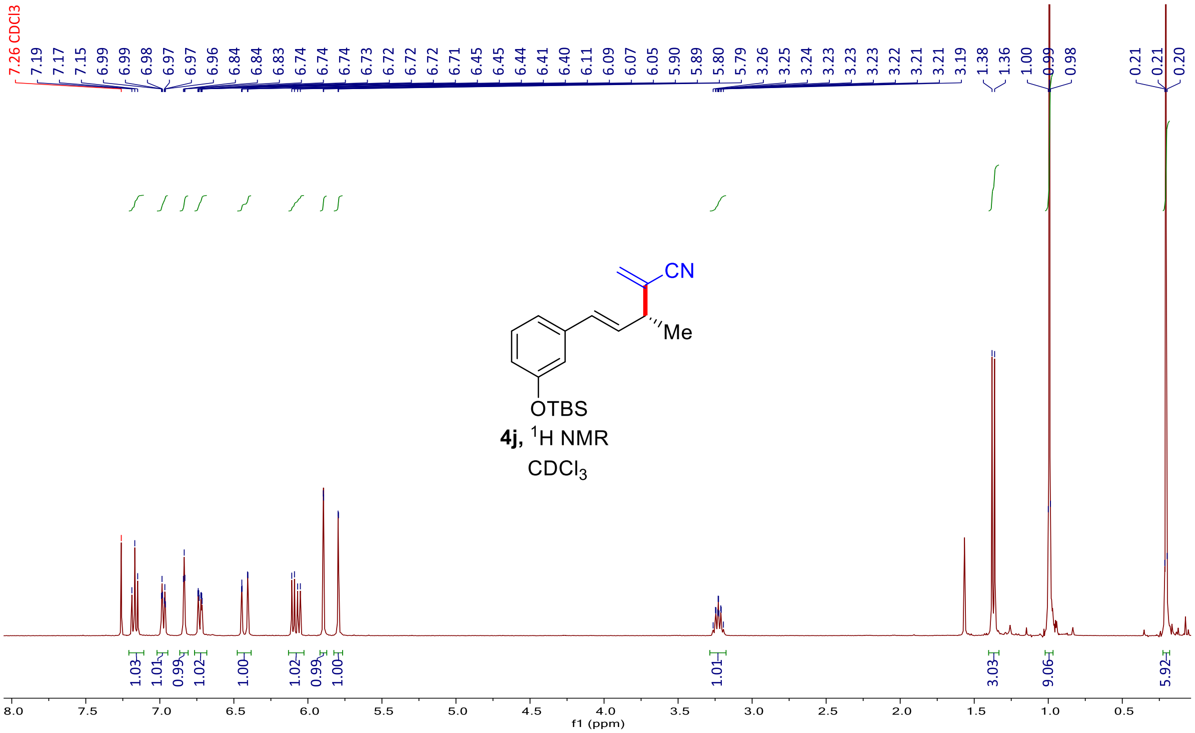

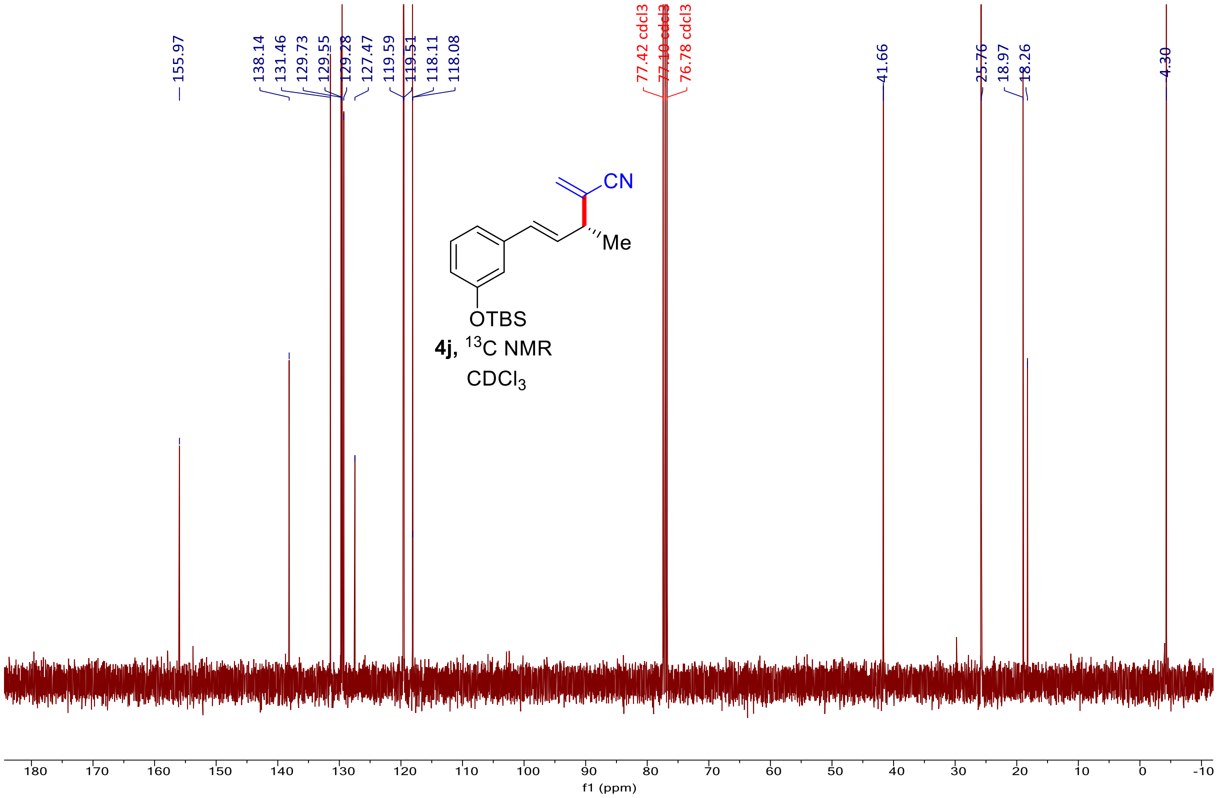

Supplementary Figure 15. ^{1}H NMR and ^{13}C NMR spectra of compound **4j**

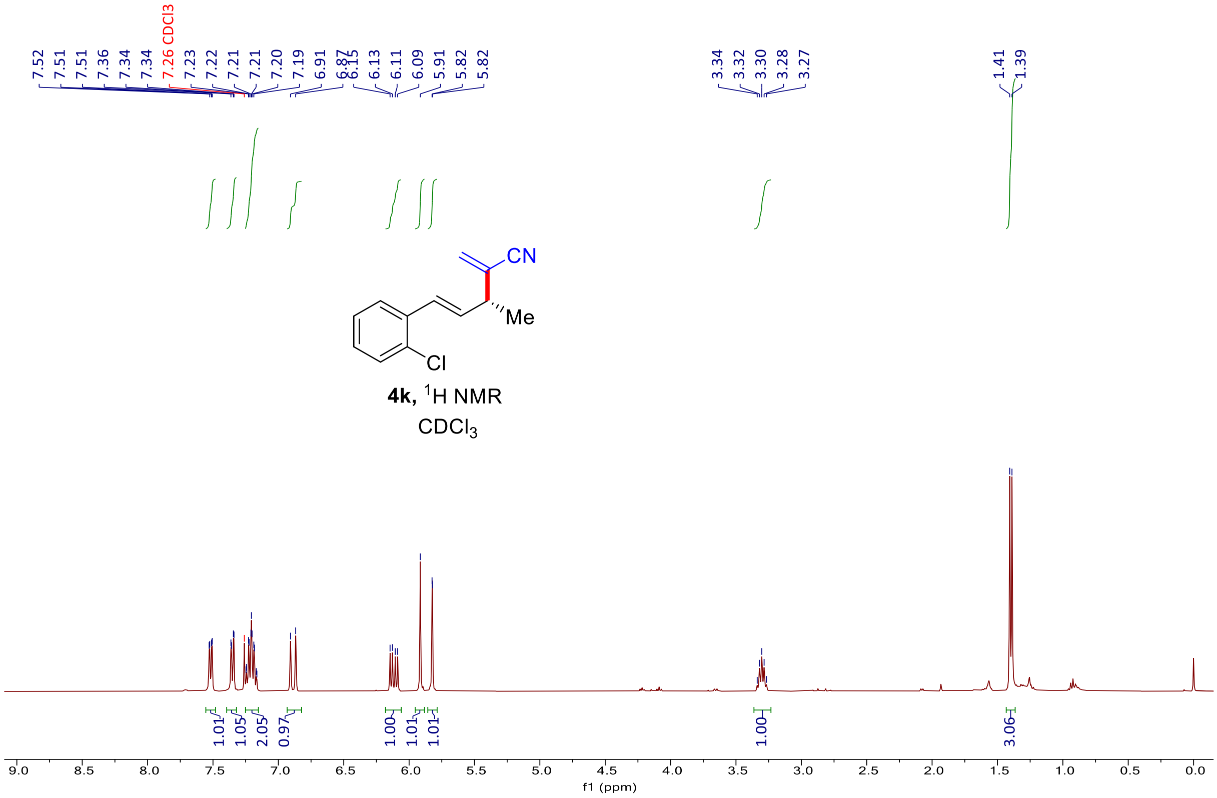

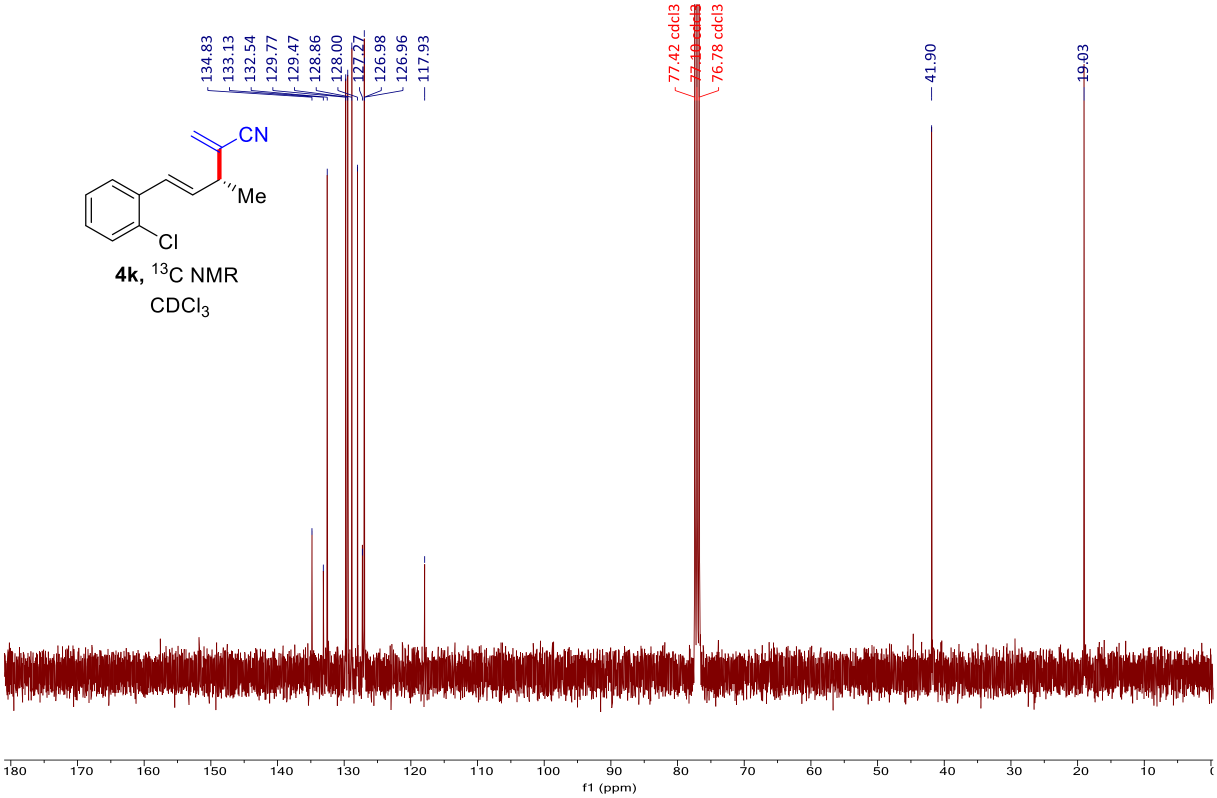

Supplementary Figure 16. ^{1}H NMR and ^{13}C NMR spectra of compound **4k**

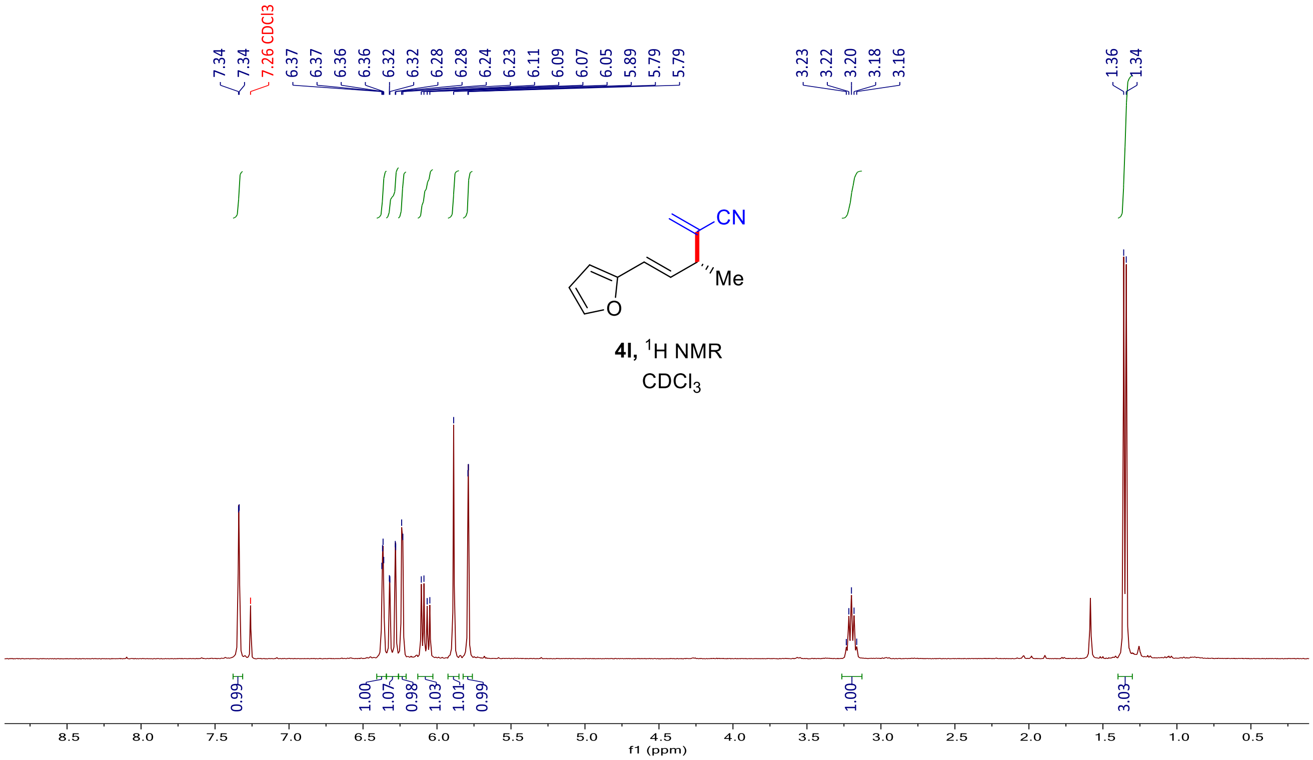

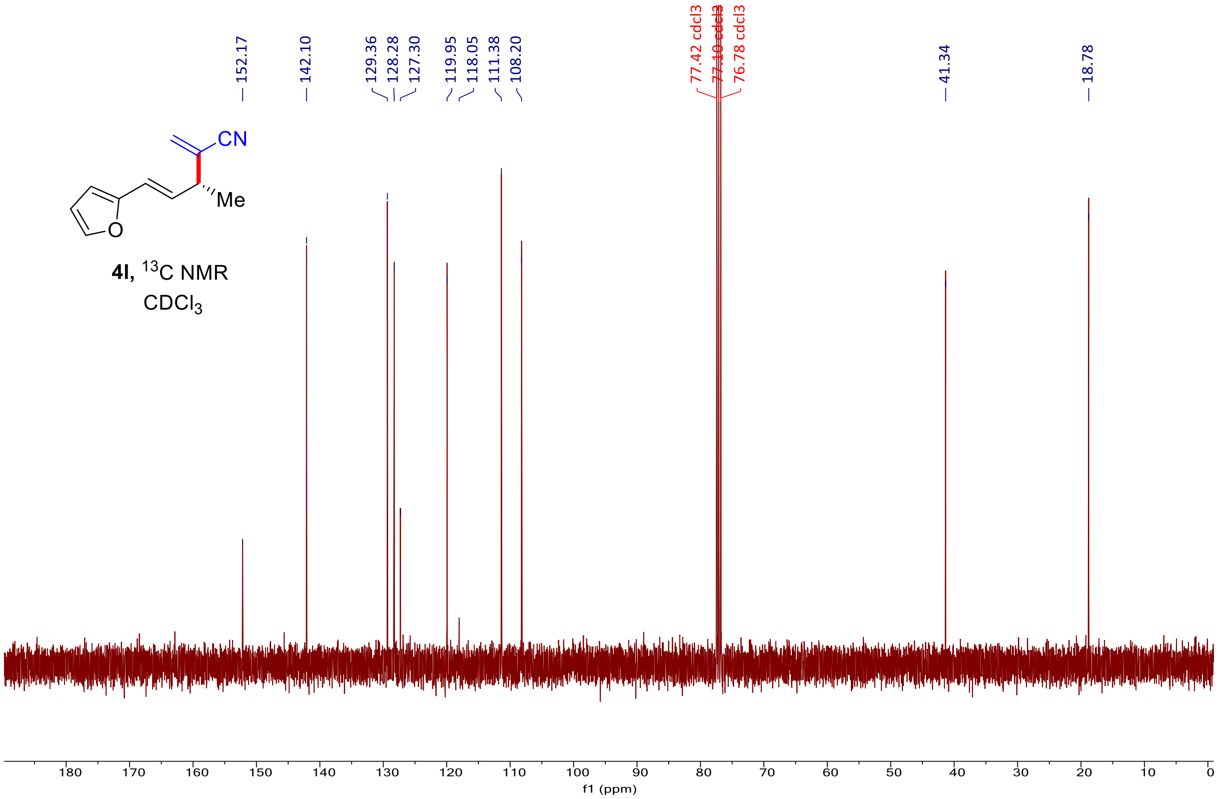

Supplementary Figure 17. ^{1}H NMR and ^{13}C NMR spectra of compound **4l**

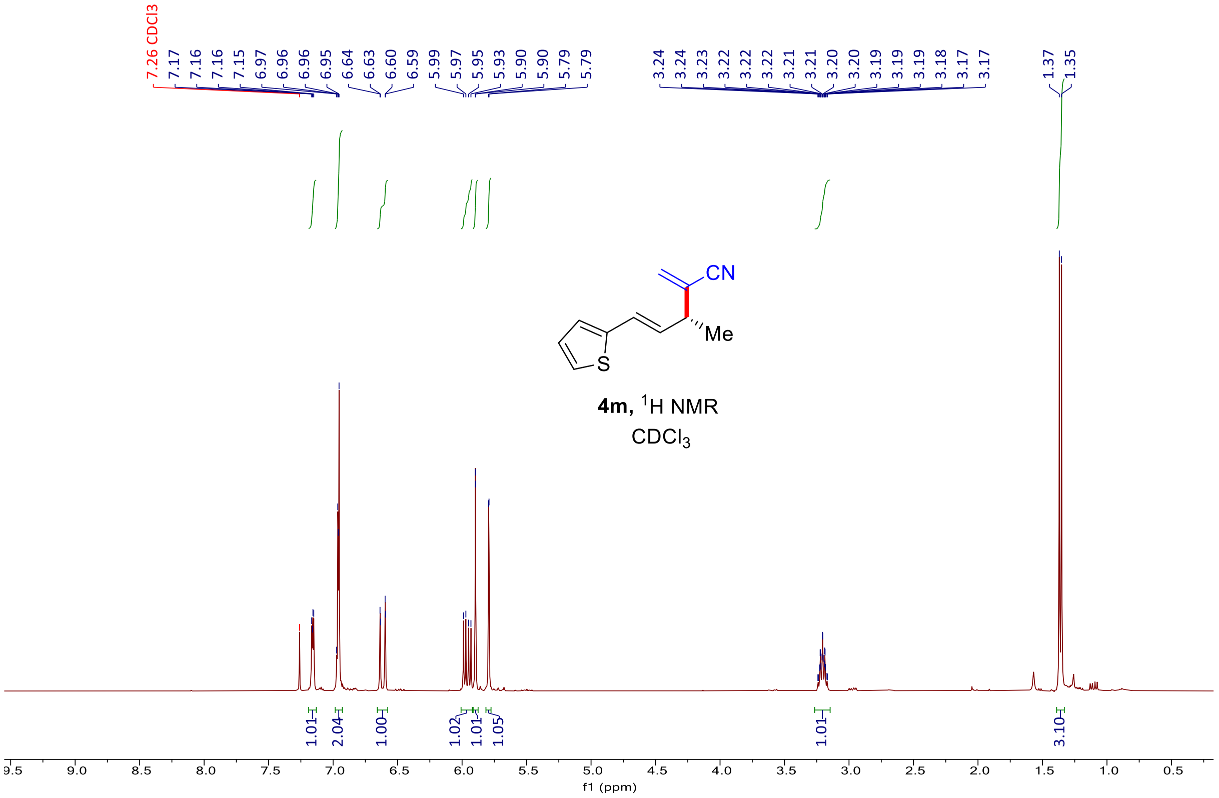

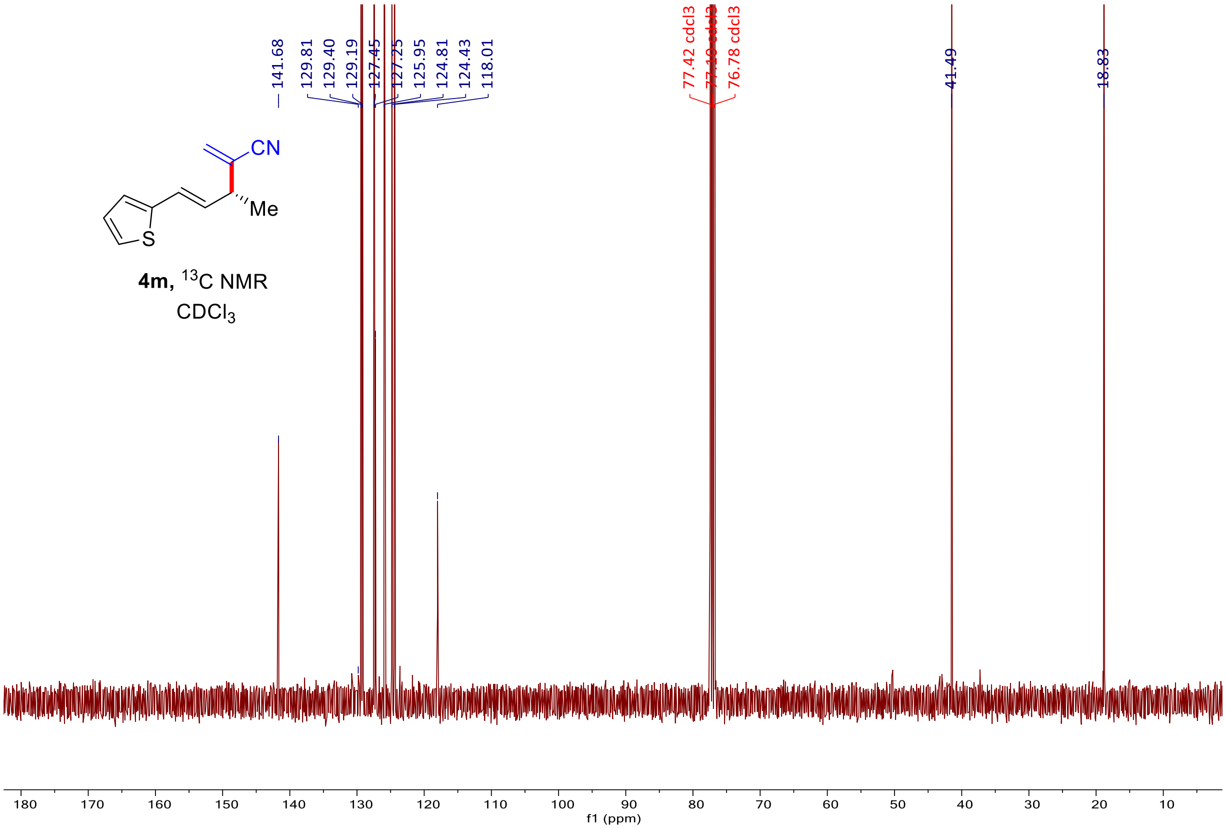

Supplementary Figure 18. ^{1}H NMR and ^{13}C NMR spectra of compound **4m**

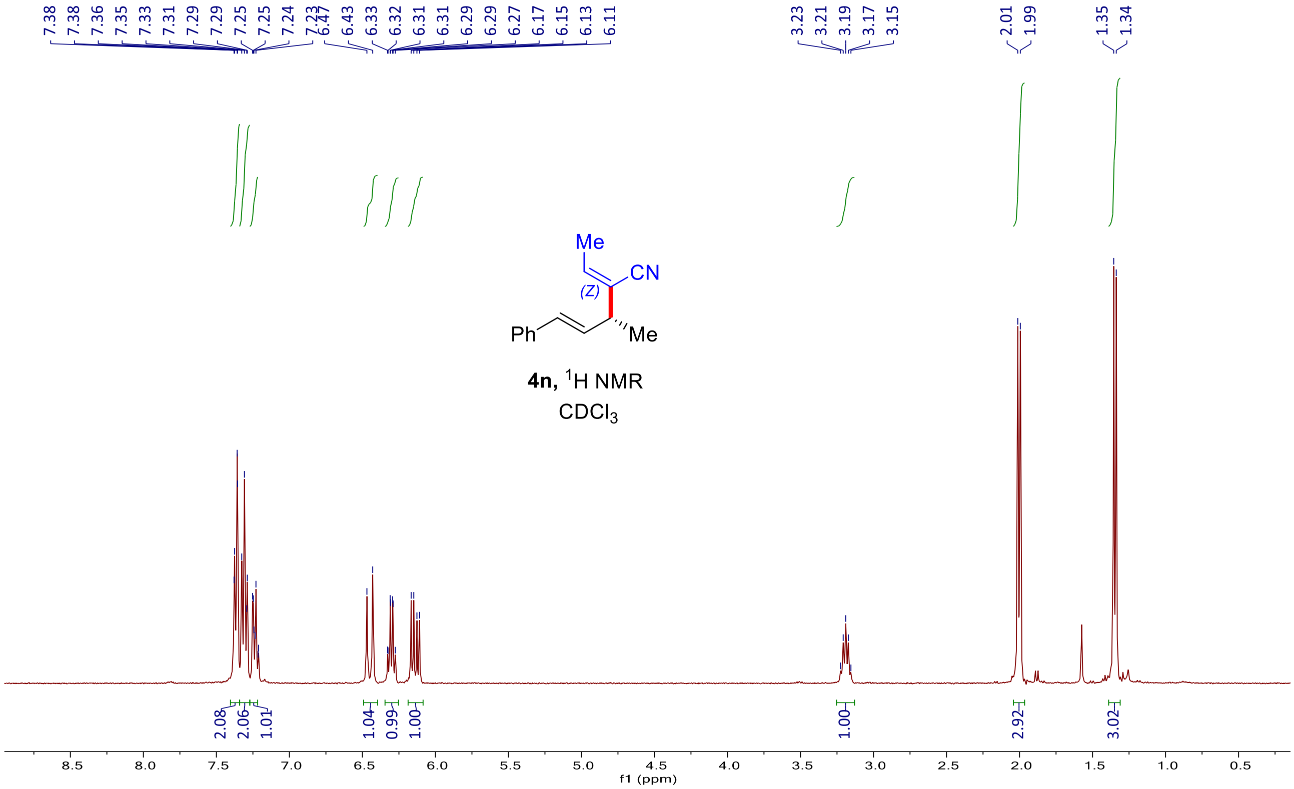

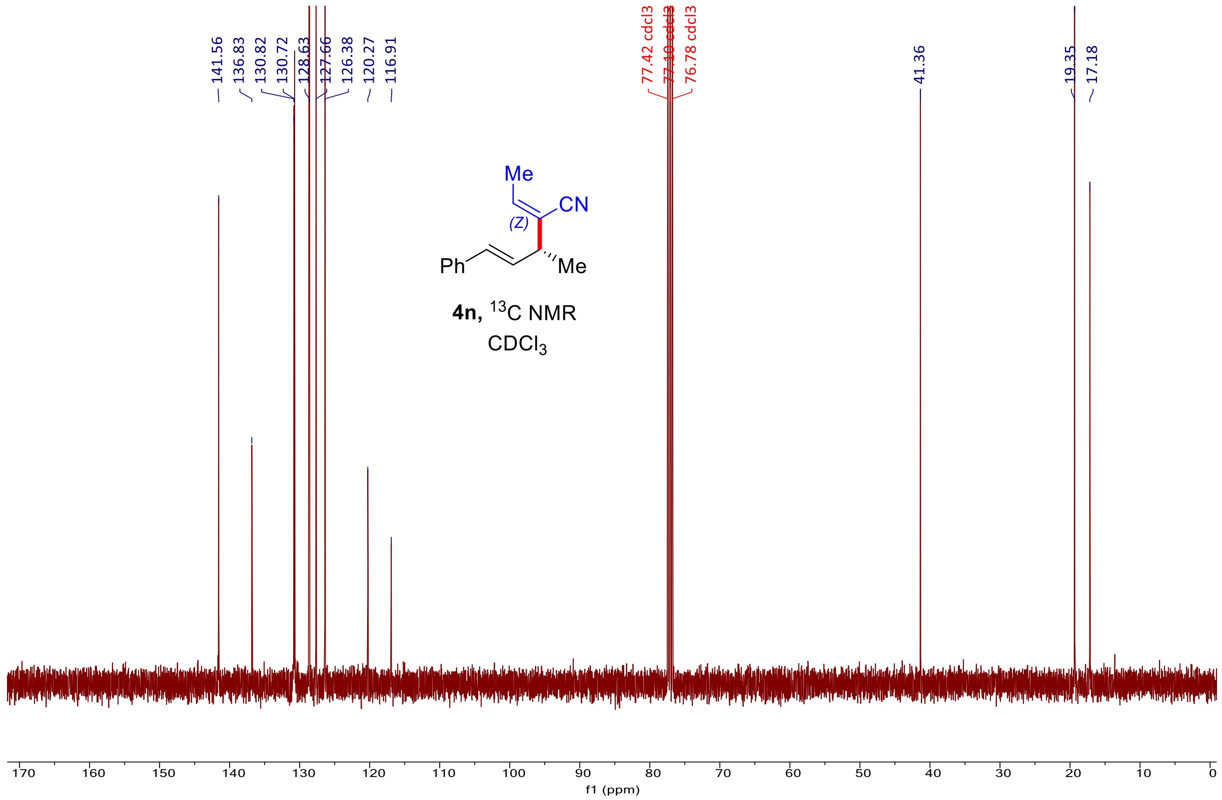

Supplementary Figure 19. ^{1}H NMR and ^{13}C NMR spectra of compound **4n**

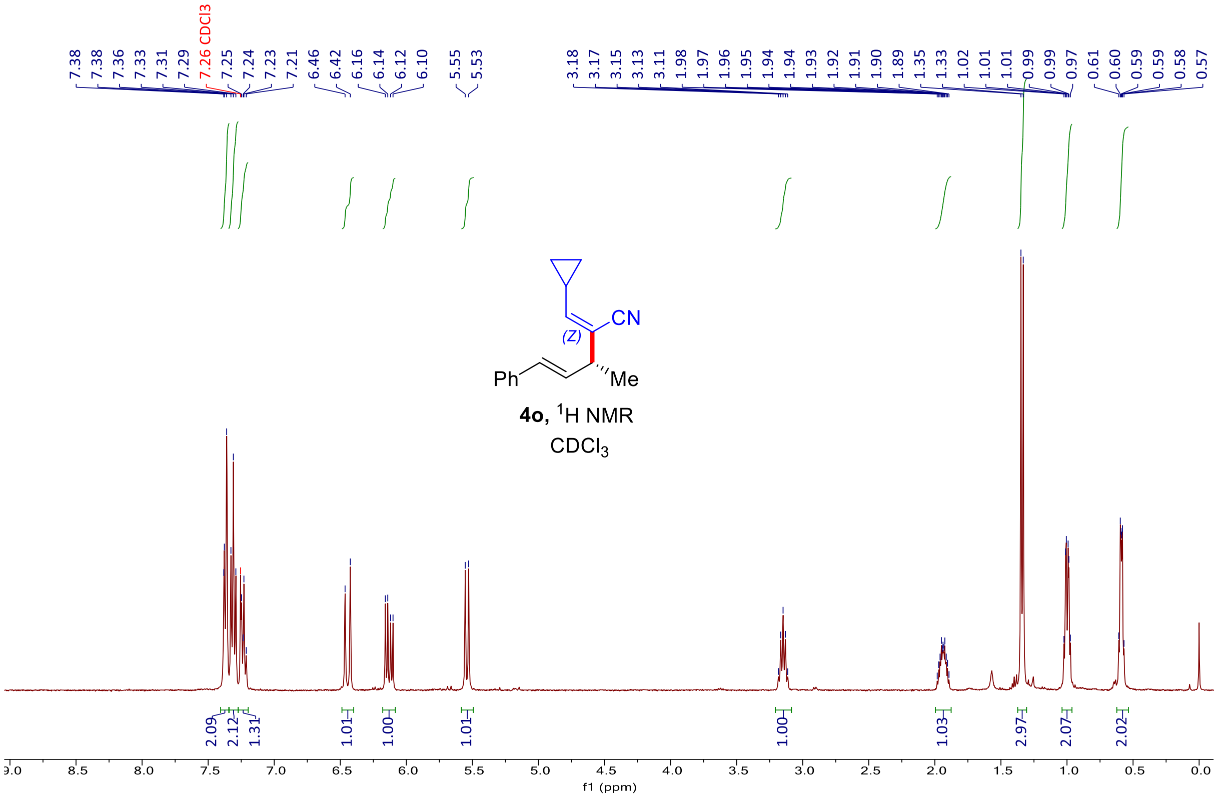

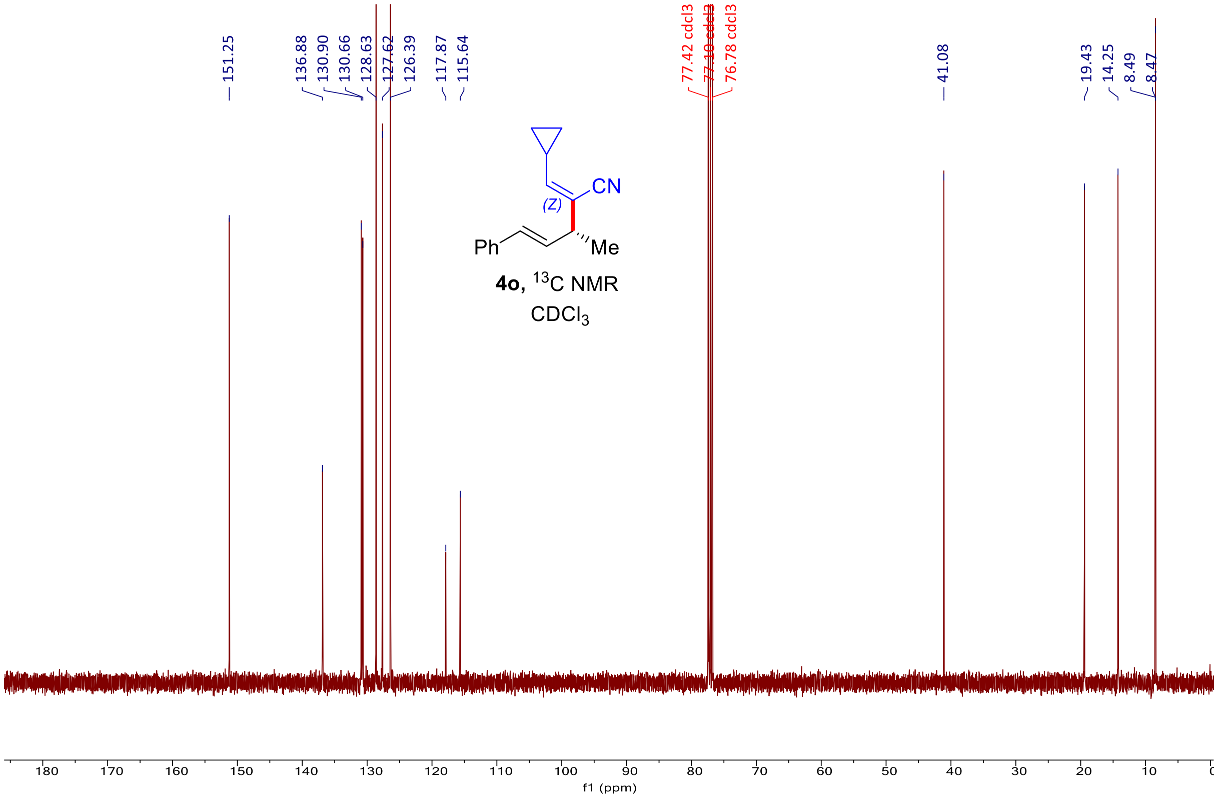

Supplementary Figure 20. ^{1}H NMR and ^{13}C NMR spectra of compound **4o**

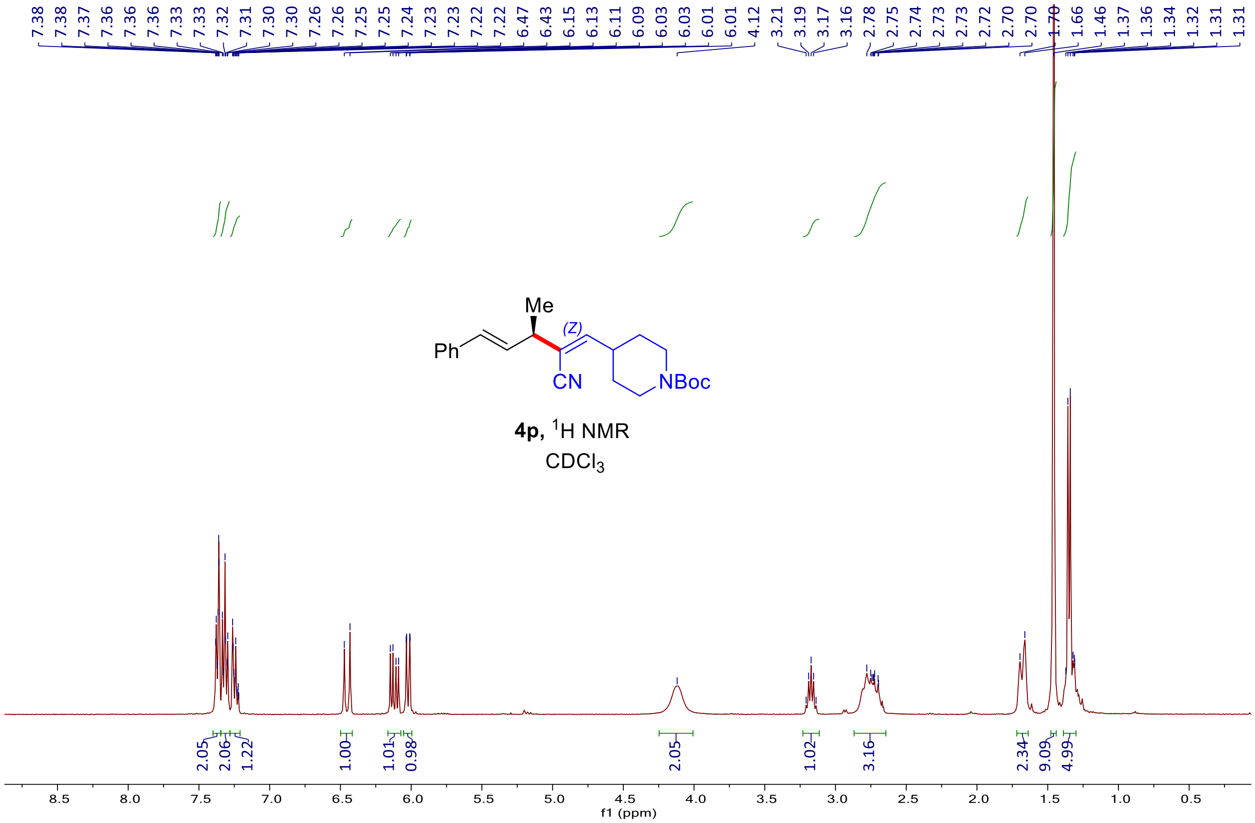

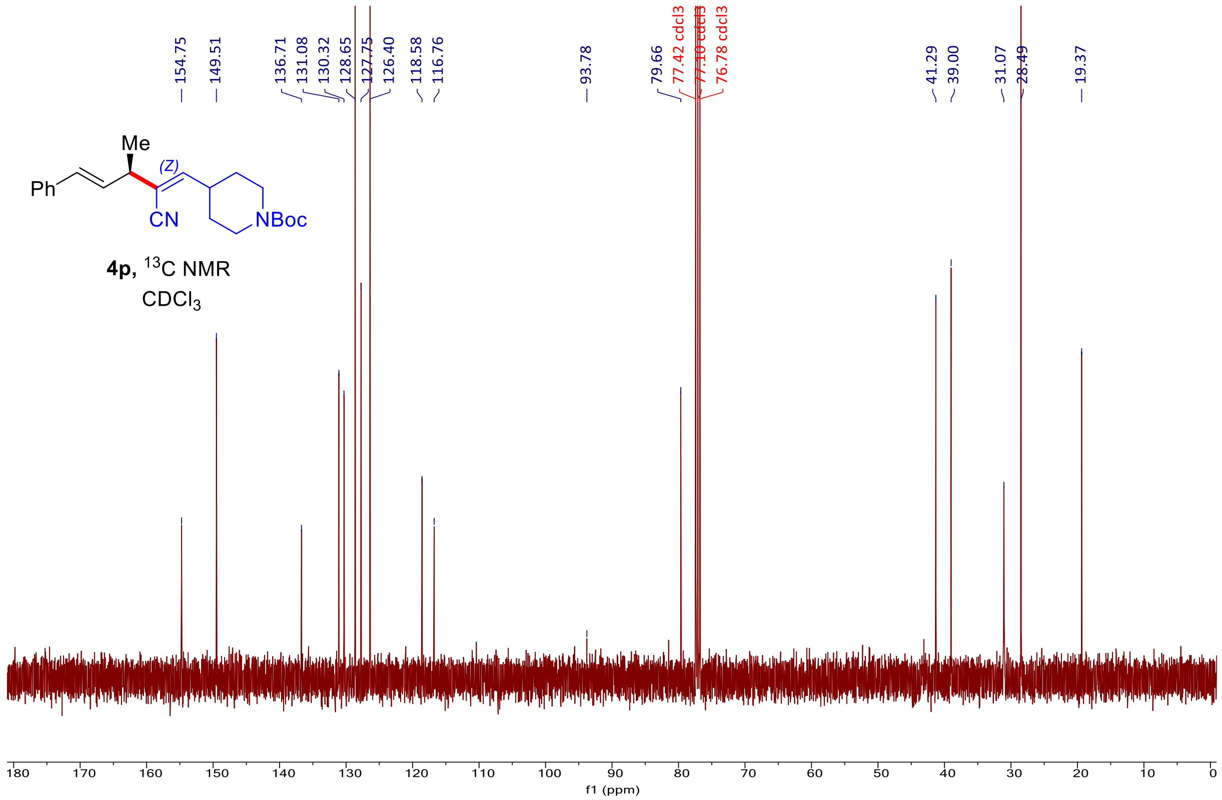

Supplementary Figure 21. ^{1}H NMR and ^{13}C NMR spectra of compound **4p**

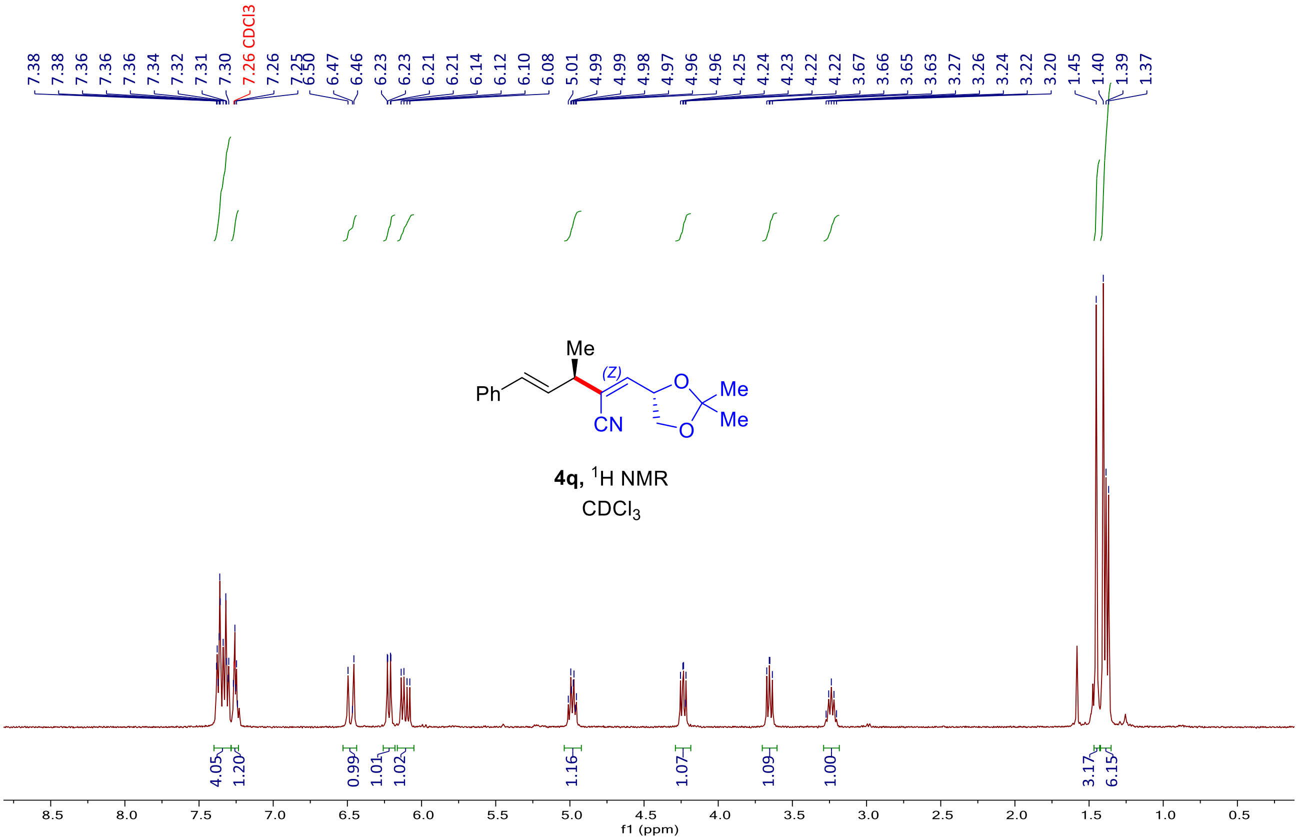

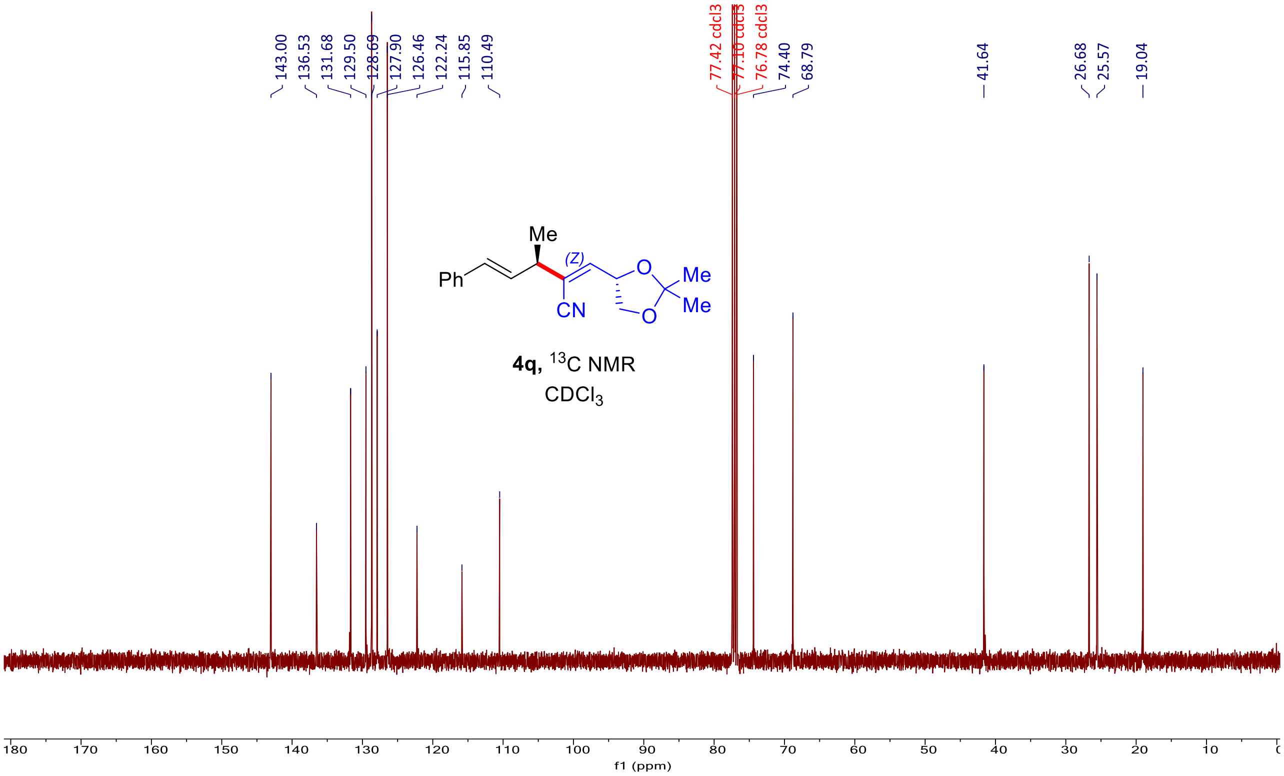

Supplementary Figure 22. ^{1}H NMR and ^{13}C NMR spectra of compound **4q**

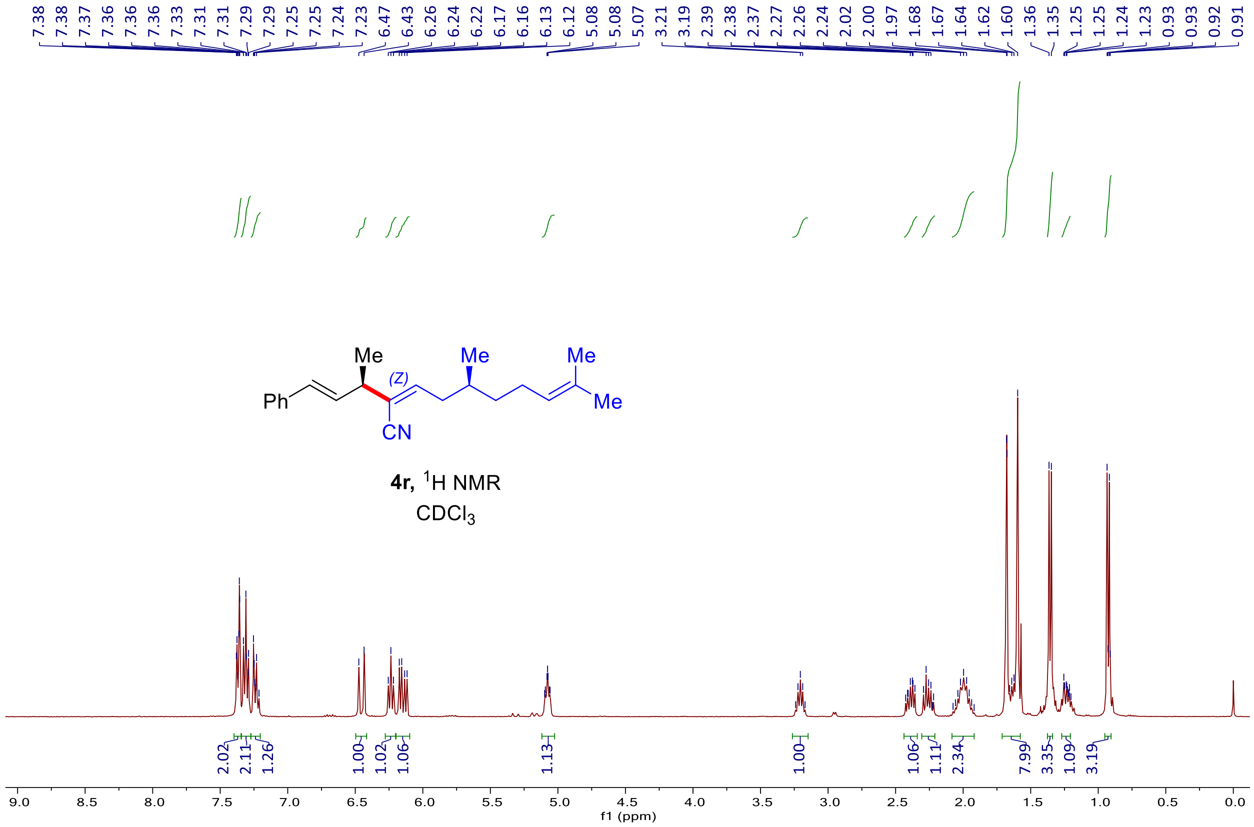

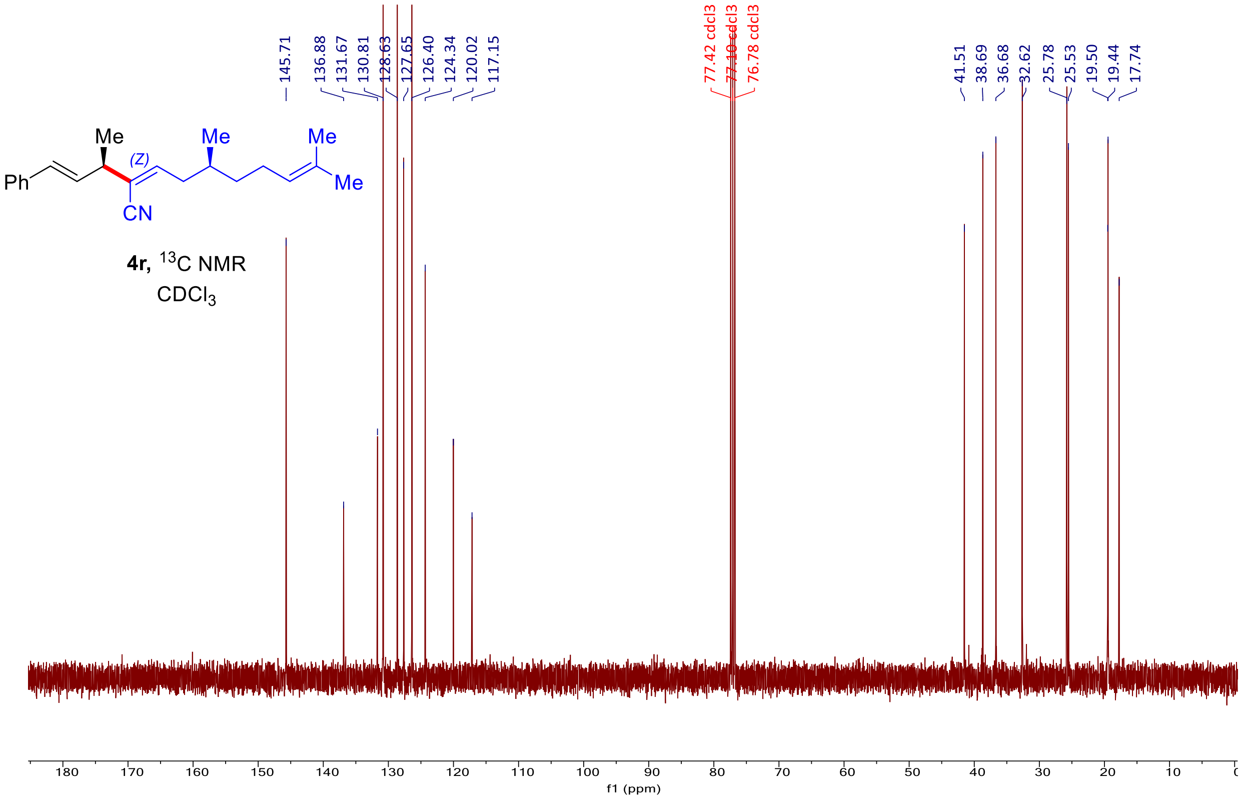

Supplementary Figure 23. ^{1}H NMR and ^{13}C NMR spectra of compound **4r**

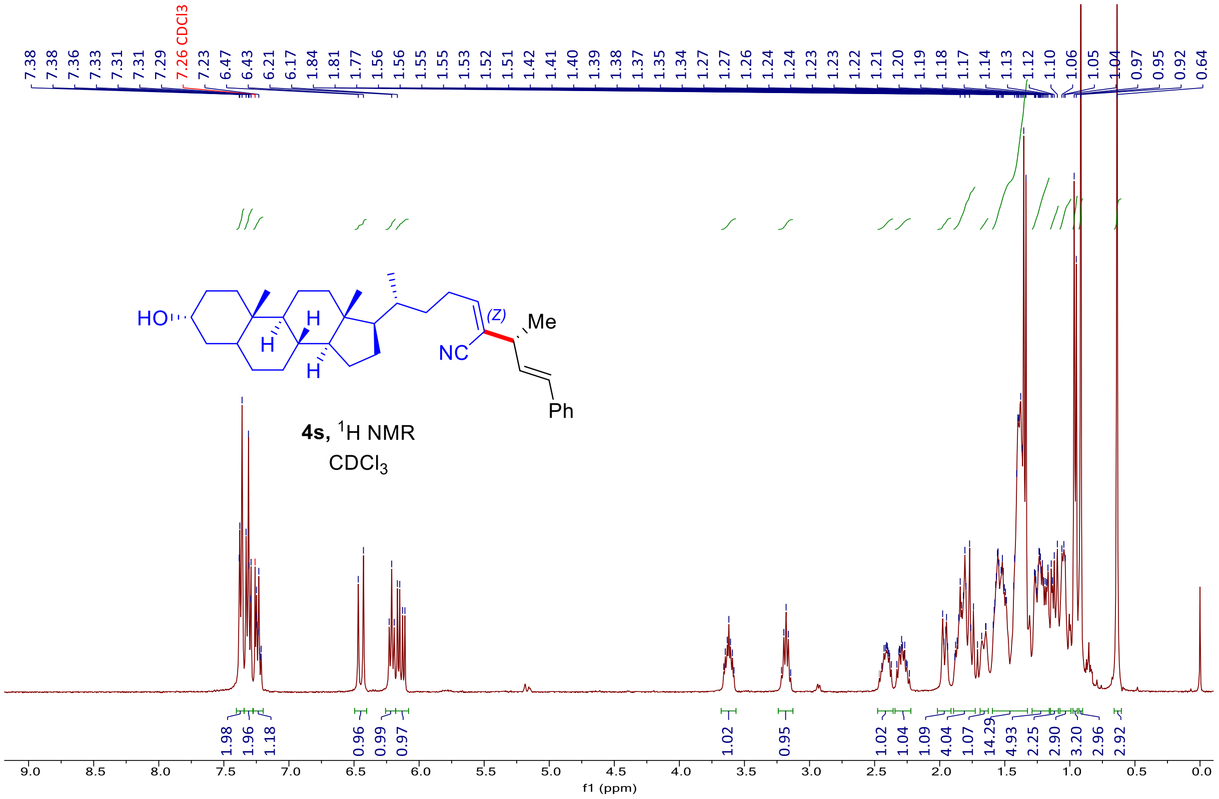

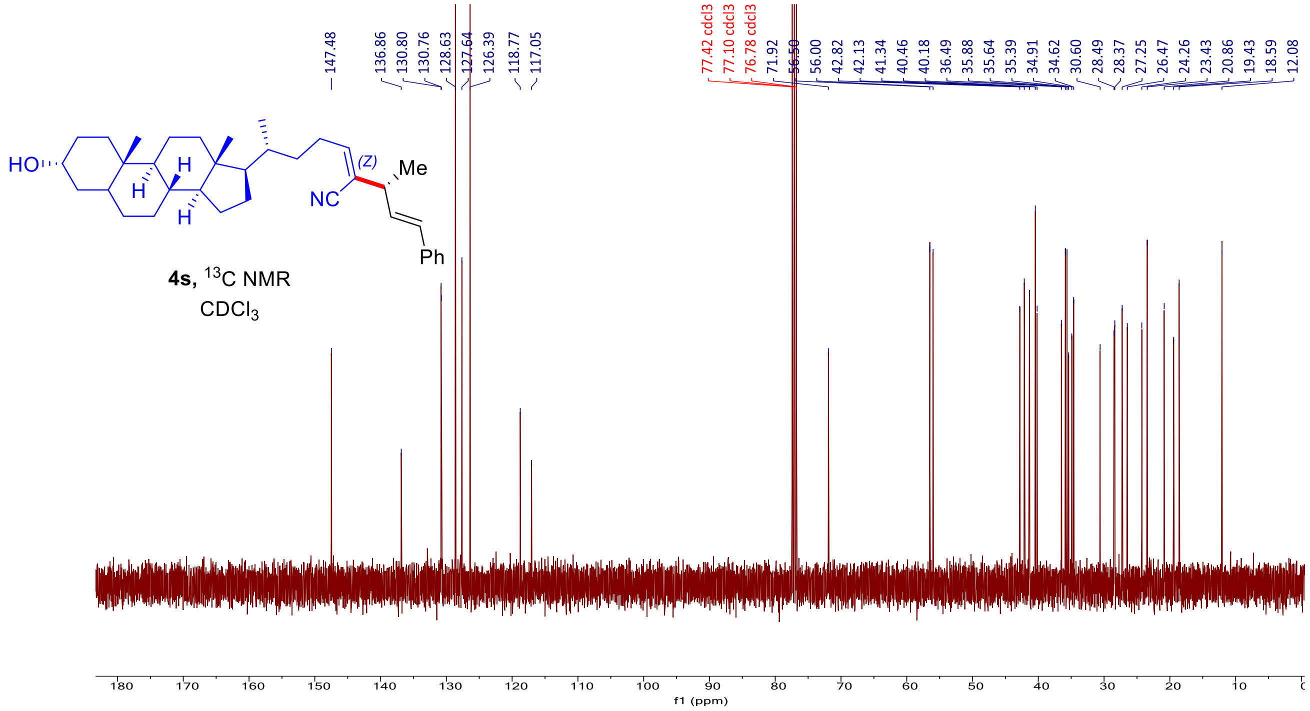

Supplementary Figure 24. ^{1}H NMR and ^{13}C NMR spectra of compound **4s**

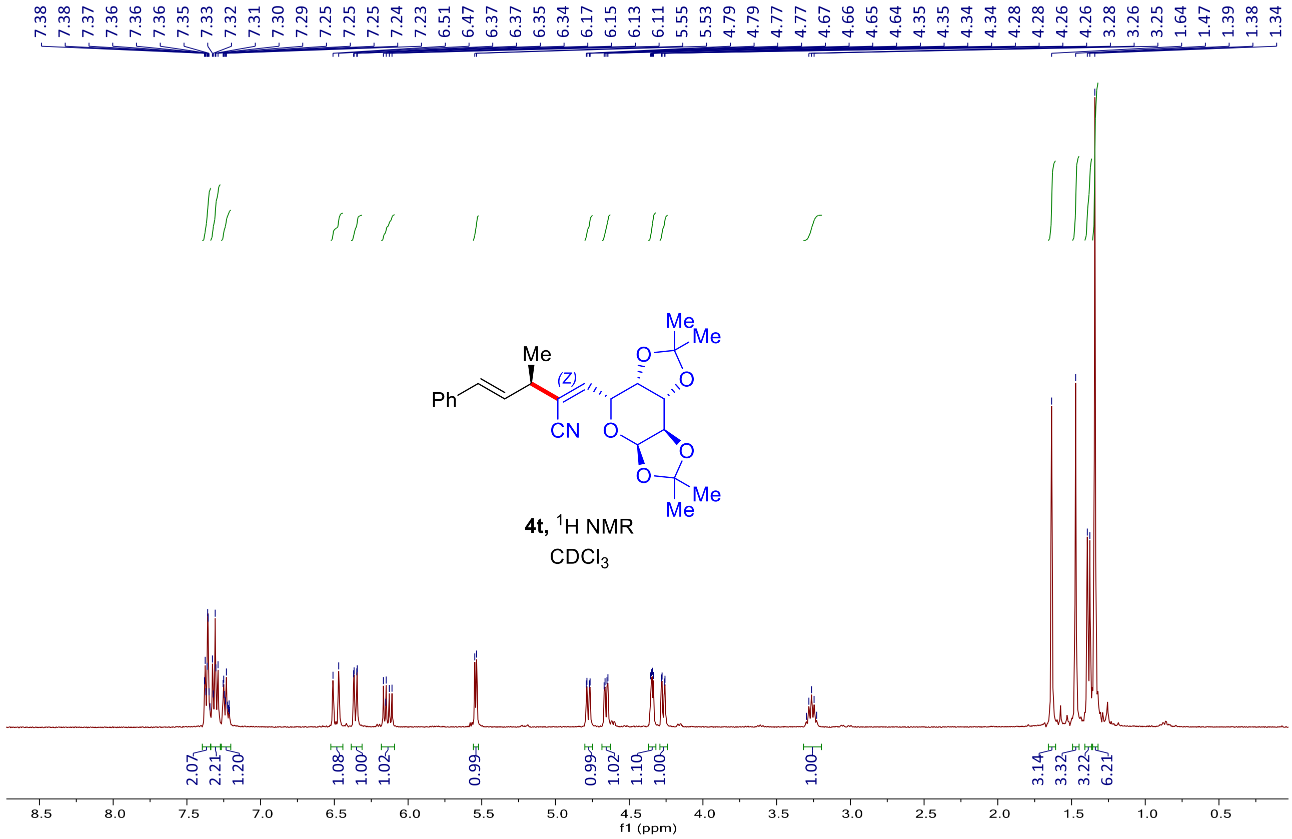

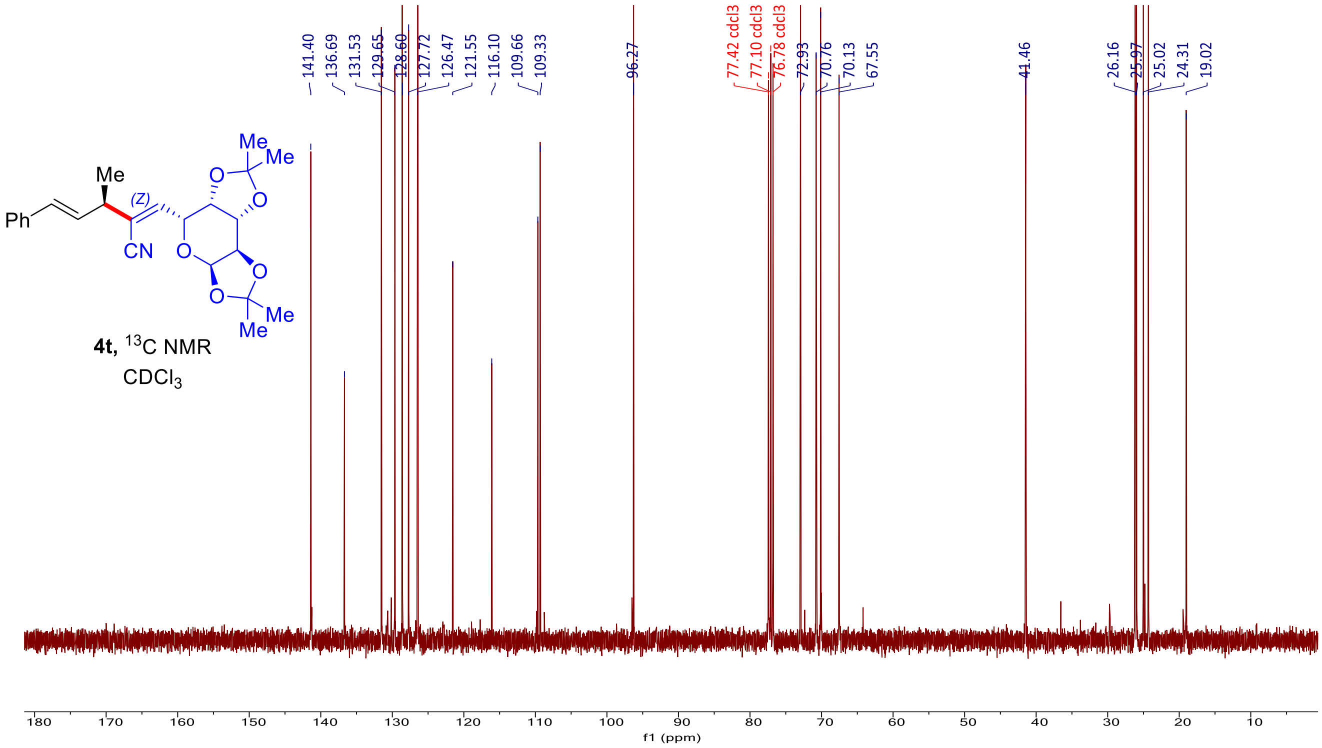

Supplementary Figure 25. ^{1}H NMR and ^{13}C NMR spectra of compound **4t**

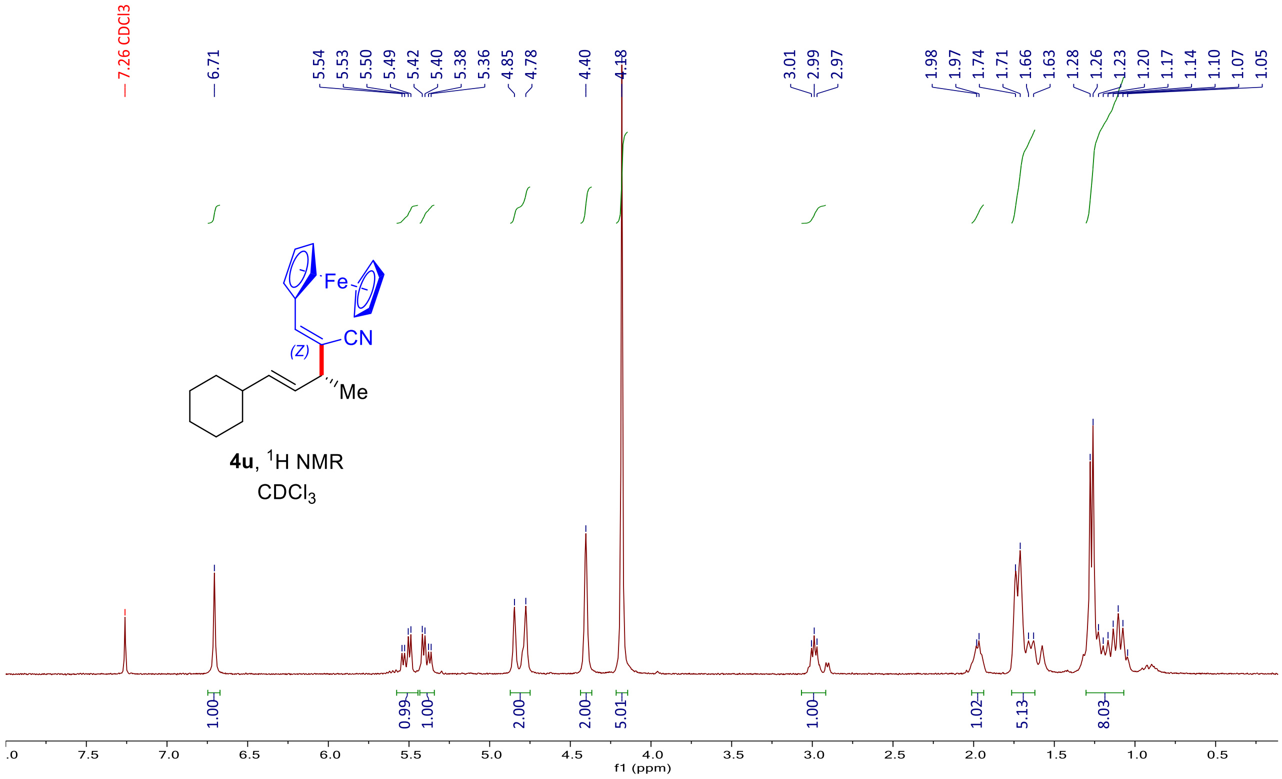

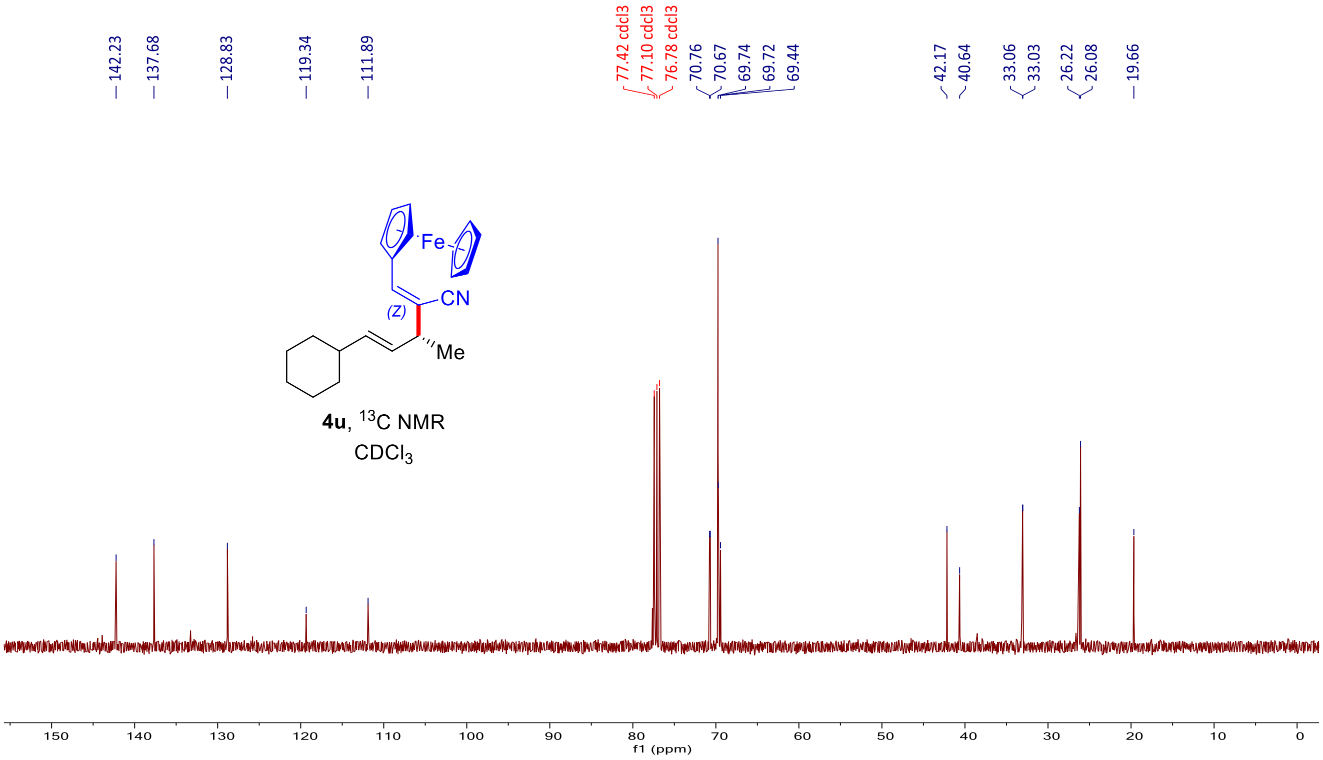

Supplementary Figure 26. ^{1}H NMR and ^{13}C NMR spectra of compound **4u**

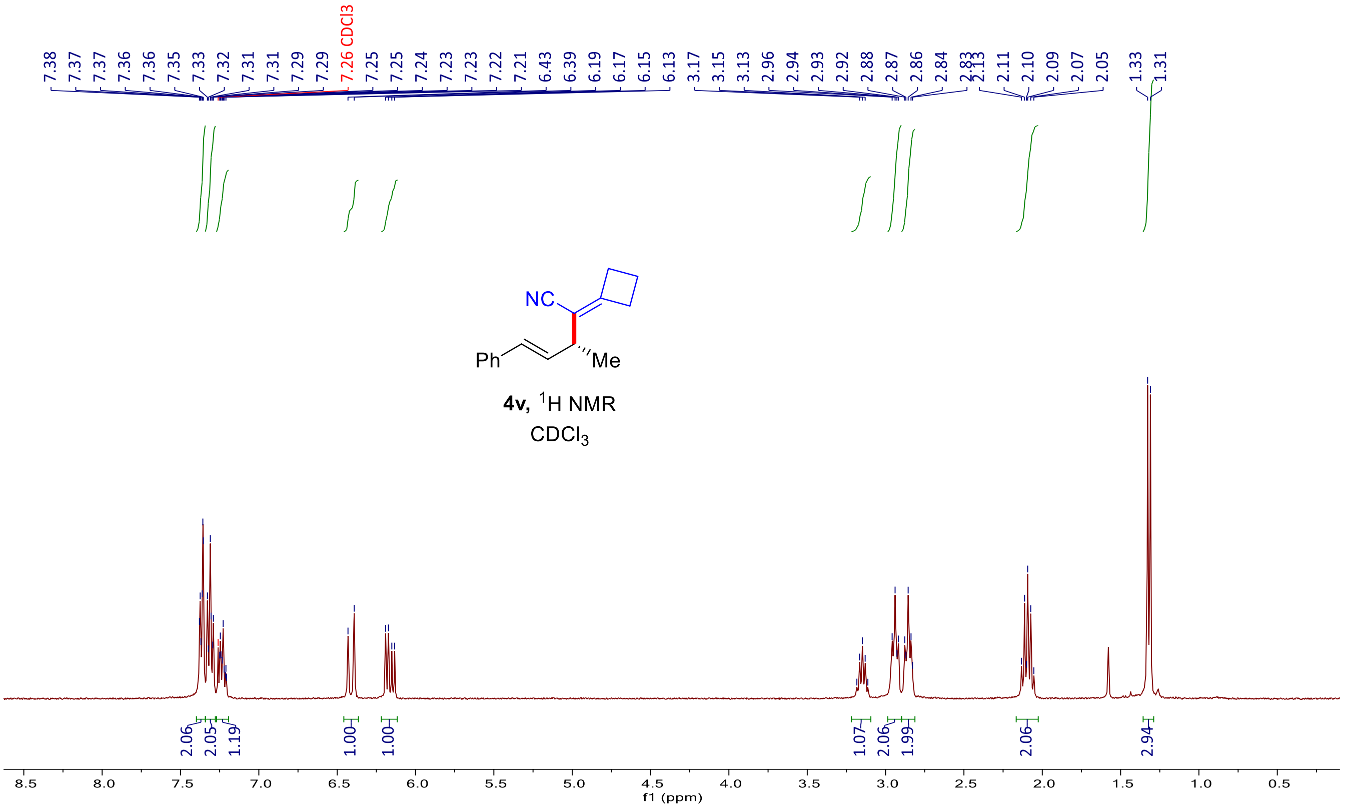

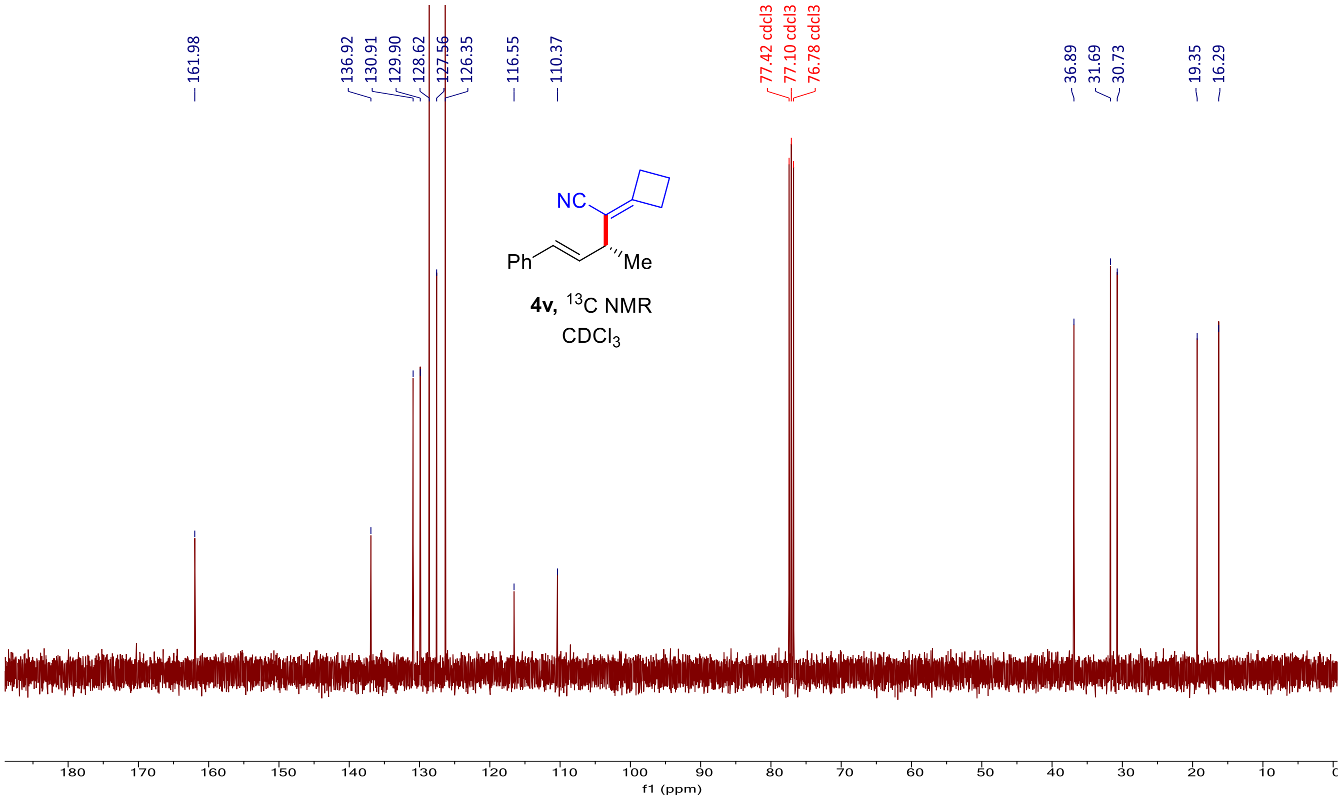

Supplementary Figure 27. ^{1}H NMR and ^{13}C NMR spectra of compound **4v**

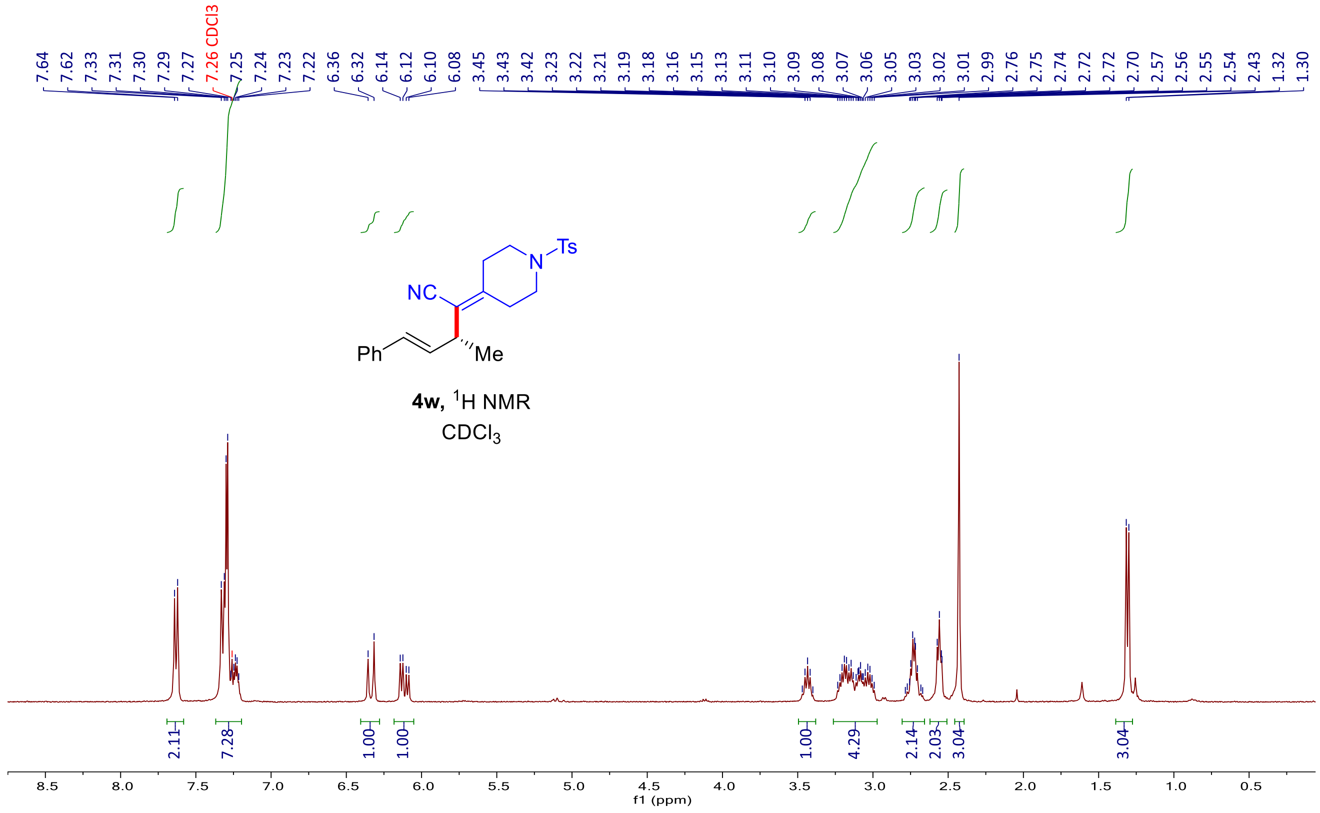

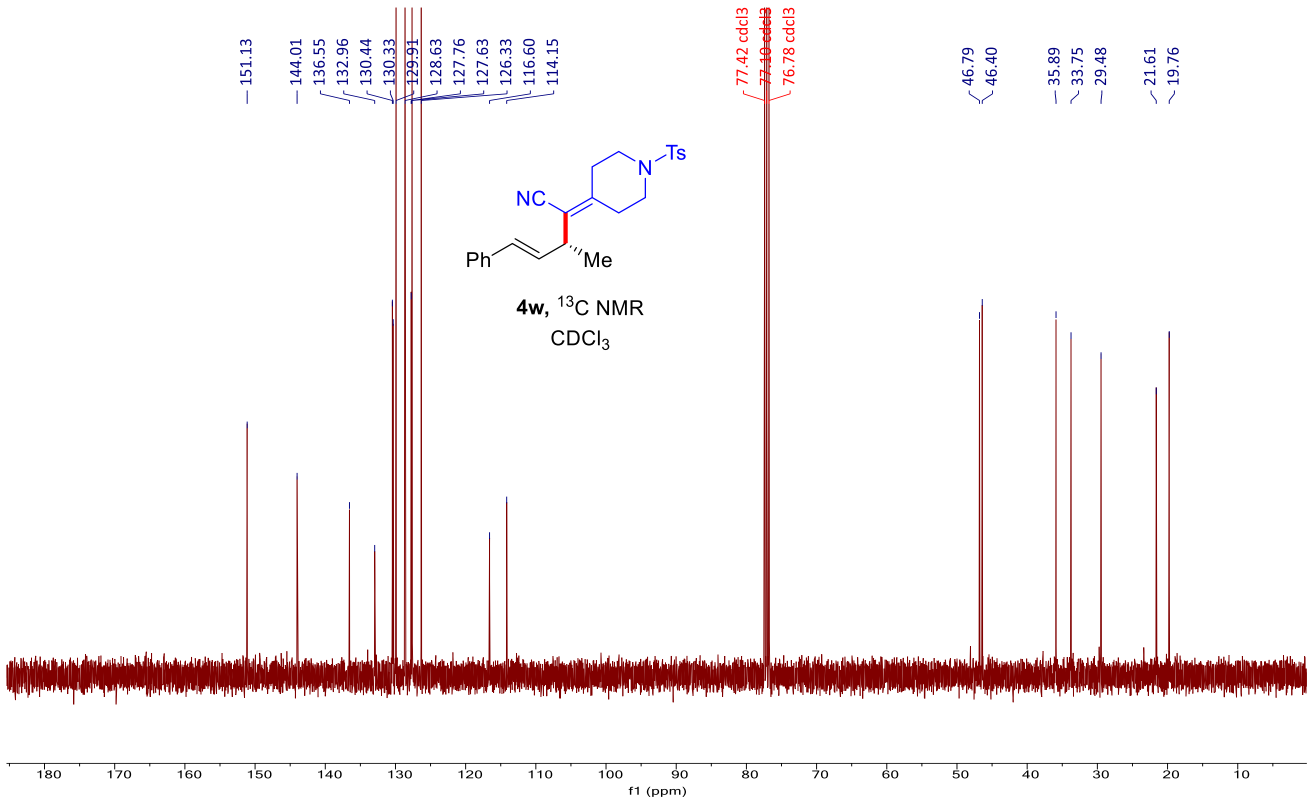

Supplementary Figure 28. ^{1}H NMR and ^{13}C NMR spectra of compound **4w**

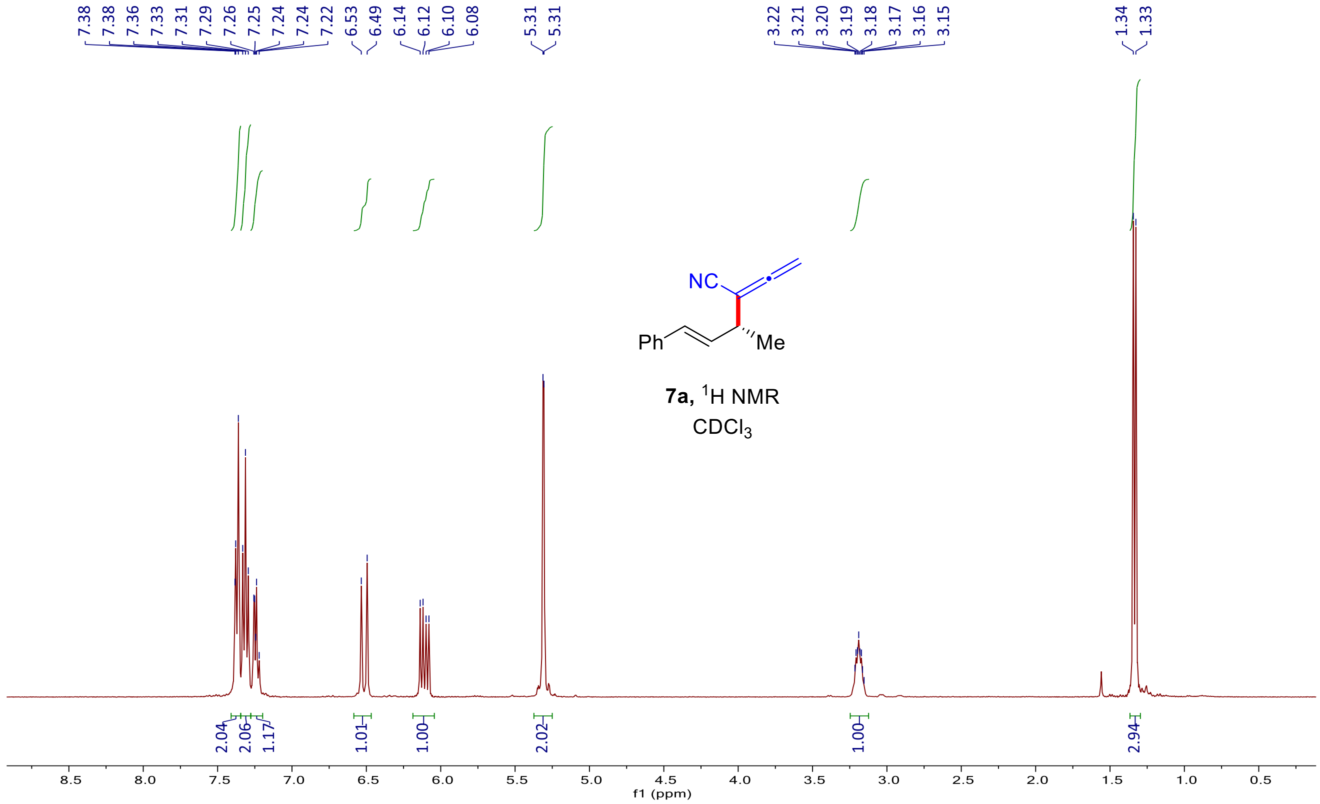

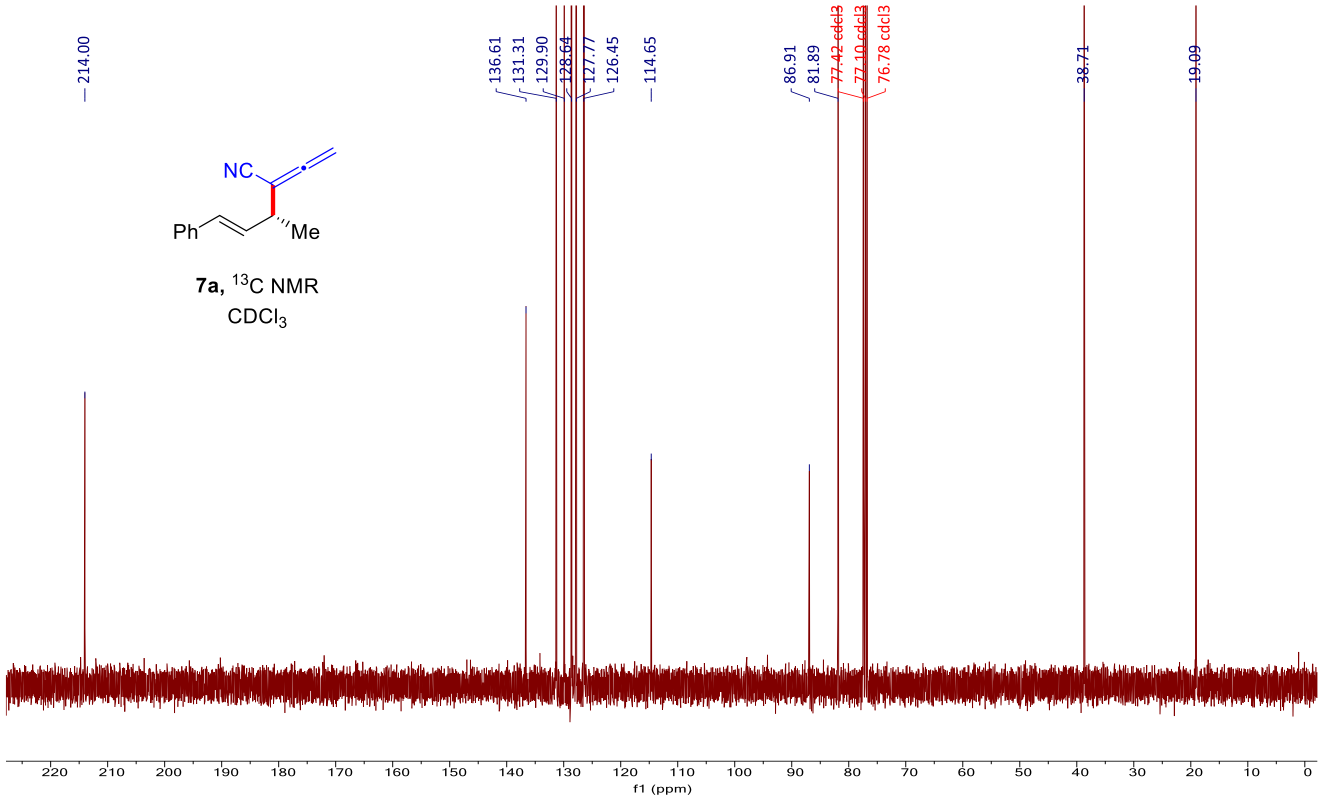

Supplementary Figure 29. ^{1}H NMR and ^{13}C NMR spectra of compound **7a**

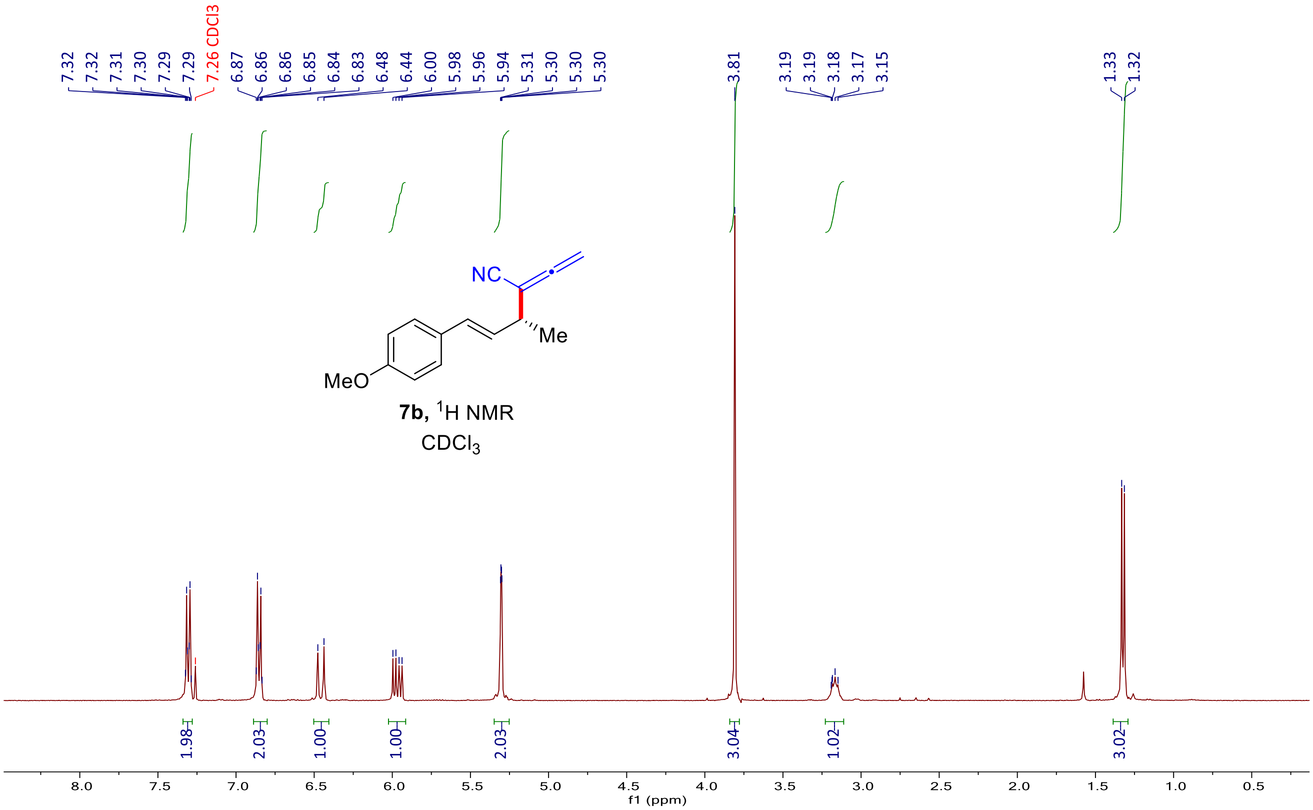

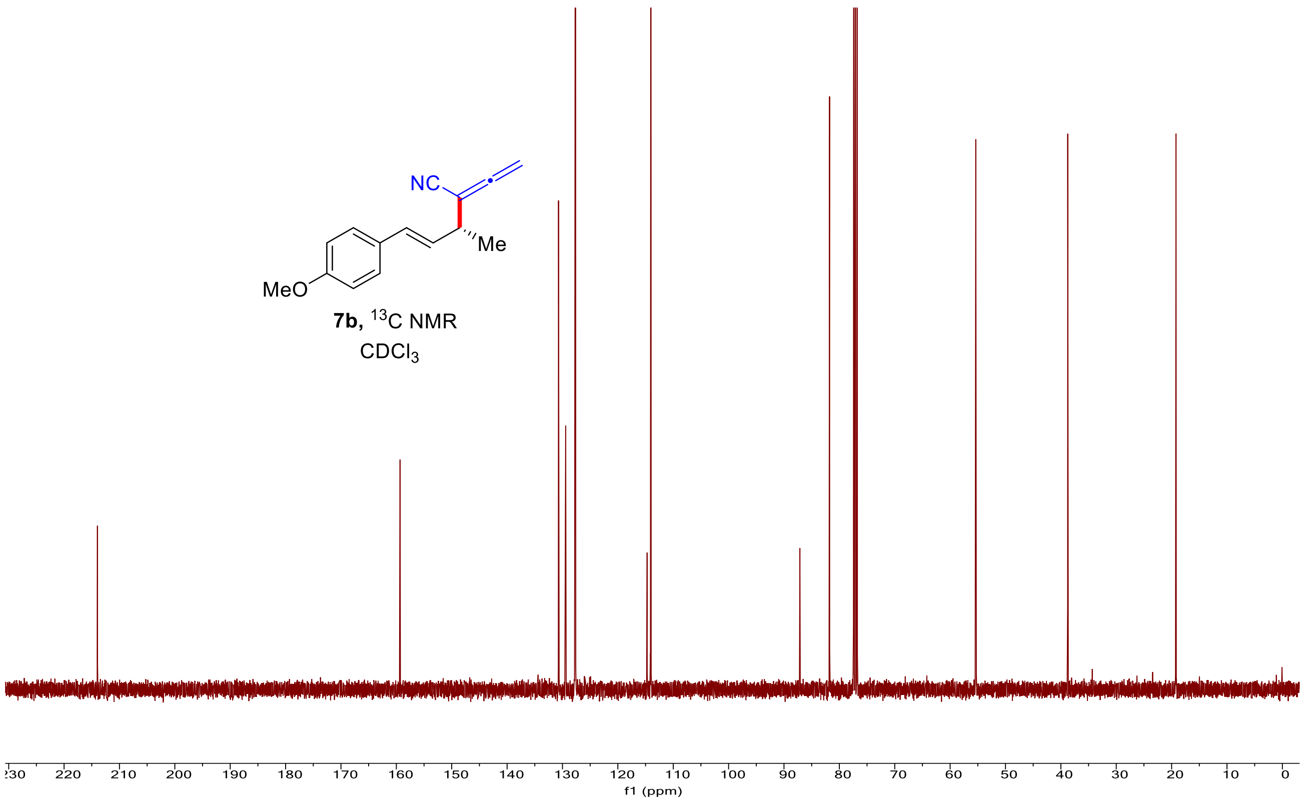

Supplementary Figure 30. ^{1}H NMR and ^{13}C NMR spectra of compound **7b**

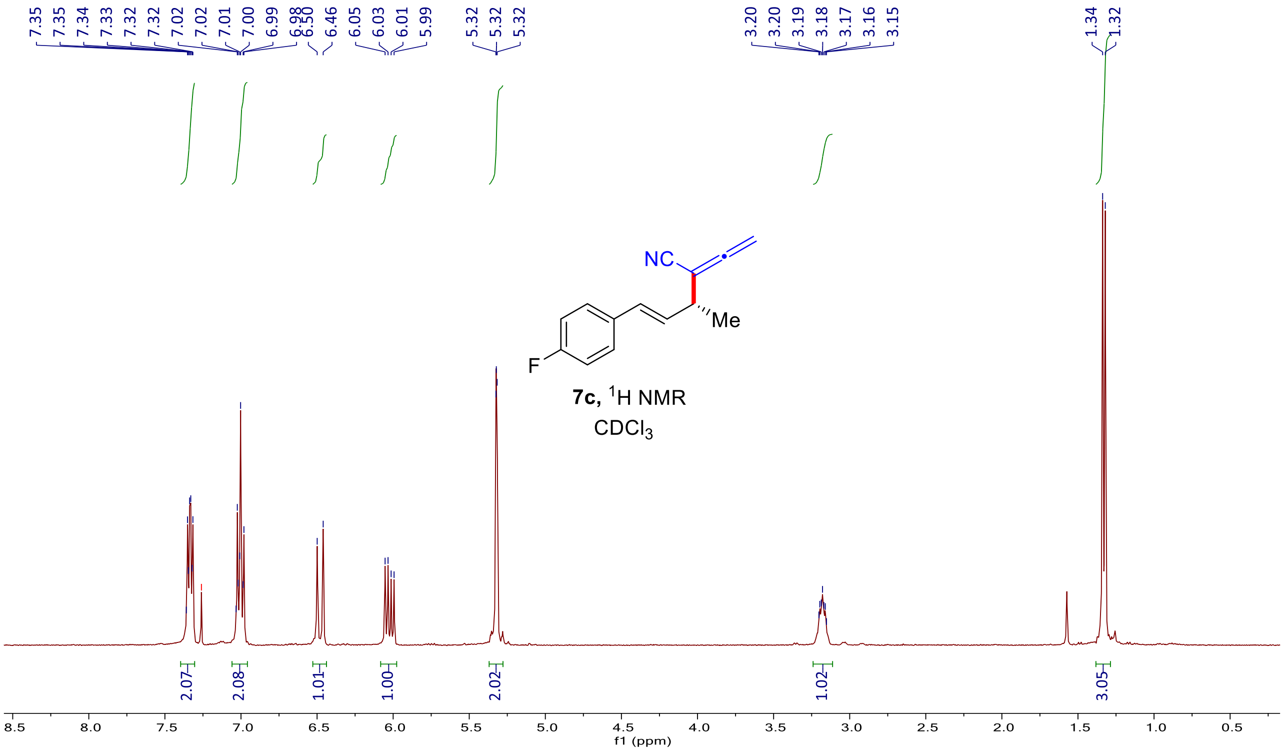

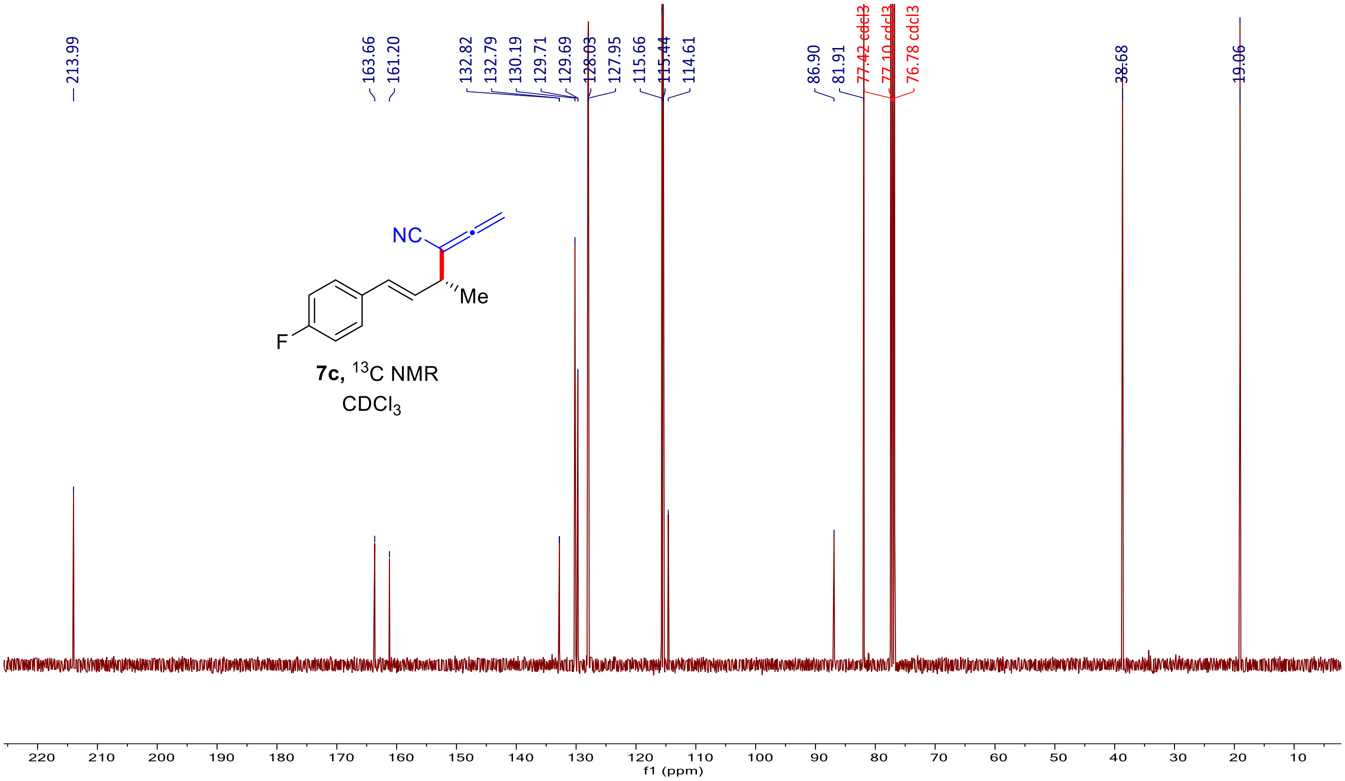

Supplementary Figure 31. ^{1}H NMR and ^{13}C NMR spectra of compound **7c**

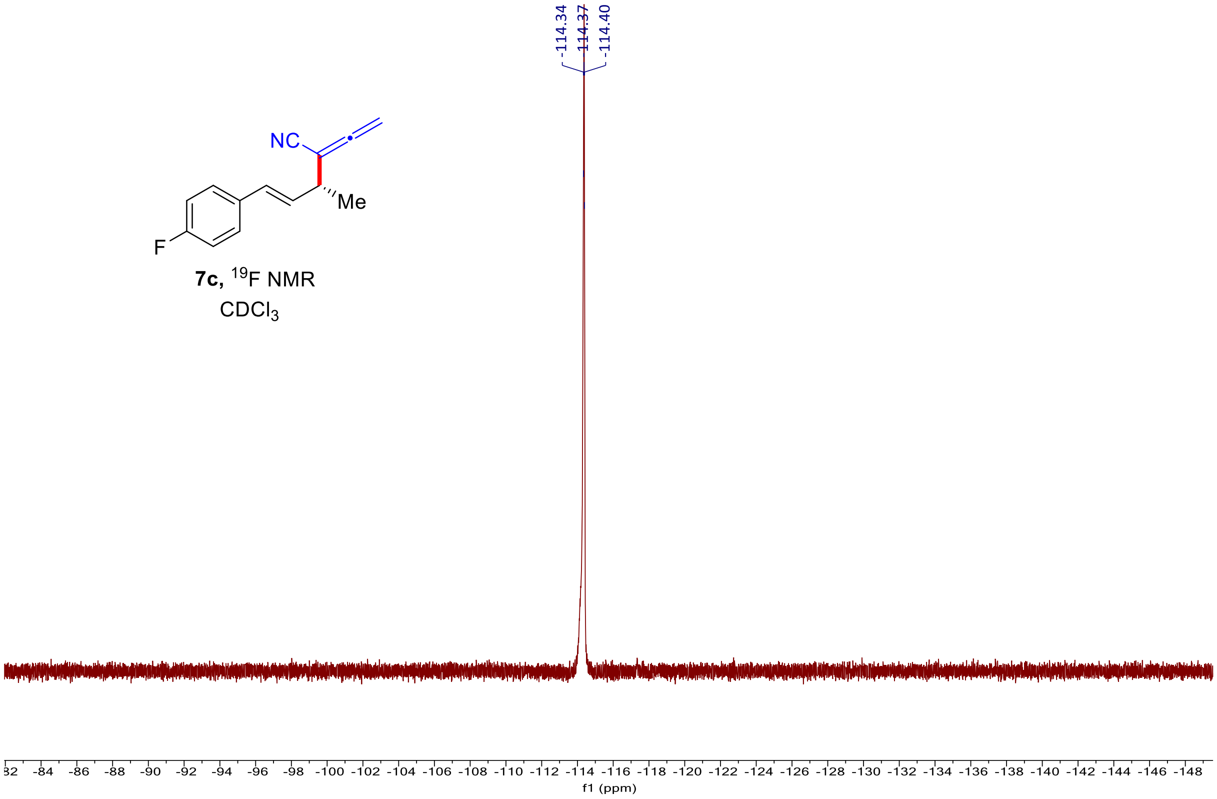

Supplementary Figure 32. ^{19}F NMR spectra of compound **7c**

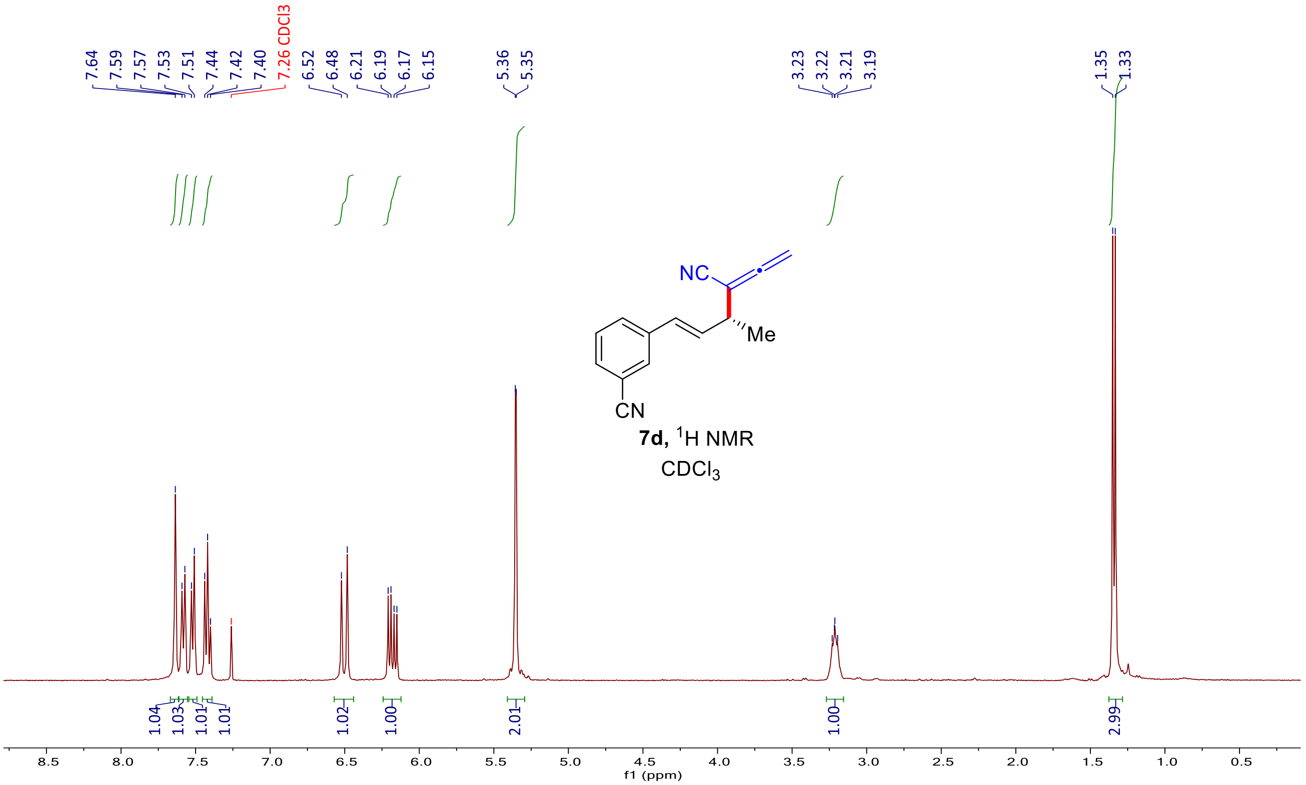

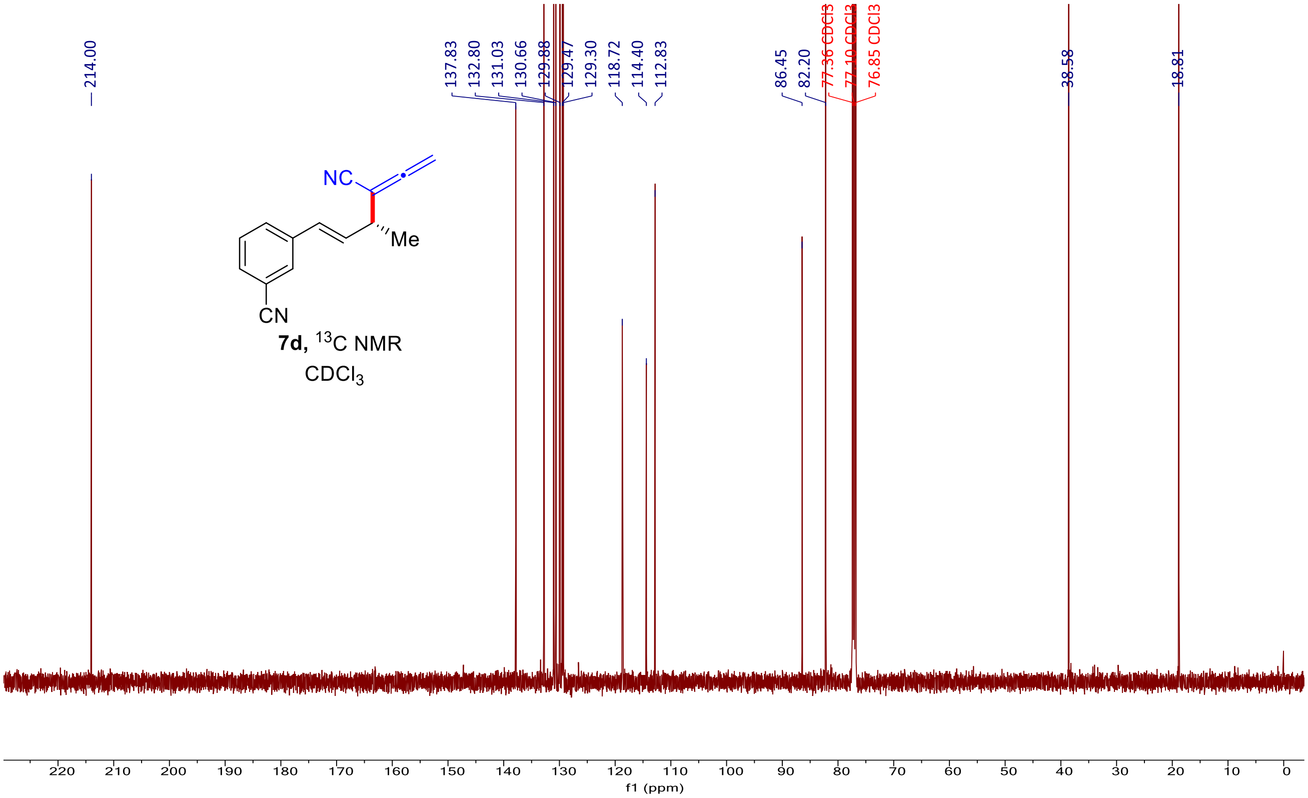

Supplementary Figure 33. ^{1}H NMR and ^{13}C NMR spectra of compound **7d**

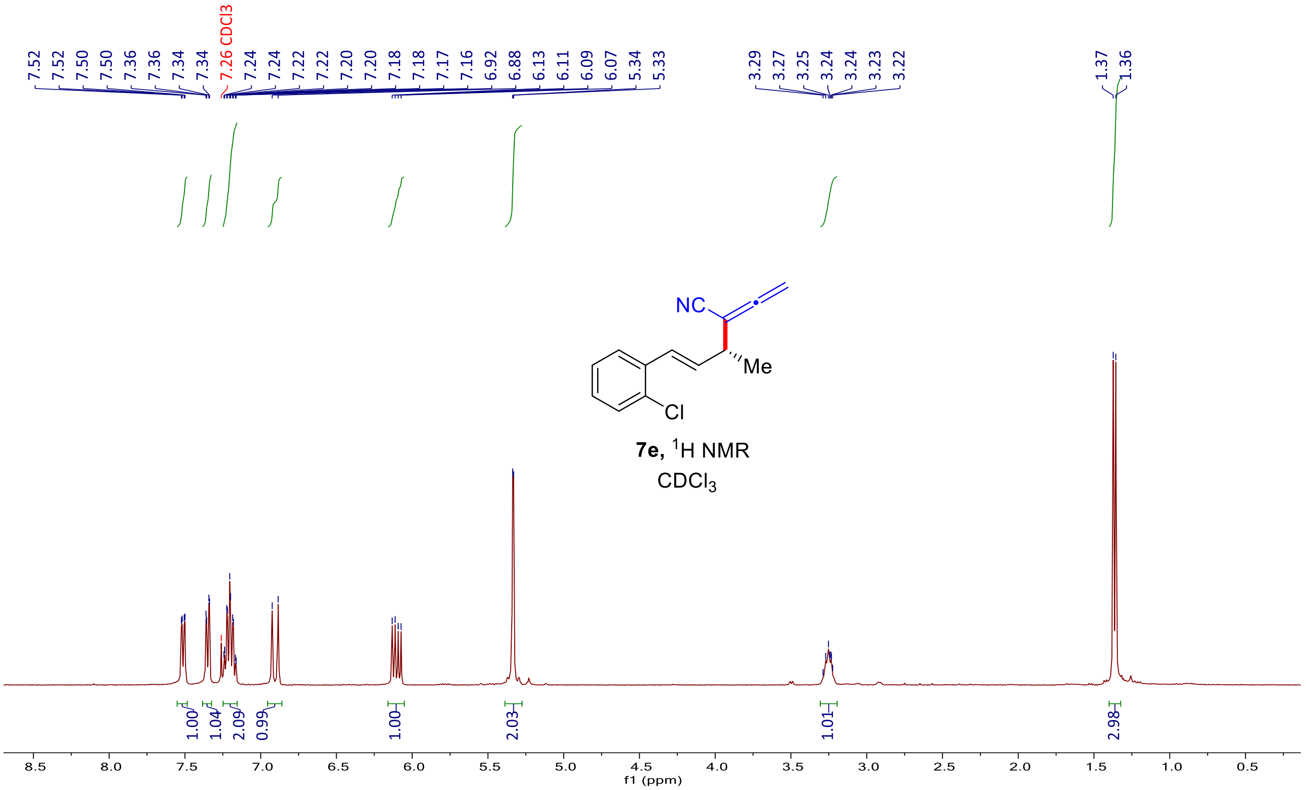

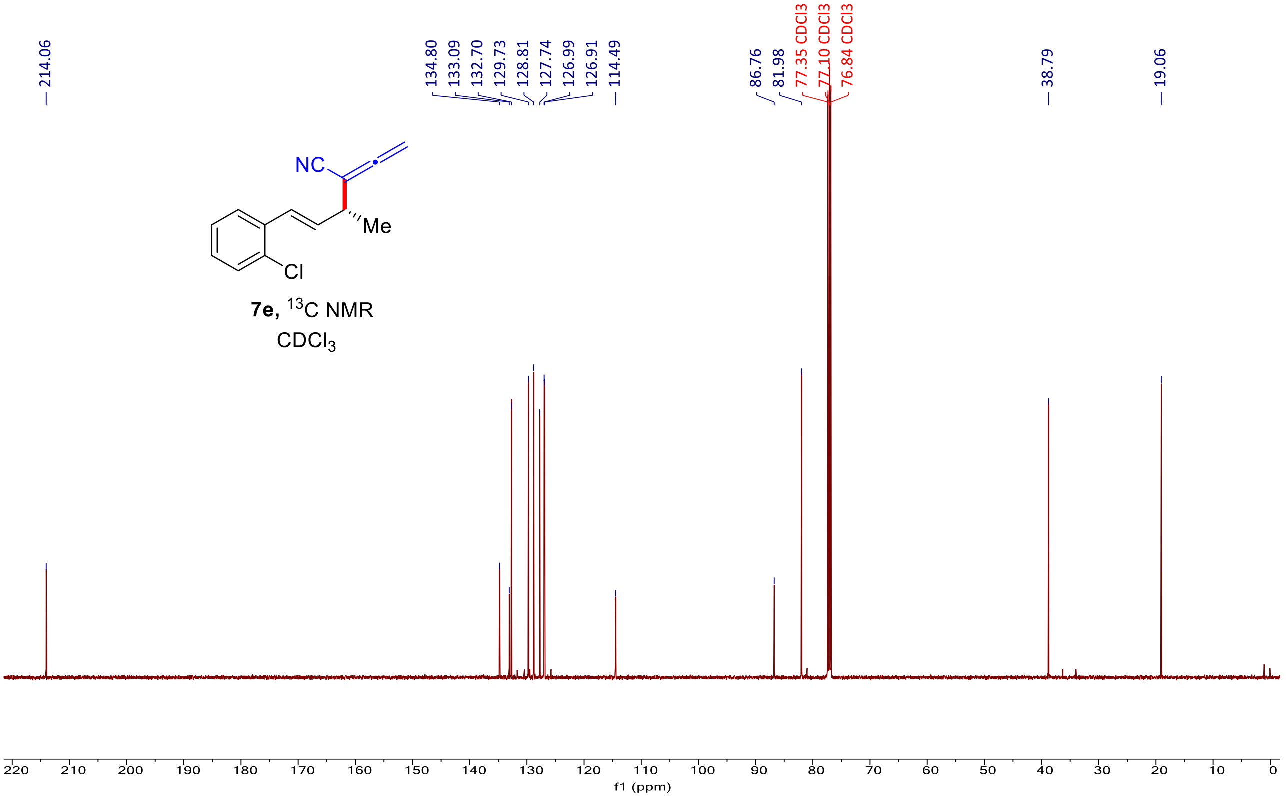

Supplementary Figure 34. ^{1}H NMR and ^{13}C NMR spectra of compound **7e**

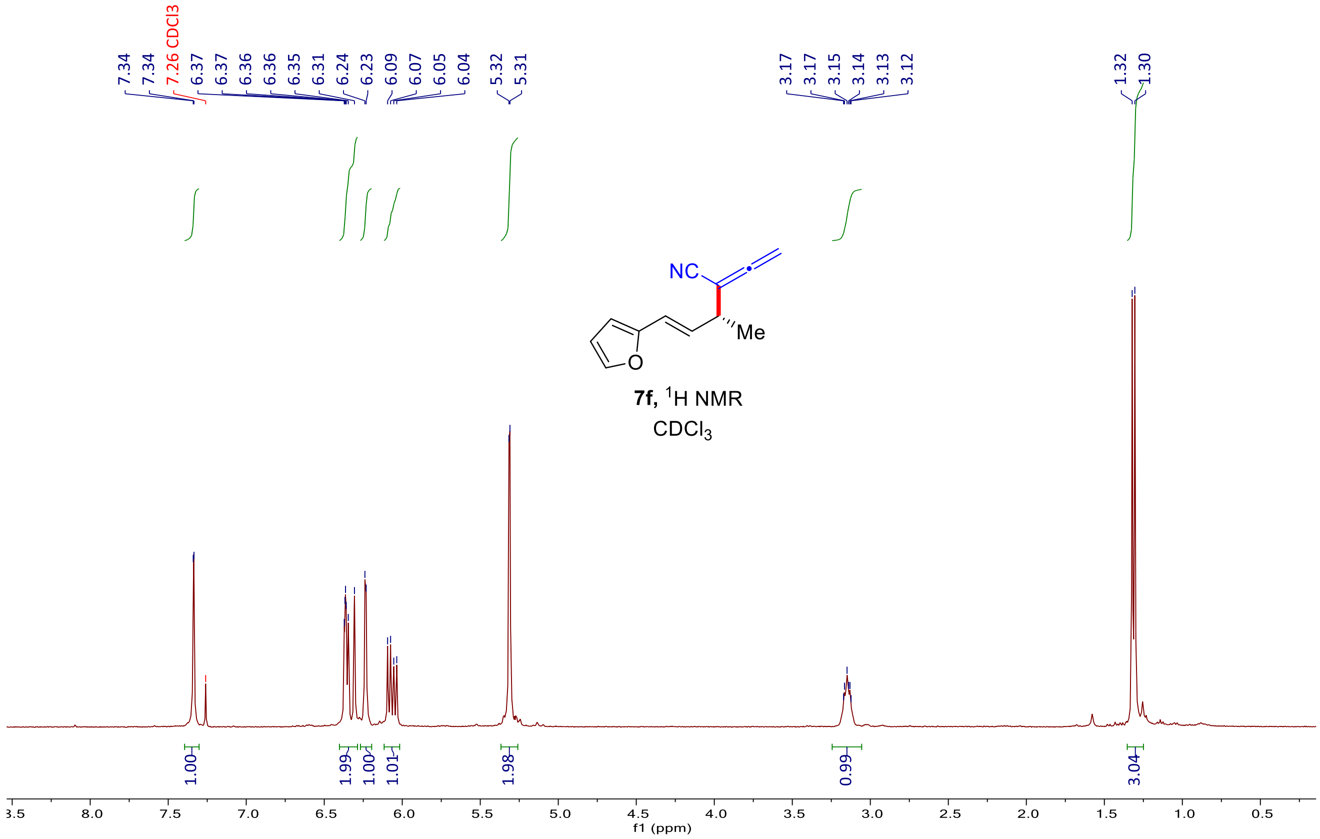

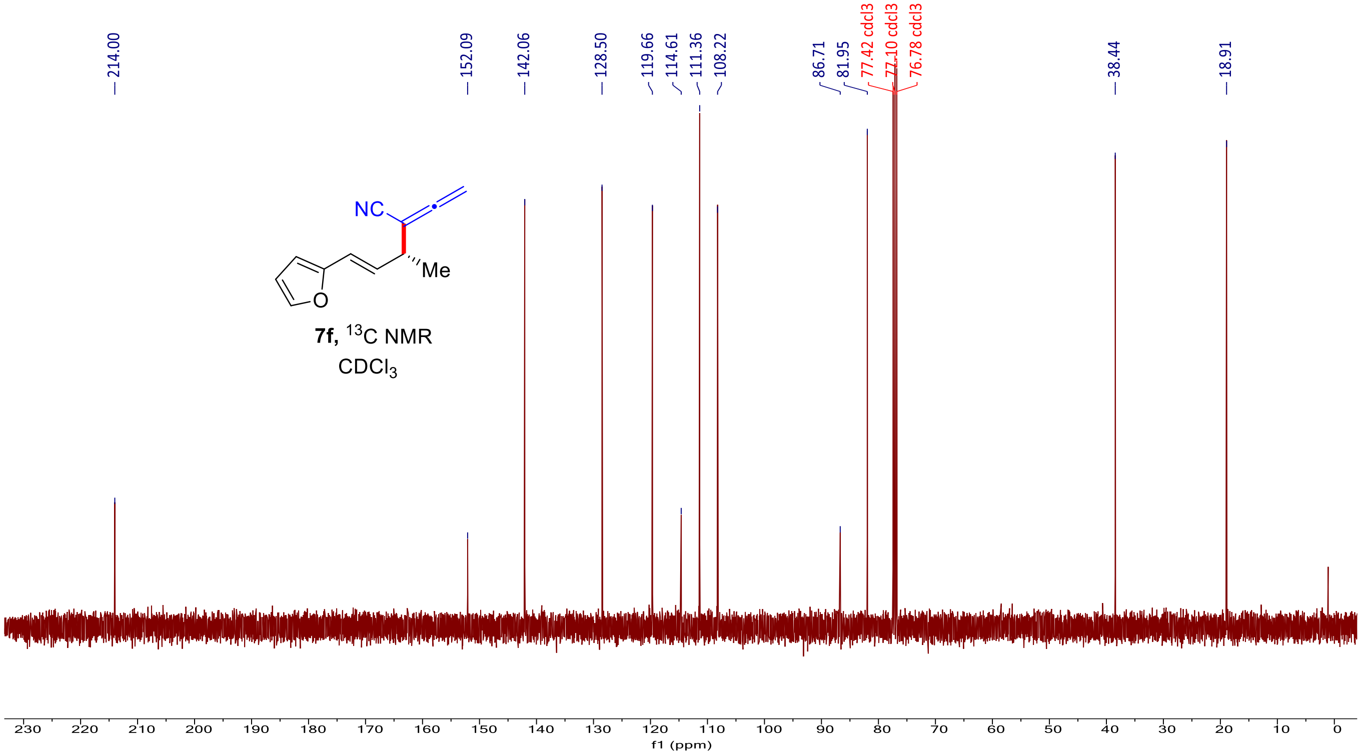

Supplementary Figure 35. ^{1}H NMR and ^{13}C NMR spectra of compound **7f**

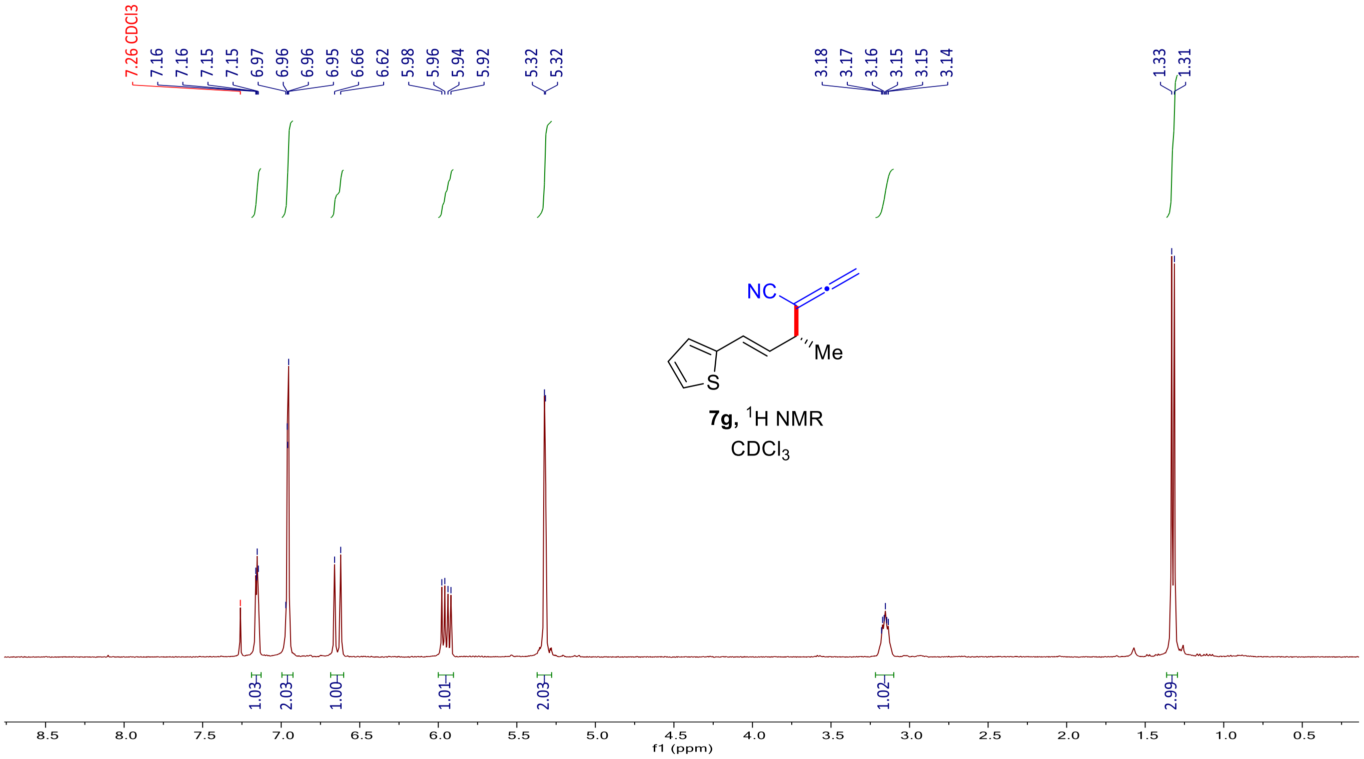

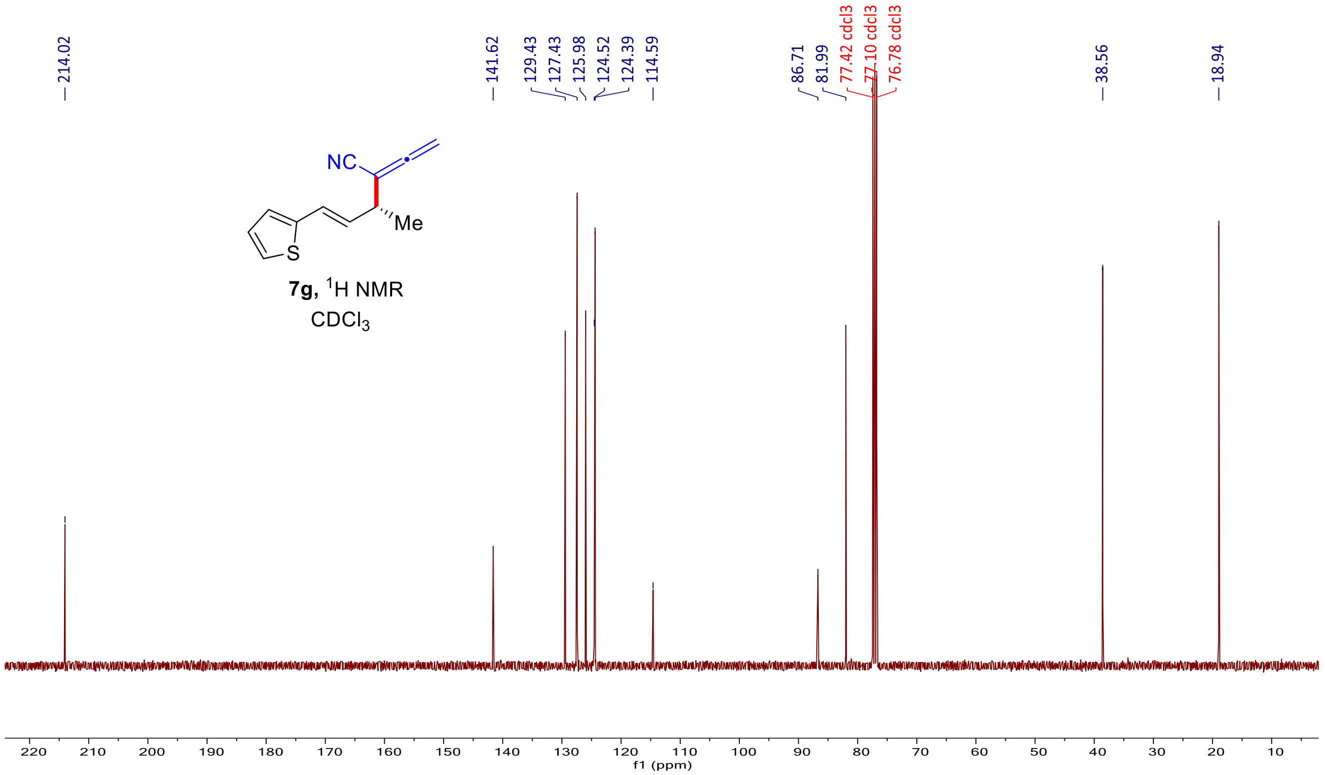

Supplementary Figure 36. ^{1}H NMR and ^{13}C NMR spectra of compound **7g**

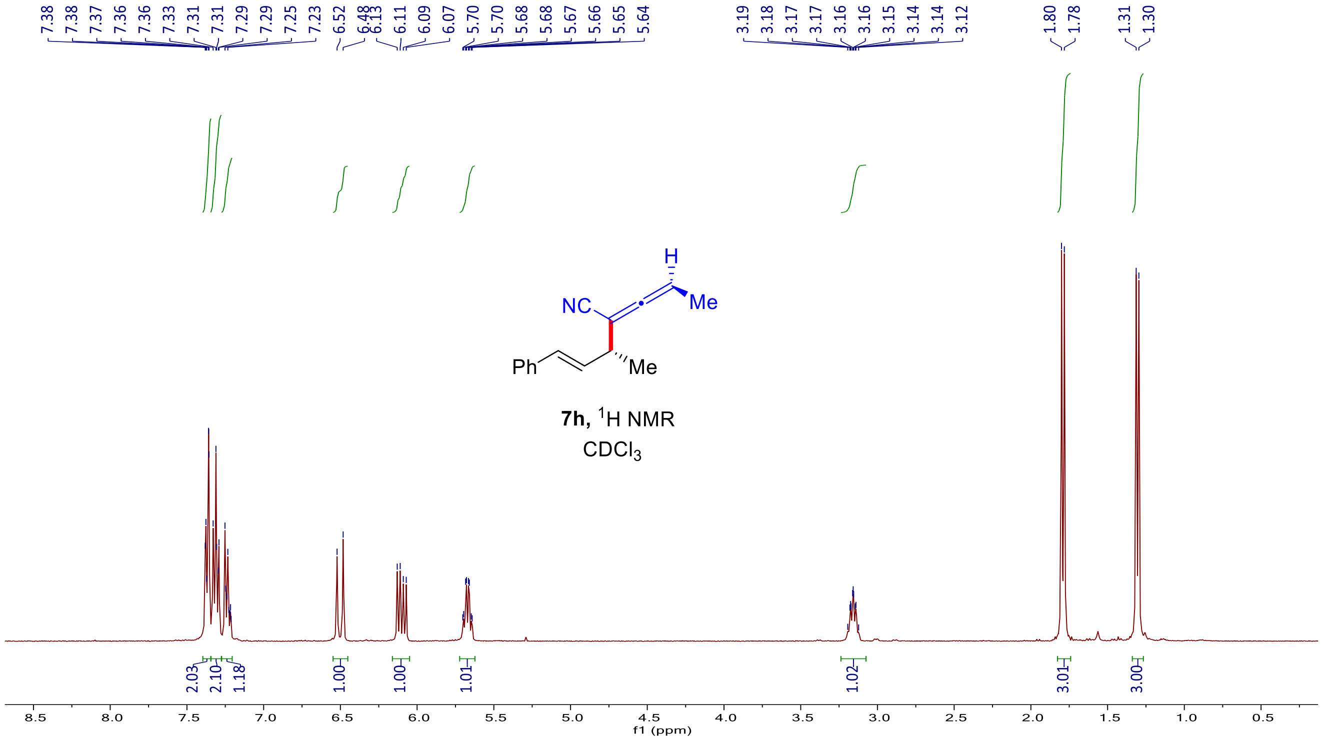

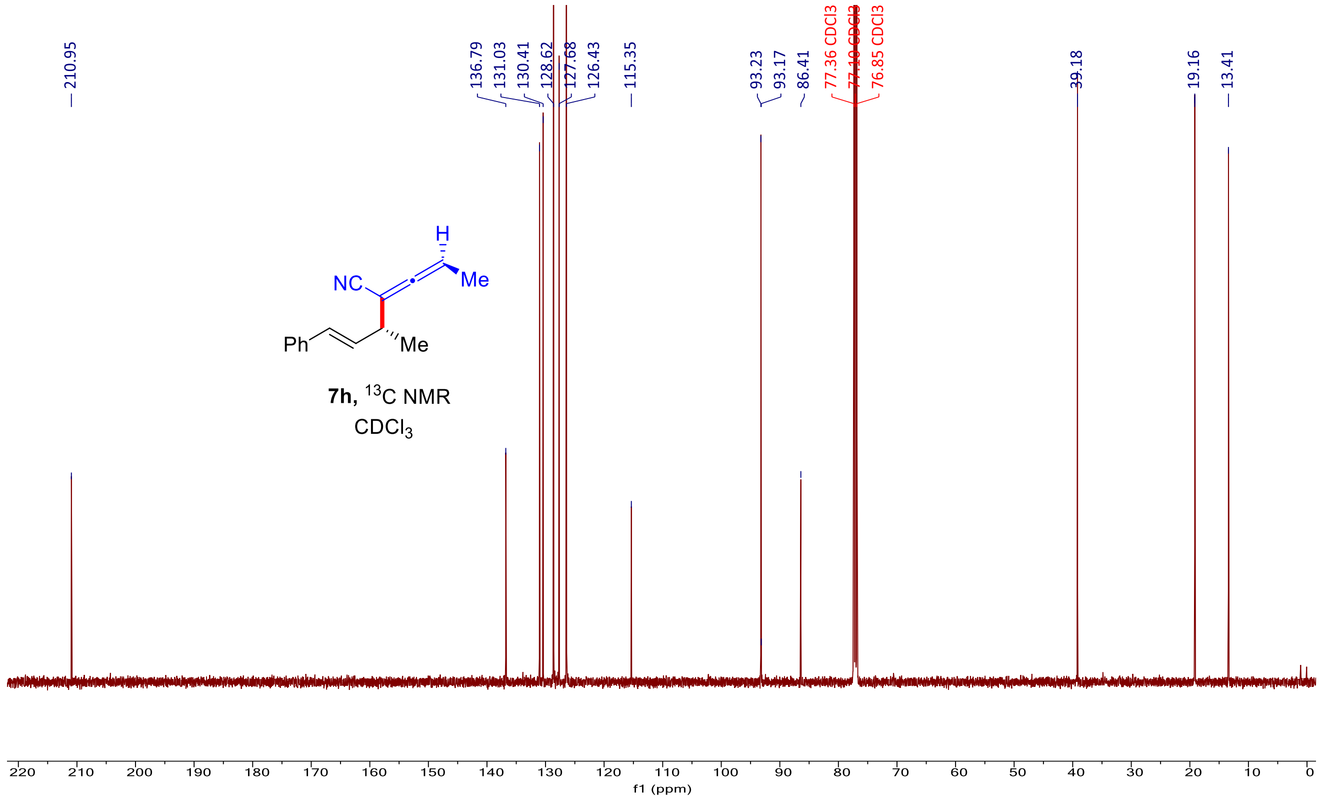

Supplementary Figure 37. ^{1}H NMR and ^{13}C NMR spectra of compound **7h**

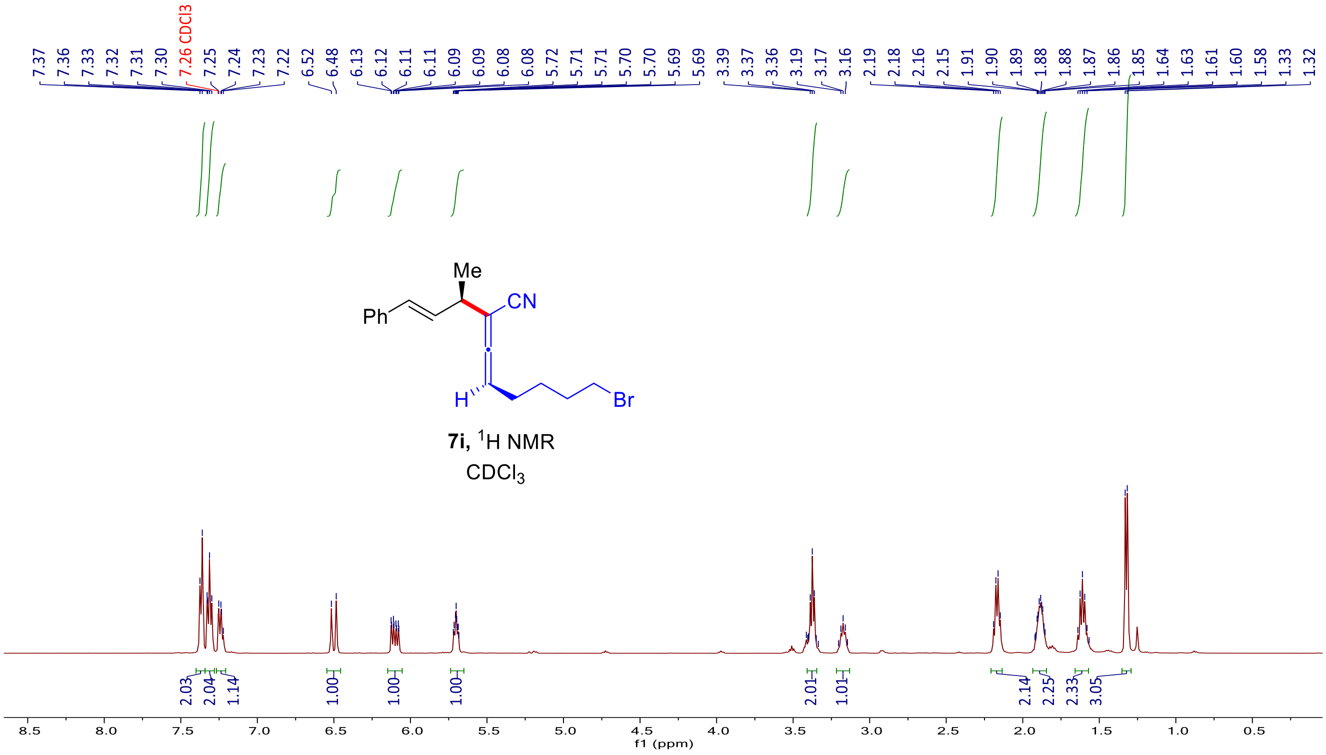

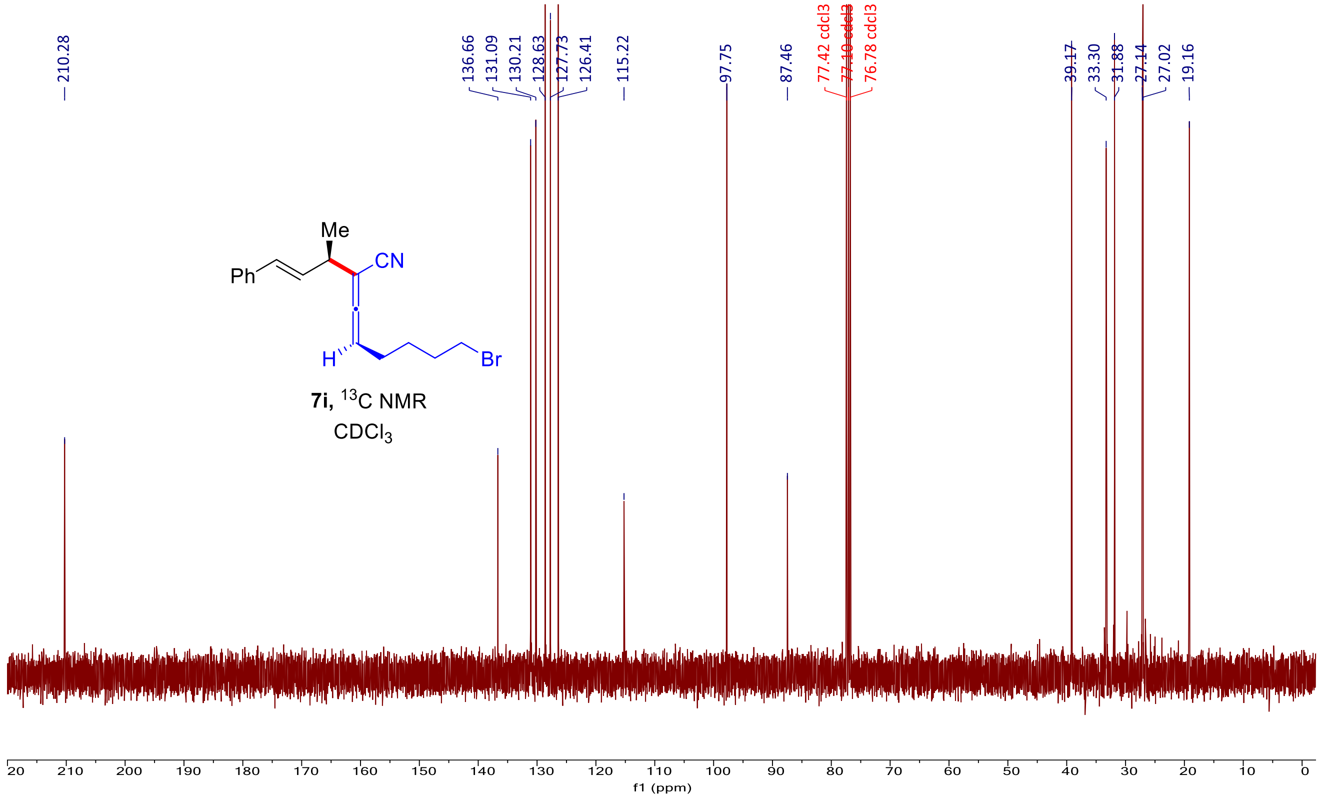

Supplementary Figure 38. ^{1}H NMR and ^{13}C NMR spectra of compound **7i**

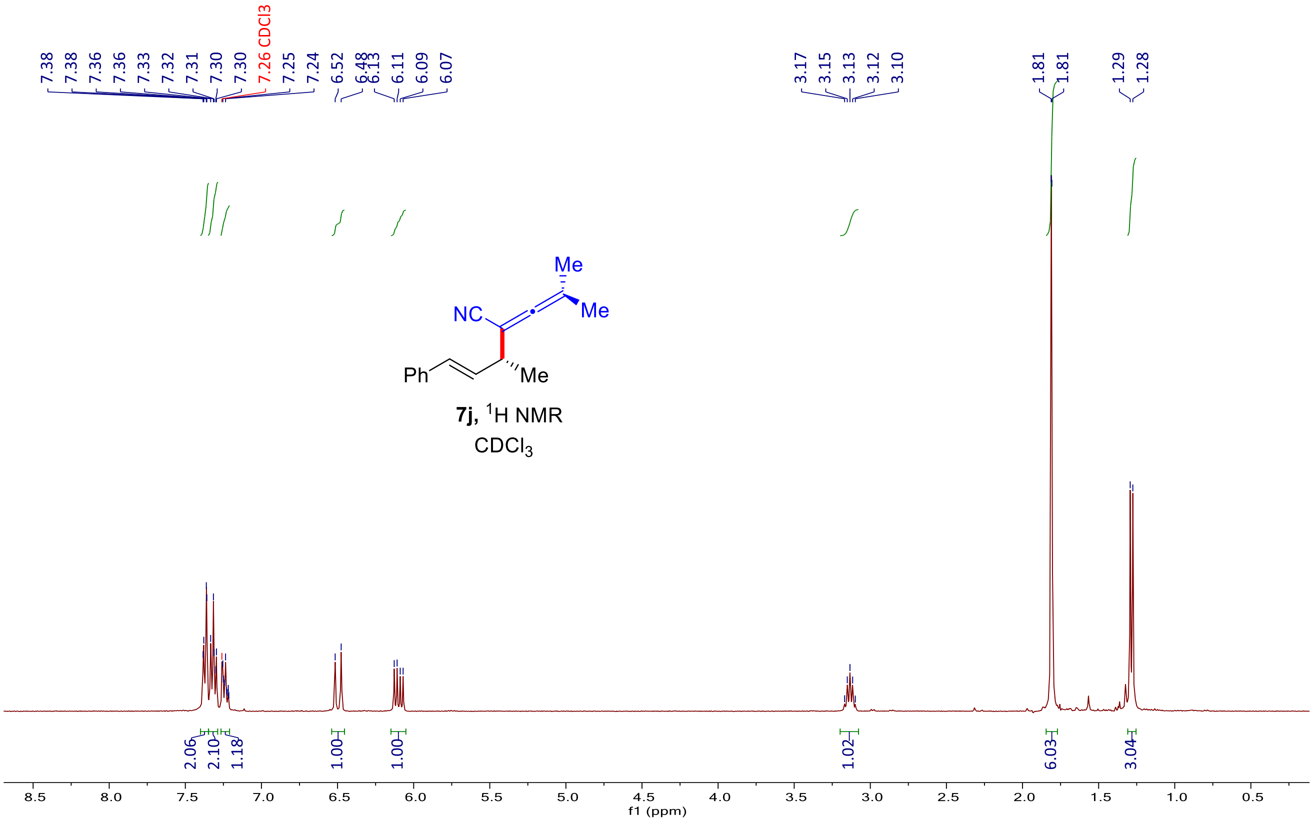

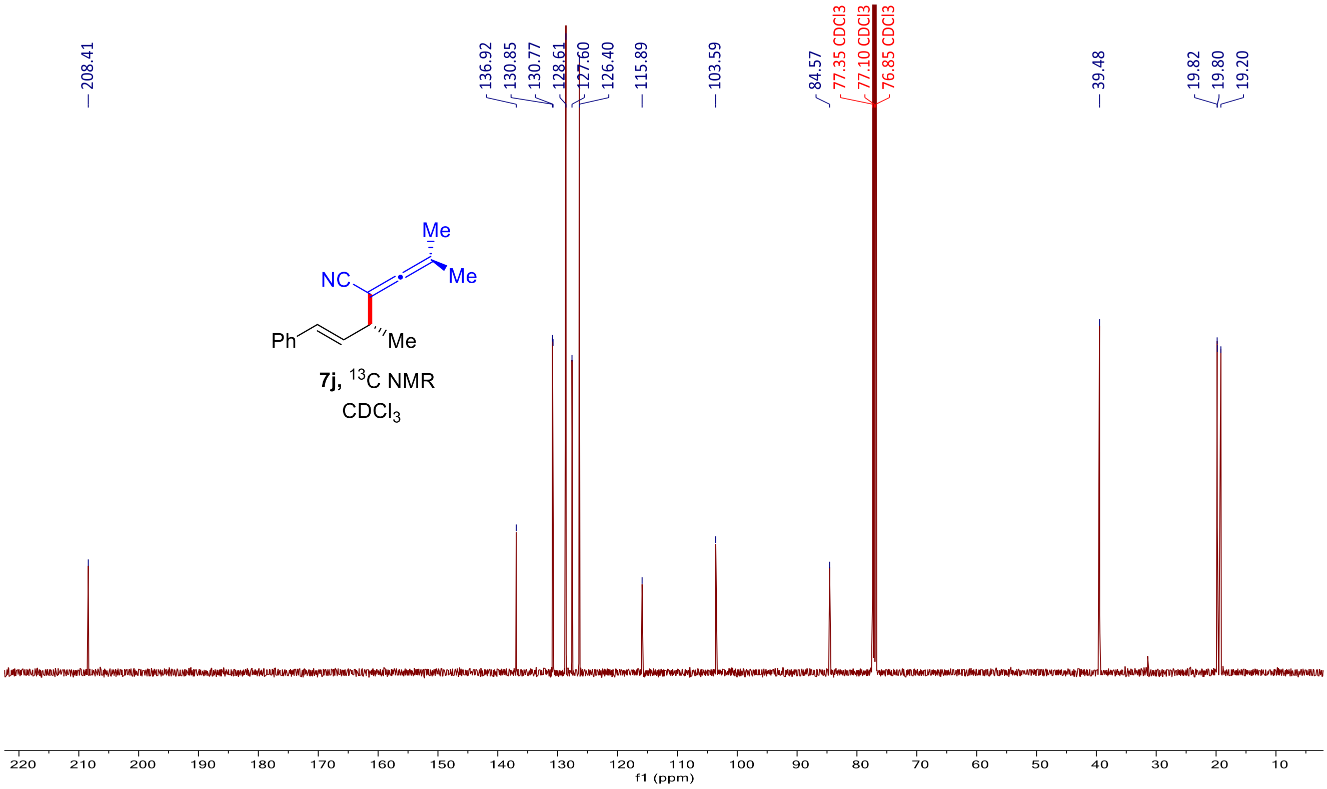

Supplementary Figure 39. ^{1}H NMR and ^{13}C NMR spectra of compound **7j**

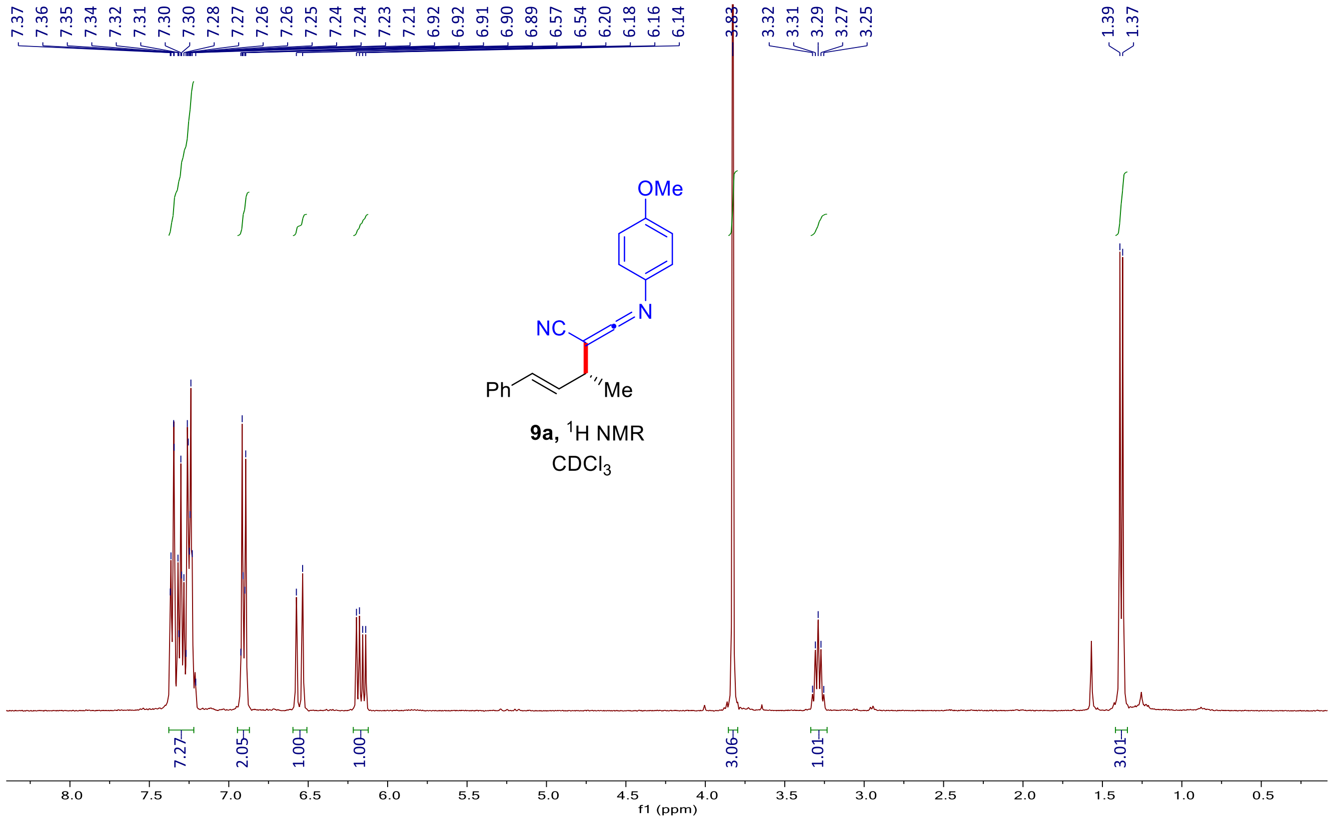

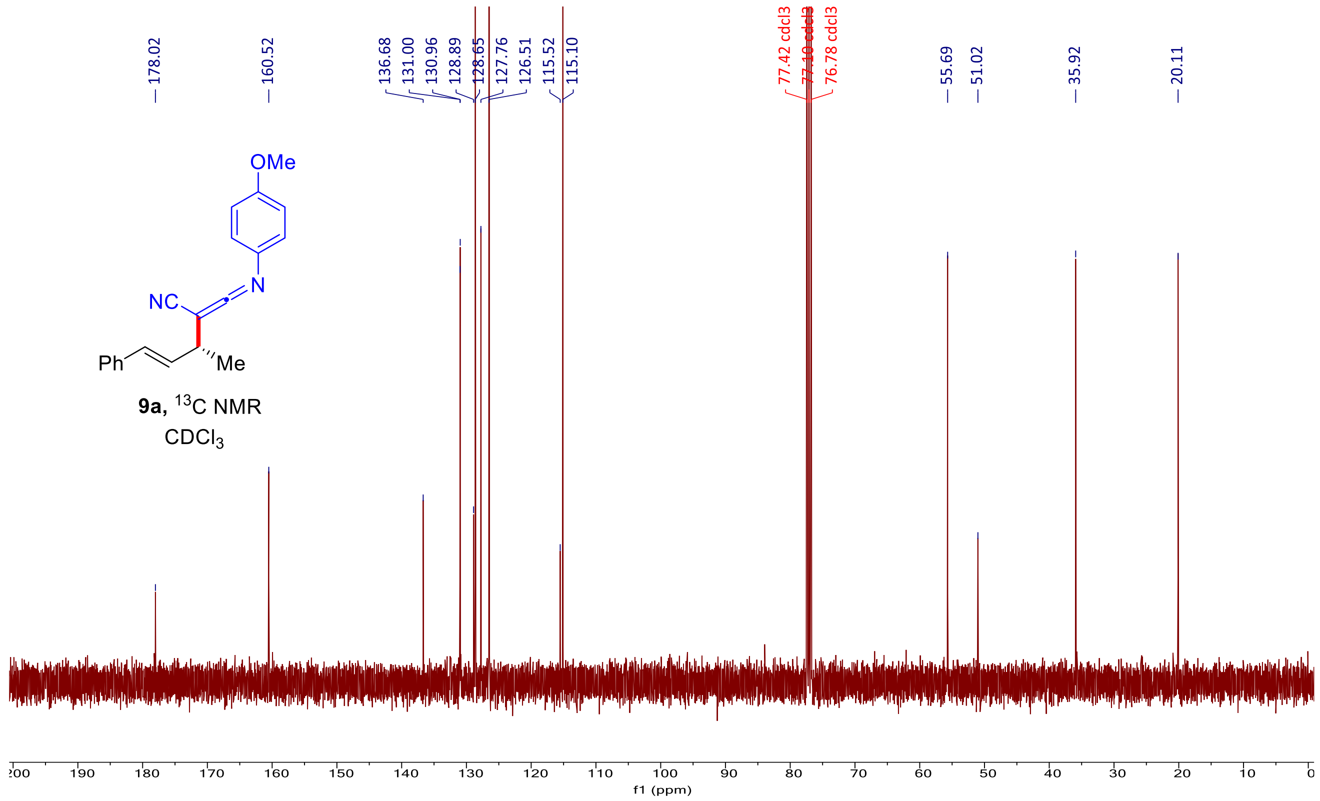

Supplementary Figure 40. ^{1}H NMR and ^{13}C NMR spectra of compound **9a**

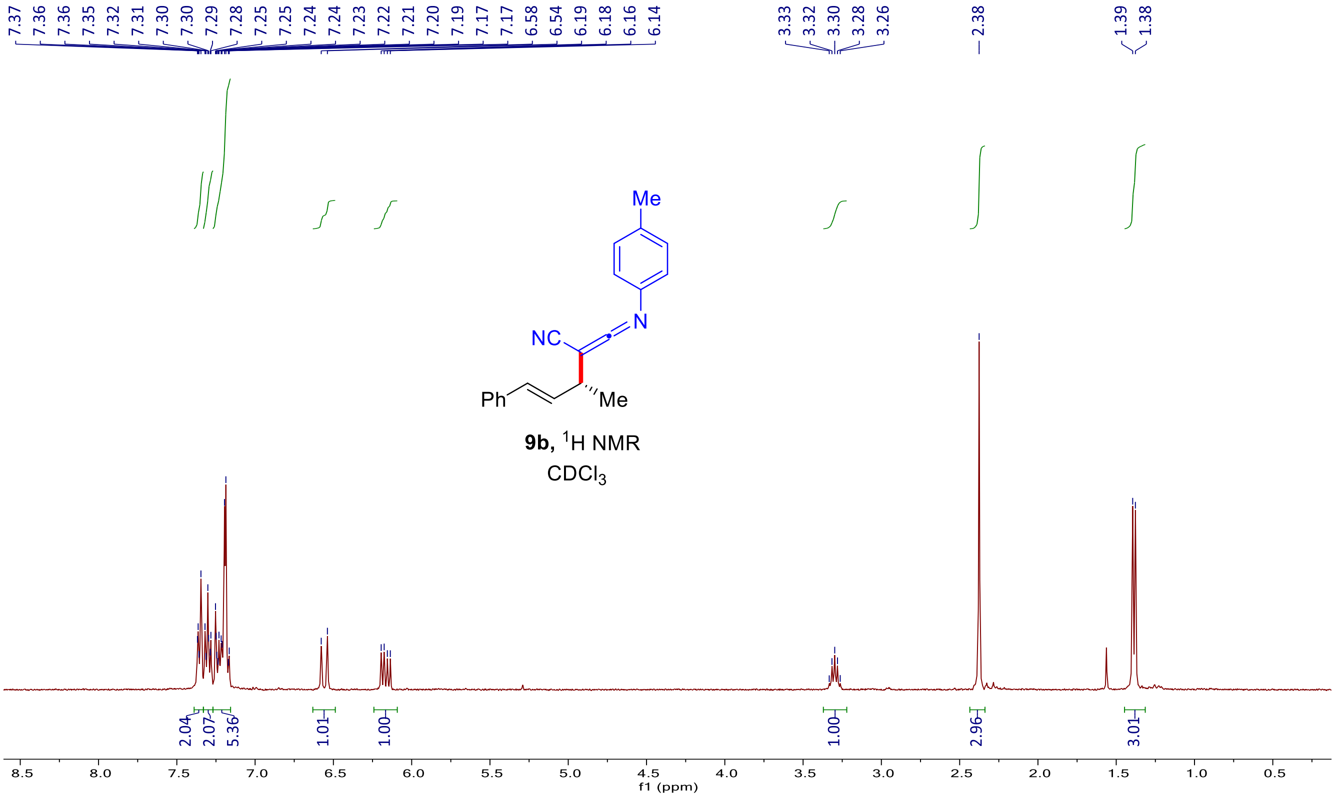

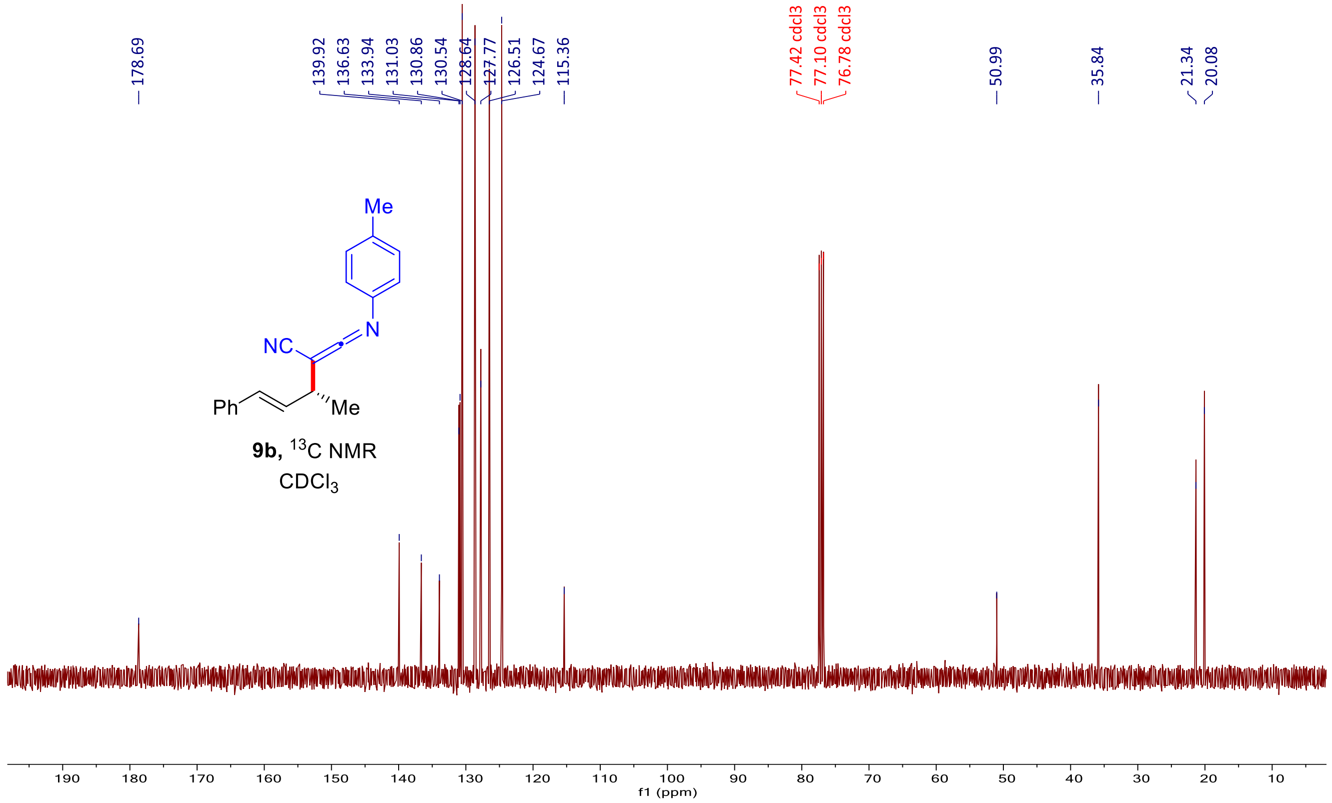

Supplementary Figure 41. ^{1}H NMR and ^{13}C NMR spectra of compound **9b**

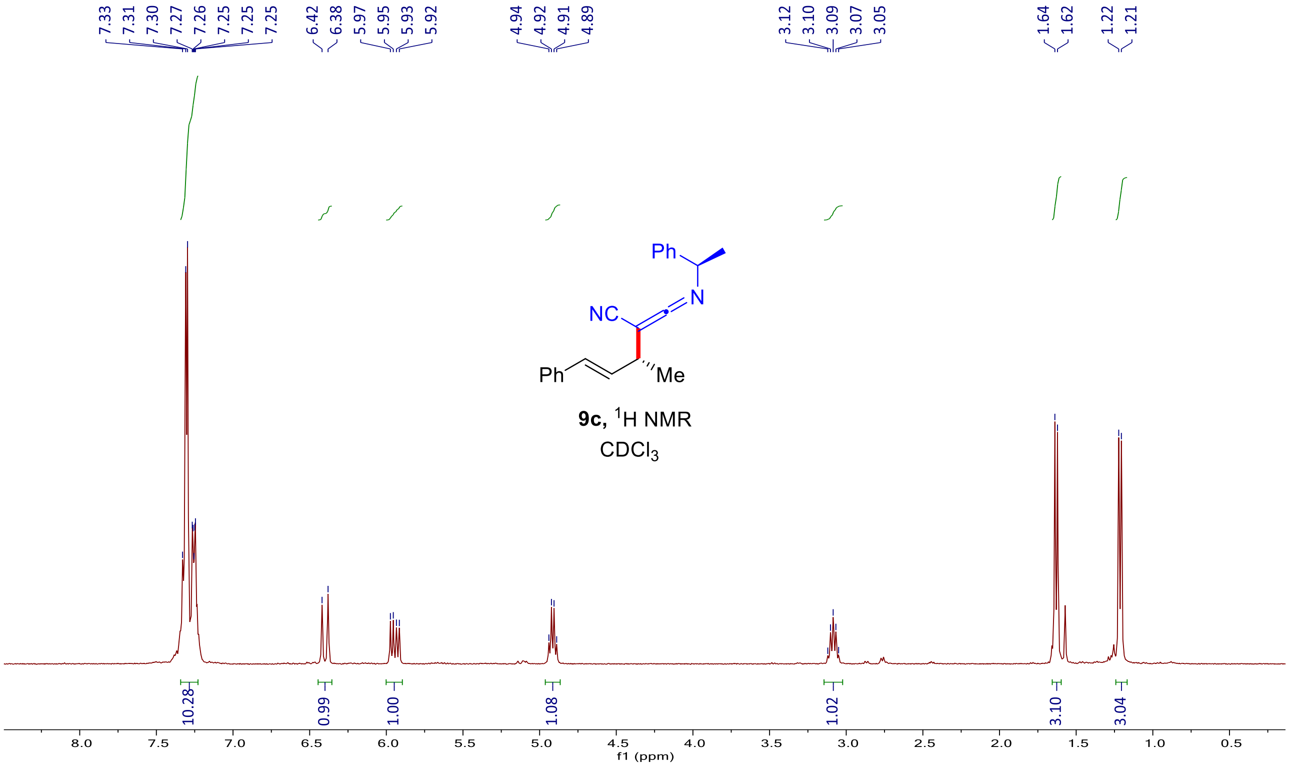

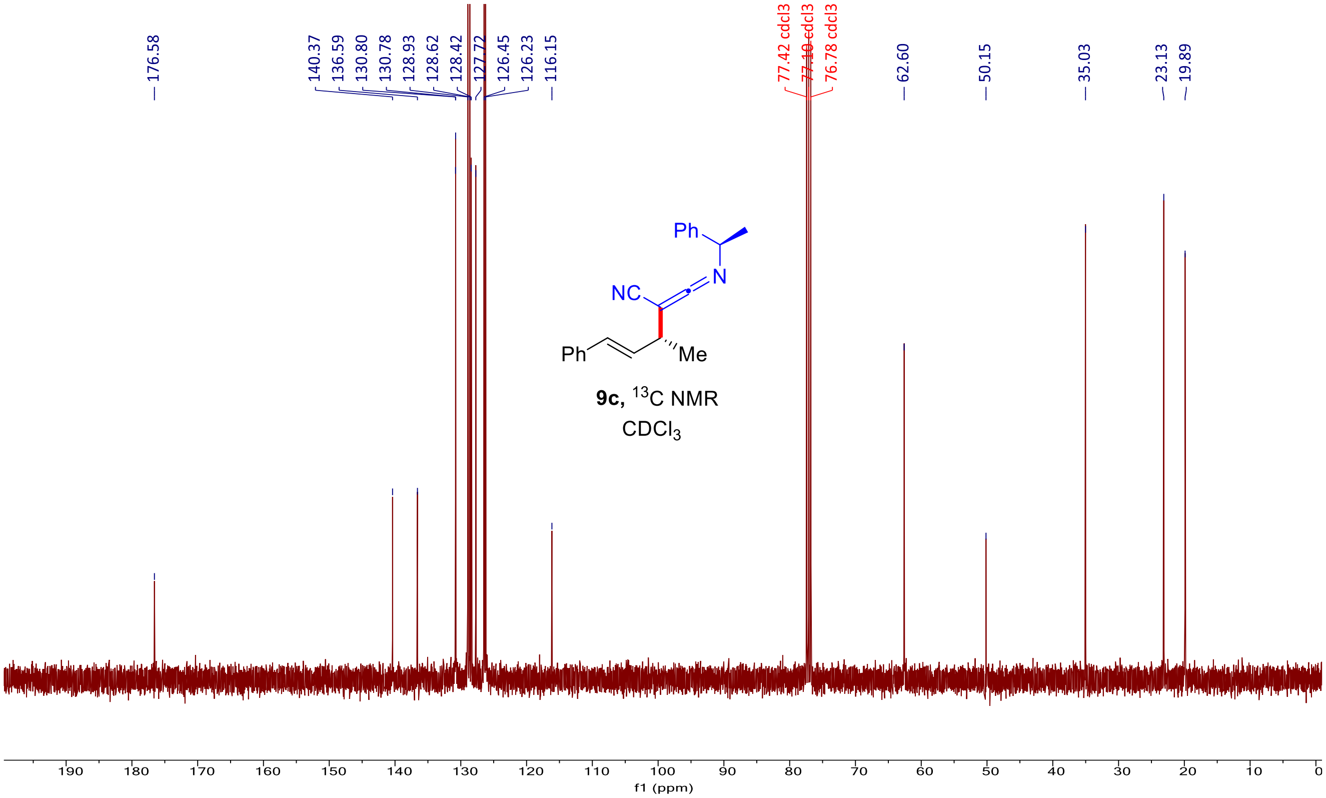

Supplementary Figure 42. ^{1}H NMR and ^{13}C NMR spectra of compound **9c**

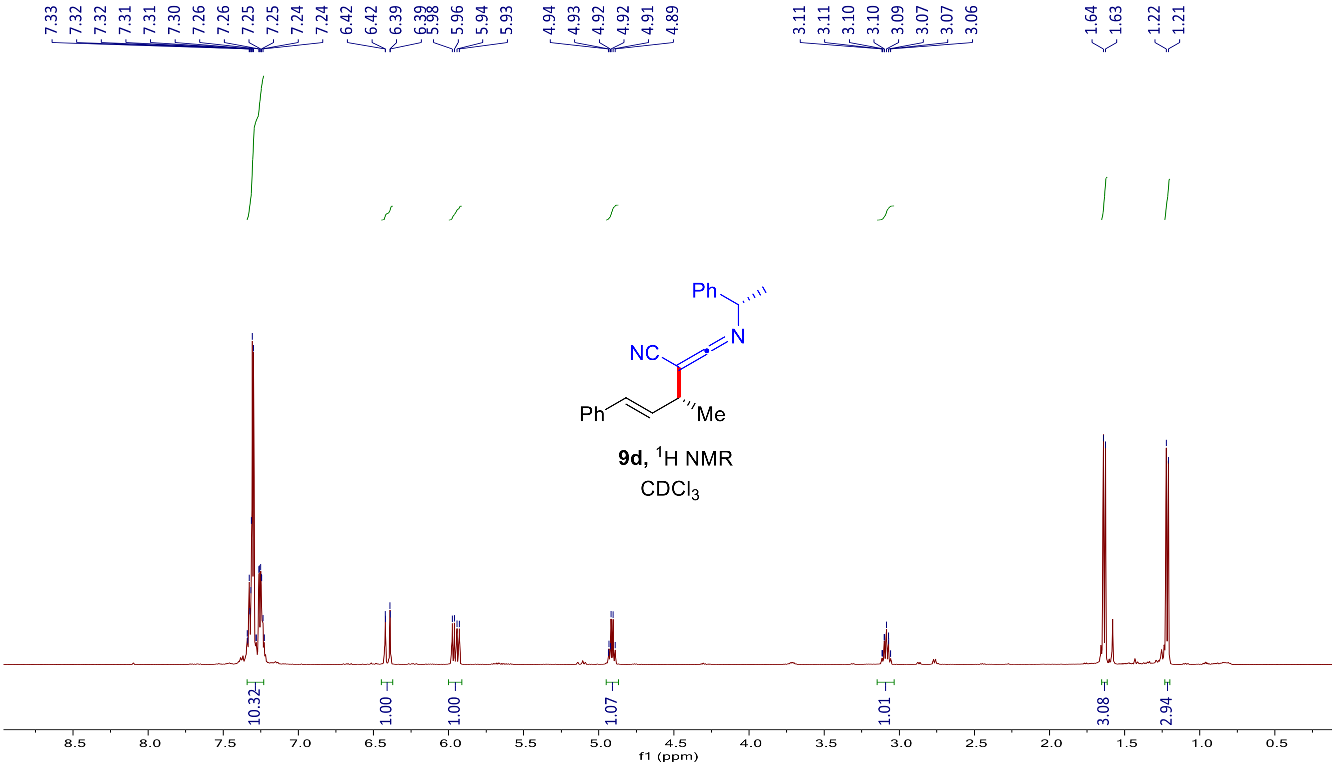

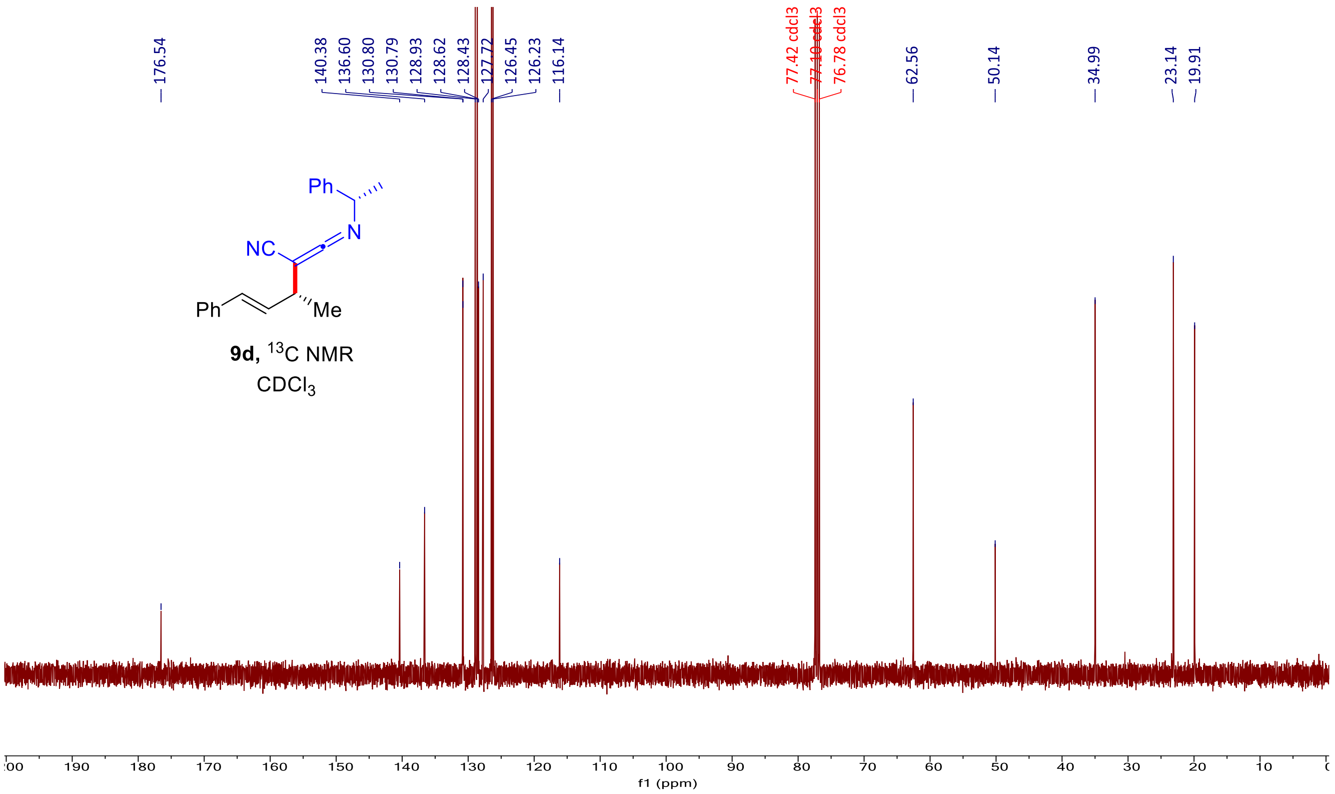

Supplementary Figure 43. ^{1}H NMR and ^{13}C NMR spectra of compound **9d**

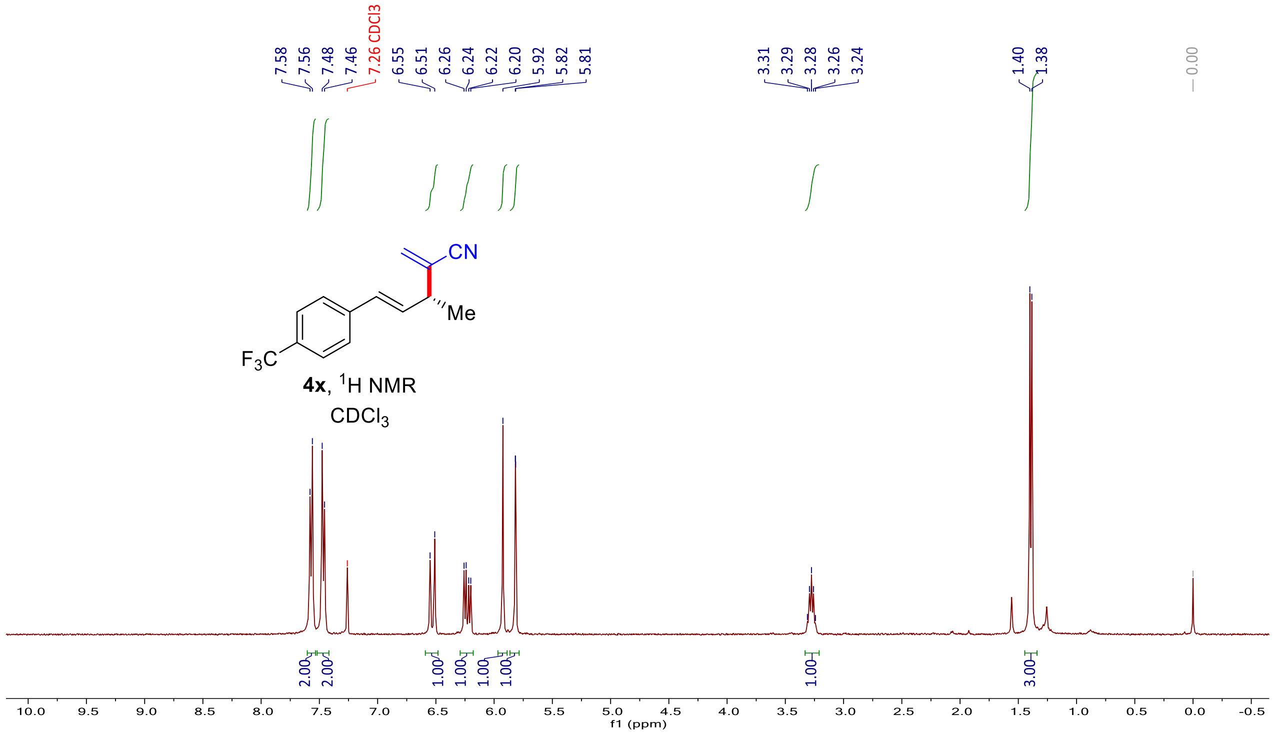

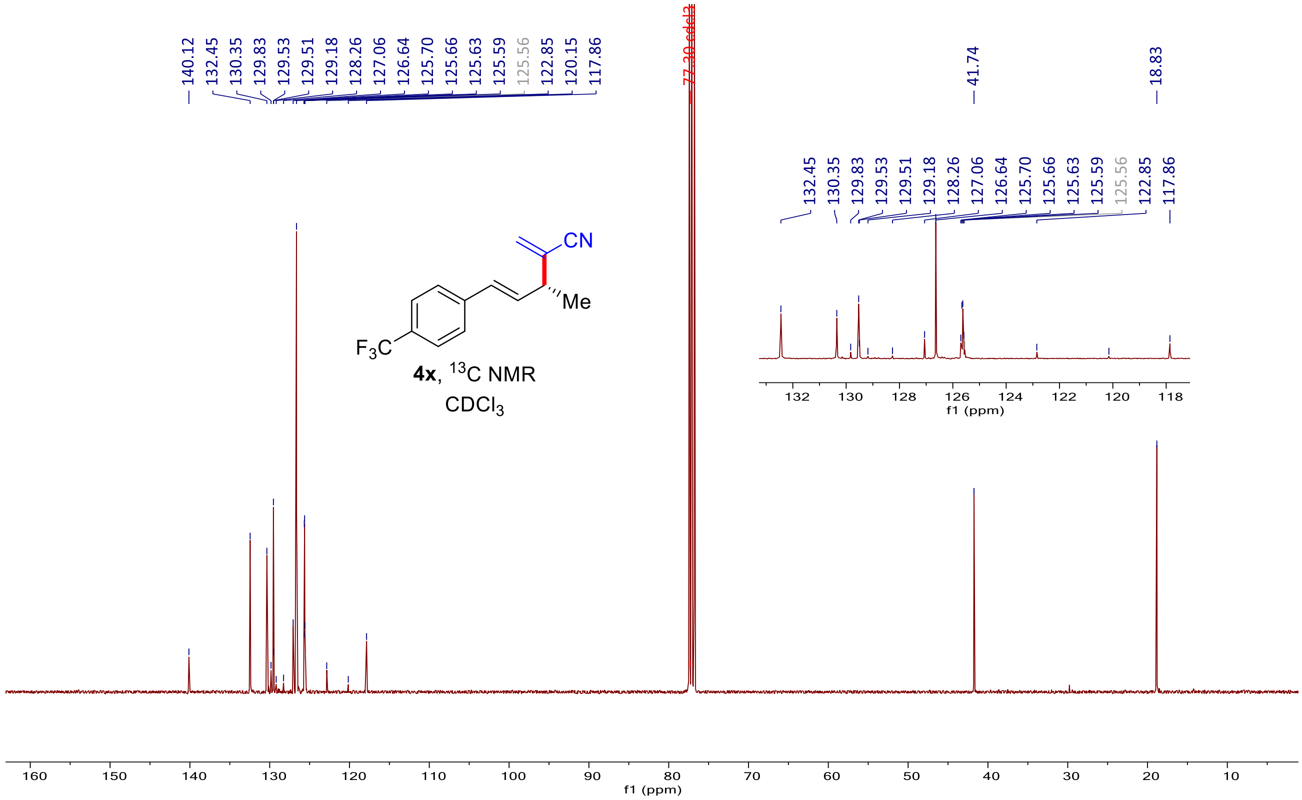

Supplementary Figure 44. ^{1}H NMR and ^{13}C NMR spectra of compound **4x**

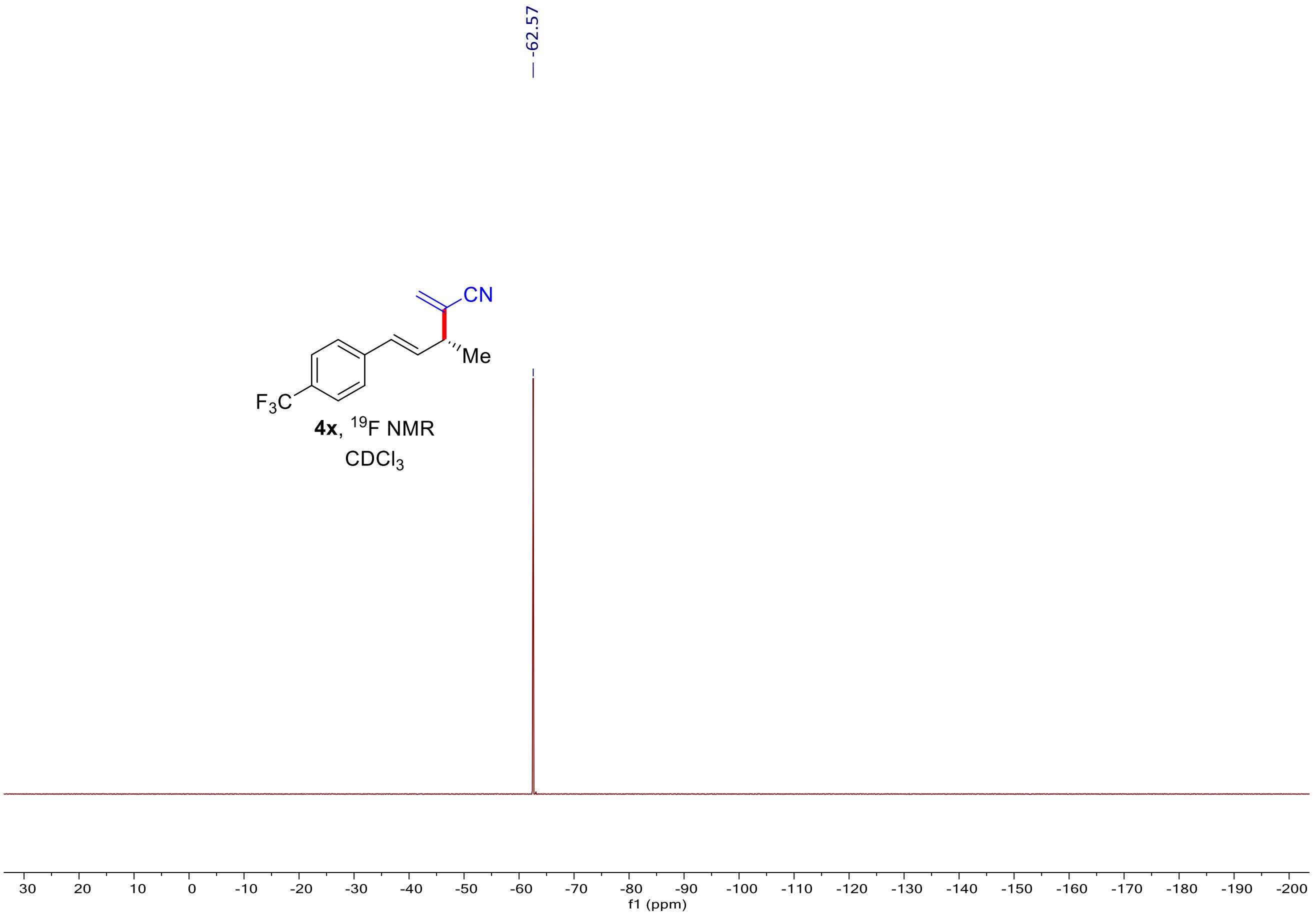

Supplementary Figure 45. ^{19}F NMR spectra of compound **4x**

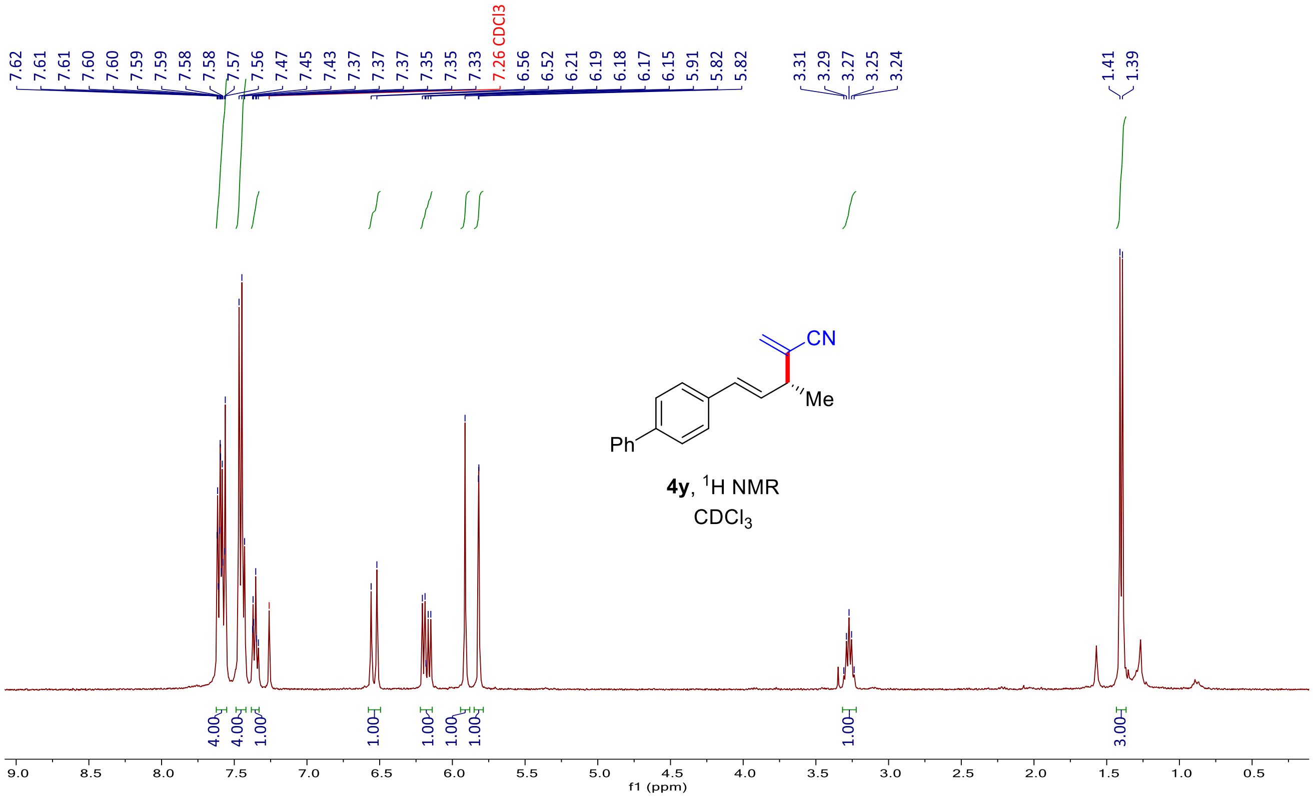

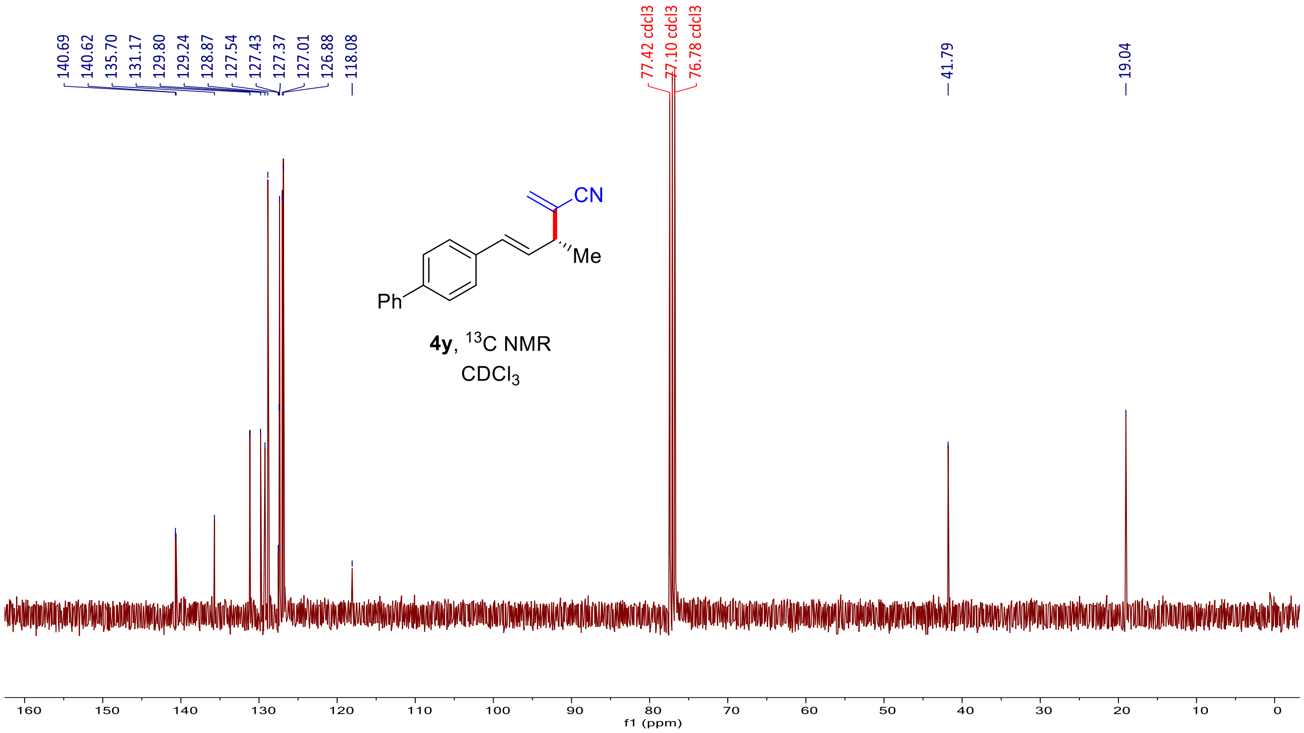

Supplementary Figure 46. ^{1}H NMR and ^{13}C NMR spectra of compound **4y**

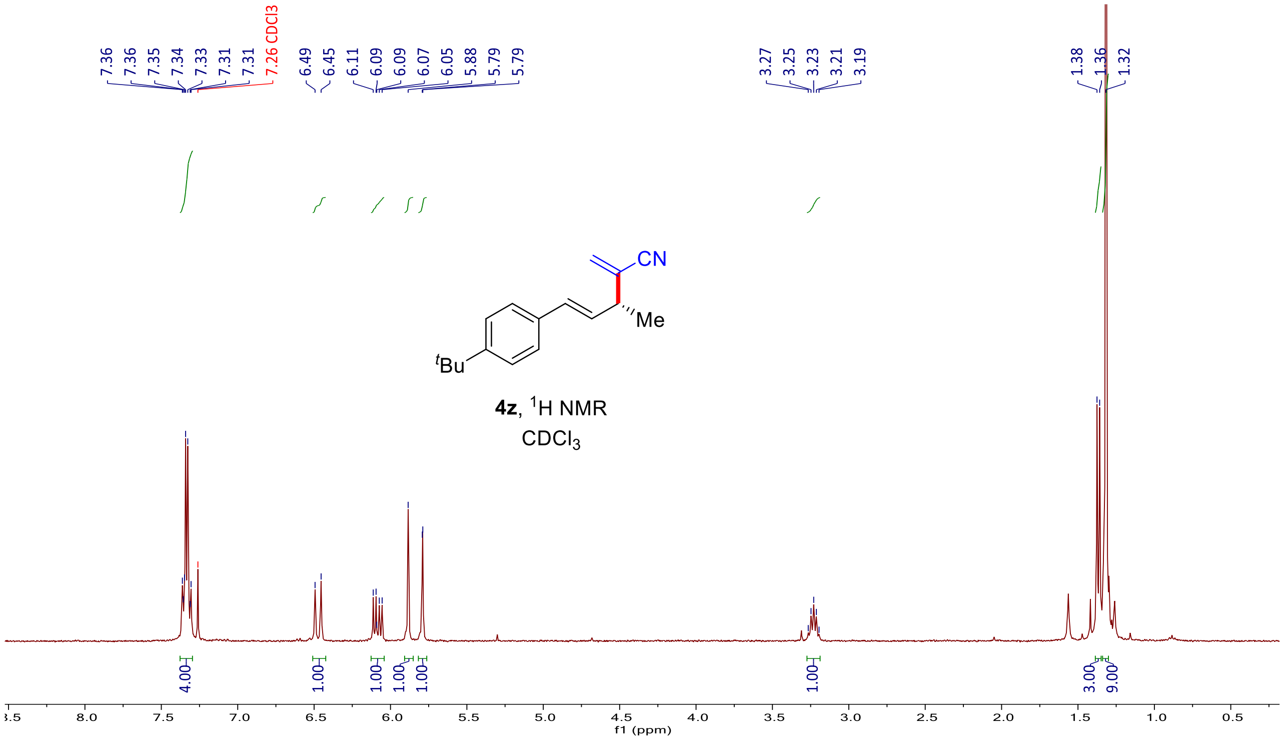

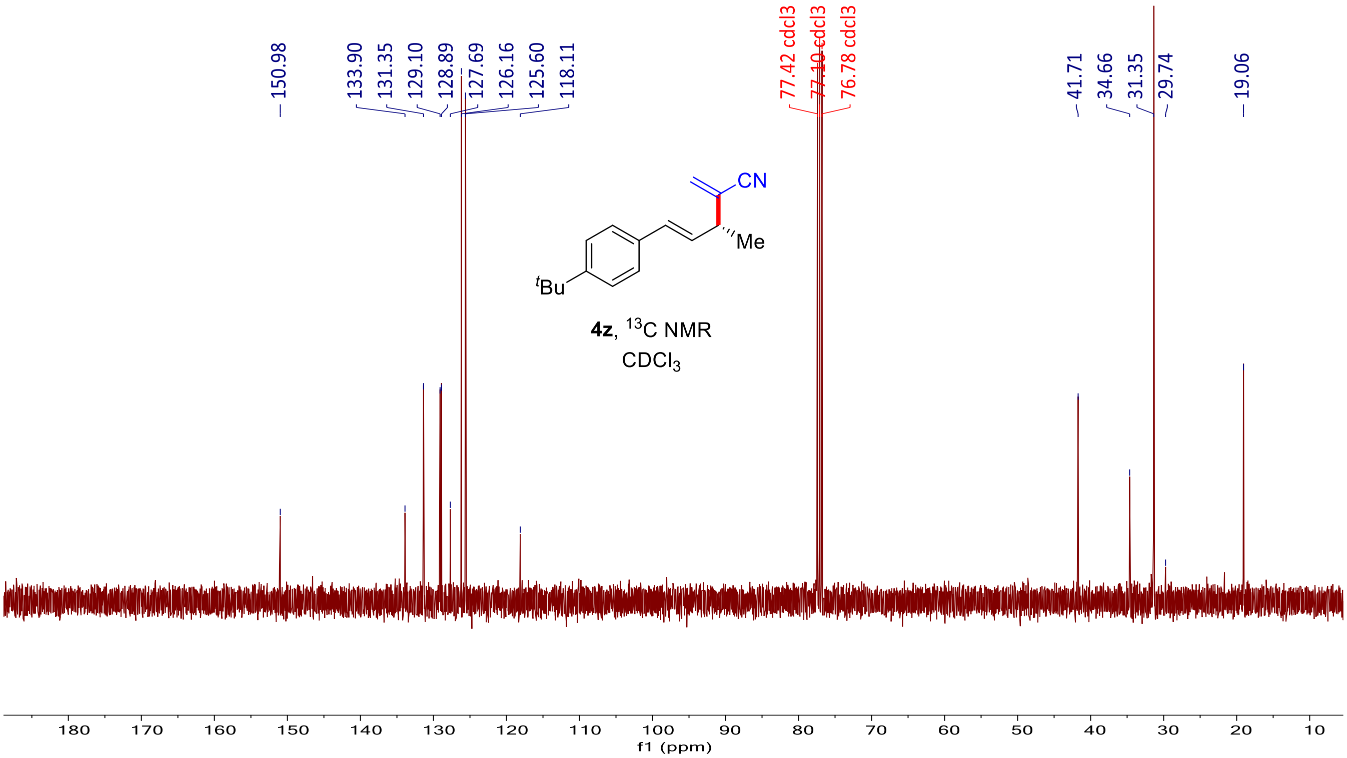

Supplementary Figure 47. ^{1}H NMR and ^{13}C NMR spectra of compound **4z**

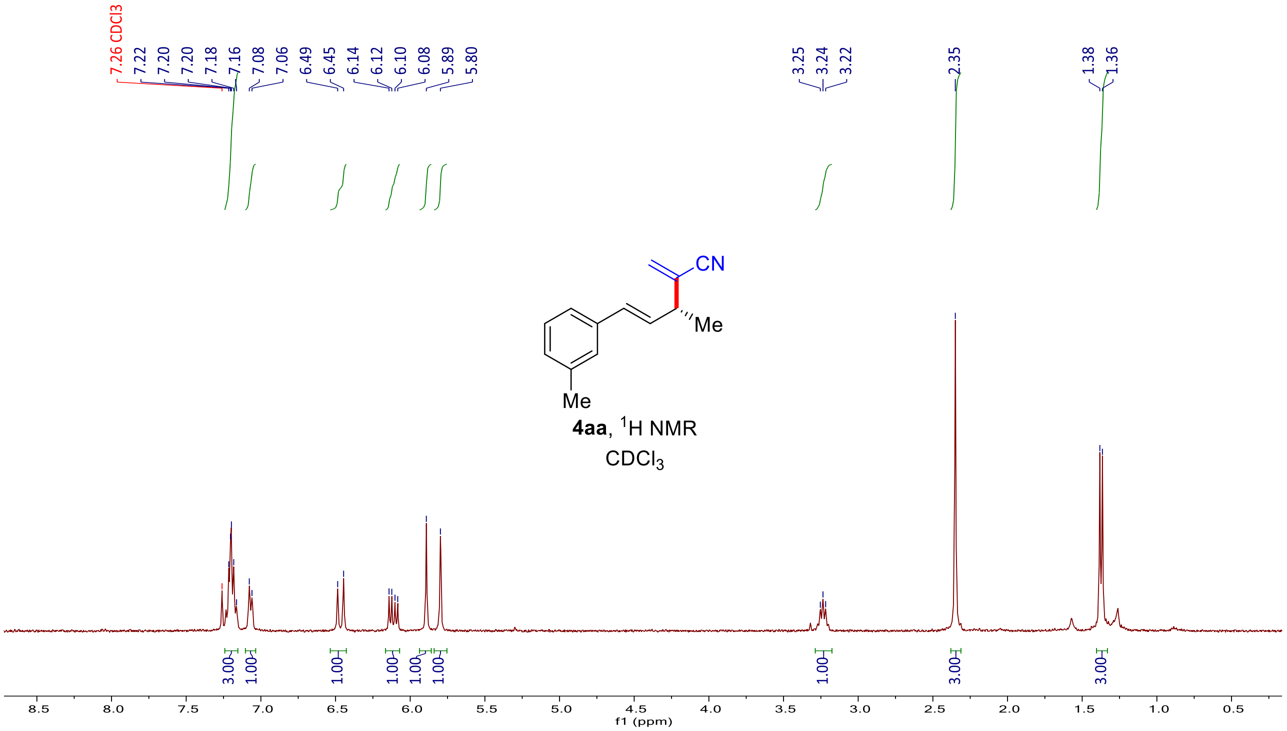

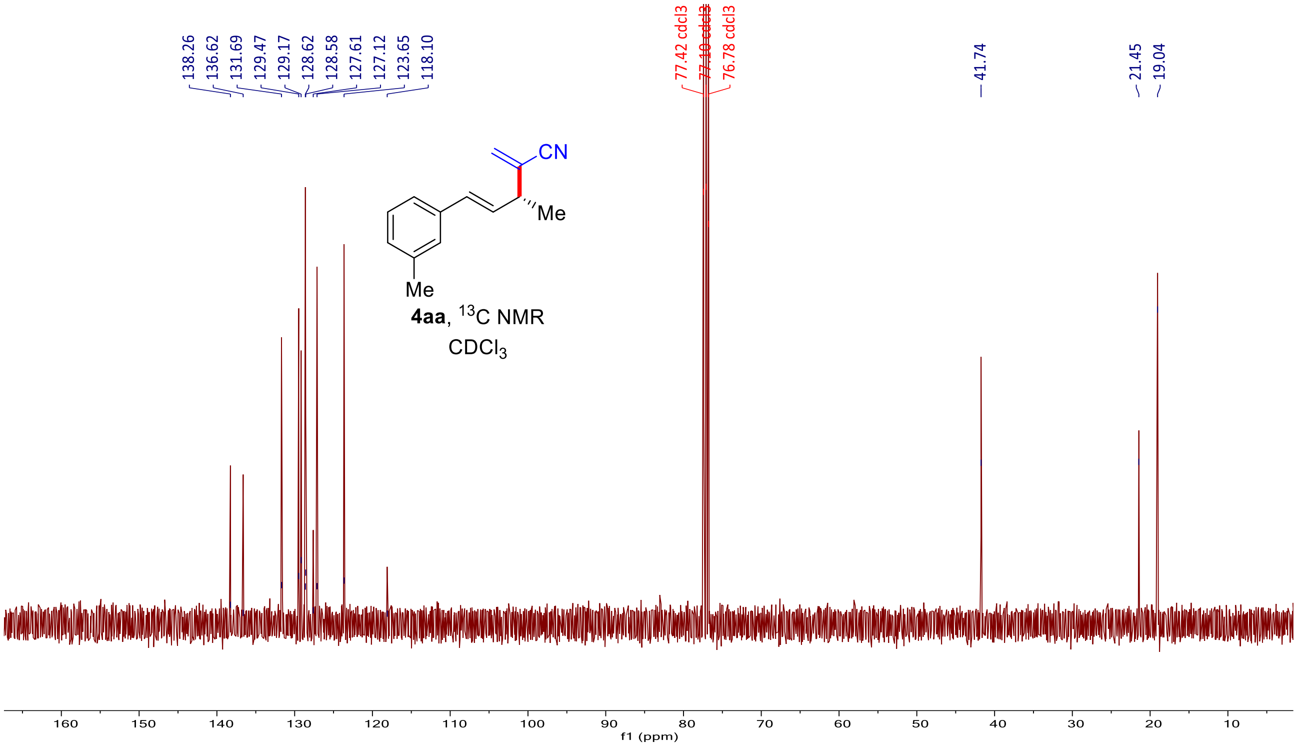

Supplementary Figure 48. ^{1}H NMR and ^{13}C NMR spectra of compound **4aa**

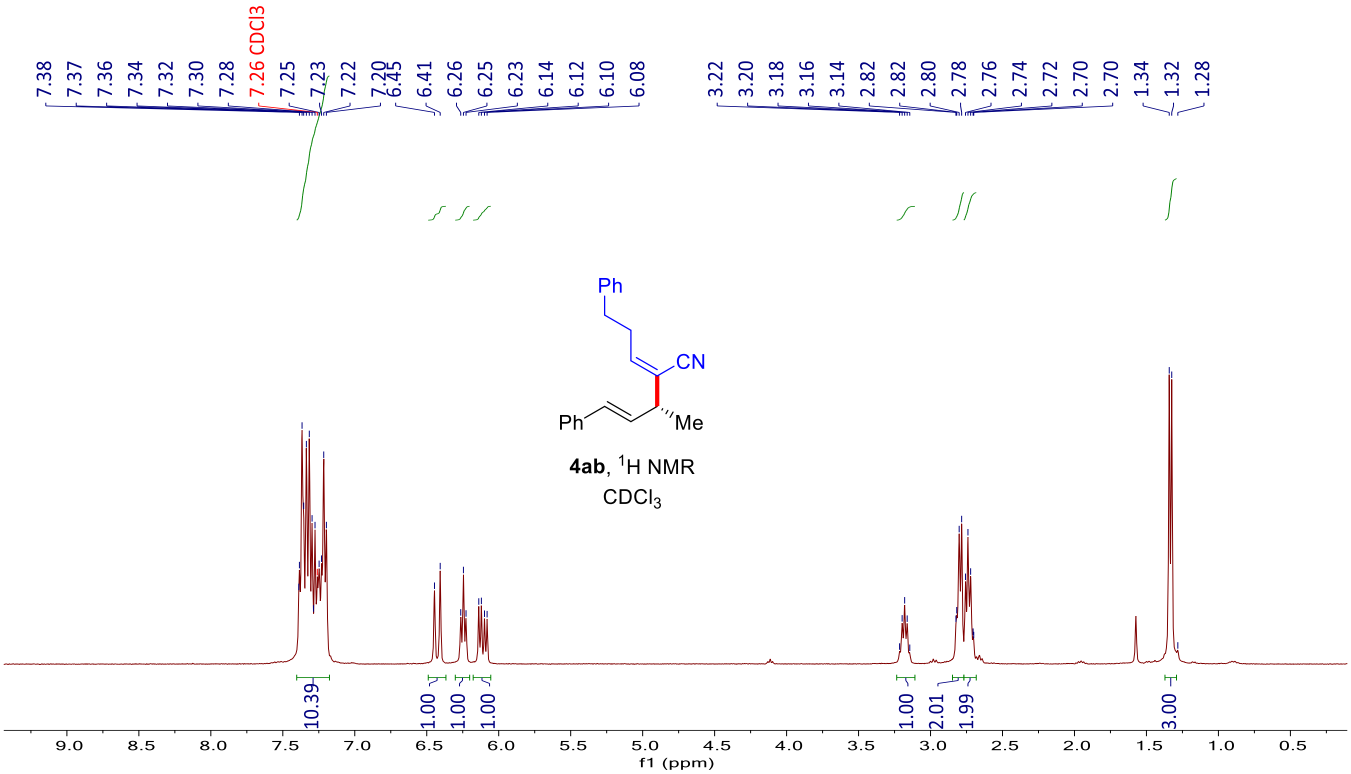

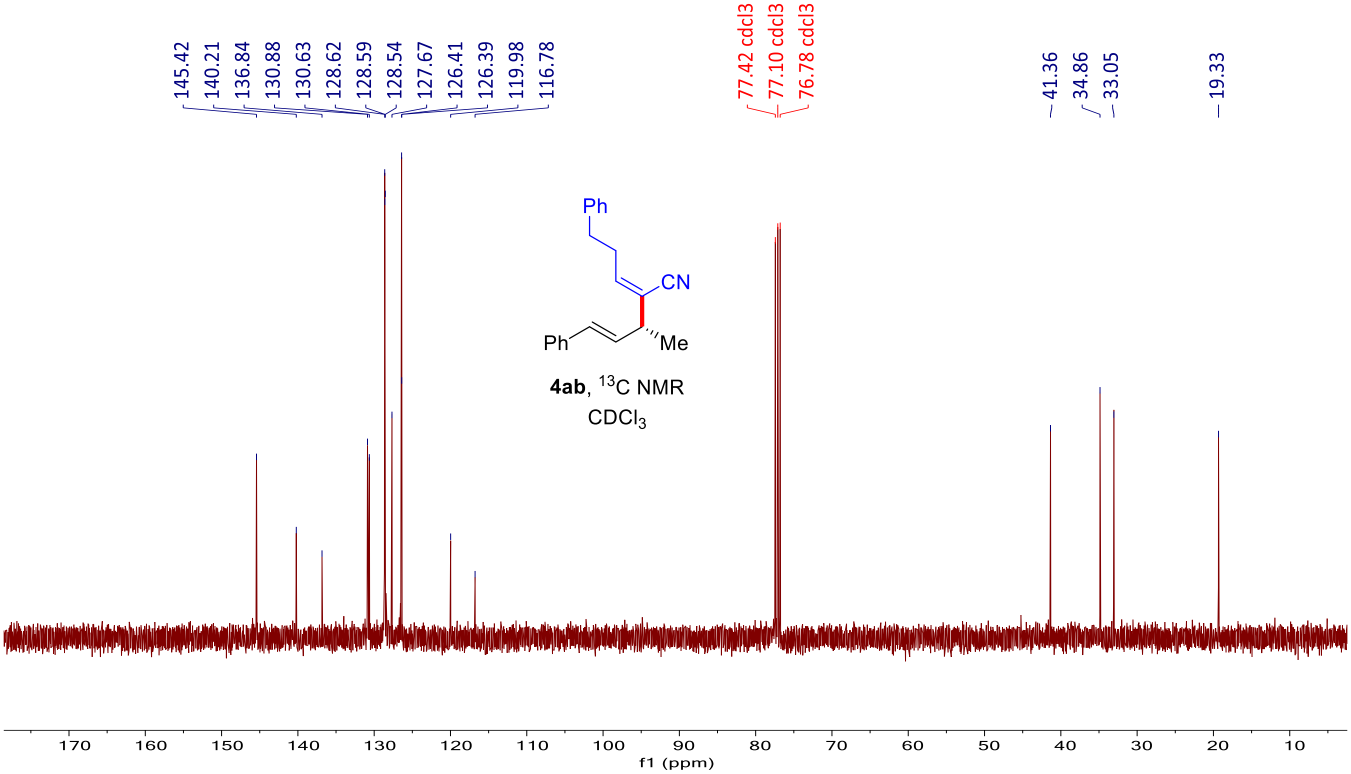

Supplementary Figure 49. ^{1}H NMR and ^{13}C NMR spectra of compound **4ab**

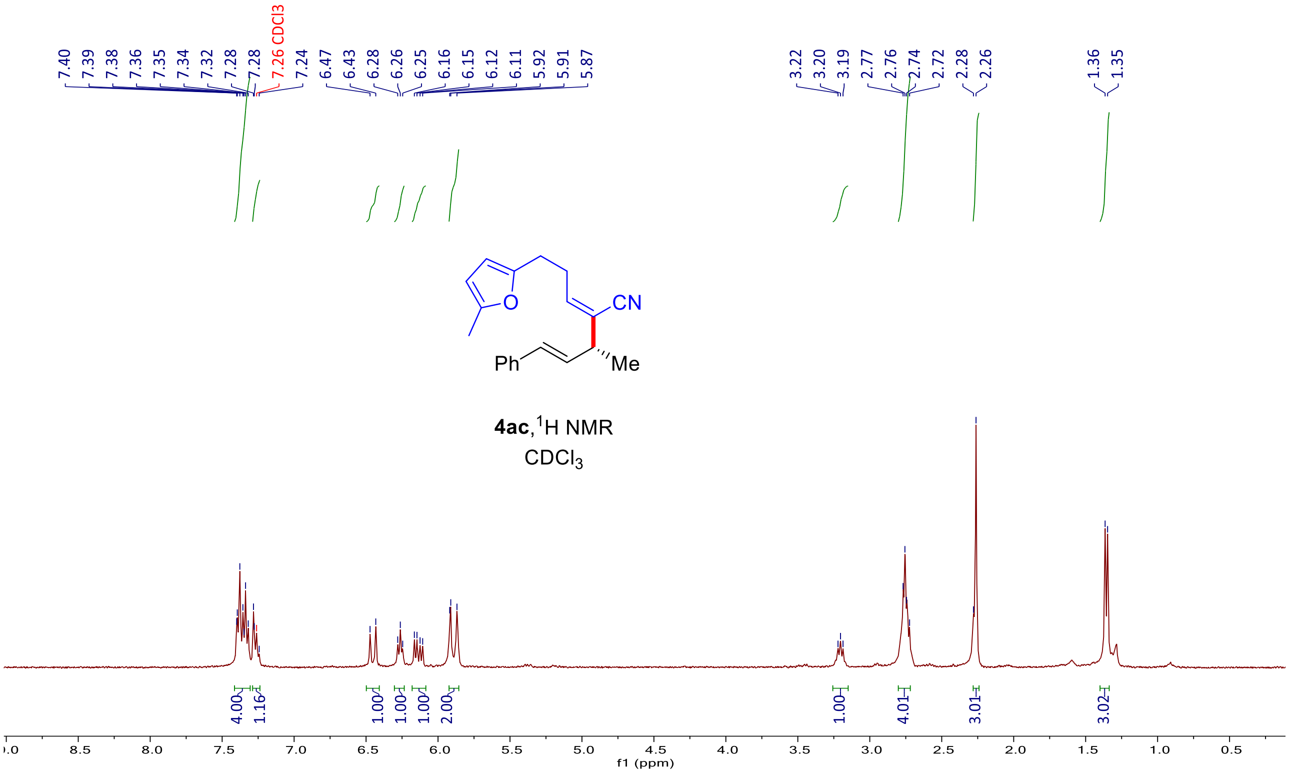

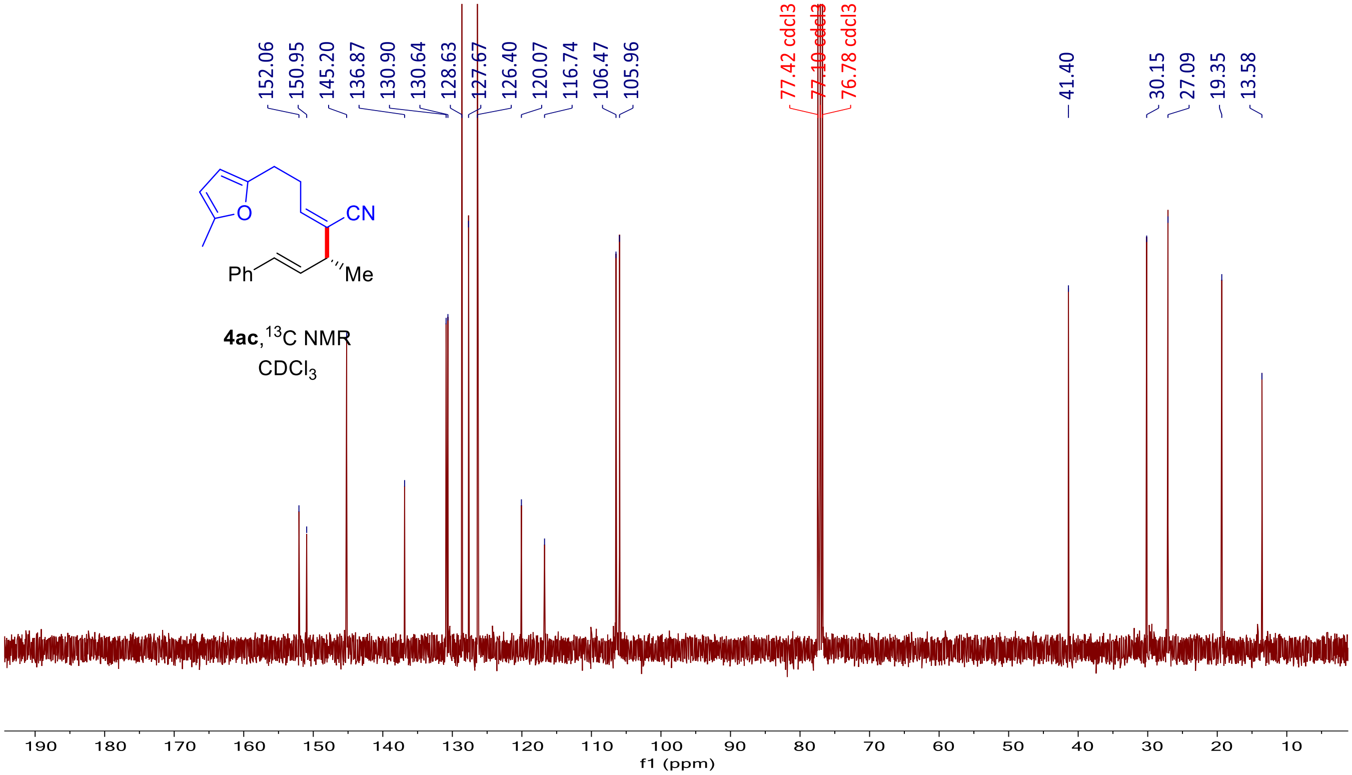

Supplementary Figure 50. ^{1}H NMR and ^{13}C NMR spectra of compound **4ac**

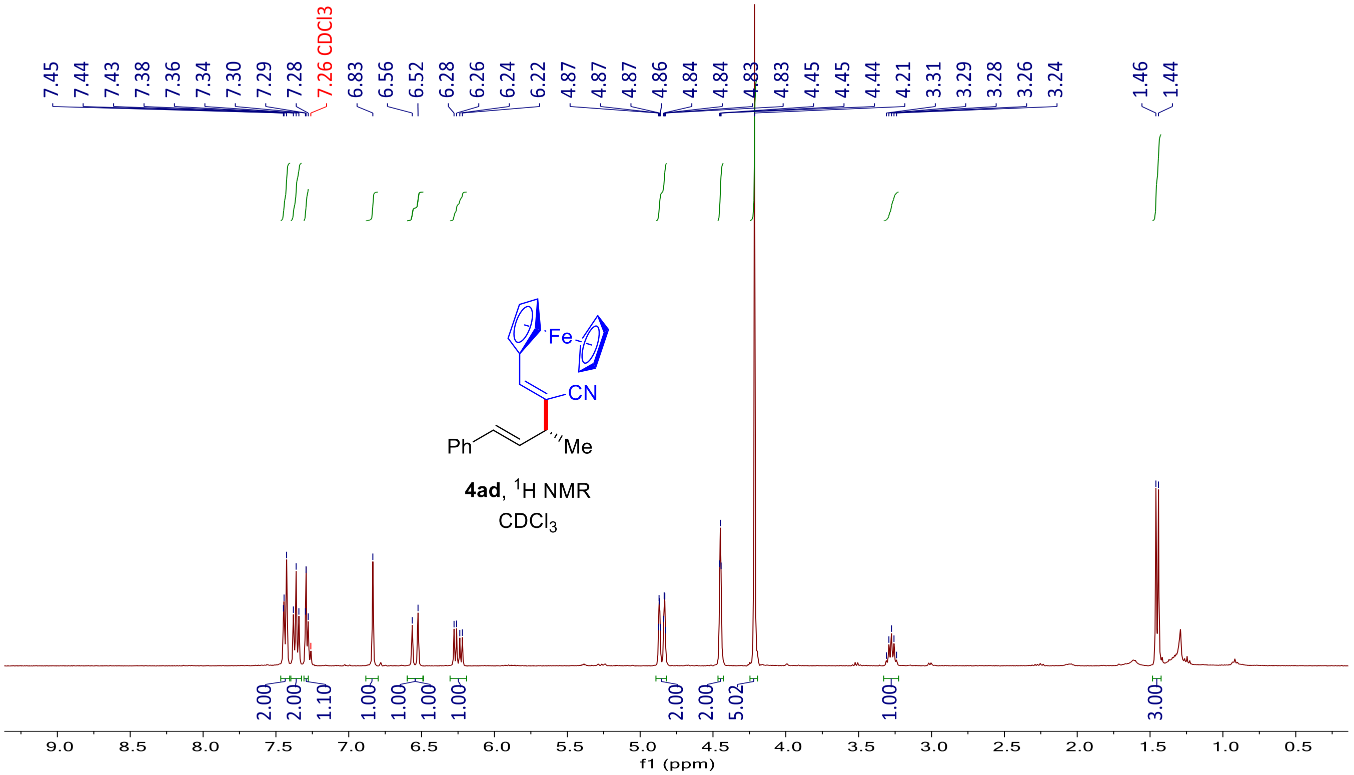

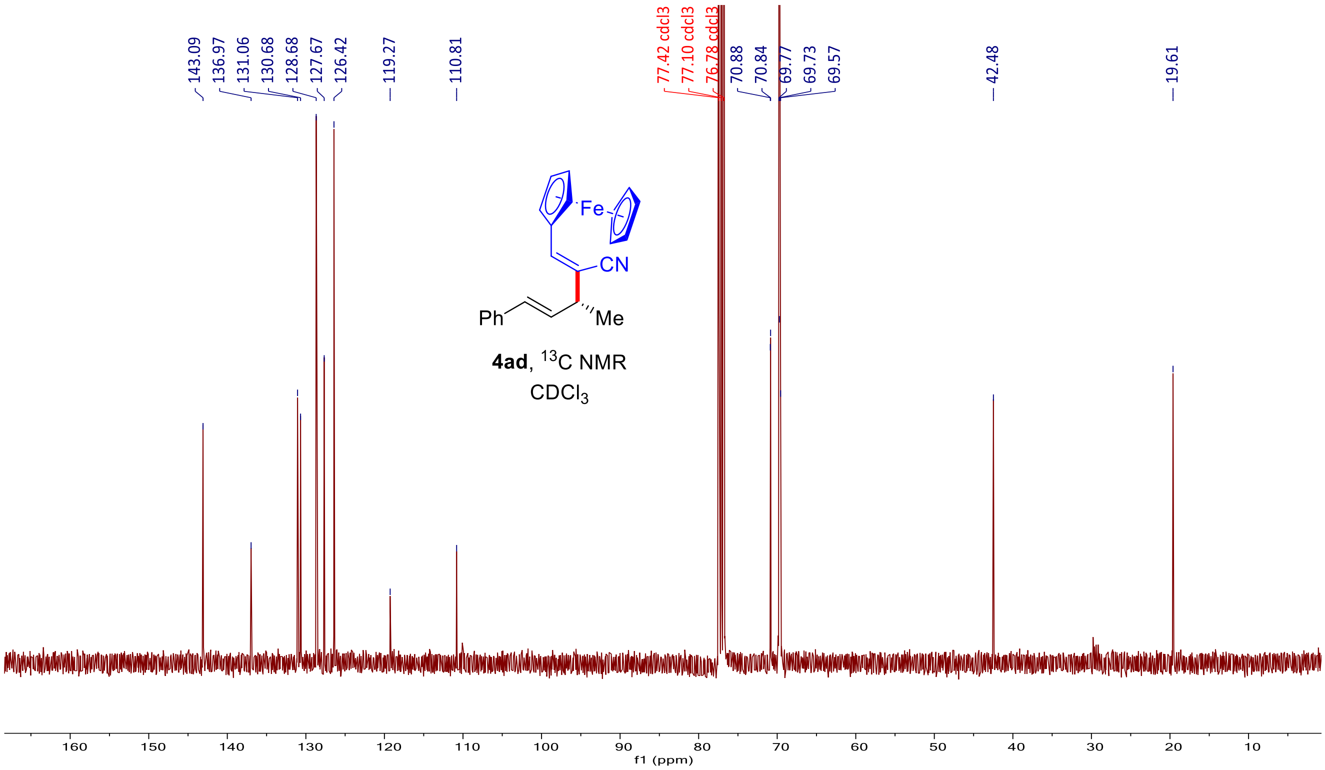

Supplementary Figure 51. ^{1}H NMR and ^{13}C NMR spectra of compound **4ad**

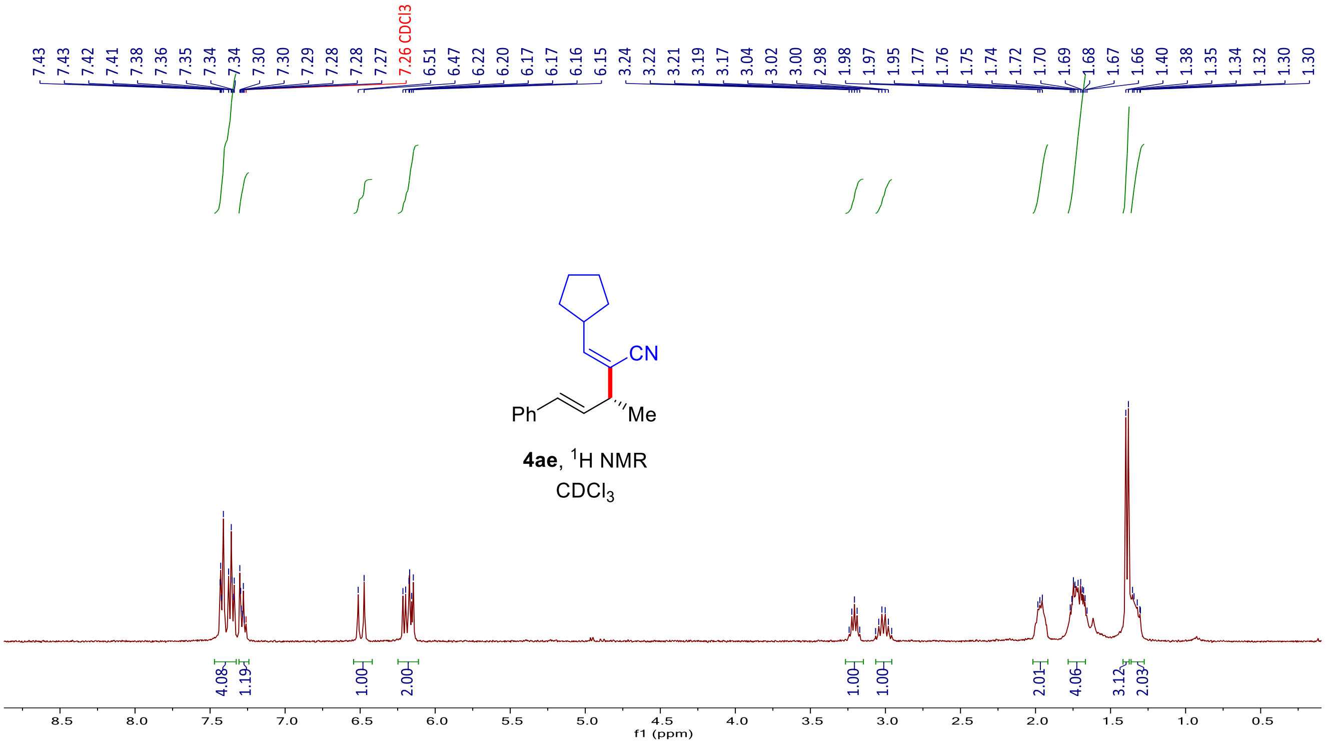

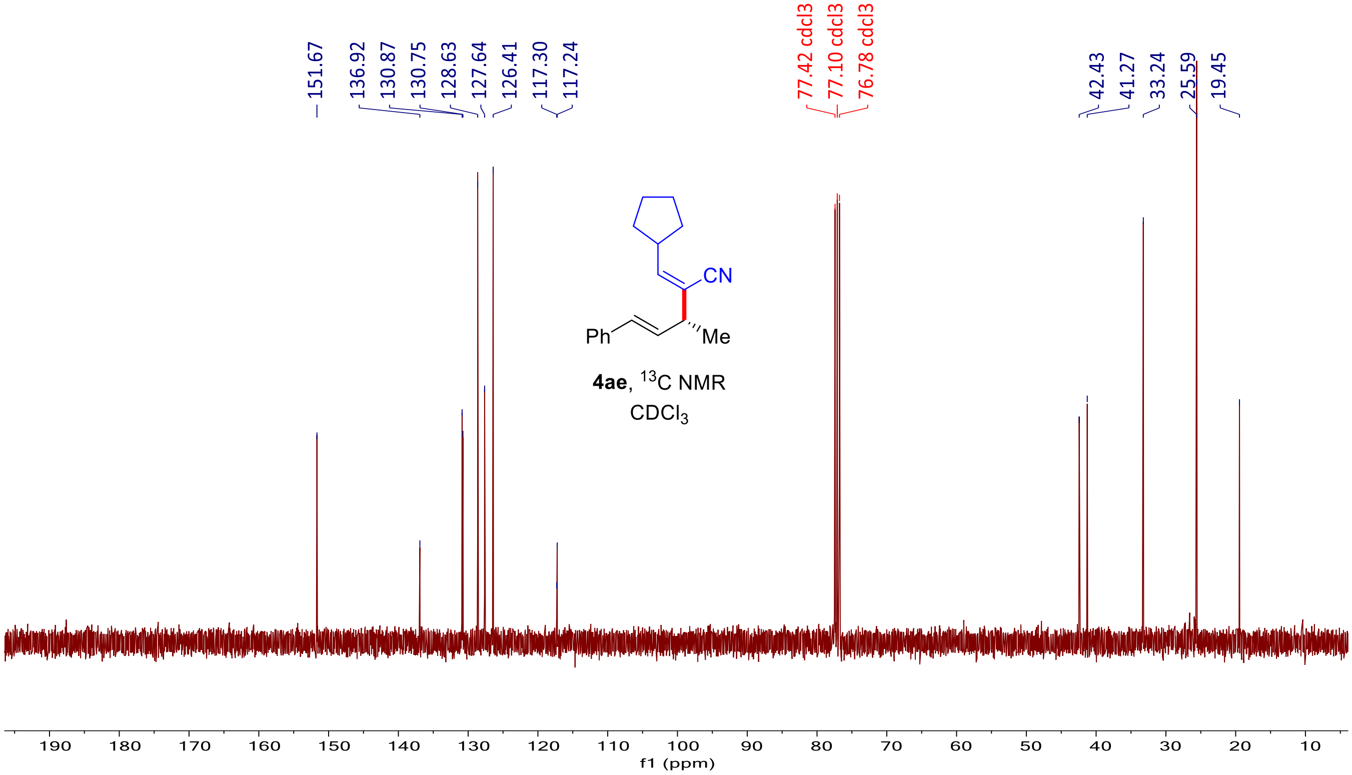

Supplementary Figure 52. ^{1}H NMR and ^{13}C NMR spectra of compound **4ae**

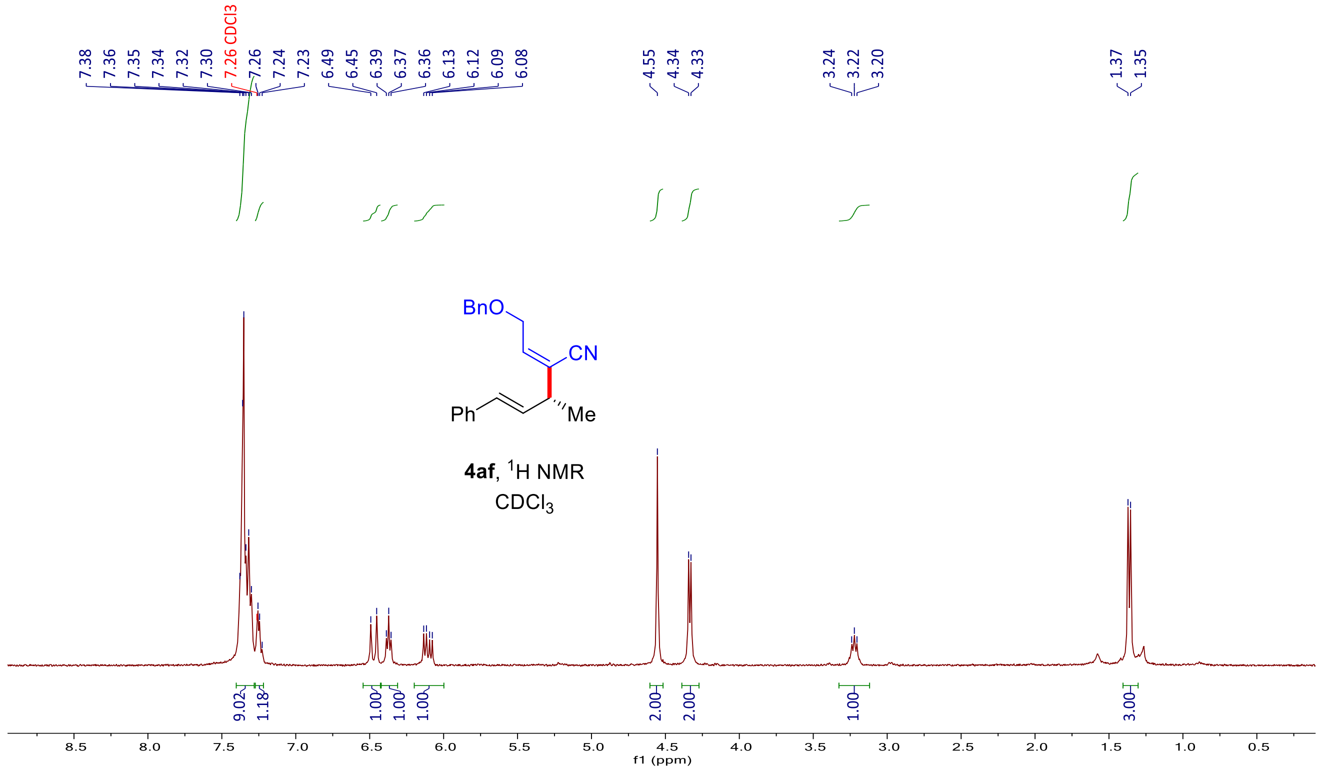

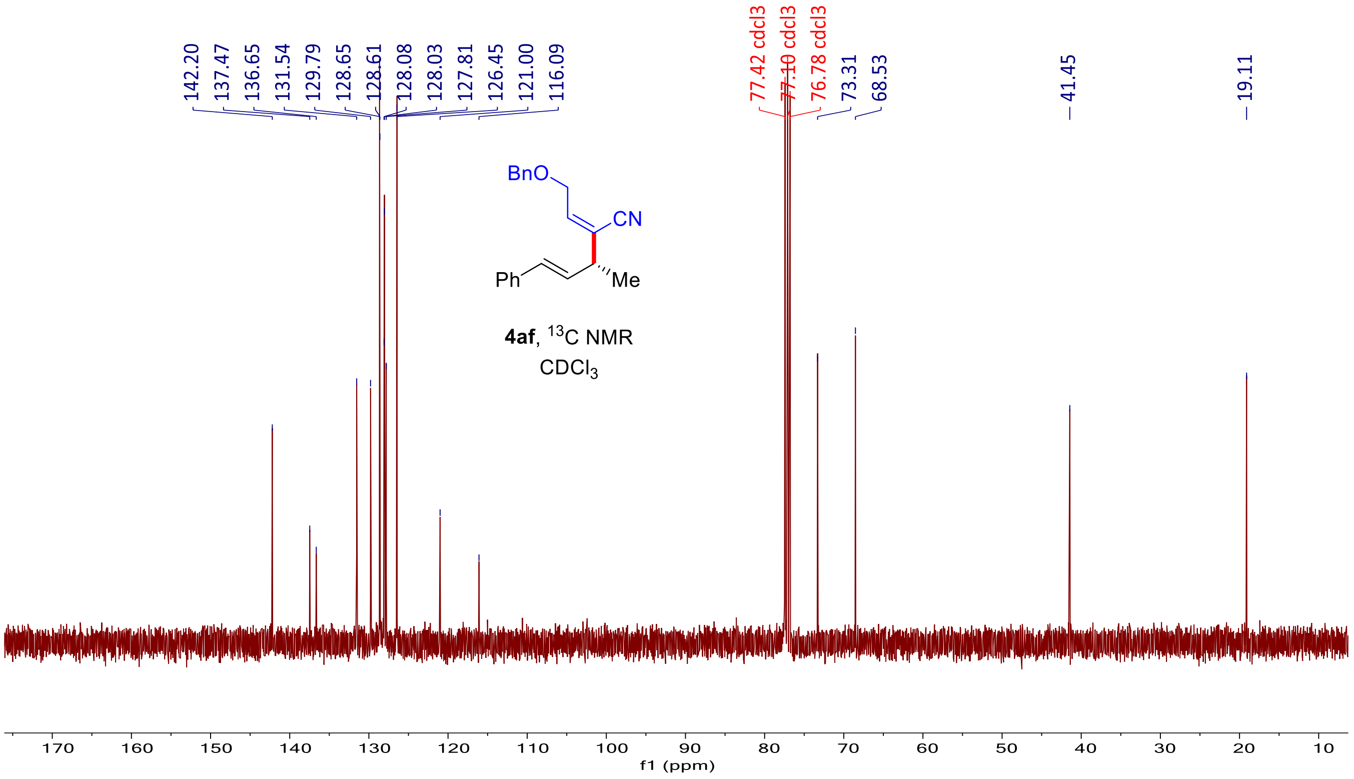

Supplementary Figure 53. ^{1}H NMR and ^{13}C NMR spectra of compound **4af**

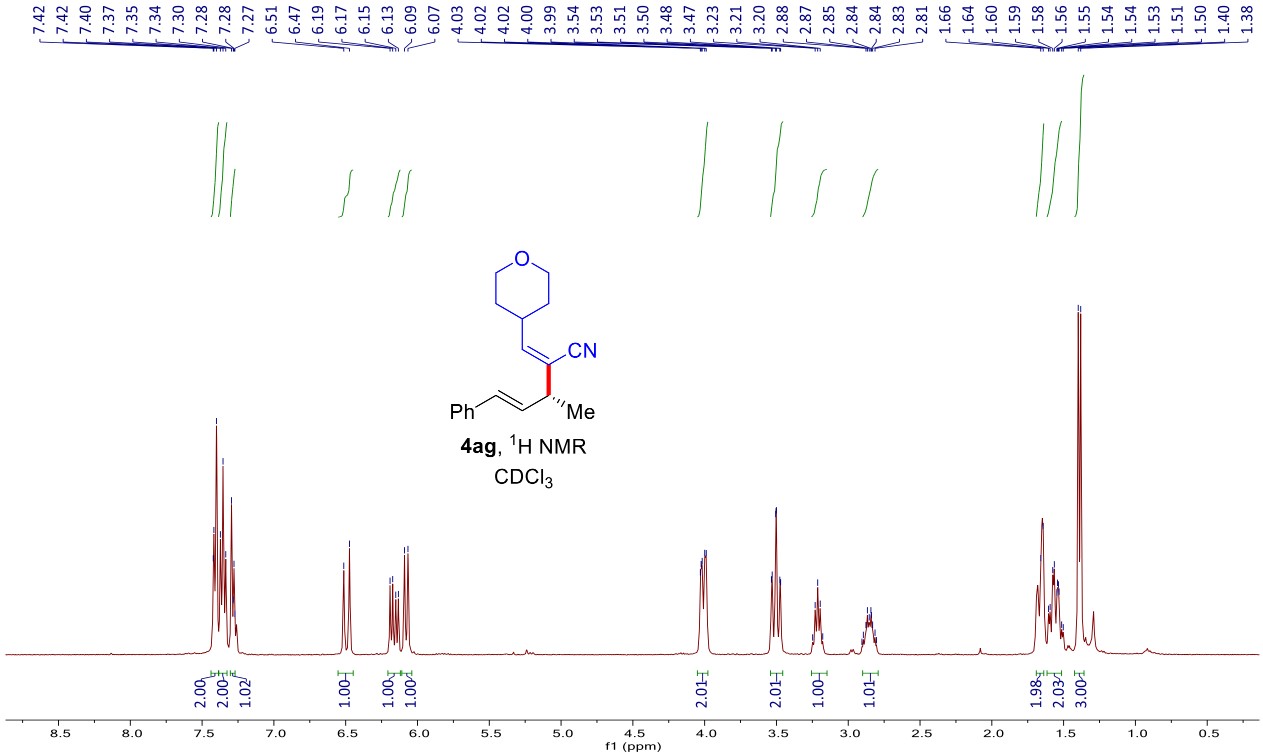

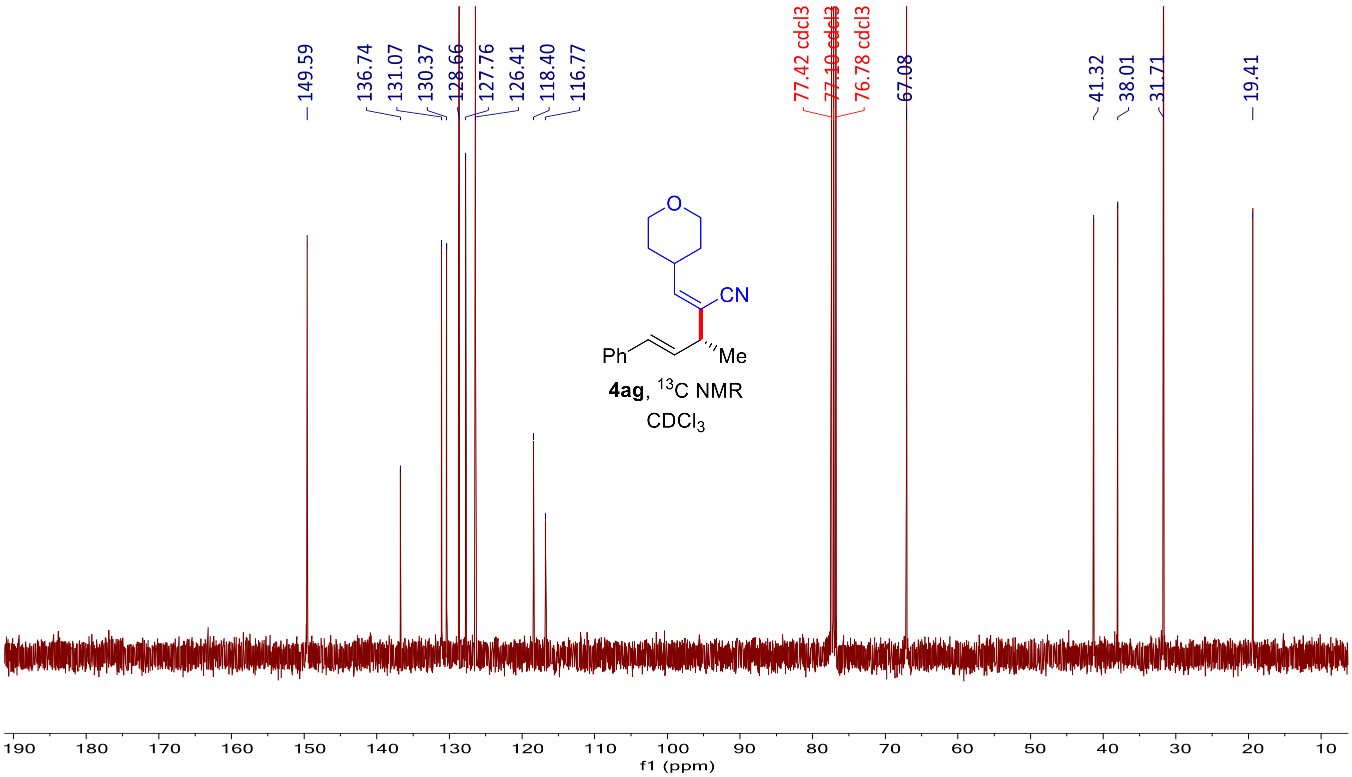

Supplementary Figure 54. ^{1}H NMR and ^{13}C NMR spectra of compound **4ag**

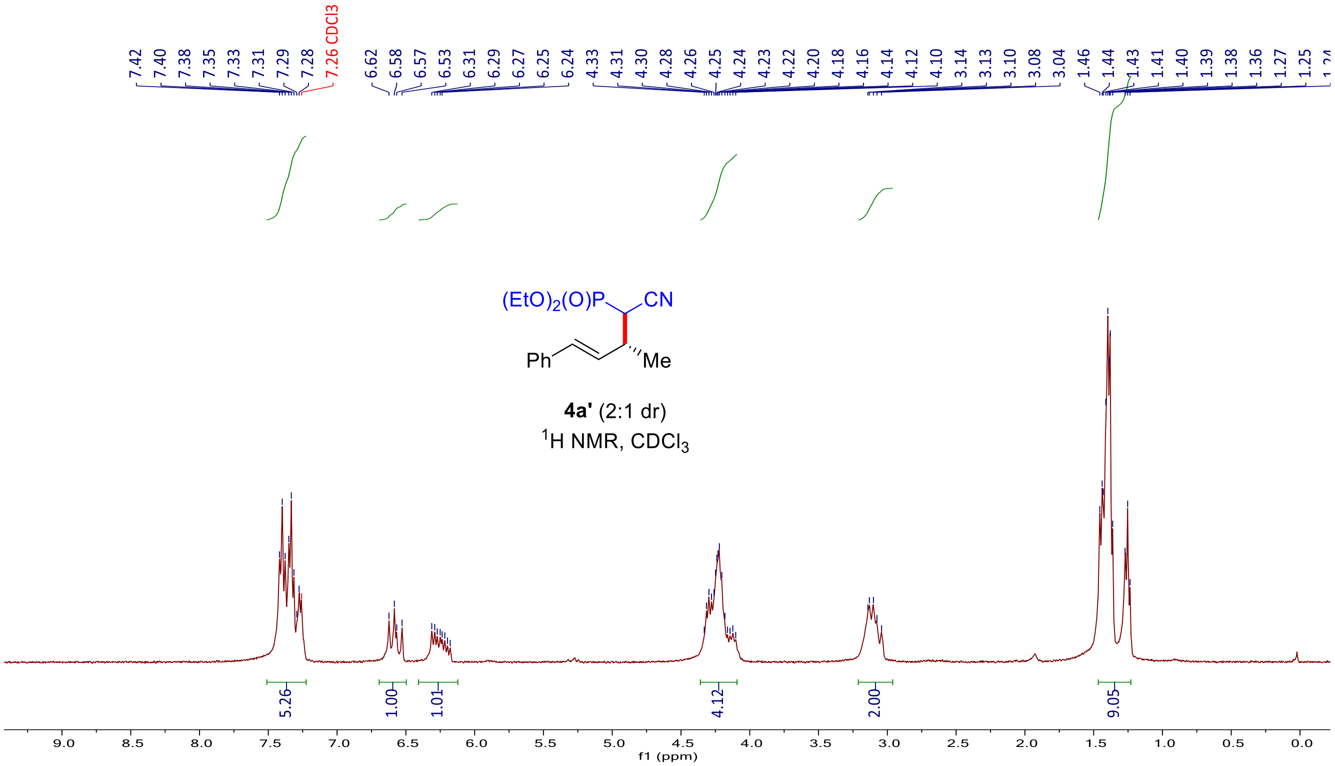

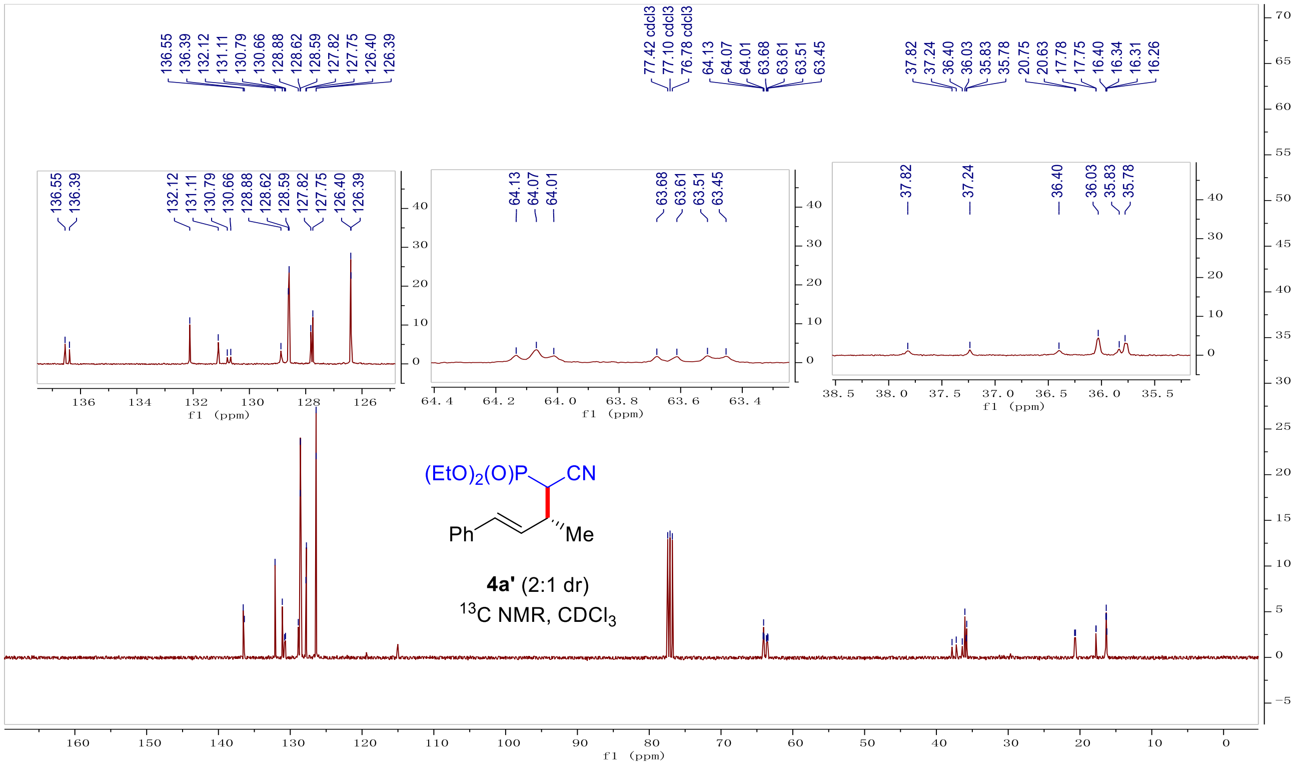

Supplementary Figure 55. ^{1}H NMR and ^{13}C NMR spectra of compound **4a'**

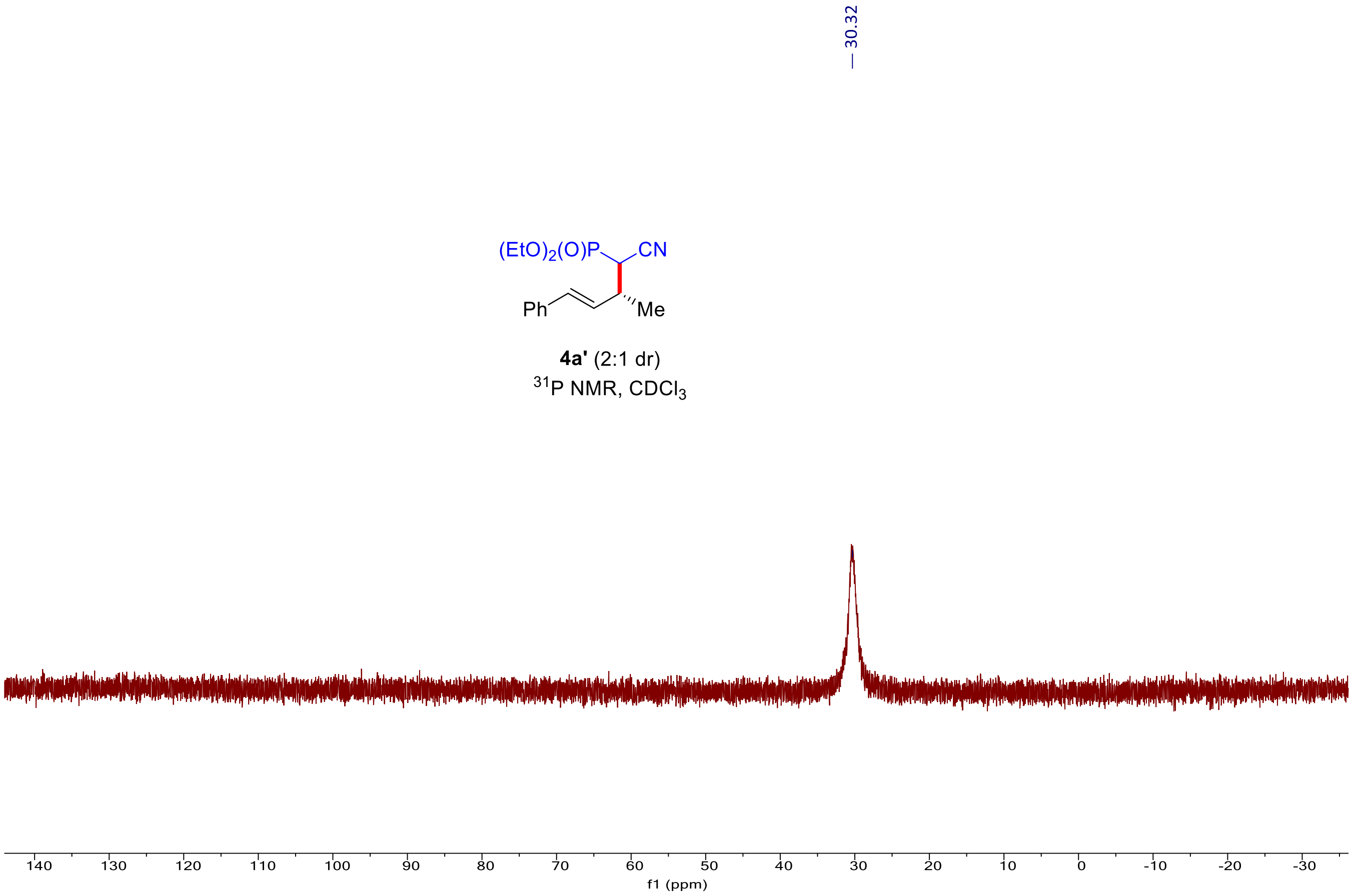

Supplementary Figure 56. ^{31}P NMR spectra of compound **4a’**

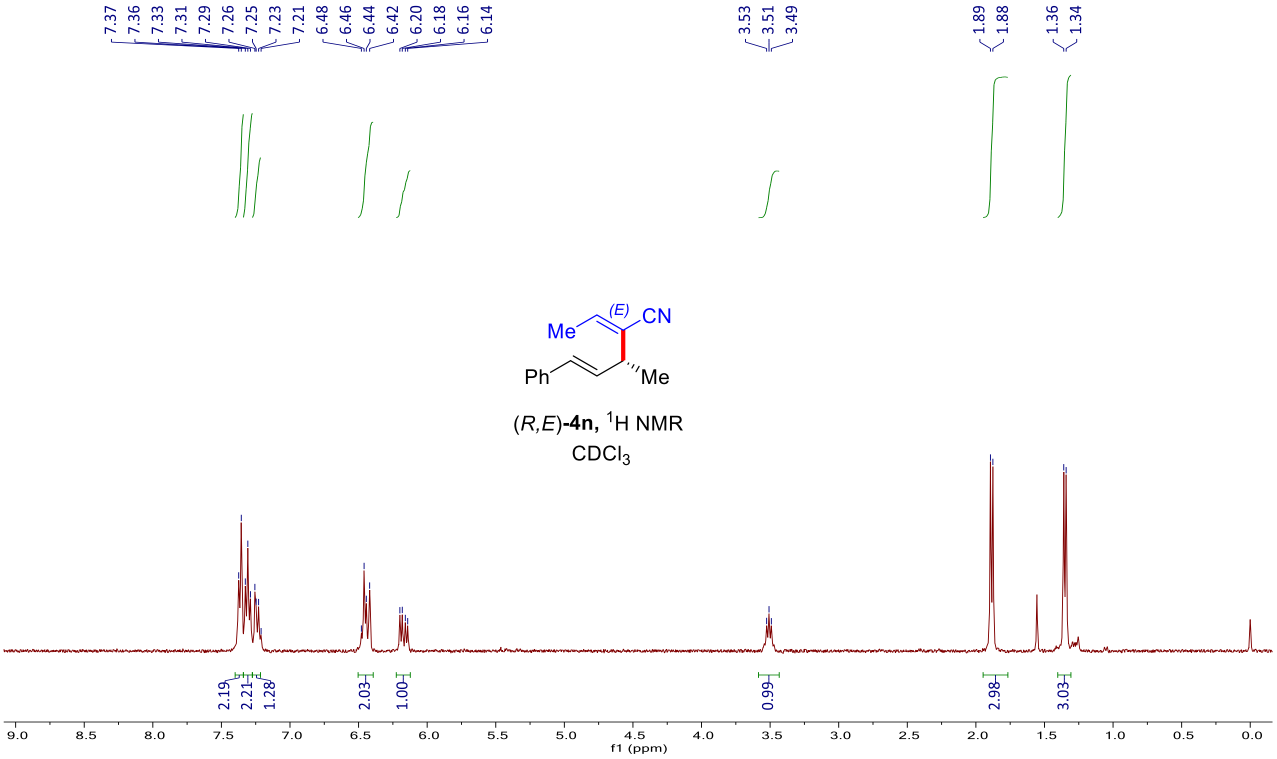

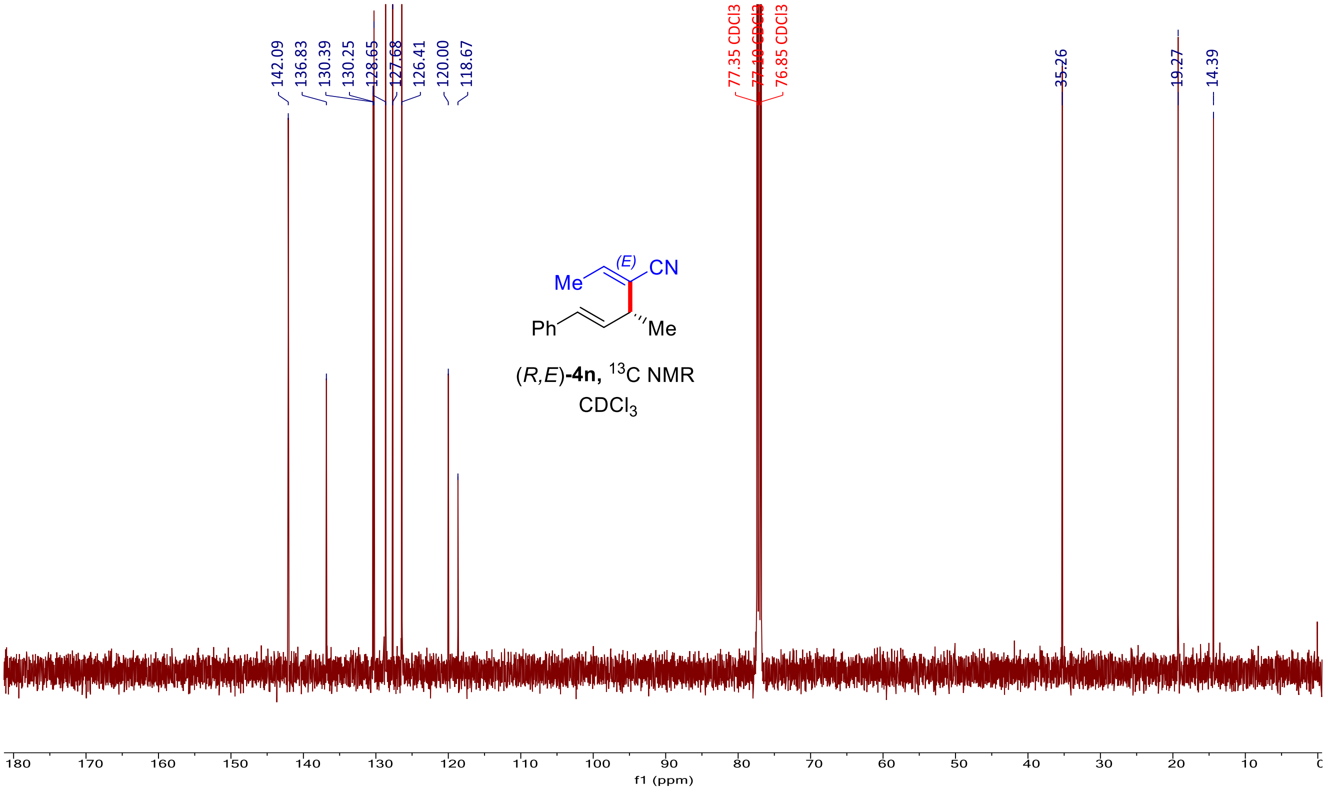

Supplementary Figure 57. ^{1}H NMR and ^{13}C NMR spectra of compound (*R*,*E*)-**4n**

Supplementary References

1. Zhang, P.-L.; Zhou, Z.-L.; Zhang, R.-M.; Zhao, Q.; Zhang, C. *Chem. Commun*. **2020**, *56*, 11469-11472.
2. Yang, S.-Q.; Han, A.-J.; Liu, Y.; Tang, X.-Y.; Lin G.-Q.; He, Z.-T. *J. Am. Chem. Soc.* **2023***, 145,* 3915−3925.
3. Zhang, R.-M.; Li, Q.-F.; Zhang, M.; Chai, S.-D.; Duan, Y.-Q.; Su, J.-F.; Zhao, Q.; Zhang, C. *Chem. Commun.* **2020**, *56*, 13551-13554.
4. Röse, P.; Emge, S.; Yoshida, J.-I.; Hilt, G. *Beilstein J. Org. Chem.* **2015**, *11*, 174–183.
5. Tortajada, A.; Ninokata, R.; Martin, R. *J. Am. Chem. Soc.* **2018**, *140*, 2050–2053.
6. Phadke, N.; Findlater, M. *Molecules* **2015**, *20*, 20195-20205.
